# Supplementary material for: Uro-pathogens: Multidrug resistance and associated factors of community-acquired UTI among HIV patients attending antiretroviral therapy in Dessie Comprehensive Specialized Hospital, Northeast Ethiopia
Source: PLoS One. 2024 May 31;19(5):e0296480. doi: 10.1371/journal.pone.0296480 (PMC11142584; doi:10.1371/journal.pone.0296480)
Supplement: Attachment — Submitted filename: PONE-D-22-31717.pdf [file pone.0296480.s001.pdf]

PLOS ONE

# Bacterial and fungal profile, multidrug resistance profile of bacterial pathogens and associated risk factors of community-acquired urinary tract infection among HIV positive patients attending Antiretroviral Therapy Clinics of Northeast Ethiopia.

--Manuscript Draft--

| | |
|---|---|
| Manuscript Number: | PONE-D-22-31717 |
| Article Type: | Research Article |
| Full Title: | Bacterial and fungal profile, multidrug resistance profile of bacterial pathogens and associated risk factors of community-acquired urinary tract infection among HIV positive patients attending Antiretroviral Therapy Clinics of Northeast Ethiopia. |
| Short Title: | Bacterial and fungal profile, drug resistance profile of bacteria, risk factors of urinary tract infection |
| Corresponding Author: | Mihret Tilahun, MSc<br>Wollo University<br>Dessie, ETHIOPIA |
| Keywords: | community-acquired UTI; Multidrug resistance; Ethiopia |
| Abstract: | Background<br><br>Urinary tract infections (UTI) are one of the most common bacterial and fungal infections in humans, occurring both in the community and in health-care settings. UTI is a significant health issue among HIV-infected patients. Antimicrobial resistance is now widespread and poses a serious clinical risk.<br><br>Objective<br><br>This study investigated bacterial and fungal profile, multidrug resistance profile of bacterial pathogens and associated risk Factors of Community acquired urinary tract infection among HIV positive patients attending Antiretroviral Therapy Clinics of Northeast Ethiopia.<br><br>Methods<br><br>Institutional based cross-sectional study was conducted in Dessie Referral Hospital, from February 1, 2021 to March 30, 2021. Socio-demographical and clinical data were collected using structured questionnaires from community acquired urinary tract infection suspected HIV-Positive patients. About 10 ml Clean-catch midstream were collected and inoculated into Blood agar, MacConkey and Cysteine lactose electrolyte deficient. Biochemical tests were performed to identify isolates and antimicrobial susceptibility pattern was performed on disc diffusion techniques. Data were entered and analyzed using SPSS version-25. Descriptive statistics such as tables and bar graphs were used to summarize the findings. Logistic regression analysis was performed and the adjusted odds ratio (AOR) with their 95% confidence interval (CI) was used as statistically significant associations.<br><br>Results<br><br>Among the total 346 study participants, 180 (52.04%) were females. The overall 26.6% (92/346) of which 21.67% (75/346) were bacterial pathogens, while 4.9% (17/346) were fungal pathogens. From a total of 75 bacteria isolates 51(68%) were Gram-negative bacteria. The most commonly isolated Gram-negative bacteria were E. coli (21.33%) followed K. pneumoniae (14.67%). In addition, From the 17 yeasts isolate of fungi 47.1% (8/17) were represented by C. tropicalis. The overall prevalence of MDR bacteria was 61(81.3%). About 13 (81.3%) of E. coli, 9(81.8%) of K. pneumoniae, 8(80%) of Enterococcus species, 7 (77.8%) of P. aeruginosa, and CoNs 7(87.5%) were the most frequently exhibited MDR. age, gender, occupation, educational status, current CD4+ count (cells/mm3), previous hospitalization and diabetes status, history of catheterizations, and urinary tract urgency had a statistically significant association with significant bacteriuria. |

Powered by Editorial Manager® and ProduXion Manager® from Aries Systems Corporation

| | |
|---|---|
| Order of Authors: | Mihret Tilahun, MSc |
| | Mesfin Fiseha |
| | Mihreteab Alebachew |
| | Alemu Gedefie |
| | Endris Ebrahim |
| | Melkam Tesfaye |
| | Melaku Ashagrie Belete |
| | Abdurahaman Seid |
| | Daniel Gebretsadik |
| | Wondmagegn Demsiss |
| | Bekele Sharew, PhD |
| | Agumas Shibabaw, PhD |
| | Habtamu Mekonnen |
| | Tewodros Dessie |
| Additional Information: | |
| Question | Response |
| **Financial Disclosure**<br><br>Enter a financial disclosure statement that describes the sources of funding for the work included in this submission. Review the submission guidelines for detailed requirements. View published research articles from *PLOS ONE* for specific examples.<br><br>This statement is required for submission and **will appear in the published article** if the submission is accepted. Please make sure it is accurate. | All the authors are under developing countries and no extrena funding was obtained |

| | |
|---|---|
| **Unfunded studies**<br>Enter: *The author(s) received no specific funding for this work.*<br><br>**Funded studies**<br>Enter a statement with the following details:<br>• Initials of the authors who received each award<br>• Grant numbers awarded to each author<br>• The full name of each funder<br>• URL of each funder website<br>• Did the sponsors or funders play any role in the study design, data collection and analysis, decision to publish, or preparation of the manuscript?<br>• **NO** - Include this sentence at the end of your statement: *The funders had no role in study design, data collection and analysis, decision to publish, or preparation of the manuscript.*<br>• **YES** - Specify the role(s) played.<br><br>* typeset | |
| **Competing Interests**<br><br>Use the instructions below to enter a competing interest statement for this submission. On behalf of all authors, disclose any competing interests that could be perceived to bias this work—acknowledging all financial support and any other relevant financial or non-financial competing interests.<br><br>This statement is required for submission and **will appear in the published article** if the submission is accepted. Please make sure it is accurate and that any funding sources listed in your Funding Information later in the submission form are also declared in your Financial Disclosure statement.<br><br>View published research articles from *PLOS ONE* for specific examples. | The authors declare that they have no competing interest. |

| | |
|---|---|
| **NO authors have competing interests**<br><br>Enter: *The authors have declared that no competing interests exist.*<br><br>**Authors with competing interests**<br><br>Enter competing interest details beginning with this statement:<br><br>*I have read the journal's policy and the authors of this manuscript have the following competing interests: [insert competing interests here]*<br><br>* typeset | |
| **Ethics Statement**<br><br>Enter an ethics statement for this submission. This statement is required if the study involved:<br><br>• Human participants<br>• Human specimens or tissue<br>• Vertebrate animals or cephalopods<br>• Vertebrate embryos or tissues<br>• Field research<br><br>Write "N/A" if the submission does not require an ethics statement.<br><br>General guidance is provided below. Consult the submission guidelines for detailed instructions. **Make sure that all information entered here is included in the Methods section of the manuscript.** | Ethical clearance and permissions were obtained from the ethical review committee of Wollo University College of Medicine and Health Sciences with protocol number CMHS 365/2021 and a letter of support was written to Dessie Referral Hospital. Permission was obtained from dessie comprehensive specialized hospitals. Written assent was also obtained from family and surrogates to obtain information those who can't give data. Information obtained from the study participants were kept confidentially. positives results were communicated and given to attending physicians for appropriate treatment |

Powered by Editorial Manager® and ProduXion Manager® from Aries Systems Corporation

| | |
|---|---|
| **Format for specific study types**<br><br>**Human Subject Research (involving human participants and/or tissue)**<br>• Give the name of the institutional review board or ethics committee that approved the study<br>• Include the approval number and/or a statement indicating approval of this research<br>• Indicate the form of consent obtained (written/oral) or the reason that consent was not obtained (e.g. the data were analyzed anonymously)<br><br>**Animal Research (involving vertebrate animals, embryos or tissues)**<br>• Provide the name of the Institutional Animal Care and Use Committee (IACUC) or other relevant ethics board that reviewed the study protocol, and indicate whether they approved this research or granted a formal waiver of ethical approval<br>• Include an approval number if one was obtained<br>• If the study involved *non-human primates*, add *additional details* about animal welfare and steps taken to ameliorate suffering<br>• If anesthesia, euthanasia, or any kind of animal sacrifice is part of the study, include briefly which substances and/or methods were applied<br><br>**Field Research**<br><br>Include the following details if this study involves the collection of plant, animal, or other materials from a natural setting:<br>• Field permit number<br>• Name of the institution or relevant body that granted permission | |
| **Data Availability**<br><br>Authors are required to make all data underlying the findings described fully available, without restriction, and from the time of publication. PLOS allows rare exceptions to address legal and ethical concerns. See the PLOS Data Policy and FAQ for detailed information. | Yes - all data are fully available without restriction |

| | |
|---|---|
| A Data Availability Statement describing where the data can be found is required at submission. Your answers to this question constitute the Data Availability Statement and **will be published in the article**, if accepted.<br><br>**Important:** Stating 'data available on request from the author' is not sufficient. If your data are only available upon request, select 'No' for the first question and explain your exceptional situation in the text box.<br><br>Do the authors confirm that all data underlying the findings described in their manuscript are fully available without restriction? | |
| **Describe where the data may be found in full sentences. If you are copying our sample text, replace any instances of XXX with the appropriate details.**<br><br>• If the data are **held or will be held in a public repository**, include URLs, accession numbers or DOIs. If this information will only be available after acceptance, indicate this by ticking the box below. For example: *All XXX files are available from the XXX database (accession number(s) XXX, XXX.).*<br>• If the data are all contained **within the manuscript and/or Supporting Information files**, enter the following: *All relevant data are within the manuscript and its Supporting Information files.*<br>• If neither of these applies but you are able to provide **details of access elsewhere**, with or without limitations, please do so. For example:<br><br>*Data cannot be shared publicly because of [XXX]. Data are available from the XXX Institutional Data Access / Ethics Committee (contact via XXX) for researchers who meet the criteria for access to confidential data.*<br><br>*The data underlying the results presented in the study are available from (include the name of the third party* | No |

| | |
|---|---|
| *and contact information or URL).* <br>• This text is appropriate if the data are owned by a third party and authors do not have permission to share the data.<br><br>* typeset | |
| Additional data availability information: | Tick here if your circumstances are not covered by the questions above and you need the journal’s help to make your data available.; Tick here if the URLs/accession numbers/DOIs will be available only after acceptance of the manuscript for publication so that we can ensure their inclusion before publication. |

*Powered by Editorial Manager® and ProduXion Manager® from Aries Systems Corporation*

Manuscript

Click here to access/download;Manuscript;updated Manuscript .docx

# Bacterial and fungal profile, multidrug resistance profile of bacterial pathogens and associated risk factors of community-acquired urinary tract infection among HIV positive patients attending Antiretroviral Therapy Clinics of Northeast Ethiopia.

**Mihret Tilahun[1*], Mesfin Fiseha[1], Mihreteab Alebachew[1], Alemu Gedefie[1], Endris Ebrahim[1], Melkam Tesfaye[1], Melaku Ashagrie Belete[1], Abdurahaman Seid [1], Daniel Gebretsadik [1], Wondmagegn Demsiss[1], Bekele Sharew[1], Agumas Shibabaw[1], Habtamu Mekonnen[2] Tewodros Dessie [2]**

[1]Department of Medical Laboratory Sciences, College of Medicine and Health Science, Wollo University, Dessie, Ethiopia

[2] Amhara Public Health Institute Dessie branch

**Authors' information:**

Mihret Tilahun (MSc in Medical Microbiology): tilahunmihret21@gmail.com

Mesfin Fiseha (MSc in hematology and immunohematology): mesfinfiseha40@gmail.com

Alemu Gedefie (MSc in Medical Microbiology): alemugedefie@gmail.com

Mihreteab Alebachew (MSc in medical parasitology and vector control): mihreteab71@gmail.com

Endris Ebrahim (MSc in immunology): endris.index@gmail.com

Melkam Tesfaye (PhD fellow in infectious diseases: Melkam66@gmail.com

Melaku Ashagrie Belete (MSc in Medical Microbiology): melakuashagrie@gmail.com

Abdurahaman Seid (MSc., Asst Prof in Medical Microbiology): gechdk2000@yahoo.com

Daniel Gebretsadik (MSc., Asst Prof in Tropical and infectious diseases): gebretsadikd@gmail.com

Wondmagegn Demsiss (MSc., Asst Prof in Medical Microbiology): *wonde1980@gmail.com*

Bekele Sharew PhD, Asst Professor in Medical Microbiology): bsharew@gmail.com

Agumas Shibabaw (PhD, Asst Professor in Medical Microbiology): agumas2000@gmail.com

Habtamu Mekonnen (MSc in Medical Microbiology: habtamumeko36@gmial.com

Tewodros Dessie (MSc in Medical Microbiology: tedydess21@gmial.com

**Corresponding author**

Mihret Tilahun (MSc.)

Department of Medical Laboratory Science

College of Medicine and Health Sciences,
Wollo University, Dessie, Ethiopia
Telephone: +251- 920988307
Fax: +251 333115250
PO.BOX: 1145
Email: tilahunmihret21@gmail.com

## Abstract

**Background:** Urinary tract infections (UTI) are one of the most common bacterial and fungal infections in humans, occurring both in the community and in health-care settings. UTI is a significant health issue among HIV-infected patients. Antimicrobial resistance is now widespread and poses a serious clinical risk.

**Objective:** This study investigated bacterial and fungal profile, multidrug resistance profile of bacterial pathogens and associated risk Factors of Community acquired urinary tract infection among HIV positive patients attending Antiretroviral Therapy Clinics of Northeast Ethiopia.

**Methods:** Institutional based cross-sectional study was conducted in Dessie Referral Hospital, from February 1, 2021 to March 30, 2021. Socio-demographical and clinical data were collected using structured questionnaires from community acquired urinary tract infection suspected HIV-Positive patients. About 10 ml Clean-catch midstream were collected and inoculated into Blood agar, MacConkey and Cysteine lactose electrolyte deficient. Biochemical tests were performed to identify isolates and antimicrobial susceptibility pattern was performed on disc diffusion techniques. Data were entered and analyzed using SPSS version-25. Descriptive statistics such as tables and bar graphs were used to summarize the findings. Logistic regression analysis was performed and the adjusted odds ratio (AOR) with their 95% confidence interval (CI) was used as statistically significant associations.

**Results:** Among the total 346 study participants, 180 (52.04%) were females. The overall 26.6% (92/346) of which 21.67% (75/346) were bacterial pathogens, while 4.9% (17/346) were fungal pathogens. From a total of 75 bacteria isolates 51(68%) were Gram-negative bacteria. The most commonly isolated Gram-negative bacteria were *E. coli* (21.33%) followed *K. pneumoniae* (14.67%). In addition, From the 17 yeasts isolate of fungi 47.1% (8/17) were represented *by C. tropicalis.* The overall prevalence of MDR bacteria was 61(81.3%). About 13 (81.3%) of E. coli, 9(81.8%) of K. pneumoniae, 8(80%) of Enterococcus species, 7 (77.8%) of P. aeruginosa, and CoNs 7(87.5%) were the most frequently exhibited MDR. age, gender, occupation, educational status, current CD4+ count (cells/mm3), previous hospitalization and diabetes status, history of catheterizations, and urinary tract urgency had a statistically significant association with significant bacteriuria.

**Conclusion**

There is high asymptomatic uropathogens and Multidrugrestistnace pathogens The most dominant bacterial isolates were *E. coli* followed by *K. pneumoniae* and *S. aureus*. Amikacin, and gentamicin were effective against gram negative uropathogens. Management of UTI among symptomatic HIV patients should be supported by laboratory result of urine culture. Regular monitoring of antimicrobial resistance patterns should be assessed by health care providers.

**Keywords:** Uropathogens, community-acquired UTI, Multidrug resistance, Ethiopia

## Introduction

Urinary tract infections (UTI) are caused by gram-negative and gram-positive bacteria, as well as certain fungi. Uro-pathogenic *Escherichia coli* is the most common cause of both uncomplicated and complicated UTIs. *Escherichia coli* is the most common agent involved in uncomplicated UTIs, followed by *Klebsiella pneumoniae*, *Staphylococcus saprophyticus*, *Enterococcus faecalis*, *group B streptococcus, Proteus mirabilis*, *Pseudomonas aeruginosa*, *Staphylococcus aureus*, and Candida spp (1). Following UPEC as the most common causative agent for complicated UTIs, the order of prevalence for causative agents is Enterococcus spp., K. pneumoniae, Candida spp., S. aureus, P. mirabilis, P. aeruginosa, and GBS(2).

The emergence of antibiotic resistance in the treatment of urinary tract infections is a major public health issue worldwide, particularly in developing countries. Antimicrobial treatment of urinary tract infections in HIV patients is required (3). Newly, the most common pathogens of UTIs have developed resistance to most chemotherapeutic agents; their antimicrobial susceptibilities are highly predictable (4). Furthermore, the three most commonly isolated bacteria (Escherichia coli, Klebsiella spp, and Proteus spp) had resistance rates to amoxicillin and tetracycline of 80.1%-90.0% and sensitivity rates of 0 to 25% to Nitrofurantoin, ciprofloxacin, and gentamicin of 0 to 25%. Furthermore, the MDR profile of the isolates revealed that 152 (47.85%) of the isolates were resistant to two or more antimicrobials(5). Resistance to these antimicrobial agents in UTI cases will thus have an impact on future treatment and management of the infection with these drugs. Adequate treatment and control of these conditions necessitate a thorough understanding of the causative bacterial species as well as their susceptibility and resistance to antimicrobial agents. Since the majority of treatments are initiated empirically, knowledge of the organisms, their epidemiological characteristics, and antibacterial susceptibility is required (6).

Every year, approximately 150 million people around the world are diagnosed with UTI, costing the global economy more than a billion dollars. UTIs are the most common bacterial infections and account for a large portion of the workload in clinical microbiology laboratories. It is also the most common infection in HIV patients in many health care settings (7).

UTIs, like respiratory infections, cause increased mortality, disability, hospitalization, and economic loss in the HIV population. Furthermore, it causes renal failure and hypertension. Furthermore, it is prone to nephritic complications such as urinary tract obstruction, which

causes renal colic, pelvic-ureteric junction obstruction, benign prostate hyperplasia, urethral strictures, and bladder dysfunction, which causes urinary stasis and, ultimately, infection (8).

Female gender, a history of recurrent UTI, a lower socioeconomic status, diabetes mellitus, anatomical or physiological genitourinary tract abnormalities, and a low CD4 count have all been linked to the occurrence of bacterial UTI. Because of the aforementioned distribution and determinants, UTI has the potential for recurrence and necessitates a longer treatment (9)

Despite numerous studies on bacterial UTI, antimicrobial susceptibility testing, and HIV infection-related factors in developed countries, there is little data on the burden of bacterial UTI and antimicrobial susceptibility pattern among UTI symptomatic HIV-infected patients in resource-limited settings, including Ethiopia in general, and no study was done in the study area in particular. As a result, the purpose of this study was to determine bacterial and fungal profile, multidrug resistance profile of bacterial pathogens and associated risk Factors of Community acquired urinary tract infection among HIV positive patients attending Antiretroviral Therapy Clinics of Northeast Ethiopia.

## Methods and materials

### Study design, area and period

From February 1, 2021 to March 30, 2021, a health facility-based cross-sectional study was conducted at Dessie Referral Hospital in Dessie town. Dessie is a town founded in total area of 15.08km2 (5.82 sq. m) and located in North Central Ethiopia, east of the Amhara Region. It has a latitude and longitude of 1108'N 39038'E with an elevation of between 2470 and 2,550 meters above sea level. Dessie is 401 km from Addis Ababa to the north and the distance from Dessie to Bahir-dar is 490 km. Dessie town has one public health institute laboratory providing different laboratory services to different areas of the region for both governmental and non-governmental health organizations. According to the Dessie town health administrative office, in the town, there are also 16 governmental health institutions (1 comprehensive specialized hospital, 1 general hospital, 8 health centers) and different private health institutions (3 general hospitals, 6 higher clinics). According to the Dessie town health administrative office, the hospital serves about 12 million people living in the catchment areas. The comprehensive specialized hospital serves about seven million people living in the catchment areas and has a 600 average daily patient flow also has 800 staff, among whom about 600 are health professionals. It has 600 beds, 8 wards, and different OPDs like adult OPD, pediatric OPD, emergency OPD, and TB & HIV

OPD. Also, the hospital provides different services like laboratory, pharmacy, imaging (X-ray, ultra-sound, city scan) and a comprehensive ART service for about 9,500 people.

**Population**

All Suspected community acquired UTI symptomatic HIV seropositive positive individuals living in Dessie town and nearby catchments were source of populations. All UTI symptomatic HIV-seropositive patients and visited ART clinic of Dessie Referral Hospital during the study period before 18 hours of admissions were target populations.

**Eligibility criteria of study participants**

The HIV positive adults who were ≥18 years old and complaining of UTI symptoms were included in the study before 18 hours of admission. However, patients who were on antibiotics for more than two weeks before specimen collection were excluded from the study, and admitted patients for more than 18 hours were excluded from the study.

**Variables**

Dependent variables included the prevalence of bacterial and fungal community acquired UTI and the antimicrobial susceptibility pattern of uro-pathogenic bacteria. While age, sex, marital status, educational level, residence, history of catheterization, ART status, symptoms of UTI (dysuria, urgency, frequency, flank pain and urine color), diabetes, CD4+cell count, Cotrimoxazole usage, and history of hospitalization were independent variables.

**Sample size and sampling technique**

The required sample size was determined by using single population proportion formula considering the following assumptions: Prevalence = 28.6% (prevalence of culture proven UTI among HIV seropositive and UTI symptomatic patients from previous study conducted in Ethiopia (10). Sample size,

$$n = \frac{z^2 * P\,(1 - P)}{d^2}$$

Where: n= the required sample size Z = Z score for 95 % confidence interval = 1.96, p = prevalence=28.6% (0.286), d = tolerable error =5 %, 1-p=Q then, 0.714, n = $((1.96)^2$ x 0.286x (1-0.286))/ $(0.05)^2$=**314. With 10% contingency 32.** However, during the study period, a total of 346 UTI symptomatic HIV-seropositive patients and fulfilling the inclusion criteria were included consecutively.

## Operational definitions

**UTI:** Urinary tract infections are one of the most common infections in humans that can happen anywhere along the urinary tract (11).

**Community acquired urinary tract infection** (CA-UTI); defined as an infection of the urinary tract that occurs in the community or within less than 48 hours of hospital admission and was not incubating at the time of hospital admission (12).

**History of catheterization:** the use of or insertion of a catheter into the bladder or participants who were catheterized prior to data collections(13).

**History of hospitalization:** participants who were taking to health facility and keeping there to 48hrs for treatment p[illegible]or to data collections.

**Multidrug resistance:** A bacterium that is simultaneously resistant for three or more antimicrobial categories (14).

**Data and specimen collection**

A pretested structured questionnaire was used to collect data on sociodemographic characteristics and associated factors. The participants' recent CD4+ count was retrieved from their medical record. About 10 mL of voided clean-catch midstream fresh urine was collected from each participant by using a leak-proof and sterile wide-mouthed screw-capped container. These specimens were labeled and stored in a cold box (4°C) (15) and before being transported to the Wollo University College of Medicine and Health Sciences microbiology laboratory for analysis.

**Isolation and identification of bacteria**

Bacterial isolation and phenotypic characterizations were performed using the recommended culture and biochemical tests (16). A calibrated loop that delivers 0.001 mL of urine was used to inoculate each urine sample on to the Cysteine lactose electrolyte deficient agar (Oxoid Ltd, UK) . The plates were incubated aerobically at 37°C. After overnight incubation, plates with the growth of $\geq 10^4$-$10^5$CFU/mL (colony-forming units per milliliter) accompanied by signs and symptoms were considered significant. Gram stain was performed and sub cultured on to MacConkey agar (HiMediaTM) and 5% blood agar plates (HiMediaTM). However, plates that did not show growth after 24 hrs of incubation were incubated again for the next 24 hrs and discarded as negative if the colony count was not significant (17). From plates with significant

bacteria growth, the phenotypic characterization of bacteria was carried out using colony characteristics, gram reactions, and a series of biochemical reactions, including catalase, coagulase, oxidase, urease, indole, citrate utilization, lysine decarboxylase, glucose, lactose fermentation, gas and H2S production, and motility tests (18) .

**Yeast Identification**

Yeasts were identified using routine diagnostic methods such as Gram stain, Germ tube test, carbohydrate fermentation, and assimilation tests, as well as chromogenic medium (CHROMagar Candida medium, Biomerieux, France) according to the manufacturer's instructions (19).

**Antimicrobial susceptibility testing (AST)**

Antimicrobial susceptibility test was performed using a Kirby–Bauer disk diffusion method based on the Clinical and Laboratory Standards Institute (CLSI) recommendation (20). Three to five pure colonies of the similar appearance of each isolate were emulsified in 4 ml of sterile normal saline. The inoculum suspension was standardized by comparing the turbidity with 0.5 McFarland standards. A Muller-Hinton agar plate (HiMediaTM) was streaked with the prepared inoculum suspension using a sterile swab, and antibiotic discs were placed on each agar plate after 5 minutes using sterile forceps. The antimicrobials (Oxoid Ltd) that were used for bacterial susceptibility testing were, amoxicillin-clavulanic acid (AMC) 10µg, ampicillin (AMP) 10µg, amikacin (Amk) 30µg, cefotaxime (CTX) 30µg, ceftriaxone (CRO) 30µg, trimethoprim-sulphamethaxazole (SXT) 25µg, ciprofloxacin (CIP) 5µg, gentamycin (Gen) 10 µg, ceftazidime (CAZ) 30µg, nitrofurantoin (F) 300µg, tetracycline (TE) 30µg and penicillin (Pen) 10µg (20). Within 15 minutes after application of the discs, the plates were incubated at 35$^{o}$c for 18 hours and the plates were examined for growth and the diameters of zone of inhibition (in mm) was measure by using a graduated caliper in millimeters and results interpreted as sensitive (S), intermediate (I), or resistance (R) (20).

**Data quality management**

The questionnaire was initially prepared in English, translated into the local language (Amharic) by language experts back to English by other experts to assure its consistency and accuracy. The questionnaire was pretested on 5% HIV-positive patients at Dessie health center, Dessie, Ethiopia. The quality of culture media was tested for sterility and performance. Sterility of culture media was checked by incubating overnight at 37 ºC without specimen inoculation. Any physical change like cracks, excess moisture, color, hemolysis, dehydration, contamination was

assessed & the expiration date was also checked. The new batch culture medium and antimicrobial disks were checked for performance and quality using the American Type Culture Collection (ATCC) reference strains such as *E. coli* (ATCC® 25922), *S. aureus* (ATCC® 25923), *Klebsiella pneumonia* (ATCC 700603) and *P. aeruginosa* (ATCC® 27853).

**Statistical analysis**

The data generated was entered every day into epi-data version 4.6.0.4. The data was then exported and analyzed using the Social Sciences Statistical Package (SPSS) version 25. The frequency and percentage descriptive statistics were calculated and presented using graphs and tables. A bivariable analysis was conducted in order to classify factors that separately affect the frequency of dependent variables. Variables with a P-value less than or equal to 0.25 in bivariable analysis were subjected to multi-variable analysis. adjusted odd ratio with a p value of <0.05 and 95% CI taken as statistically significant. Finally, the results were presented in words, graphs and tables.

**Ethical considerations**

Ethical clearance and permissions were obtained from the ethical review committee of Wollo University College of Medicine and Health Sciences with protocol number CMHS 365/2021 and a letter of support was written to Dessie Referral Hospital. Permission was obtained from dessie comprehensive specialized hospitals. Written assent was also obtained from family and surrogates to obtain information those who can't give data. Information obtained from the study participants were kept confidentially. positives results were communicated and given to attending physicians for appropriate treatment.

## Results

**Socio-demographic characteristics**

A total of 346 HIV positive patients were included in this study. The age of the study participants ranged from 21 to 80 years, with a mean (±SD) age of 39.44 (±10.87) years, and 116 (33.5%) of them were in the age range of 35–44 years. About 180 (52.02%) of the respondents were females. Regarding educational status, 115 (33.2%) of the respondents had a primary level education. Concerning occupational status, 90 (26.01%) were self-employed. Concerning residences, 215 (62.1%) of them were urban dwellers, as shown in **(Table 1).**

**Table 1. Sociodemographic characteristics of UTI symptomatic HIV positive patients attending at Dessie referral hospital, Dessie, Ethiopia (February 1, 2021- march 30, 2021).**

| Variable | Category | Culture Result | | Frequency | Percentage |
|---|---|---|---|---|---|
| | | Positive No (%) | Negative No (%) | | |
| Sex | Male | 42(25.3) | 124(74.7) | 166 | (47.98) |
| | Female | 50(27.8) | 130(72.2) | 180 | (52.02) |
| Residence | Rural | 62(28.8) | 153(71.2) | 215 | 62.1 |
| | Urban | 30(22.9) | 101(77.1) | 131 | 37.9 |
| Age (in years) | 18–24 | 10(18.9) | 43(81.1) | 53 | 15.3 |
| | 25–34 | 21(24.7) | 64(75.3) | 85 | 24.6) |
| | 35–44 | 30(25.9) | 86(74.1) | 116 | 33.5 |
| | >44 | 31(33.7) | 61(66.3) | 92 | 26.6 |
| Educational status | Illiterate | 42(36.5) | 73(63.5) | 115 | 33.2 |
| | Primary | 20(23.3) | 66(76.7) | 86 | 24.9 |
| | Secondary | 22(24.2) | 69(75.8) | 91 | 26.3 |
| | College/University | 8(14.8) | 46(85.2) | 54 | 15.6 |
| Marital status | Married | 45(37.2) | 76(62.8) | 121 | 35 |
| | Unmarried | 18(22.5) | 62(77.5) | 80 | 23.1 |
| | Divorced | 13(13.7) | 82(86.3) | 95 | 27.5 |
| | Widowed | 16(32) | 34(68) | 50 | 14.5 |
| Occupation | House Wife | 15(25.9) | 43(74.1) | 58 | 16.8 |
| | Civil Servant | 10(22.2) | 35(77.8) | 45 | 13 |
| | Self-Employee | 9(18) | 41(82) | 50 | 14.5 |
| | Driver | 10(20.8) | 38(79.2) | 48 | 13.9 |
| | Daily Labor | 23(25.6) | 67(74.4) | 90 | 26.01 |
| | Farmer | 25(45.5) | 30(55.5) | 55 | 15.9 |

**Clinical characteristics of study participants**

The clinical picture of study participants revealed that 150 (43.4%) and 140(40.5%) had a history of previous hospitalization and catheterization, of which 35(30.4%) and 42(30%) were culture positive for community acquired respectively. About 121(35%) of the respondents had diabetes at the time of the survey, with positive growth of 32(25.6%). Among the respondents, 105 (30.3%) were currently on their CD4 count was 201-350 cells/mm3 with 35(33.3%). Moreover, 210(60.7%), 200 (57.8%), 165 (47.7%), and 164 (47.4%) of participants showed dysuria, frequency of urination, flank pain, and abnormal urine color with positive growth of 58(27.6%), 55(27.5%), 61 (37%), and 64 (39%), respectively (**Table 2)**.

**Table 2. Frequency of clinical characteristics of UTI symptomatic HIV positive patients attending Dessie Referral Hospital, Dessie, Ethiopia (February 1, 2021- march 30, 2021).**

| Variables | Category | Culture Result | | Frequency | Percentage |
|---|---|---|---|---|---|
| | | Positive No (%) | Negative No (%) | | |
| History of hospitalization | Yes | 35(30.4) | 115(69.6) | 150 | 43.4 |
| | No | 57(29.1) | 139(70.9) | 196 | 56.6 |
| Use of cotrimoxazole as prophylaxis | Yes | 40(26) | 114(74) | 154 | 44.5 |
| | No | 54(29.1) | 138(70.9) | 192 | 55.5 |
| Diabetes | Yes | 32(25.6) | 89(74.4) | 121 | 35 |
| | No | 60(24.6) | 184(75.4) | 244 | 65 |
| History of catheterization | Yes | 42(30) | 120(70) | 140 | 40.5 |
| | No | 50(24.3) | 136(75.7) | 206 | 59.5 |
| UTI symptoms | | | | | |
| Dysuria | Yes | 58(27.6) | 152(72.4) | 210 | 60.7 |
| | No | 34(25) | 102(75) | 136 | 39.3 |
| Frequency of urination | Yes | 55(27.5) | 155(72.5) | 200 | 57.8 |
| | No | 37(25.3) | 109(74.7) | 146 | 42.2 |
| Urine color | abnormal | 64(39) | 100(61) | 164 | 47.4 |
| | Normal | 28(15.4) | 154(84.6) | 182 | 52.6 |
| flank pain | Yes | 61(37) | 104(63) | 165 | 47.7 |
| | No | 31(17.1) | 157(82.9) | 181 | 52.8 |
| Current CD4+ count | ≤200 cells/mm$^3$ | 24(32.9) | 49(67.1) | 73 | 21.1 |
| | 201-350 cells/mm3 | 35(33.3) | 70(66.7) | 105 | 30.3 |
| | 351-500 cells/mm3 | 20(23.8) | 64(762) | 84 | 24.3 |
| | ≥500 cells/mm3 | 13(15.3) | 72(84.7) | 85 | 24.6 |

**Prevalence of community acquired UTI and isolated pathogens**

The prevalence of community-acquired UTIs Out of two hundred twenty-seven (346) urine samples collected, significant bacterial and yeast growth were observed in 92 samples, giving an overall prevalence of 26.6% (92/346) of which 21.67% (75/346) were bacterial pathogens, while 4.9% (17/346) were fungal pathogens. Among 75 bacterial isolates, 21.33% (16/75) were in *E. coli*, the most frequent bacterium, followed by *K. pneumoniae*, *enterococcus species*, and *P. aeruginosa*, accounting for 14.67% (11/75), 13.33% (10/75), and 12% (9/75), respectively. The least number of bacterial isolates were *Enterobacter cloacae*, which accounted for 4 (5.33%). Gram-positive bacteria accounted for only 32% (24/75) of the bacterial isolates, of which

41.67% (10/24) of the isolates were *Enterococcus species*. Of 17 yeast isolates, 47.1% (8/17) were represented by *C. tropicalis* **(Fig.1)**

**Antimicrobial susceptibility pattern of bacterial Uropathogens**

Antimicrobial susceptibility patterns of the isolated gram negative and gram positive uropathogens were shown in Tables 3 and 4, respectively. 52.9%, 52.9%, and 58.8% of the total isolated gram negative uropathogens were susceptible to ceftriaxone, ciprofloxacin, and gentamicin, respectively, whereas 51%, 72.5%, 72.5%, 84.3%, 60.8% and 60.8% of the isolates were resistant to amikacin, ampicillin, amoxicillin-Clavulanic acid, trimethoprim-sulphsmethsoxazole, nitrofurantoin, and ceftazidime, Discretely, it was observed that 62.5% of Escherichia coli strains were sensitive to gentamycin. But, 87.5%, 87.5%, 81.3%, 75%, 62.5%. and 56 % of bacterial isolates were resistant to tetracycline, Trimethoprim-sulphsmethsoxazole, amoxicillin-Clavulanic acid, ampicillin, nitrofurantoin, and ceftazidime, respectively. Similarly, 80% of ampicillin, 90.9% of trimethoprim-sulphsmethsoxazole, 90.9% of tetracycline and 63.6% of nitrofurantoin, and 63.6% ceftazidime, 54.5% was resistance to K. *pneumoniae*. However, (54.5%) of gentamicin, 54.5% of both amikacin and ciprofloxacillin, were sensitive against to *K. pneumoniae* **(Table 3).**

**Table 3. Antimicrobial susceptibility pattern of Gram-negative bacterial isolates to antimicrobial agents among Community acquired UTI HIV-positive patients at Dessie Referral Hospital, Dessie, Ethiopia (February 1, 2021- march 30, 2021).**

| Etiologic agents | Pattern | Antimicrobial agents N (%) | | | | | | | | | | |
|---|---|---|---|---|---|---|---|---|---|---|---|---|
| | | AMP | AMC | AK | CRO | CIP | GN | CAZ | TE | CTX | SXT | F |
| *E. coli (16)* | S | 4(25) | 3(18.7) | 9(56.2 | 9(56.2 | 8(50) | 10(62.5) | 7(43.8) | 2(12.5) | 8(50) | 2(12.5) | 6(37.5) |
| | R | 12(75) | 13(81.3) | 7(43.8) | 7(43.8) | 8(50) | 6(37.5) | 9(56.2) | 14(87.5) | 8(50) | 14(87.5) | 10(62.5 |
| *K. pneumoniae (11)* | S | 3(27.3) | 4(36.4) | 5(45.5) | 6(54.5) | 5(45.5) | 6(54.5) | 4(36.4) | 1(9.1) | 6(54.5) | 1(9.1) | 4(36.4) |
| | R | 8(72.7 | 7(63.6) | 6(54.5) | 5(45.5) | 6(54.5) | 5(45.5) | 7(63.6) | 10(90.9) | 5(45.5) | 10(90.9) | 7(63.6) |
| *P. mirabilis (5)* | S | 1(20) | 2(40) | 3(60) | 3(60) | 3(60) | 1(20) | 2(40) | 1(20) | 3(60) | 1(20) | 2(40) |
| | R | 4(80) | 3(60) | 2(40) | 2(40) | 2(40) | 4(80) | 3(60) | 4(80) | 2(40) | 4(80) | 3(60) |
| *P. aeruginosa (9)* | S | 3(33.3) | 2(22.2) | 5(55.6) | 5(55.6) | 5(55.6) | 6(66.7) | 4(44.4) | 1(11.1) | 4(44.4) | 2(22.2) | 3(33.3) |
| | R | 6(66.7) | 7(87.8) | 4(44.4) | 4(44.4) | 4(44.4) | 3(33.3) | 5(55.6) | 8(88.9) | 5(55.6) | 7(87.8) | 6(66.7) |
| *Enterobacte* | S | 1(25) | 1(25) | 2(50) | 2(50) | 2(50) | 3(75) | 1(25) | 1(25) | 2(50) | 1(25) | 2(50) |

| | | | | | | | | | | | | |
|---|---|---|---|---|---|---|---|---|---|---|---|---|
| *r cloacae. (4)* | R | 3(75) | 3(75) | 2(50) | 2(50) | 2(50) | 1(25) | 3(75) | 3(75) | 2(50) | 3(75) | 2(500 |
| *Providencia spp (6)* | S | 2(33.3) | 2(33.3) | 3(50) | 2(33.3) | 4(66.7) | 4(66.7) | 2(33.3) | 1(16.7) | 3(50) | 1(16.7) | 3(50) |
| | R | 4(66.7) | 4(66.7) | 3(50) | 4(66.7) | 2(33.3) | 2(33.30 | 4(66.7) | 5(83.3) | 3(50) | 5(83.3) | 3(50) |
| Total (51) | S | 14(27.5) | 14(27.5) | 25(49) | 27(52.9) | 27(52.9) | 30(58.8) | 20(39.2) | 7(13.7) | 26(51) | 8(15.7) | 20(39.2) |
| | R | 37(72.5) | 37(72.5) | 26(51) | 24(47.1) | 24(47.1) | 21(41.2) | 31(60.8 | 44(86.3 | 25(49) | 43(84.3) | 31(60.80 |

S: sensitive; R: resistant; AMP: ampicillin; AMC: Amoxicillin-Clavulanic acid; CTX: cefotaxime; CAZ: Ceftazidime; F: Nitrofurantoin; CRO: ceftriaxone; CIP: ciprofloxacin; GN: gentamicin; AK: Amikacin; TE: tetracycline; SXT: Trimethoprim-sulphsmethsoxazole.

From the total isolated Gram-positive uropathogens, 66.7% of isolates were susceptible to clindamycin. Whereas, 87.5%, 83.3%, 75%, 58.3%, and 58.3% of Gram-positive isolates were resistant to tetracycline, trimethoprim-sulfamethoxazole, penicillin, chloramphenicol, and Nitrofurantoin, respectively. About 70% of clindamycin and 60% of ciprofloxacin were more effective against *Enterococcus species,* respectively. However, *Enterococcus species* showed more resistance to 90% for tetracycline, 80% for trimethoprim-sulphsmethsoxazole and 70% for nitrofurantoin. Likewise, *coagulase negative staphylococci* (CoNs) were resistant to most of the antimicrobials 87.5% for tetracycline, 87.5% for trimethoprim-sulphsmethsoxazole, and 75% for penicillin and chloramphenicol each **(Table 4).**

**Table 4. Antimicrobial susceptibility pattern of Gram-positive bacterial isolates to antimicrobial agents among community acquired** UTI **HIV-positive patients attending at Dessie Referral Hospital, Dessie, Ethiopia (February 1, 2021- March 30, 2021).**

| Bacterial isolates | Number of Resistance Pathogens to Antimicrobial Agents No (%) | | | | | | | | | | |
|---|---|---|---|---|---|---|---|---|---|---|---|
| | | E<br>N (%) | P<br>N (%) | C<br>N (%) | CXT<br>N (%) | F | CL<br>N (%) | CIP<br>N (%) | SXT<br>N (%) | FOX<br>N (%) | TE<br>N (%) |
| S. *aureus* (6) | S | 4(66.7) | 1(16.7) | 3(50) | 3(50) | 2(33.3) | 4(66.7) | 4(66.7) | 1(16.7) | 3(50) | 1(16.7) |
| | R | 2(33.3) | 5(83.3) | 3(50) | 3(50) | 4(66.7) | 2(33.3) | 2(33.3) | 5(83.3) | 3(50) | 5(83.3) |
| CONS (8) | S | 3(37.5) | 2(25) | 2(25) | 3(37.5) | 5(62.5) | 5(62.5) | 2(25) | 1(12.5) | NT | 1(12.5) |
| | R | 5(62.5) | 6(75) | 6(75) | 5(62.5) | 3(37.5) | 3(37.5) | 6(75) | 7(87.5) | NT | 7(87.5) |
| *Enterococcus species (10)* | S | 4(40) | 3(30) | 5(50) | 6(60) | 3(30) | 7(70) | 6(60) | 2(20) | 4(40) | 1(10) |
| | R | 6(60) | 7(70) | 5(50) | 4(40) | 7(70) | 3(30) | 4(40) | 8(80) | 6(60) | 9(90) |
| Total (24) | S | 11(45.83) | 6(25) | 10(41.7) | 12(50) | 10(41.7) | 16(66.7) | 12(50) | 4(16.7) | 7(43.75) | 3(12.5) |
| | R | 13(54.17) | 18(75) | 14(58.3) | 12(50) | 14(58.3) | 8(33.3) | 12(50) | 20(83.3) | 9(56.25) | 21(87.5) |

**Key: CL** = Clindamycin, **E** = Erythromycin, **C** = Chloramphenicol, **CIP** = Ciprofloxacin, **TE** = Tetracycline, **SXT** = Trimethoprim-Sulfamethoxazole, **CTX**= cefotaxime, **FOX**=Cefoxitin, **F:** nitrofurantoin**, P** =penicillin, **R** = Resistant, **OX=**oxacillin**, FOX**=Cefoxitin **S** = Sensitive, **NT** = Not tested

### Multi-drug resistance patterns of the bacterial isolates

Overall, 70 (93.3%) bacterial isolates were resistant to at least one antimicrobial agent, and 63 (84%) isolates were resistant to ≥2 antimicrobials. About 11 (14.7%) isolates had resistance to five or more antimicrobials. The overall prevalence of MDR bacteria was 61(81.3%). About *13* (81.3%) of *E. coli*, 9(81.8%) of *K. pneumoniae*, *8*(80%) of *Enterococcus species, 7 (77.8%) of P. aeruginosa*, and *CoNs* 7(87.5%) were the most frequently exhibited MDR **(Table 5).**

**Table 5. Multi-drug resistance patterns of uropathogens bacterial isolates among UTI symptomatic HIV-positive patients attending at Dessie Referral Hospital, Dessie, Ethiopia (February 1, 2021- March 30, 2021).**

| Isolated organisms | Total | MDR to antimicrobials | | | | | | |
|---|---|---|---|---|---|---|---|---|
| | | R0 N (%) | R1 N (%) | R2 N (%) | R3 N (%) | R4 N (%) | ≥R5 N (%) | MDR N (%) |
| *E. coli* | 16 | 1(6.3) | 1(6.3) | 1(6.3) | 4(25) | 7(43.7) | 2(12.5) | 13(81.3) |
| *K. pneumoniae* | 11 | 1(9.1) | 1(9.1) | - | 4(36.4) | 2(18.2) | 3(27.3) | 9(81.8) |
| *S. aureus* | 6 | 1(16.7) | - | - | 3(50) | 2(33.3) | - | 5(83.3) |
| *P. mirabilis* | 5 | 0 | 1(20) | - | 2(40) | 1(20) | 1(20) | 4(80) |
| *CoNs* | 8 | 1(12.5) | - | - | 4(50) | 2(25) | 1(12.5) | 7(87.5) |
| *P. aeruginosa* | 9 | 0 | 1(11.1) | 1(11.1) | 3(33.3) | 2(22.2) | 2(22.2) | 7(77.8) |
| *Enterobacter Cloacae* | 4 | 0 | 1(25) | - | 1(25) | 2(50) | - | 3(75) |
| *Enterococcus species* | 10 | 1(10) | 1(10) | - | 4(40) | 3(30) | 1(10) | 8(80) |
| *Providencia species* | 6 | 0 | 1(16.7) | - | 2(33.3) | 2(33.3) | 1(16.7) | 5(83.3) |
| Total | 75 | 5(6.7) | 7(9.3) | 2(2.7) | 27(36) | 23(30.7) | 11(14.7) | 61(81.3) |

R0=No antibiotic resistance category, R1=Resistance to one antibiotic category, R2=Resistance to two antibiotics category, R3=Resistance to three antibiotics category, R4=Resistance to four antibiotics category, R5=Resistance to five antibiotics category, R6=Resistance to six antibiotics category, R7=Resistance to seven antibiotics category

**Factors associated with significant bacteriuria**

In the current study, bivariate analysis was performed on sociodemographic and facility characteristics such as sex, family size, residence, occupations, immunization status, education status, and current marital status, current CD4 count, history of hospitalizations, catheterizations, and status of diabetics; and signs and symptoms of urinary tract infections. Multivariate analysis was done by including variables that had a p-value of $\leq 0.25$ in bivariate analysis. After adjustment for age, sex, occupations, educational status, current CD4+ count (cells/mm3), history of previous hospitalization and diabetes status, history of catheterizations, urgency of

urinary tract. Age >44year [AOR=1.56, 95%CI: 1.11-9.35, P=0.001], being female [AOR=3.29, 95%CI: 1.26-13.41, P=0.002], being daily labor [AOR=4.54, 95%CI: 1.41-21.71, P=0.003], being farmer [AOR=6.54, 95%CI: 1.21-23.51, P=0.004], participants unable to read and write [AOR=4.58, 95%CI: 1.21-13.58, P=0.001], HIV patients with CD4 count of ≤ 200 cells/mm$^3$ (AOR: 7.44; 95%CI: 1.64-27.21, P=0.001) and CD4 count of 201-350 cells/mm$^3$ (AOR: 4.55; 95%CI: 1.36-18.28, P=0.009), patients who had chronic diabetics (AOR: 4.80; 95%CI: 1.85-16.31), patients who had chronic diabetics (AOR: 4.80; 95%CI: 1.85-16.31, p=0.009), Patients having history of hospitalization (AOR: 6.27; 95%CI: 1.25-15.17, p=0.002) and patients who had urgency of urinations (AOR: 6.51; 95%CI: 1.75-36.31, p=0.0025) were statistically significant associated risk factors for community acquired urinary tract infections (Table 6).

**Table 6. Bivariable and multivariable analysis for variables associated with bacteriuria among UTI symptomatic HIV-positive patients attending at Dessie Referral Hospital, Dessie, Ethiopia (February 1, 2021- march 30, 2021).**

| Characteristics | | Urine Culture Result | | COR (95% CI) | p-value | AOR (95% CI) | P-value |
|---|---|---|---|---|---|---|---|
| | | Positive n (%) | Negative n (%) | | | | |
| Age in years | 18–24 | 10(18.9) | 43(81.1) | 1 | 1 | | |
| | 25–34 | 21(24.7) | 64(75.3) | 0.65[0.25-5.295] | 0.984 | | |
| | 35–44 | 30(25.9) | 86(74.1) | 0.985[0.154-14.038] | 0.838 | | |
| | >44 | 31(33.7) | 61(66.3) | 1.56[1.11-19.035] | 0.012 | 1.56[1.11-9.35] | 0.001 |
| Sex | Male | 42(25.3) | 124(74.7) | 1 | 1 | 1 | 1 |
| | Female | 50(27.8) | 130(72.2) | 1.79[1.1-3.655] | 0.185 | 3.29[1.26- 13.41] | 0.002 |
| Residence | Urban | 62(28.8) | 153(71.2) | 1 | 1 | | |
| | Rural | 30(22.9) | 101(77.1) | 1.4[0.427-3.026] | 0.89 | | |
| Occupation | House Wife | 15(25.9) | 43(74.1) | 1 | 1 | | |
| | Civil Servant | 10(22.2) | 35(77.8) | 0.95[0.41-5.34] | 0.54 | | |
| | Self-Employee | 9(18) | 41(82) | 1.25[0.36-13.54] | 0.36 | | |
| | Driver | 10(20.8) | 38(79.2) | 0.71[0.15-16.12] | 0.65 | | |
| | Daily Labor | 23(25.6) | 67(74.4) | 3.842[1.37-17.91] | 0.17 | 4.54[1.41-21.71] | 0.003 |
| | Farmer | 25(45.5) | 30(55.5) | 4.842[1.17-17.91] | 0.18 | 6.54[1.21-23.51] | 0.004 |
| Educational status | Illiterate | 42(36.5) | 73(63.5) | 3.85[1.76-12.61] | 0.114 | 4.58[1.21-13.85] | 0.001 |
| | Primary | 20(23.3) | 66(76.7) | 0.98 [0.928-12.398] | 0.265 | | |
| | Secondary | 22(24.2) | 69(75.8) | 1.95[0.43-12.22] | 0.655 | | |
| | College/University | 8(14.8) | 46(85.2) | 1 | 1 | 1 | 1 |
| Current marital status | Married | 45(37.2) | 76(62.8) | 1.89[0.61-16.86] | 0.43 | | |
| | Unmarried | 18(22.5) | 62(77.5) | 1.369[0.325-5.766] | 0.668 | | |
| | Divorced | 13(13.7) | 82(86.3) | 1.05[0.153 -7.270] | 0.956 | | |
| | Widowed | 16(32) | 34(68) | 1 | 1 | | |
| Current CD4+ count (cells/mm$^3$) | ≤200 | 24(32.9) | 49(67.1) | 5.29[1.39-18.51] | **0.018** | 7.44[1.64-27.21] | **0.001** |
| | 201-350 | 35(33.3) | 70(66.7) | 4.18[1.55-17.10] | **0.024** | 4.55[1.36-18.28] | **0.009** |

| | | | | | | | |
|---|---|---|---|---|---|---|---|
| | 351-500 | 20(23.8) | 64(762) | 0.98[0.57-12.56] | 0.705 | 0.99[0.89-12.24] | 0.861 |
| | ≥501 | 13(15.3) | 72(84.7) | 1 | 1 | 1 | 1 |
| History of hospitalization | Yes | 35(30.4) | 115(69.6) | 1.98[1.78- 13.28] | 0.181 | 6.27[1.25- 15.17] | 0.002 |
| | No | 57(29.1) | 139(70.9) | 1 | 1 | 1 | 1 |
| Use of cotrimoxazole as prophylaxis | Yes | 40(26) | 114(74) | 1 | 1 | | |
| | No | 54(29.1) | 138(70.9) | 0.953[0.455-1.995] | 0.898 | | |
| Diabetes | Yes | 32((25.6) | 89(74.4) | 3.78[1.22-15.42] | 0.016 | 4.80[1.85- 16.31] | 0.009 |
| | No | 60(24.6) | 184(75.4) | 1 | 1 | 1 | 1 |
| History of catheterization | Yes | 42(30) | 120(70) | 1.794[1.53-16.03] | 0.048 | 4.65[1.55- 26.31] | 0.0012 |
| | No | 50(24.3) | 136(75.7) | 1 | 1 | | |
| Dysuria | Yes | 58(27.6) | 152(72.4) | 0.753[0.368-1.979] | 0.711 | | |
| | No | 34(25.4) | 100(74.6) | 1 | 1 | | |
| Frequency of urination | Yes | 55(27.5) | 155(72.5) | 0.629[0.395-1.741] | 0.621 | | |
| | No | 37(25.3) | 109(74.7) | 1 | 1 | | |
| Urgency | Yes | 64(39) | 100(61) | 1.427[1.89-22.55] | 0.039 | 6.51[1.75- 36.31] | 0.0025 |
| | No | 28(15.4) | 154(84.6) | 1 | 1 | | |
| flank pain | Yes | 61(37) | 104(63) | 1.193[0.548-2.599] | 0.657 | | |
| | No | 31(17.1) | 157(82.9) | 1 | 1 | | |

## Discussion

A urinary tract infection (UTI) is defined as the invasion of the urinary tract by one or more uro-pathogenic bacteria species, which results in significant bacteriuria and the presence of symptoms such as dysuria (21). According to demographic data, it is more common in women. Women are predisposed to UTIs due to anatomical and physical factors such as a shorter urethra. Surgery, pregnancy, advanced age, diabetes mellitus, kidney stones, immune system impairment, neurogenic bladder, and urinary tract abnormalities may all increase the risk of developing an UTI (22). In this study, the prevalence of significant bacteriuria was 26.6% [95% CI: 21.5% - 31.2%]. The current study is comparable with study done in Hawassa (23.1%)(23), Uganda (22.0%)(24). However, this finding was relatively higher than studies done in Jimma (13.7%) (25), Gojjam Ethiopia (20.6%)(26) , Hawassa (4.9%) (27), in Addis Ababa, Ethiopia (18.6%) and 11.3% (28, 29) and Harar, Ethiopia (18%) (30). But, it is lower than studies conducted in Nigeria (32.5%) (31), South Africa (48.7%) (32), Nepal (54.76%) and India (100%) and (49.15%) (33, 34). This variation might be due to differences in sample size, the degree of the

immune status of the study participants, geographical variation, ART status and socioeconomic conditions.

Candidauria is the presence of Candida spp. in the urine, such as C. albicans. Candidauria is classified as asymptomatic (in healthy people or patients) or symptomatic. Patients with cystitis, epididymorchitis, prostatis, pyelonephritis, and renal candidiasis experience symptomatic candidiasis (35). Asymptomatic Candidauria, on the other hand, is usually harmless and is not considered a disease. C. albicans is one of the most common fungi that can cause Candidauria (20% of nosocomial infections) (36). In the current study, the prevalence of community-acquired UTIs Out of two hundred twenty-seven (346) urine samples collected, significant yeast growth was observed in 4.9% (17/346) were fungal pathogens. Of the 17 yeast isolates, 47.1% (8/17) were represented by *C. tropicalis. Study also supported* C. albicans, C. glabrata, and C. tropicalis have been reported as the three most common species among young and adult individuals (37). Multiple studies showed that within hospital settings at least 10–15% of hospital-acquired UTIs are caused by Candida species and Candidauria is especially common in intensive care units. In study conducted in Addis Ababa, Candidauria represented 7% UTIs and 94% were hospital-acquired in which 62.5% of them were observed in ICU patients. C. albicans was the leading cause of Candidauria and 81.1% candida species were isolated from ward-acquired positive urine culture and ICU acquired positive urine culture samples (10) and study done in two microorganisms were Candida spp. (Candida glabrata and Candida krusei) (38).

Among 75 bacterial isolates, 21.33% (16/75) were in *E. coli*, the most frequent bacterium, followed by *K. pneumoniae*, *enterococcus species*, and *P. aeruginosa*, accounting for 14.67% (11/75), 13.33% (10/75), and 12% (9/75), respectively. Similarly finding was showed that *E. coli* was the predominant on those studies in Bahirdar Ethiopia (38.1%)(30), Gondar, Ethiopia (56.1%)(39) , Jimma, Ethiopia (54.3%) (25), and Tertiary Care Hospital, India (41.7%)(29), Saudi Arabia (40) and Ethiopia (27). However, it was inconsistent with the findings reported in Ebony State, Nigeria (41) and India (33), they found that *S. aureus* (45.3%) and *P. aeruginosa* (41.9%) and in Turkey the most common causative agent was *E. coli* (66.6% of cases) followed by *K. pneumoniae* (16.6%) and others such as *Enterobacter spp*. (7.7%) (38)were the commonest urinary tract pathogens. This discrepancy might be due to differences in test facility, strain variety, geographical variation, or study group variations. The presence of a

unique structure that aids bacteria attachment to uroepithelial cells, allowing for multiplication and tissue invasion, could explain the predominance of *E. coli* (42, 43).

Even though not statistically significant association was observed in this study, we found that the bacteriuria was high among females (27.8%) than males (25.3%). This finding is in agreement with various studies conducted in Ethiopia (28, 30, 39), South Africa (32), Nigeria (41). This could be explained by physiological and anatomical differences between males and females, such as males' shorter urethra, a decrease in normal vaginal flora, and females have less acidic vaginal surface pH (28).

In the current study, about 52.9%, 52.9%, and 58.8% of the total isolated gram negative uropathogens were susceptible to ceftriaxone, ciprofloxacin, and gentamicin, respectively, whereas, 51%, 72.5%, 72.3%, 84.3%, 60.8%, and 60.8% of the isolates were resistant to amikacin, ampicillin, amoxicillin-Clavulanic acid, and trimethoprim. The other study on urinary tract bacterial and fungal isolates supported these current findings with Frequency Percentage 11 sulphsmethsoxazole, nitrofurantoin, and ceftazidime and Most of the urinary bacterial isolates were highly sensitive to ceftazidime (95%) and ciprofloxacin (88% (44). In this study 87.5%, 87.5%, 81.3%, 75%, 62.5%. and 56 % of Escherichia coli strains were resistant to tetracycline, Trimethoprim-sulphsmethsoxazole, amoxicillin-Clavulanic acid, ampicillin, nitrofurantoin, and ceftazidime, respectively. Similarly, 80% of ampicillin, 90.9% of trimethoprim-sulphsmethsoxazole, 90.9% of tetracycline and 63.6% of nitrofurantoin, and 63.6% ceftazidime, 54.5% was resistance to K. pneumoniae.

In this study, 87.5%, 87.5%, 81.3%, 75%, 62.5%, and 56 % of Escherichia coli strains were resistant to tetracycline, Trimethoprim-sulphsmethsoxazole, amoxicillin-Clavulanic acid, ampicillin, nitrofurantoin, and ceftazidime, respectively. Similarly, 80% of ampicillin, 90.9% of trimethoprim-sulphsmethsoxazole, 90.9% of tetracycline, 63.6% of nitrofurantoin, and 63.6% of ceftazidime, 54.5% was resistance to K. pneumoniae. There were also similar findings from studies conducted Addis Ababa In K. pneumoniae, the highest level of resistance was observed against sulfamethoxazole trimethoprim (86.4%) cefotaxime (86.4%), cefepime (85.4%), ceftazidime (85.4%), amoxacillin-clavulinic acid (85.4%), gentamicin (70.0%), and ciprofloxacin (50.5% (45), In Iran: sulfamethoxazole-trimethoprim (91.4%), ceftazidime (91.4%), and gentamicin (82.8%) (46) ; in Sierra Leone: ciprofloxacin (73.4%) and gentamicin (60%) (47), in Equatorial Guinea: sulfamethoxazole-trimethoprim (100%), amoxacillin-

clavulinic acid (96.6%), gentamicin (86.2%), and ciprofloxacin (87.5% (48). The high antibiotic resistance of Enterobacteriaceae may be due to antibiotic misuse and overuse, combined with inadequate infection control measures (49) This was comparable with the study done in Gondar, Ethiopia (39) and Harar, Ethiopia (30), *P. aeruginosa* was 100% resistant to ampicillin, cefotaxime, and gentamicin and trimethoprim-sulphsmethsoxazole *P.mirabilis* 100% resistant to ampicillin. This is in agreement with a study done in Ethiopia (28).

Similarly, about 87.5%, 83.3%, 75%, 58.3%, and 58.3% of gram-positive isolates were resistant to tetracycline, trimethoprim-sulfamethoxazole, penicillin, chloramphenicol, and Nitrofurantoin, respectively. The most isolated gram-positive *Enterococcus species* showed more resistance to 90% of tetracycline, 80% of trimethoprim-sulphsmethsoxazole and 70% of nitrofurantoin. Likewise, coagulase negative staphylococci (CoNs) were resistant to most of the antimicrobials; 87.5% for tetracycline, 87.5% for trimethoprim-sulphsmethsoxazole, and 75% for penicillin and chloramphenicol each. This is in line with a study done in Harar, Ethiopia, where 57.1% of *S. aureus* were resistant to gentamicin, tetracycline, and cefoxitin (57.1%) (30). We also found that 85% of *Coagulase-negative staphylococcus spp*. were resistant to Trimethoprim-sulphamethaxazole (85%) and over 60% were resistant to penicillin, gentamicin, and tetracycline.

Multidrug resistance has serious implications on the health outcome of HIV-infected patients (50). It is quite alarming to note that almost was 61(81.3%) of currently isolated bacteria were found to be resistant to three or more antimicrobials. This was higher compared to previous findings reported in Harar, Ethiopia (46%) (30), Dessie (74.6%) (51), Gondar (68%) (52) and Nepal (64.04%)(53) and India (28%) (54) . But it was lower than a report from Gondar, Ethiopia (95%) and 87.4%) (3, 39), Addis Ababa (100%) (28), in Bahirdar (93.1%) (55), in Nepal (96.84%) (53). These differences might be due to the irrational drug utilization habit of the communities or to the over the distribution of those sensitive and resistant strains of bacteria.

There was an intra-species difference in MDR level. The present study showed that the level of MDR in E. coli 13 (81.3%), K. pneumoniae 9(81.8%), Enterococcus species 8(80%), P. aeruginosa 7 (77.8%) and CoNs 7(87.5%). This was comparable with a study conducted in Addis Ababa K. pneumoniae (83.5%) and E. coli (66.2%) (56), Equatorial Guinea in E. coli (74.4%) (48); in Sierra Leone K. pneumoniae (73.3%) and E. coli (61.5%) (47); in Gondar K. pneumoniae was (95.6%) and in E. coli was (92.9%) (3); in Khartoum-Sudan E. coli

(92.2%)(57); and in Equatorial Guinea in K. pneumoniae (91.7%) (48). The MDR level among E. coli (50.2%) in Dessie is lower than our study(51). The difference in MDR level among K. pneumoniae and E. coli in our study might be due to the fact that most K. pneumoniae were isolated from blood specimens that were collected from inpatients in the hospitals.

In this study, there was no statistically significant association between significant bacteriuria and in patients' residence, marital status, dysuria, frequency of urination, flank pain, and use of cotrimoxazole as prophylaxis However, age, gender, occupation, educational status, current CD4+ count (cells/mm3), previous hospitalization and diabetes status, history of catheterizations, and urinary tract urgency had a statistically significant association with significant bacteriuria. Similar findings were reported from Addis Jimma Ababa and Harar (25, 28, 30). Furthermore, Individuals age >44year [AOR=1.56, 95%CI: 1.11-9.35, P=0.001], being female [AOR=3.29, 95%CI: 1.26- 13.41, P=0.002], being daily labor [AOR=4.54, 95%CI: 1.41-21.71, P=0.003], being farmer [AOR=6.54, 95%CI: 1.21-23.51, P=0.004], participants unable to read and write [AOR=4.58, 95%CI: 1.21-13.58, P=0.001], HIV patients with CD4 count of ≤ 200 cells/mm3 (AOR: 7.44; 95%CI: 1.64-27.21, P=0.001) and CD4 count of 201-350 cells/mm3 (AOR: 4.55; 95%CI: 1.36- 18.28, P=0.009), patients who had chronic diabetics (AOR: 4.80; 95%CI: 1.85-16.31), patients who had chronic diabetics (AOR: 4.80; 95%CI: 1.85-16.31, p=0.009), Patients having history of hospitalization (AOR: 6.27; 95%CI: 1.25-15.17, p=0.002) and patients who had urgency of urinations (AOR: 6.51; 95%CI: 1.75-36.31, p=0.0025). This finding was similar to studies conducted elsewhere [26, 22, 27, and 29]. The explanation for the inverse relationship of UTI and a CD4+ count is unknown; it is probably due to the impaired immunity at a declining CD4+count that makes it easier for bacterial pathogens to adhere to the urinary epithelium.

**Limitation of the study**

However, the study was a single hospital-based study and might not represent all HIV infected patients and did not include control group due to budget constraints. We did not attempt to identify other causative agents (anaerobic bacteria, and fungus) that would have made a significant contribution to a true prevalence of UTI in HIV-positive patients.

## Conclusions and Recommendations

The asymptomatic community's acquired UTI is high, with an overall prevalence of 26.6% (92/346). From the isolated pathogens, 21.67% (75/346) were bacterial pathogens, while 4.9% (17/346) were fungal pathogens. Among bacterial pathogens, *E. coli*, the most frequent bacterium, followed by *K. pneumoniae, enterococcus species*, and *P. aeruginosa*, accounting for, respectively, the overall prevalence of MDR bacteria was 61(81.3%). About 13 (81.3%) of E. coli, 9(81.8%) of *K. pneumoniae*, 8(80%) of *Enterococcus species*, 7 (77.8%) of *P. aeruginosa*, and *CoNs* 7(87.5%). Most Gram-negative and Gram-positive isolates were resistant to tetracycline, trimethoprim-sulfamethoxazole, penicillin, chloramphenicol, and Nitrofurantoin, respectively. However, uropathogens were susceptible to amikacin, it was more effective against these pathogens. Patients with low CD4 counts were more at risk of developing bacterial urinary tract infections, previous hospitalization and diabetes status, and having a history of catheterizations than their counterparts. Management of UTI among symptomatic HIV patients should be supported by laboratory results of urine culture. Regular monitoring of antimicrobial resistance patterns should be undertaken by healthcare providers. Individuals at risk for bacterial associated UTI should have good adherence to ART and their CD4 levels should be monitored on a regular basis. Health information dissemination should be given about UTI and the habit of drug use for HIV positive patients.

## List of abbreviation and acronyms

AIDS; Acquired immune deficiency syndrome, ARTC; Attending antiretroviral therapy clinic, ART; Antiretroviral therapy, ATCC; American Type Culture collection, Ba; Blood agar, CFU; Colony Forming Unit, CLED; Cysteine Lactose Electrolyte Deficient, CONS; Coagulase negative Staphylococci, HAART; Highly active antiretroviral therapy, HIV; Human Immunodeficiency Virus, MACA; MacConkey agar, MDR; Multi-Drug Resistant, MSU; Midstream urine, OIS; Opportunistic infections, UTI; Urinary Tract Infection, VCT Voluntary counseling and Testing.

### Data availability statement

Data supporting the conclusions of this article are within the manuscript and is available on reasonable request from the principal investigators due to ethical reasons.

## Funding

No external funds were obtained

## Competing interests

The authors declare that they have no competing interest.

## Authors' contributions

Conceived and designed the experiments: Mihret Tilahun, Chernet Belayhun, Abdurrahman Seid, Bekele Sharew, Tewodros Dessie, Habtamu Mekonen ; Performed the experiments: Mihret Tilahun, Abdurrahman Seid, Agumas Shibabaw; Analyzed the data: Mihret Tilahun, Abdurrahman Seid, Daniel G/tsadik, Mesfin Fiseha, Melkam Tesfaye, Alemu Gedefie, Melaku Ashagrie Belete; Wrote and edited the manuscript: Mihret Tilahun, Abdurrahman Seid, Bekele Sharew, Alemu Gedefie, Habtu Debash, Agumas Shibabaw, Melaku Ashagrie Belete and Habtamu Mekonnen. All authors read and approved the final manuscript.

## Acknowledgement

The authors would like to acknowledge Department of Medical Laboratory Science, College of Medicine and Health Sciences, Wollo University for providing the laboratory set up and facilities to conduct the experiments. Dessie comprehensive specialized hospital and all study participants are gratefully acknowledged for their kind cooperation during data collection.

## References


1. Çoban B, Ülkü N, Kaplan H, Topal B, Erdoğan H, Baskın E. Five-year assessment of causative agents and antibiotic resistances in urinary tract infections. Turkish Archives of Pediatrics/Türk Pediatri Arşivi. 2014;49(2):124.
2. Flores-Mireles AL, Walker JN, Caparon M, Hultgren SJ. Urinary tract infections: epidemiology, mechanisms of infection and treatment options. Nature reviews microbiology. 2015;13(5):269-84.
3. Eshetie S, Unakal C, Gelaw A, Ayelign B, Endris M, Moges F. Multidrug resistant and carbapenemase producing Enterobacteriaceae among patients with urinary tract infection at referral Hospital, Northwest Ethiopia. Antimicrobial resistance and infection control. 2015;4(1):1-8.
4. Ahmed SS, Shariq A, Alsalloom AA, Babikir IH, Alhomoud BN. Uropathogens and their antimicrobial resistance patterns: Relationship with urinary tract infections. International Journal of Health Sciences. 2019;13(2):48.
5. Rao P, Chennu M, Vennila T, Kosanam S, Ponsudha P, Suriyakrishnaan K, et al. Assessment of Bacterial Isolates from the Urine Specimens of Urinary Tract Infected Patient. BioMed Research International. 2022;2022.
6. Paschke AA, Zaoutis T, Conway PH, Xie D, Keren R. Previous antimicrobial exposure is associated with drug-resistant urinary tract infections in children. Pediatrics. 2010;125(4):664-72.
7. Burton RJ, Albur M, Eberl M, Cuff SM. Using artificial intelligence to reduce diagnostic workload without compromising detection of urinary tract infections. BMC medical informatics and decision making. 2019;19(1):1-11.
8. Frank-Peterside N, Okerentugba P, Nwodo C, Okonko I. Prevalence of bacterial uropathogens in a cohort of HIV-positive males in Port Harcourt, Nigeria. Cancer Biology. 2013;3(4):12-7.
9. Birhanu MY, Habtegiorgis SD, Gietaneh W, Alemu S, Tsegaye TB, Bekele GM, et al. Magnitude and associated factors of urinary tract infections among adults living with HIV in Ethiopia. Systematic review and meta-analysis. PloS one. 2022;17(4):e0264732.
10. Bitew A, Zena N, Abdeta A. Bacterial and fungal profile, antibiotic susceptibility patterns of bacterial pathogens and associated risk factors of urinary tract infection among symptomatic pediatrics patients attending St. Paul's Hospital Millennium Medical College: a cross-sectional study. Infection and drug resistance. 2022;15:1613.
11. Foxman B. Urinary tract infection syndromes: occurrence, recurrence, bacteriology, risk factors, and disease burden. Infectious Disease Clinics. 2014;28(1):1-13.
12. Islam MA, Islam MR, Khan R, Amin MB, Rahman M, Hossain MI, et al. Prevalence, etiology and antibiotic resistance patterns of community-acquired urinary tract infections in Dhaka, Bangladesh. Plos one. 2022;17(9):e0274423.

13. Haider MZ, Annamaraju P. Bladder Catheterization. StatPearls [Internet]: StatPearls Publishing; 2022.
14. Magiorakos A-P, Srinivasan A, Carey RB, Carmeli Y, Falagas M, Giske C, et al. Multidrug-resistant, extensively drug-resistant and pandrug-resistant bacteria: an international expert proposal for interim standard definitions for acquired resistance. Clinical microbiology and infection. 2012;18(3):268-81.
15. Bigwan E, Wakjissa F. Prevalence of urinary tract infections among HIV patients attending a non-governmental health facility in Jos, Plateau State, Nigeria. International Journal of Biomedical and Advance Research. 2013;4(8):528-33.
16. Murray PR, Rosenthal KS, Pfaller MA. Medical microbiology E-book: Elsevier Health Sciences; 2020.
17. Pezzlo M. Laboratory diagnosis of urinary tract infections: guidelines, challenges, and innovations. Clinical Microbiology Newsletter. 2014;36(12):87-93.
18. Bullock NO, Aslanzadeh J. Biochemical profile-based microbial identification systems. Advanced techniques in diagnostic microbiology: Springer; 2013. p. 87-121.
19. Malik UM, Khan AB, Satti ML. Comparative evaluation of CHROMagar and API 20C AUX in isolation and identification of Candida species. Journal of Islamic International Medical College (JIIMC). 2018;13(2):85-90.
20. Institute CLS. CLSI Performance standard for antimicrobial susceptibility testing, M100. 2021.
21. Behzadi P, Urbán E, Matuz M, Benkő R, Gajdács M. The role of gram-negative bacteria in urinary tract infections: current concepts and therapeutic options. Advances in Microbiology, Infectious Diseases and Public Health. 2020:35-69.
22. Fatima SS, Mussaed EA. Urinary tract infection. Bacterial Identification and Drug Susceptibility Patterns in Pregnant and Non Pregnant UTI Patients: Springer; 2018. p. 1-22.
23. Nigussie D, Amsalu A. Prevalence of uropathogen and their antibiotic resistance pattern among diabetic patients. Turkish Journal of Urology. 2017;43(1):85.
24. Nabaigwa BI, Mwambi B, Okiria J, Oyet C. Common uropathogens among diabetic patients with urinary tract infection at Jinja Regional Referral Hospital, Uganda. African Journal of Laboratory Medicine. 2018;7(1):1-3.
25. Debalke S, Cheneke W, Tassew H, Awol M. Urinary tract infection among antiretroviral therapy users and nonusers in Jimma University Specialized Hospital, Jimma, Ethiopia. International journal of microbiology. 2014;2014.
26. Girma A, Aemiro A. The Bacterial Profile and Antimicrobial Susceptibility Patterns of Urinary Tract Infection Patients at Pawe General Hospital, Northwest Ethiopia. Scientifica. 2022;2022.
27. Tessema NN, Ali MM, Zenebe MH. Bacterial associated urinary tract infection, risk factors, and drug susceptibility profile among adult people living with HIV at Haswassa University Comprehensive Specialized Hospital, Hawassa, Southern Esthiopia. Scientific Reports. 2020;10(1):1-9.

28. Getu Y, Ali I, Lema T, Belay H, Yeshetela B. Bacteriuria and antimicrobial susceptibility pattern among HIV patients attending ALERT Center, Addis Ababa, Ethiopia. Am J Health Res. 2017;5(3):76-82.
29. Fenta G, Legese M, Weldearegay G. Bacteriuria and their antibiotic susceptibility patterns among people living with HIV attending Tikur Anbessa Specialized and Zewditu Memorial Hospital ART Clinics, Addis Ababa, Ethiopia. J Bacteriol Parasitol. 2016;7(05).
30. Marami D, Balakrishnan S, Seyoum B. Prevalence, Antimicrobial Susceptibility Pattern of Bacterial Isolates, and Associated Factors of Urinary Tract Infections among HIV- Positive Patients at Hiwot Fana Specialized University Hospital, Eastern Ethiopia. Canadian Journal of Infectious Diseases and Medical Microbiology. 2019;2019.
31. Okechukwu A, Thairu Y. Bacteria urinary tract infection in HIV-infected children and adolescents in Abuja, Nigeria: a cross-sectional study. African Journal of Clinical and Experimental Microbiology. 2019;20(4):306-14.
32. Iweriebor B, Obi C, Akinyemi O, Ramalivhana N, Hattori T, Okoh A. Uropathogens isolated from HIV-infected patients from Limpopo Province, South Africa. African Journal of Biotechnology. 2012;11(46):10598-604.
33. Xavier TF, Auxilia A, Kannan M. Isolation and characterization of UTI pathogens from HIV positive patients of Karur District, Tamil Nadu, India. Int J Curr Microbiol Appl Sci. 2015;4(1):558-63.
34. Sharma V, Gupta V, Mittal M. Prevalence of uropathogens in diabetic patients and their antimicrobial susceptibility pattern. Natl J lab Med. 2012;1(1):26-8.
35. Odabasi Z, Mert A. Candida urinary tract infections in adults. World journal of urology. 2020;38(11):2699-707.
36. Behzadi P, Behzadi E, Ranjbar R. Urinary tract infections and Candida albicans. Central European journal of urology. 2015;68(1):96-101.
37. Achkar JM, Fries BC. Candida infections of the genitourinary tract. Clinical microbiology reviews. 2010;23(2):253-73.
38. Erdem I, Ali RK, Ardic E, Omar SE, Mutlu R, Topkaya AE. Community-acquired lower urinary tract infections: Etiology, antimicrobial resistance, and treatment results in female patients. Journal of global infectious diseases. 2018;10(3):129.
39. Alemu A, Dagnew M, Alem M, Gizachew M. Uropathogenic bacterial isolates and their antimicrobial susceptibility patterns among HIV/AIDS patients attending Gondar University Specialized Hospital Gondar, Northwest Ethiopia. 2013.
40. Biradar SK, Doddamani P. Prevalence and antibiogram of uropathogens in a tertiary care hospital. World J Pharmaceut Res. 2013;2:1534-43.

41. Ifeanyichukwu I, Emmanuel N, Chika E, Anthonia O, Esther U-I, Ngozi A, et al. Frequency and antibiogram of uropathogens isolated from urine samples of HIV infected patients on antiretroviral therapy. American Journal of BioScience. 2013;1(3):50-3.
42. Ejerssa AW, Gadisa DA, Orjino TA. Prevalence of bacterial uropathogens and their antimicrobial susceptibility patterns among pregnant women in Eastern Ethiopia: hospital-based cross-sectional study. BMC women's health. 2021;21(1):1-15.
43. Fierer J, Looney D, Kok M, Pechère J-C. Nature and pathogenicity of micro-organisms. Infectious diseases. 2010:3.
44. Nwadioha S, Nwokedi E, Ikeh I, Egesie J, Kashibu E. Antibiotic susceptibility pattern of uropathogenic bacterial isolates from AIDS patients in a Nigerian tertiary hospital. J Med Med Sci. 2010;1(11):530-4.
45. Desta MK. College of Health Sciences School of Allied Health Sciences Department of Medical Laboratory Sciences: Addis Ababa University; 2017.
46. Mansouri S, Abbasi S. Prevalence of multiple drug resistant clinical isolates of extended-spectrum beta-lactamase producing Enterobacteriaceae in Southeast Iran. 2010.
47. Leski TA, Taitt CR, Bangura U, Stockelman MG, Ansumana R, Cooper WH, et al. High prevalence of multidrug resistant Enterobacteriaceae isolated from outpatient urine samples but not the hospital environment in Bo, Sierra Leone. BMC infectious diseases. 2016;16(1):1-9.
48. Shatalov A. Prevalence and antibiotic resistance pattern of Escherichia coli and Klebsiella pneumoniae in urine tract infections at the La Paz Medical center, Malabo, Equatorial Guinea. Open Journal of Medical Microbiology. 2015;5(04):177.
49. Adler A, Katz DE, Marchaim D. The continuing plague of extended-spectrum β-lactamase–producing Enterobacteriaceae infections. Infectious Disease Clinics. 2016;30(2):347-75.
50. Kim H-R, Hwang SS, Kim HJ, Lee SM, Yoo C-G, Kim YW, et al. Impact of extensive drug resistance on treatment outcomes in non-HIV-infected patients with multidrug-resistant tuberculosis. Clinical Infectious Diseases. 2007;45(10):1290-5.
51. Kibret M, Abera B. Antimicrobial susceptibility patterns of E. coli from clinical sources in northeast Ethiopia. African health sciences. 2011;11:40-5.
52. Moges F, Mengistu G, Genetu A. Multiple drug resistance in Urinary pathogens at Gondar College of Medical science hospital, Ethiopia. East African medical journal. 2002;79(8):415-20.
53. Yadav KK, Adhikari N, Khadka R, Pant AD, Shah B. Multidrug resistant Enterobacteriaceae and extended spectrum β-lactamase producing Escherichia coli: a cross-sectional study in National Kidney Center, Nepal. Antimicrobial resistance and infection control. 2015;4(1):1-7.
54. Murugesh K, Deepa S, Ravindranath C, Venkatesha D. Multi drug resistant uropathogens in HIV: Are they a threat to community? International Journal of Scientific Study. 2014;2(3):38-42.

55. Biadglegne F, Abera B. Antimicrobial resistance of bacterial isolates from urinary tract infections at Felge Hiwot Referral Hospital, Ethiopia. The Ethiopian Journal of Health Development. 2009;23(3).
56. Teklu DS, Negeri AA, Legese MH, Bedada TL, Woldemariam HK, Tullu KD. Extended-spectrum beta-lactamase production and multi-drug resistance among Enterobacteriaceae isolated in Addis Ababa, Ethiopia. Antimicrobial Resistance & Infection Control. 2019;8(1):1-12.
57. Ibrahim M, Bilal N, Hamid M. Increased multi-drug resistant Escherichia coli from hospitals in Khartoum state, Sudan. African health sciences. 2012;12(3):368-75.

## Figure legend

**Figure 1. Frequency of uropathogens isolates among Community Acquired UTI HIV-seropositive patients attending at Dessie Referral Hospital, Dessie, Ethiopia From February 1, 2021 to march 30, 2021.**

Figure Click here to access/download;Figure;Figure one.jpg

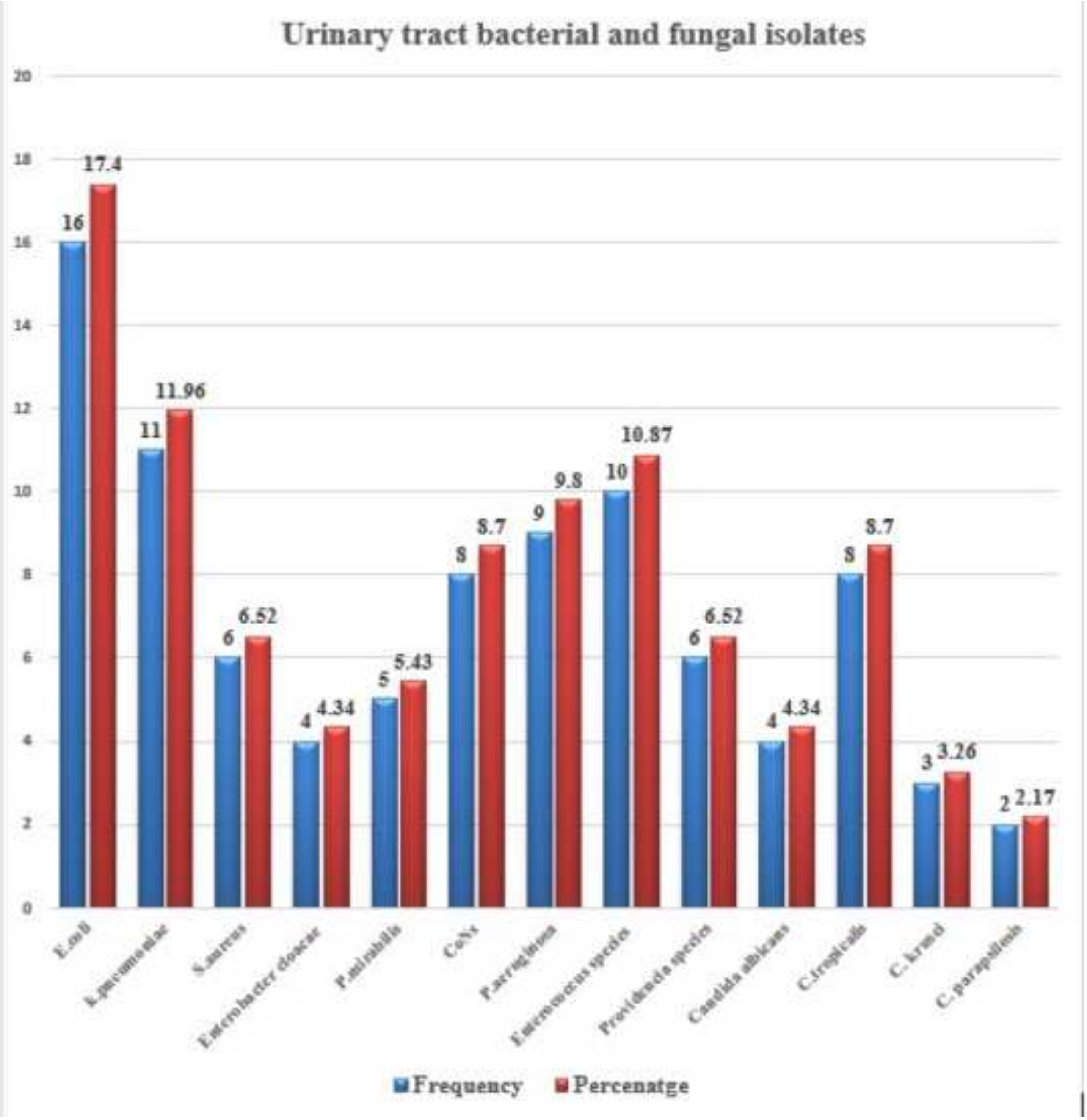

Click here to access/download
**Supporting Information**
Ethical clearance.pdf
