# Supplementary material for: Uro-pathogens: Multidrug resistance and associated factors of community-acquired UTI among HIV patients attending antiretroviral therapy in Dessie Comprehensive Specialized Hospital, Northeast Ethiopia
Source: PLoS One. 2024 May 31;19(5):e0296480. doi: 10.1371/journal.pone.0296480 (PMC11142584; doi:10.1371/journal.pone.0296480)
Supplement: Attachment — Submitted filename: PONE-D-22-31717_R2.pdf [file pone.0296480.s006.pdf]

PLOS ONE

Uropathogens: Multidrug resistance and risk factors of community-acquired UTI among HIV patients attending antiretroviral therapy clinics of Northeast Ethiopia

--Manuscript Draft--

**Manuscript Number:**	PONE-D-22-31717R2
**Article Type:**	Research Article
**Full Title:**	Uropathogens: Multidrug resistance and risk factors of community-acquired UTI among HIV patients attending antiretroviral therapy clinics of Northeast Ethiopia
**Short Title:**	Uropathogens, Drug resistance profile of pathogen, risk factors of urinary tract infection
**Corresponding Author:**	Mihret Tilahun, MSc<br>Wollo University<br>Dessie, ETHIOPIA
**Keywords:**	Bacteriuria, Candidauria, Uropathogens, community-acquired UTI, Multidrug resistance, Ethiopia
**Abstract:**	Background: Urinary tract infections are the common bacterial and fungal infections in humans, occurring both in the community and in immunocompromised patients in health care settings. UTI has significant health impact on HIV-infected patients. Drug resistance pathogens is now widespread and poses a serious clinical risk and causes urinary tract infection. This study investigated uropathogens, multidrug resistance pattern of bacteria and associated factors of community-acquired urinary tract infection among HIV positive patients attending antiretroviral therapy clinic of Northeast Ethiopia from February 1, 2021, to March 30, 2021.<br>Methods: Institutional based cross-sectional study was conducted at Dessie comprehensive specialized Hospital. Socio-demographic and clinical data were collected by using structured questionnaire from HIV patients suspected of community-acquired urinary tract infection. About 10 ml of clean-catch midstream urine was collected and inoculated into Blood agar, MacConkey, and Cysteine lactose electrolyte deficient media. Gram stain and biochemical tests were performed to identify isolates and antimicrobial susceptibility pattern was performed on disc diffusion techniques. Data were entered and analyzed using SPSS version-25. Both bivariate and multivariable logistic regression analysis was performed and a P value of < 0.05 with an adjusted odds ratio with their 95% confidence interval (CI) was used as statistically significant associations.<br>Results: From the total 346 study participants, 92 (26.6%) were culture positive and 75 (81.52%) were bacterial and 17 (18.48%) were fungal pathogens. From a total of 75 bacteria isolates 51(68%) were Gram-negative bacteria and the most commonly isolated bacteria were E. coli 16 (21.33%) followed by K. pneumoniae 11(14.67%) and enterococcus species 10(10.87. Of the 17 fungal isolates of fungi, 8(47.1%) were represented by C. tropicalis. The overall prevalence of Multi-Drug Resistance bacteria was 61(81.3%). About 13 (81.3%) of E. coli, 9(81.8%) of K. pneumoniae, 8(80%) of Enterococcus species, 7 (77.8%) of P. aeruginosa, and CoNs 7(87.5%) were the most frequently exhibited multi-drug Resistance. Amikacin, and gentamicin were effective against Gram-negative uropathogens. Participants with age >44year, being female, being daily labor, being farmer, unable to read and write, patients with CD4 count of ≤ 200 cells/mm3 and CD4 count of 201-350 cells/mm3, who had chronic diabetics, patients having a history of hospitalization and who had urgency of urinations were statistically significant association with significant urinary tract infections.<br>Conclusion: The burden of community acquired urinary tract infections among HIV patients is alarmingly increased. Therefore, behavior change communications might be considered for promoting the health status of HIV patients. Moreover, CD4 level monitoring and therapeutics selection based on microbiological culture are quite advisable for management of urinary tract infections of HIV patients
**Order of Authors:**	Mihret Tilahun, MSc
	Mesfin Fiseha
	Mihreteab Alebachew

Powered by Editorial Manager® and ProduXion Manager® from Aries Systems Corporation

	Alemu Gedefie
	Endris Ebrahim
	Melkam Tesfaye
	Melaku Ashagrie Belete
	Abdurahaman Seid
	Daniel Gebretsadik
	Ermiyas Alemayehu
	Wondmagegn Demsiss
	Bekele Sharew, PhD
	Agumas Shibabaw, PhD
	Habtamu Mekonnen
	Tewodros Dessie
**Response to Reviewers:**	If there is comments I will egger to revise
**Additional Information:**	
**Question**	**Response**
**Financial Disclosure**<br><br>Enter a financial disclosure statement that describes the sources of funding for the work included in this submission. Review the submission guidelines for detailed requirements. View published research articles from *PLOS ONE* for specific examples.<br><br>This statement is required for submission and **will appear in the published article** if the submission is accepted. Please make sure it is accurate.	No external funds were obtained only internal support was obtained from Wollo university. The funders had no role in study design, data collection and analysis, decision to publish, or preparation of the manuscript.

Powered by Editorial Manager® and ProduXion Manager® from Aries Systems Corporation

**Unfunded studies**<br>Enter: *The author(s) received no specific funding for this work.*<br><br>**Funded studies**<br>Enter a statement with the following details:<br>• Initials of the authors who received each award<br>• Grant numbers awarded to each author<br>• The full name of each funder<br>• URL of each funder website<br>• Did the sponsors or funders play any role in the study design, data collection and analysis, decision to publish, or preparation of the manuscript?<br>• **NO** - Include this sentence at the end of your statement: *The funders had no role in study design, data collection and analysis, decision to publish, or preparation of the manuscript.*<br>• **YES** - Specify the role(s) played.<br><br>* typeset	
**Competing Interests**<br><br>Use the instructions below to enter a competing interest statement for this submission. On behalf of all authors, disclose any competing interests that could be perceived to bias this work—acknowledging all financial support and any other relevant financial or non-financial competing interests.<br><br>This statement is required for submission and **will appear in the published article** if the submission is accepted. Please make sure it is accurate and that any funding sources listed in your Funding Information later in the submission form are also declared in your Financial Disclosure statement.<br><br>View published research articles from *PLOS ONE* for specific examples.	The authors declare that they have no competing interest.

**NO authors have competing interests**<br><br>Enter: *The authors have declared that no competing interests exist.*<br><br>**Authors with competing interests**<br><br>Enter competing interest details beginning with this statement:<br><br>*I have read the journal's policy and the authors of this manuscript have the following competing interests: [insert competing interests here]*<br><br>* typeset	
**Ethics Statement**<br><br>Enter an ethics statement for this submission. This statement is required if the study involved:<br><br>• Human participants<br>• Human specimens or tissue<br>• Vertebrate animals or cephalopods<br>• Vertebrate embryos or tissues<br>• Field research<br><br>Write "N/A" if the submission does not require an ethics statement.<br><br>General guidance is provided below. Consult the submission guidelines for detailed instructions. **Make sure that all information entered here is included in the Methods section of the manuscript.**	Ethical clearance and permissions were obtained from the ethical review committee of Wollo University College of Medicine and Health Sciences with protocol number CMHS 365/2021 and a letter of support was written to Dessie Referral Hospital. Permission was obtained from dessie comprehensive specialized hospitals. Written assent was also obtained from family and surrogates to obtain information those who can't give data. Information obtained from the study participants were kept confidentially. positives results were communicated and given to attending physicians for appropriate treatment

Powered by Editorial Manager® and ProduXion Manager® from Aries Systems Corporation

**Format for specific study types**<br><br>**Human Subject Research (involving human participants and/or tissue)**<br>• Give the name of the institutional review board or ethics committee that approved the study<br>• Include the approval number and/or a statement indicating approval of this research<br>• Indicate the form of consent obtained (written/oral) or the reason that consent was not obtained (e.g. the data were analyzed anonymously)<br><br>**Animal Research (involving vertebrate animals, embryos or tissues)**<br>• Provide the name of the Institutional Animal Care and Use Committee (IACUC) or other relevant ethics board that reviewed the study protocol, and indicate whether they approved this research or granted a formal waiver of ethical approval<br>• Include an approval number if one was obtained<br>• If the study involved *non-human primates*, add *additional details* about animal welfare and steps taken to ameliorate suffering<br>• If anesthesia, euthanasia, or any kind of animal sacrifice is part of the study, include briefly which substances and/or methods were applied<br><br>**Field Research**<br><br>Include the following details if this study involves the collection of plant, animal, or other materials from a natural setting:<br>• Field permit number<br>• Name of the institution or relevant body that granted permission	
**Data Availability**<br><br>Authors are required to make all data underlying the findings described fully available, without restriction, and from the time of publication. PLOS allows rare exceptions to address legal and ethical concerns. See the PLOS Data Policy and FAQ for detailed information.	Yes - all data are fully available without restriction

Powered by Editorial Manager® and ProduXion Manager® from Aries Systems Corporation

A Data Availability Statement describing where the data can be found is required at submission. Your answers to this question constitute the Data Availability Statement and **will be published in the article**, if accepted.<br><br>**Important:** Stating 'data available on request from the author' is not sufficient. If your data are only available upon request, select 'No' for the first question and explain your exceptional situation in the text box.<br><br>Do the authors confirm that all data underlying the findings described in their manuscript are fully available without restriction?	
**Describe where the data may be found in full sentences. If you are copying our sample text, replace any instances of XXX with the appropriate details.**<br><br>• If the data are **held or will be held in a public repository**, include URLs, accession numbers or DOIs. If this information will only be available after acceptance, indicate this by ticking the box below. For example: *All XXX files are available from the XXX database (accession number(s) XXX, XXX.*).<br>• If the data are all contained **within the manuscript and/or Supporting Information files**, enter the following: *All relevant data are within the manuscript and its Supporting Information files.*<br>• If neither of these applies but you are able to provide **details of access elsewhere**, with or without limitations, please do so. For example:<br><br>*Data cannot be shared publicly because of [XXX]. Data are available from the XXX Institutional Data Access / Ethics Committee (contact via XXX) for researchers who meet the criteria for access to confidential data.*<br><br>*The data underlying the results presented in the study are available from (include the name of the third party*	No

and contact information or URL).<br>• This text is appropriate if the data are owned by a third party and authors do not have permission to share the data.<br><br>* typeset	
Additional data availability information:	Tick here if your circumstances are not covered by the questions above and you need the journal’s help to make your data available.; Tick here if the URLs/accession numbers/DOIs will be available only after acceptance of the manuscript for publication so that we can ensure their inclusion before publication.

Manuscript
Click here to access/download;Manuscript;updated Manuscript .docx

Bacterial and fungal profile, multidrug resistance profile of bacterial pathogens and associated risk factors of community-acquired urinary tract infection among HIV positive patients attending Antiretroviral Therapy Clinics of Northeast Ethiopia.

Uropathogens, drug resistance and associated factors of communit

Mihret Tilahun[1*], Mesfin Fiseha[1], Mihreteab Alebachew[1], Alemu Gedefie[1], Endris Ebrahim[1], Melkam Tesfaye[1], Melaku Ashagrie Belete[1], Abdurahaman Seid [1], Daniel Gebretsadik [1], Wondmagegn Demsiss[1], Bekele Sharew[1], Agumas Shibabaw[1], Habtamu Mekonnen[2] Tewodros Dessie [2]

[1]Department of Medical Laboratory Sciences, College of Medicine and Health Science, Wollo University, Dessie, Ethiopia

[2] Amhara Public Health Institute Dessie branch

Authors' information:

Mihret Tilahun (MSc in Medical Microbiology): tilahunmihret21@gmail.com

Mesfin Fiseha (MSc in hematology and immunohematology): mesfinfiseha40@gmail.com

Alemu Gedefie (MSc in Medical Microbiology): alemugedefie@gmail.com

Mihreteab Alebachew (MSc in medical parasitology and vector control): mihreteab71@gmail.com

Endris Ebrahim (MSc in immunology): endris.index@gmail.com

Melkam Tesfaye (PhD fellow in infectious diseases: Melkam66@gmail.com

Melaku Ashagrie Belete (MSc in Medical Microbiology): melakuashagrie@gmail.com

Abdurahaman Seid (MSc., Asst Prof in Medical Microbiology): gechdk2000@yahoo.com

Daniel Gebretsadik (MSc., Asst Prof in Tropical and infectious diseases): gebretsadikd@gmail.com

Wondmagegn Demsiss (MSc., Asst Prof in Medical Microbiology): *wonde1980@gmail.com*

Bekele Sharew PhD, Asst Professor in Medical Microbiology): bsharew@gmail.com

Agumas Shibabaw (PhD, Asst Professor in Medical Microbiology): agumas2000@gmail.com

Habtamu Mekonnen (MSc in Medical Microbiology: habtamumeko36@gmial.com

Tewodros Dessie (MSc in Medical Microbiology: tedydess21@gmial.com

Corresponding author

Mihret Tilahun (MSc.)

Department of Medical Laboratory Science

College of Medicine and Health Sciences,
Wollo University, Dessie, Ethiopia
Telephone: +251- 920988307
Fax: +251 333115250
PO.BOX: 1145
Email: tilahunmihret21@gmail.com

Abstract

Background: Urinary tract infections (UTI) are one of the most common bacterial and fungal infections in humans, occurring both in the community and in health-care settings. UTI is a significant health issue among HIV-infected patients. Antimicrobial resistance is now widespread and poses a serious clinical risk.

Objective: This study investigated bacterial and fungal profile, multidrug resistance profile of bacterial pathogens and associated risk Factors of Community acquired urinary tract infection among HIV positive patients attending Antiretroviral Therapy Clinics of Northeast Ethiopia.

Methods: Institutional based cross-sectional study was conducted in Dessie Referral Hospital, from February 1, 2021 to March 30, 2021. Socio-demographical and clinical data were collected using structured questionnaires from community acquired urinary tract infection suspected HIV-Positive patients. About 10 ml Clean-catch midstream were collected and inoculated into Blood agar, MacConkey and Cysteine lactose electrolyte deficient. Biochemical tests were performed to identify isolates and antimicrobial susceptibility pattern was performed on disc diffusion techniques. Data were entered and analyzed using SPSS version-25. Descriptive statistics such as tables and bar graphs were used to summarize the findings. Logistic regression analysis was performed and the adjusted odds ratio (AOR) with their 95% confidence interval (CI) was used as statistically significant associations.

Results: Among the total 346 study participants, 180 (52.04%) were females. The overall 26.6% (92/346) of which 21.67% (75/346) were bacterial pathogens, while 4.9% (17/346) were fungal pathogens. From a total of 75 bacteria isolates 51(68%) were Gram-negative bacteria. The most commonly isolated Gram-negative bacteria were *E. coli* (21.33%) followed *K. pneumoniae* (14.67%). In addition, From the 17 yeasts isolate of fungi 47.1% (8/17) were represented *by C. tropicalis*. The overall prevalence of MDR bacteria was 61(81.3%). About 13 (81.3%) of E. coli, 9(81.8%) of K. pneumoniae, 8(80%) of Enterococcus species, 7 (77.8%) of P. aeruginosa, and CoNs 7(87.5%) were the most frequently exhibited MDR. age, gender, occupation, educational status, current CD4+ count (cells/mm3), previous hospitalization and diabetes status, history of catheterizations, and urinary tract urgency had a statistically significant association with significant bacteriuria.

Conclusion

There is high asymptomatic uropathogens and Multidrugrestistnace pathogens The most dominant bacterial isolates were *E. coli* followed by *K. pneumoniae* and *S. aureus*. Amikacin, and gentamicin were effective against gram negative uropathogens. Management of UTI among symptomatic HIV patients should be supported by laboratory result of urine culture. Regular monitoring of antimicrobial resistance patterns should be assessed by health care providers.

Keywords: Uropathogens, community-acquired UTI, Multidrug resistance, Ethiopia

Introduction

Urinary tract infections (UTI) are caused by gram-negative and gram-positive bacteria, as well as certain fungi. Uro-pathogenic *Escherichia coli* is the most common cause of both uncomplicated and complicated UTIs. *Escherichia coli* is the most common agent involved in uncomplicated UTIs, followed by *Klebsiella pneumoniae*, *Staphylococcus saprophyticus*, *Enterococcus faecalis*, *group B streptococcus*, *Proteus mirabilis*, *Pseudomonas aeruginosa*, *Staphylococcus aureus*, and Candida spp (1). Following UPEC as the most common causative agent for complicated UTIs, the order of prevalence for causative agents is Enterococcus spp., K. pneumoniae, Candida spp., S. aureus, P. mirabilis, P. aeruginosa, and GBS(2).

The emergence of antibiotic resistance in the treatment of urinary tract infections is a major public health issue worldwide, particularly in developing countries. Antimicrobial treatment of urinary tract infections in HIV patients is required (3). Newly, the most common pathogens of UTIs have developed resistance to most chemotherapeutic agents; their antimicrobial susceptibilities are highly predictable (4). Furthermore, the three most commonly isolated bacteria (Escherichia coli, Klebsiella spp, and Proteus spp) had resistance rates to amoxicillin and tetracycline of 80.1%-90.0% and sensitivity rates of 0 to 25% to Nitrofurantoin, ciprofloxacin, and gentamicin of 0 to 25%. Furthermore, the MDR profile of the isolates revealed that 152 (47.85%) of the isolates were resistant to two or more antimicrobials(5). Resistance to these antimicrobial agents in UTI cases will thus have an impact on future treatment and management of the infection with these drugs. Adequate treatment and control of these conditions necessitate a thorough understanding of the causative bacterial species as well as their susceptibility and resistance to antimicrobial agents. Since the majority of treatments are initiated empirically, knowledge of the organisms, their epidemiological characteristics, and antibacterial susceptibility is required (6).

Every year, approximately 150 million people around the world are diagnosed with UTI, costing the global economy more than a billion dollars. UTIs are the most common bacterial infections and account for a large portion of the workload in clinical microbiology laboratories. It is also the most common infection in HIV patients in many health care settings (7).

UTIs, like respiratory infections, cause increased mortality, disability, hospitalization, and economic loss in the HIV population. Furthermore, it causes renal failure and hypertension. Furthermore, it is prone to nephritic complications such as urinary tract obstruction, which

causes renal colic, pelvic-ureteric junction obstruction, benign prostate hyperplasia, urethral strictures, and bladder dysfunction, which causes urinary stasis and, ultimately, infection (8).

Female gender, a history of recurrent UTI, a lower socioeconomic status, diabetes mellitus, anatomical or physiological genitourinary tract abnormalities, and a low CD4 count have all been linked to the occurrence of bacterial UTI. Because of the aforementioned distribution and determinants, UTI has the potential for recurrence and necessitates a longer treatment (9)

Despite numerous studies on bacterial UTI, antimicrobial susceptibility testing, and HIV infection-related factors in developed countries, there is little data on the burden of bacterial UTI and antimicrobial susceptibility pattern among UTI symptomatic HIV-infected patients in resource-limited settings, including Ethiopia in general, and no study was done in the study area in particular. As a result, the purpose of this study was to determine bacterial and fungal profile, multidrug resistance profile of bacterial pathogens and associated risk Factors of Community acquired urinary tract infection among HIV positive patients attending Antiretroviral Therapy Clinics of Northeast Ethiopia.

Methods and materials

Study design, area and period

From February 1, 2021 to March 30, 2021, a health facility-based cross-sectional study was conducted at Dessie Referral Hospital in Dessie town. Dessie is a town founded in total area of 15.08km2 (5.82 sq. m) and located in North Central Ethiopia, east of the Amhara Region. It has a latitude and longitude of 1108’N 39038’E with an elevation of between 2470 and 2,550 meters above sea level. Dessie is 401 km from Addis Ababa to the north and the distance from Dessie to Bahir-dar is 490 km. Dessie town has one public health institute laboratory providing different laboratory services to different areas of the region for both governmental and non-governmental health organizations. According to the Dessie town health administrative office, in the town, there are also 16 governmental health institutions (1 comprehensive specialized hospital, 1 general hospital, 8 health centers) and different private health institutions (3 general hospitals, 6 higher clinics). According to the Dessie town health administrative office, the hospital serves about 12 million people living in the catchment areas. The comprehensive specialized hospital serves about seven million people living in the catchment areas and has a 600 average daily patient flow also has 800 staff, among whom about 600 are health professionals. It has 600 beds, 8 wards, and different OPDs like adult OPD, pediatric OPD, emergency OPD, and TB & HIV

OPD. Also, the hospital provides different services like laboratory, pharmacy, imaging (X-ray, ultra-sound, city scan) and a comprehensive ART service for about 9,500 people.

Population

All Suspected community acquired UTI symptomatic HIV seropositive positive individuals living in Dessie town and nearby catchments were source of populations. All UTI symptomatic HIV-seropositive patients and visited ART clinic of Dessie Referral Hospital during the study period before 18 hours of admissions were target populations.

Eligibility criteria of study participants

The HIV positive adults who were ≥18 years old and complaining of UTI symptoms were included in the study before 18 hours of admission. However, patients who were on antibiotics for more than two weeks before specimen collection were excluded from the study, and admitted patients for more than 18 hours were excluded from the study.

Variables

Dependent variables included the prevalence of bacterial and fungal community acquired UTI and the antimicrobial susceptibility pattern of uro-pathogenic bacteria. While age, sex, marital status, educational level, residence, history of catheterization, ART status, symptoms of UTI (dysuria, urgency, frequency, flank pain and urine color), diabetes, CD4+cell count, Cotrimoxazole usage, and history of hospitalization were independent variables.

Sample size and sampling technique

The required sample size was determined by using single population proportion formula considering the following assumptions: Prevalence = 28.6% (prevalence of culture proven UTI among HIV seropositive and UTI symptomatic patients from previous study conducted in Ethiopia (10) . Sample size,

$$n = \frac{z^2 * P\,(1 - P)}{d^2}$$

Where: n= the required sample size Z = Z score for 95 % confidence interval = 1.96, p = prevalence=28.6% (0.286), d = tolerable error =5 %, 1-p=Q then, 0.714, n = $((1.96)^2$ x 0.286x (1-0.286))/ $(0.05)^2$**=314. With 10% contingency 32.** However, during the study period, a total of 346 UTI symptomatic HIV-seropositive patients and fulfilling the inclusion criteria were included consecutively.

Operational definitions

UTI: Urinary tract infections are one of the most common infections in humans that can happen anywhere along the urinary tract (11).

Community acquired urinary tract infection (CA-UTI); defined as an infection of the urinary tract that occurs in the community or within less than 48 hours of hospital admission and was not incubating at the time of hospital admission (12).

History of catheterization: the use of or insertion of a catheter into the bladder or participants who were catheterized prior to data collections(13).

History of hospitalization: participants who were taking to health facility and keeping there to 48hrs for treatment prior to data collections.

Multidrug resistance: A bacterium that is simultaneously resistant for three or more antimicrobial categories (14).

Data and specimen collection

A pretested structured questionnaire was used to collect data on sociodemographic characteristics and associated factors. The participants' recent CD4+ count was retrieved from their medical record. About 10 mL of voided clean-catch midstream fresh urine was collected from each participant by using a leak-proof and sterile wide-mouthed screw-capped container. These specimens were labeled and stored in a cold box (4°C) (15) and before being transported to the Wollo University College of Medicine and Health Sciences microbiology laboratory for analysis.

Isolation and identification of bacteria

Bacterial isolation and phenotypic characterizations were performed using the recommended culture and biochemical tests (16). A calibrated loop that delivers 0.001 mL of urine was used to inoculate each urine sample on to the Cysteine lactose electrolyte deficient agar (Oxoid Ltd, UK) . The plates were incubated aerobically at 37°C. After overnight incubation, plates with the growth of $\geq 10^4$-10^5CFU/mL (colony-forming units per milliliter) accompanied by signs and symptoms were considered significant. Gram stain was performed and sub cultured on to MacConkey agar (HiMediaTM) and 5% blood agar plates (HiMediaTM). However, plates that did not show growth after 24 hrs of incubation were incubated again for the next 24 hrs and discarded as negative if the colony count was not significant (17). From plates with significant

bacteria growth, the phenotypic characterization of bacteria was carried out using colony characteristics, gram reactions, and a series of biochemical reactions, including catalase, coagulase, oxidase, urease, indole, citrate utilization, lysine decarboxylase, glucose, lactose fermentation, gas and H2S production, and motility tests (18) .

Yeast Identification

Yeasts were identified using routine diagnostic methods such as Gram stain, Germ tube test, carbohydrate fermentation, and assimilation tests, as well as chromogenic medium (CHROMagar Candida medium, Biomerieux, France) according to the manufacturer's instructions (19).

Antimicrobial susceptibility testing (AST)

Antimicrobial susceptibility test was performed using a Kirby–Bauer disk diffusion method based on the Clinical and Laboratory Standards Institute (CLSI) recommendation (20). Three to five pure colonies of the similar appearance of each isolate were emulsified in 4 ml of sterile normal saline. The inoculum suspension was standardized by comparing the turbidity with 0.5 McFarland standards. A Muller-Hinton agar plate (HiMediaTM) was streaked with the prepared inoculum suspension using a sterile swab, and antibiotic discs were placed on each agar plate after 5 minutes using sterile forceps. The antimicrobials (Oxoid Ltd) that were used for bacterial susceptibility testing were, amoxicillin-clavulanic acid (AMC) 10µg, ampicillin (AMP) 10µg, amikacin (Amk) 30µg, cefotaxime (CTX) 30µg, ceftriaxone (CRO) 30µg, trimethoprim-sulphamethaxazole (SXT) 25µg, ciprofloxacin (CIP) 5µg, gentamycin (Gen) 10 µg, ceftazidime (CAZ) 30µg, nitrofurantoin (F) 300µg, tetracycline (TE) 30µg and penicillin (Pen) 10µg (20). Within 15 minutes after application of the discs, the plates were incubated at 35°c for 18 hours and the plates were examined for growth and the diameters of zone of inhibition (in mm) was measure by using a graduated caliper in millimeters and results interpreted as sensitive (S), intermediate (I), or resistance (R) (20).

Data quality management

The questionnaire was initially prepared in English, translated into the local language (Amharic) by language experts back to English by other experts to assure its consistency and accuracy. The questionnaire was pretested on 5% HIV-positive patients at Dessie health center, Dessie, Ethiopia. The quality of culture media was tested for sterility and performance. Sterility of culture media was checked by incubating overnight at 37 ºC without specimen inoculation. Any physical change like cracks, excess moisture, color, hemolysis, dehydration, contamination was

assessed & the expiration date was also checked. The new batch culture medium and antimicrobial disks were checked for performance and quality using the American Type Culture Collection (ATCC) reference strains such as *E. coli* (ATCC® 25922), *S. aureus* (ATCC® 25923), *Klebsiella pneumonia* (ATCC 700603) and *P. aeruginosa* (ATCC® 27853).

Statistical analysis

The data generated was entered every day into epi-data version 4.6.0.4. The data was then exported and analyzed using the Social Sciences Statistical Package (SPSS) version 25. The frequency and percentage descriptive statistics were calculated and presented using graphs and tables. A bivariable analysis was conducted in order to classify factors that separately affect the frequency of dependent variables. Variables with a P-value less than or equal to 0.25 in bivariable analysis were subjected to multi-variable analysis. adjusted odd ratio with a p value of <0.05 and 95% CI taken as statistically significant. Finally, the results were presented in words, graphs and tables.

Ethical considerations

Ethical clearance and permissions were obtained from the ethical review committee of Wollo University College of Medicine and Health Sciences with protocol number CMHS 365/2021 and a letter of support was written to Dessie Referral Hospital. Permission was obtained from dessie comprehensive specialized hospitals. Written assent was also obtained from family and surrogates to obtain information those who can't give data. Information obtained from the study participants were kept confidentially. positives results were communicated and given to attending physicians for appropriate treatment.

Results

Socio-demographic characteristics

A total of 346 HIV positive patients were included in this study. The age of the study participants ranged from 21 to 80 years, with a mean (±SD) age of 39.44 (±10.87) years, and 116 (33.5%) of them were in the age range of 35–44 years. About 180 (52.02%) of the respondents were females. Regarding educational status, 115 (33.2%) of the respondents had a primary level education. Concerning occupational status, 90 (26.01%) were self-employed. Concerning residences, 215 (62.1%) of them were urban dwellers, as shown in **(Table 1).**

Table 1. Sociodemographic characteristics of UTI symptomatic HIV positive patients attending at Dessie referral hospital, Dessie, Ethiopia (February 1, 2021- march 30, 2021).

Variable	Category	Culture Result		Frequency	Percentage
		Positive No (%)	Negative No (%)		
Sex	Male	42(25.3)	124(74.7)	166	(47.98)
	Female	50(27.8)	130(72.2)	180	(52.02)
Residence	Rural	62(28.8)	153(71.2)	215	62.1
	Urban	30(22.9)	101(77.1)	131	37.9
Age (in years)	18–24	10(18.9)	43(81.1)	53	15.3
	25–34	21(24.7)	64(75.3)	85	24.6)
	35–44	30(25.9)	86(74.1)	116	33.5
	>44	31(33.7)	61(66.3)	92	26.6
Educational status	Illiterate	42(36.5)	73(63.5)	115	33.2
	Primary	20(23.3)	66(76.7)	86	24.9
	Secondary	22(24.2)	69(75.8)	91	26.3
	College/University	8(14.8)	46(85.2)	54	15.6
Marital status	Married	45(37.2)	76(62.8)	121	35
	Unmarried	18(22.5)	62(77.5)	80	23.1
	Divorced	13(13.7)	82(86.3)	95	27.5
	Widowed	16(32)	34(68)	50	14.5
Occupation	House Wife	15(25.9)	43(74.1)	58	16.8
	Civil Servant	10(22.2)	35(77.8)	45	13
	Self-Employee	9(18)	41(82)	50	14.5
	Driver	10(20.8)	38(79.2)	48	13.9
	Daily Labor	23(25.6)	67(74.4)	90	26.01
	Farmer	25(45.5)	30(55.5)	55	15.9

Clinical characteristics of study participants

The clinical picture of study participants revealed that 150 (43.4%) and 140(40.5%) had a history of previous hospitalization and catheterization, of which 35(30.4%) and 42(30%) were culture positive for community acquired respectively. About 121(35%) of the respondents had diabetes at the time of the survey, with positive growth of 32(25.6%). Among the respondents, 105 (30.3%) were currently on their CD4 count was 201-350 cells/mm3 with 35(33.3%). Moreover, 210(60.7%), 200 (57.8%), 165 (47.7%), and 164 (47.4%) of participants showed dysuria, frequency of urination, flank pain, and abnormal urine color with positive growth of 58(27.6%), 55(27.5%), 61 (37%), and 64 (39%), respectively (**Table 2)**.

Table 2. Frequency of clinical characteristics of UTI symptomatic HIV positive patients attending Dessie Referral Hospital, Dessie, Ethiopia (February 1, 2021- march 30, 2021).

Variables	Category	Culture Result		Frequency	Percentage
		Positive No (%)	Negative No (%)		
History of hospitalization	Yes	35(30.4)	115(69.6)	150	43.4
	No	57(29.1)	139(70.9)	196	56.6
Use of cotrimoxazole as prophylaxis	Yes	40(26)	114(74)	154	44.5
	No	54(29.1)	138(70.9)	192	55.5
Diabetes	Yes	32(25.6)	89(74.4)	121	35
	No	60(24.6)	184(75.4)	244	65
History of catheterization	Yes	42(30)	120(70)	140	40.5
	No	50(24.3)	136(75.7)	206	59.5
UTI symptoms					
Dysuria	Yes	58(27.6)	152(72.4)	210	60.7
	No	34(25)	102(75)	136	39.3
Frequency of urination	Yes	55(27.5)	155(72.5)	200	57.8
	No	37(25.3)	109(74.7)	146	42.2
Urine color	abnormal	64(39)	100(61)	164	47.4
	Normal	28(15.4)	154(84.6)	182	52.6
flank pain	Yes	61(37)	104(63)	165	47.7
	No	31(17.1)	157(82.9)	181	52.8
Current CD4+ count	≤200 cells/mm^3	24(32.9)	49(67.1)	73	21.1
	201-350 cells/mm3	35(33.3)	70(66.7)	105	30.3
	351-500 cells/mm3	20(23.8)	64(762)	84	24.3
	≥500 cells/mm3	13(15.3)	72(84.7)	85	24.6

Prevalence of community acquired UTI and isolated pathogens

The prevalence of community-acquired UTIs Out of two hundred twenty-seven (346) urine samples collected, significant bacterial and yeast growth were observed in 92 samples, giving an overall prevalence of 26.6% (92/346) of which 21.67% (75/346) were bacterial pathogens, while 4.9% (17/346) were fungal pathogens. Among 75 bacterial isolates, 21.33% (16/75) were in *E. coli*, the most frequent bacterium, followed by *K. pneumoniae*, *enterococcus species*, and *P. aeruginosa*, accounting for 14.67% (11/75), 13.33% (10/75), and 12% (9/75), respectively. The least number of bacterial isolates were *Enterobacter cloacae*, which accounted for 4 (5.33%). Gram-positive bacteria accounted for only 32% (24/75) of the bacterial isolates, of which

41.67% (10/24) of the isolates were *Enterococcus species*. Of 17 yeast isolates, 47.1% (8/17) were represented by *C. tropicalis* (**Fig.1**)

Antimicrobial susceptibility pattern of bacterial Uropathogens

Antimicrobial susceptibility patterns of the isolated gram negative and gram positive uropathogens were shown in Tables 3 and 4, respectively. 52.9%, 52.9%, and 58.8% of the total isolated gram negative uropathogens were susceptible to ceftriaxone, ciprofloxacin, and gentamicin, respectively, whereas 51%, 72.5%, 72.5%, 84.3%, 60.8% and 60.8% of the isolates were resistant to amikacin, ampicillin, amoxicillin-Clavulanic acid, trimethoprim-sulphsmethsoxazole, nitrofurantoin, and ceftazidime, Discretely, it was observed that 62.5% of Escherichia coli strains were sensitive to gentamycin. But, 87.5%, 87.5%, 81.3%, 75%, 62.5%. and 56 % of bacterial isolates were resistant to tetracycline, Trimethoprim-sulphsmethsoxazole, amoxicillin-Clavulanic acid, ampicillin, nitrofurantoin, and ceftazidime, respectively. Similarly, 80% of ampicillin, 90.9% of trimethoprim-sulphsmethsoxazole, 90.9% of tetracycline and 63.6% of nitrofurantoin, and 63.6% ceftazidime, 54.5% was resistance to K. *pneumoniae*. However, (54.5%) of gentamicin, 54.5% of both amikacin and ciprofloxacillin, were sensitive against to *K. pneumoniae* **(Table 3).**

Table 3. Antimicrobial susceptibility pattern of Gram-negative bacterial isolates to antimicrobial agents among Community acquired UTI HIV-positive patients at Dessie Referral Hospital, Dessie, Ethiopia (February 1, 2021- march 30, 2021).

Etiologic agents	Pattern	Antimicrobial agents N (%)										
		AMP	AMC	AK	CRO	CIP	GN	CAZ	TE	CTX	SXT	F
E. coli (16)	S	4(25)	3(18.7)	9(56.2	9(56.2	8(50)	10(62.5)	7(43.8)	2(12.5)	8(50)	2(12.5)	6(37.5)
	R	12(75)	13(81.3)	7(43.8)	7(43.8)	8(50)	6(37.5)	9(56.2)	14(87.5)	8(50)	14(87.5)	10(62.5
K. pneumoniae (11)	S	3(27.3)	4(36.4)	5(45.5)	6(54.5)	5(45.5)	6(54.5)	4(36.4)	1(9.1)	6(54.5)	1(9.1)	4(36.4)
	R	8(72.7	7(63.6)	6(54.5)	5(45.5)	6(54.5)	5(45.5)	7(63.6)	10(90.9)	5(45.5)	10(90.9)	7(63.6)
P. mirabilis (5)	S	1(20)	2(40)	3(60)	3(60)	3(60)	1(20)	2(40)	1(20)	3(60)	1(20)	2(40)
	R	4(80)	3(60)	2(40)	2(40)	2(40)	4(80)	3(60)	4(80)	2(40)	4(80)	3(60)
P. aeruginosa (9)	S	3(33.3)	2(22.2)	5(55.6)	5(55.6)	5(55.6)	6(66.7)	4(44.4)	1(11.1)	4(44.4)	2(22.2)	3(33.3)
	R	6(66.7)	7(87.8)	4(44.4)	4(44.4)	4(44.4)	3(33.3)	5(55.6)	8(88.9)	5(55.6)	7(87.8)	6(66.7)
Enterobacte	S	1(25)	1(25)	2(50)	2(50)	2(50)	3(75)	1(25)	1(25)	2(50)	1(25)	2(50)

r cloacae. (4)	R	3(75)	3(75)	2(50)	2(50)	2(50)	1(25)	3(75)	3(75)	2(50)	3(75)	2(500
Providencia spp (6)	S	2(33.3)	2(33.3)	3(50)	2(33.3)	4(66.7)	4(66.7)	2(33.3)	1(16.7)	3(50)	1(16.7)	3(50)
	R	4(66.7)	4(66.7)	3(50)	4(66.7)	2(33.3)	2(33.30	4(66.7)	5(83.3)	3(50)	5(83.3)	3(50)
Total (51)	S	14(27.5)	14(27.5)	25(49)	27(52.9)	27(52.9)	30(58.8)	20(39.2)	7(13.7)	26(51)	8(15.7)	20(39.2)
	R	37(72.5)	37(72.5)	26(51)	24(47.1)	24(47.1)	21(41.2)	31(60.8	44(86.3	25(49)	43(84.3)	31(60.80

S: sensitive; R: resistant; AMP: ampicillin; AMC: Amoxicillin-Clavulanic acid; CTX: cefotaxime; CAZ: Ceftazidime; F: Nitrofurantoin; CRO: ceftriaxone; CIP: ciprofloxacin; GN: gentamicin; AK: Amikacin; TE: tetracycline; SXT: Trimethoprim-sulphsmethsoxazole.

From the total isolated Gram-positive uropathogens, 66.7% of isolates were susceptible to clindamycin. Whereas, 87.5%, 83.3%, 75%, 58.3%, and 58.3% of Gram-positive isolates were resistant to tetracycline, trimethoprim-sulfamethoxazole, penicillin, chloramphenicol, and Nitrofurantoin, respectively. About 70% of clindamycin and 60% of ciprofloxacin were more effective against *Enterococcus species,* respectively. However, *Enterococcus species* showed more resistance to 90% for tetracycline, 80% for trimethoprim-sulphsmethsoxazole and 70% for nitrofurantoin. Likewise, *coagulase negative staphylococci* (CoNs) were resistant to most of the antimicrobials 87.5% for tetracycline, 87.5% for trimethoprim-sulphsmethsoxazole, and 75% for penicillin and chloramphenicol each **(Table 4).**

Table 4. Antimicrobial susceptibility pattern of Gram-positive bacterial isolates to antimicrobial agents among community acquired UTI **HIV-positive patients attending at Dessie Referral Hospital, Dessie, Ethiopia (February 1, 2021- March 30, 2021).**

Bacterial isolates	Number of Resistance Pathogens to Antimicrobial Agents No (%)										
		E<br>N (%)	P<br>N (%)	C<br>N (%)	CXT<br>N (%)	F	CL<br>N (%)	CIP<br>N (%)	SXT<br>N (%)	FOX<br>N (%)	TE<br>N (%)
S. *aureus* (6)	S	4(66.7)	1(16.7)	3(50)	3(50)	2(33.3**)**	4(66.7)	4(66.7)	1(16.7)	3(50)	1(16.7)
	R	2(33.3)	5(83.3)	3(50)	3(50)	4(66.7)	2(33.3)	2(33.3)	5(83.3)	3(50)	5(83.3)
CONS (8)	S	3(37.5)	2(25)	2(25)	3(37.5)	5(62.5)	5(62.5)	2(25)	1(12.5)	NT	1(12.5)
	R	5(62.5)	6(75)	6(75)	5(62.5)	3(37.5)	3(37.5)	6(75)	7(87.5)	NT	7(87.5)
Enterococcus species (10)	S	4(40)	3(30)	5(50)	6(60)	3(30)	7(70)	6(60)	2(20)	4(40)	1(10)
	R	6(60)	7(70)	5(50)	4(40)	7(70)	3(30)	4(40)	8(80)	6(60)	9(90)
Total (24)	S	11(45.83)	6(25)	10(41.7)	12(50)	10(41.7)	16(66.7)	12(50)	4(16.7)	7(43.75)	3(12.5)
	R	13(54.17)	18(75)	14(58.3)	12(50)	14(58.3)	8(33.3)	12(50)	20(83.3)	9(56.25)	21(87.5)

Key: CL = Clindamycin, **E** = Erythromycin, **C** = Chloramphenicol, **CIP** = Ciprofloxacin, **TE** = Tetracycline, **SXT** = Trimethoprim-Sulfamethoxazole, **CTX**= cefotaxime, **FOX=**Cefoxitin, **F:** nitrofurantoin**, P** =penicillin, **R** = Resistant, **OX=**oxacillin**, FOX=**Cefoxitin **S** = Sensitive, **NT** = Not tested

Multi-drug resistance patterns of the bacterial isolates

Overall, 70 (93.3%) bacterial isolates were resistant to at least one antimicrobial agent, and 63 (84%) isolates were resistant to ≥2 antimicrobials. About 11 (14.7%) isolates had resistance to five or more antimicrobials. The overall prevalence of MDR bacteria was 61(81.3%). About *13* (81.3%) of *E. coli*, 9(81.8%) of *K. pneumoniae*, *8*(80%) of *Enterococcus species, 7 (77.8%) of P. aeruginosa*, and *CoNs* 7(87.5%) were the most frequently exhibited MDR **(Table 5).**

Table 5. Multi-drug resistance patterns of uropathogens bacterial isolates among UTI symptomatic HIV-positive patients attending at Dessie Referral Hospital, Dessie, Ethiopia (February 1, 2021- March 30, 2021).

Isolated organisms	Total	MDR to antimicrobials						
		R0 N (%)	R1 N (%)	R2 N (%)	R3 N (%)	R4 N (%)	≥R5 N (%)	MDR N (%)
E. coli	16	1(6.3)	1(6.3)	1(6.3)	4(25)	7(43.7)	2(12.5)	13(81.3)
K. pneumoniae	11	1(9.1)	1(9.1)	-	4(36.4)	2(18.2)	3(27.3)	9(81.8)
S. aureus	6	1(16.7)	-	-	3(50)	2(33.3)	-	5(83.3)
P. mirabilis	5	0	1(20)	-	2(40)	1(20)	1(20)	4(80)
CoNs	8	1(12.5)	-	-	4(50)	2(25)	1(12.5)	7(87.5)
P. aeruginosa	9	0	1(11.1)	1(11.1)	3(33.3)	2(22.2)	2(22.2)	7(77.8)
Enterobacter Cloacae	4	0	1(25)	-	1(25)	2(50)	-	3(75)
Enterococcus species	10	1(10)	1(10)	-	4(40)	3(30)	1(10)	8(80)
Providencia species	6	0	1(16.7)	-	2(33.3)	2(33.3)	1(16.7)	5(83.3)
Total	75	5(6.7)	7(9.3)	2(2.7)	27(36)	23(30.7)	11(14.7)	61(81.3)

R0=No antibiotic resistance category, R1=Resistance to one antibiotic category, R2=Resistance to two antibiotics category, R3=Resistance to three antibiotics category, R4=Resistance to four antibiotics category, R5=Resistance to five antibiotics category, R6=Resistance to six antibiotics category, R7=Resistance to seven antibiotics category

Factors associated with significant bacteriuria

In the current study, bivariate analysis was performed on sociodemographic and facility characteristics such as sex, family size, residence, occupations, immunization status, education status, and current marital status, current CD4 count, history of hospitalizations, catheterizations, and status of diabetics; and signs and symptoms of urinary tract infections. Multivariate analysis was done by including variables that had a p-value of ≤0.25 in bivariate analysis. After adjustment for age, sex, occupations, educational status, current CD4+ count (cells/mm3), history of previous hospitalization and diabetes status, history of catheterizations, urgency of

urinary tract. Age >44year [AOR=1.56, 95%CI: 1.11-9.35, P=0.001], being female [AOR=3.29, 95%CI: 1.26-13.41, P=0.002], being daily labor [AOR=4.54, 95%CI: 1.41-21.71, P=0.003], being farmer [AOR=6.54, 95%CI: 1.21-23.51, P=0.004], participants unable to read and write [AOR=4.58, 95%CI: 1.21-13.58, P=0.001], HIV patients with CD4 count of ≤ 200 cells/mm^3 (AOR: 7.44; 95%CI: 1.64-27.21, P=0.001) and CD4 count of 201-350 cells/mm^3 (AOR: 4.55; 95%CI: 1.36-18.28, P=0.009), patients who had chronic diabetics (AOR: 4.80; 95%CI: 1.85-16.31), patients who had chronic diabetics (AOR: 4.80; 95%CI: 1.85-16.31, p=0.009), Patients having history of hospitalization (AOR: 6.27; 95%CI: 1.25-15.17, p=0.002) and patients who had urgency of urinations (AOR: 6.51; 95%CI: 1.75-36.31, p=0.0025) were statistically significant associated risk factors for community acquired urinary tract infections (Table 6).

Table 6. Bivariable and multivariable analysis for variables associated with bacteriuria among UTI symptomatic HIV-positive patients attending at Dessie Referral Hospital, Dessie, Ethiopia (February 1, 2021- march 30, 2021).

Characteristics		Urine Culture Result		COR (95% CI)	p-value	AOR (95% CI)	P-value
		Positive n (%)	Negative n (%)				
	18–24	10(18.9)	43(81.1)	1	1		
	25–34	21(24.7)	64(75.3)	0.65[0.25-5.295]	0.984		
	35–44	30(25.9)	86(74.1)	0.985[0.154-14.038]	0.838		
Age in years	>44	31(33.7)	61(66.3)	1.56[1.11-19.035]	0.012	1.56[1.11-9.35]	0.001
	Male	42(25.3)	124(74.7)	1	1	1	1
Sex	Female	50(27.8)	130(72.2)	1.79[1.1-3.655]	0.185	3.29[1.26- 13.41]	0.002
	Urban	62(28.8)	153(71.2)	1	1		
Residence	Rural	30(22.9)	101(77.1)	1.4[0.427-3.026]	0.89		
	House Wife	15(25.9)	43(74.1)	1	1		
	Civil Servant	10(22.2)	35(77.8)	0.95[0.41-5.34]	0.54		
	Self-Employee	9(18)	41(82)	1.25[0.36-13.54]	0.36		
	Driver	10(20.8)	38(79.2)	0.71[0.15-16.12]	0.65		
	Daily Labor	23(25.6)	67(74.4)	3.842[1.37-17.91]	0.17	4.54[1.41-21.71]	0.003
Occupation	Farmer	25(45.5)	30(55.5)	4.842[1.17-17.91]	0.18	6.54[1.21-23.51]	0.004
	Illiterate	42(36.5)	73(63.5)	3.85[1.76-12.61]	0.114	4.58[1.21-13.85]	0.001
	Primary	20(23.3)	66(76.7)	0.98 [0.928-12.398]	0.265		
	Secondary	22(24.2)	69(75.8)	1.95[0.43-12.22]	0.655		
Educational status	College/University	8(14.8)	46(85.2)	1	1	1	1
	Married	45(37.2)	76(62.8)	1.89[0.61-16.86]	0.43		
	Unmarried	18(22.5)	62(77.5)	1.369[0.325-5.766]	0.668		
	Divorced	13(13.7)	82(86.3)	1.05[0.153 -7.270]	0.956		
Current marital status	Widowed	16(32)	34(68)	1	1		
Current CD4+ count (cells/mm^3)	≤200	24(32.9)	49(67.1)	5.29[1.39-18.51]	**0.018**	7.44[1.64-27.21]	**0.001**
	201-350	35(33.3)	70(66.7)	4.18[1.55-17.10]	**0.024**	4.55[1.36-18.28]	**0.009**

	351-500	20(23.8)	64(762)	0.98[0.57-12.56]	0.705	0.99[0.89-12.24]	0.861
	≥501	13(15.3)	72(84.7)	1	1	1	1
History of hospitalization	Yes	35(30.4)	115(69.6)	1.98[1.78- 13.28]	0.181	6.27[1.25- 15.17]	0.002
	No	57(29.1)	139(70.9)	1	1	1	1
Use of cotrimoxazole as prophylaxis	Yes	40(26)	114(74)	1	1		
	No	54(29.1)	138(70.9)	0.953[0.455-1.995]	0.898		
Diabetes	Yes	32((25.6)	89(74.4)	3.78[1.22-15.42]	0.016	4.80[1.85- 16.31]	0.009
	No	60(24.6)	184(75.4)	1	1	1	1
History of catheterization	Yes	42(30)	120(70)	1.794[1.53-16.03]	0.048	4.65[1.55- 26.31]	0.0012
	No	50(24.3)	136(75.7)	1	1		
Dysuria	Yes	58(27.6)	152(72.4)	0.753[0.368-1.979]	0.711		
	No	34(25.4)	100(74.6)	1	1		
Frequency of urination	Yes	55(27.5)	155(72.5)	0.629[0.395-1.741]	0.621		
	No	37(25.3)	109(74.7)	1	1		
Urgency	Yes	64(39)	100(61)	1.427[1.89-22.55]	0.039	6.51[1.75- 36.31]	0.0025
	No	28(15.4)	154(84.6)	1	1		
flank pain	Yes	61(37)	104(63)	1.193[0.548-2.599]	0.657		
	No	31(17.1)	157(82.9)	1	1		

Discussion

A urinary tract infection (UTI) is defined as the invasion of the urinary tract by one or more uro-pathogenic bacteria species, which results in significant bacteriuria and the presence of symptoms such as dysuria (21). According to demographic data, it is more common in women. Women are predisposed to UTIs due to anatomical and physical factors such as a shorter urethra. Surgery, pregnancy, advanced age, diabetes mellitus, kidney stones, immune system impairment, neurogenic bladder, and urinary tract abnormalities may all increase the risk of developing an UTI (22). In this study, the prevalence of significant bacteriuria was 26.6% [95% CI: 21.5% - 31.2%]. The current study is comparable with study done in Hawassa (23.1%)(23), Uganda (22.0%)(24). However, this finding was relatively higher than studies done in Jimma (13.7%) (25), Gojjam Ethiopia (20.6%)(26) , Hawassa (4.9%) (27), in Addis Ababa, Ethiopia (18.6%) and 11.3% (28, 29) and Harar, Ethiopia (18%) (30). But, it is lower than studies conducted in Nigeria (32.5%) (31), South Africa (48.7%) (32), Nepal (54.76%) and India (100%) and (49.15%) (33, 34). This variation might be due to differences in sample size, the degree of the

immune status of the study participants, geographical variation, ART status and socioeconomic conditions.

Candidauria is the presence of Candida spp. in the urine, such as C. albicans. Candidauria is classified as asymptomatic (in healthy people or patients) or symptomatic. Patients with cystitis, epididymorchitis, prostatis, pyelonephritis, and renal candidiasis experience symptomatic candidiasis (35). Asymptomatic Candidauria, on the other hand, is usually harmless and is not considered a disease. C. albicans is one of the most common fungi that can cause Candidauria (20% of nosocomial infections) (36). In the current study, the prevalence of community-acquired UTIs Out of two hundred twenty-seven (346) urine samples collected, significant yeast growth was observed in 4.9% (17/346) were fungal pathogens. Of the 17 yeast isolates, 47.1% (8/17) were represented by *C. tropicalis*. *Study also supported* C. albicans, C. glabrata, and C. tropicalis have been reported as the three most common species among young and adult individuals (37). Multiple studies showed that within hospital settings at least 10–15% of hospital-acquired UTIs are caused by Candida species and Candidauria is especially common in intensive care units. In study conducted in Addis Ababa, Candidauria represented 7% UTIs and 94% were hospital-acquired in which 62.5% of them were observed in ICU patients. C. albicans was the leading cause of Candidauria and 81.1% candida species were isolated from ward-acquired positive urine culture and ICU acquired positive urine culture samples (10) and study done in two microorganisms were Candida spp. (Candida glabrata and Candida krusei) (38).

Among 75 bacterial isolates, 21.33% (16/75) were in *E. coli*, the most frequent bacterium, followed by *K. pneumoniae*, *enterococcus species*, and *P. aeruginosa*, accounting for 14.67% (11/75), 13.33% (10/75), and 12% (9/75), respectively. Similarly finding was showed that *E. coli* was the predominant on those studies in Bahirdar Ethiopia (38.1%)(30), Gondar, Ethiopia (56.1%)(39) , Jimma, Ethiopia (54.3%) (25), and Tertiary Care Hospital, India (41.7%)(29), Saudi Arabia (40) and Ethiopia (27). However, it was inconsistent with the findings reported in Ebony State, Nigeria (41) and India (33), they found that *S. aureus* (45.3%) and *P. aeruginosa* (41.9%) and in Turkey the most common causative agent was *E. coli* (66.6% of cases) followed by *K. pneumoniae* (16.6%) and others such as *Enterobacter spp*. (7.7%) (38)were the commonest urinary tract pathogens. This discrepancy might be due to differences in test facility, strain variety, geographical variation, or study group variations. The presence of a

unique structure that aids bacteria attachment to uroepithelial cells, allowing for multiplication and tissue invasion, could explain the predominance of *E. coli* (42, 43).

Even though not statistically significant association was observed in this study, we found that the bacteriuria was high among females (27.8%) than males (25.3%). This finding is in agreement with various studies conducted in Ethiopia (28, 30, 39), South Africa (32), Nigeria (41). This could be explained by physiological and anatomical differences between males and females, such as males' shorter urethra, a decrease in normal vaginal flora, and females have less acidic vaginal surface pH (28).

In the current study, about 52.9%, 52.9%, and 58.8% of the total isolated gram negative uropathogens were susceptible to ceftriaxone, ciprofloxacin, and gentamicin, respectively, whereas, 51%, 72.5%, 72.3%, 84.3%, 60.8%, and 60.8% of the isolates were resistant to amikacin, ampicillin, amoxicillin-Clavulanic acid, and trimethoprim. The other study on urinary tract bacterial and fungal isolates supported these current findings with Frequency Percentage 11 sulphsmethsoxazole, nitrofurantoin, and ceftazidime and Most of the urinary bacterial isolates were highly sensitive to ceftazidime (95%) and ciprofloxacin (88% (44). In this study 87.5%, 87.5%, 81.3%, 75%, 62.5%. and 56 % of Escherichia coli strains were resistant to tetracycline, Trimethoprim-sulphsmethsoxazole, amoxicillin-Clavulanic acid, ampicillin, nitrofurantoin, and ceftazidime, respectively. Similarly, 80% of ampicillin, 90.9% of trimethoprim-sulphsmethsoxazole, 90.9% of tetracycline and 63.6% of nitrofurantoin, and 63.6% ceftazidime, 54.5% was resistance to K. pneumoniae.

In this study, 87.5%, 87.5%, 81.3%, 75%, 62.5%, and 56 % of Escherichia coli strains were resistant to tetracycline, Trimethoprim-sulphsmethsoxazole, amoxicillin-Clavulanic acid, ampicillin, nitrofurantoin, and ceftazidime, respectively. Similarly, 80% of ampicillin, 90.9% of trimethoprim-sulphsmethsoxazole, 90.9% of tetracycline, 63.6% of nitrofurantoin, and 63.6% of ceftazidime, 54.5% was resistance to K. pneumoniae. There were also similar findings from studies conducted Addis Ababa In K. pneumoniae, the highest level of resistance was observed against sulfamethoxazole trimethoprim (86.4%) cefotaxime (86.4%), cefepime (85.4%), ceftazidime (85.4%), amoxacillin-clavulinic acid (85.4%), gentamicin (70.0%), and ciprofloxacin (50.5% (45), In Iran: sulfamethoxazole-trimethoprim (91.4%), ceftazidime (91.4%), and gentamicin (82.8%) (46) ; in Sierra Leone: ciprofloxacin (73.4%) and gentamicin (60%) (47), in Equatorial Guinea: sulfamethoxazole-trimethoprim (100%), amoxacillin-

clavulinic acid (96.6%), gentamicin (86.2%), and ciprofloxacin (87.5% (48). The high antibiotic resistance of Enterobacteriaceae may be due to antibiotic misuse and overuse, combined with inadequate infection control measures (49) This was comparable with the study done in Gondar, Ethiopia (39) and Harar, Ethiopia (30), *P. aeruginosa* was 100% resistant to ampicillin, cefotaxime, and gentamicin and trimethoprim-sulphsmethsoxazole *P.mirabilis* 100% resistant to ampicillin. This is in agreement with a study done in Ethiopia (28).

Similarly, about 87.5%, 83.3%, 75%, 58.3%, and 58.3% of gram-positive isolates were resistant to tetracycline, trimethoprim-sulfamethoxazole, penicillin, chloramphenicol, and Nitrofurantoin, respectively. The most isolated gram-positive *Enterococcus species* showed more resistance to 90% of tetracycline, 80% of trimethoprim-sulphsmethsoxazole and 70% of nitrofurantoin. Likewise, coagulase negative staphylococci (CoNs) were resistant to most of the antimicrobials; 87.5% for tetracycline, 87.5% for trimethoprim-sulphsmethsoxazole, and 75% for penicillin and chloramphenicol each. This is in line with a study done in Harar, Ethiopia, where 57.1% of *S. aureus* were resistant to gentamicin, tetracycline, and cefoxitin (57.1%) (30). We also found that 85% of *Coagulase-negative staphylococcus spp*. were resistant to Trimethoprim-sulphamethaxazole (85%) and over 60% were resistant to penicillin, gentamicin, and tetracycline.

Multidrug resistance has serious implications on the health outcome of HIV-infected patients (50). It is quite alarming to note that almost was 61(81.3%) of currently isolated bacteria were found to be resistant to three or more antimicrobials. This was higher compared to previous findings reported in Harar, Ethiopia (46%) (30), Dessie (74.6%) (51), Gondar (68%) (52) and Nepal (64.04%)(53) and India (28%) (54) . But it was lower than a report from Gondar, Ethiopia (95%) and 87.4%) (3, 39), Addis Ababa (100%) (28), in Bahirdar (93.1%) (55), in Nepal (96.84%) (53). These differences might be due to the irrational drug utilization habit of the communities or to the over the distribution of those sensitive and resistant strains of bacteria.

There was an intra-species difference in MDR level. The present study showed that the level of MDR in E. coli 13 (81.3%), K. pneumoniae 9(81.8%), Enterococcus species 8(80%), P. aeruginosa 7 (77.8%) and CoNs 7(87.5%). This was comparable with a study conducted in Addis Ababa K. pneumoniae (83.5%) and E. coli (66.2%) (56), Equatorial Guinea in E. coli (74.4%) (48); in Sierra Leone K. pneumoniae (73.3%) and E. coli (61.5%) (47); in Gondar K. pneumoniae was (95.6%) and in E. coli was (92.9%) (3); in Khartoum-Sudan E. coli

(92.2%)(57); and in Equatorial Guinea in K. pneumoniae (91.7%) (48). The MDR level among E. coli (50.2%) in Dessie is lower than our study(51). The difference in MDR level among K. pneumoniae and E. coli in our study might be due to the fact that most K. pneumoniae were isolated from blood specimens that were collected from inpatients in the hospitals.

In this study, there was no statistically significant association between significant bacteriuria and in patients' residence, marital status, dysuria, frequency of urination, flank pain, and use of cotrimoxazole as prophylaxis However, age, gender, occupation, educational status, current CD4+ count (cells/mm3), previous hospitalization and diabetes status, history of catheterizations, and urinary tract urgency had a statistically significant association with significant bacteriuria. Similar findings were reported from Addis Jimma Ababa and Harar (25, 28, 30). Furthermore, Individuals age >44year [AOR=1.56, 95%CI: 1.11-9.35, P=0.001], being female [AOR=3.29, 95%CI: 1.26- 13.41, P=0.002], being daily labor [AOR=4.54, 95%CI: 1.41-21.71, P=0.003], being farmer [AOR=6.54, 95%CI: 1.21-23.51, P=0.004], participants unable to read and write [AOR=4.58, 95%CI: 1.21-13.58, P=0.001], HIV patients with CD4 count of ≤ 200 cells/mm3 (AOR: 7.44; 95%CI: 1.64-27.21, P=0.001) and CD4 count of 201-350 cells/mm3 (AOR: 4.55; 95%CI: 1.36- 18.28, P=0.009), patients who had chronic diabetics (AOR: 4.80; 95%CI: 1.85-16.31), patients who had chronic diabetics (AOR: 4.80; 95%CI: 1.85-16.31, p=0.009), Patients having history of hospitalization (AOR: 6.27; 95%CI: 1.25-15.17, p=0.002) and patients who had urgency of urinations (AOR: 6.51; 95%CI: 1.75-36.31, p=0.0025). This finding was similar to studies conducted elsewhere [26, 22, 27, and 29]. The explanation for the inverse relationship of UTI and a CD4+ count is unknown; it is probably due to the impaired immunity at a declining CD4+count that makes it easier for bacterial pathogens to adhere to the urinary epithelium.

Limitation of the study

However, the study was a single hospital-based study and might not represent all HIV infected patients and did not include control group due to budget constraints. We did not attempt to identify other causative agents (anaerobic bacteria, and fungus) that would have made a significant contribution to a true prevalence of UTI in HIV-positive patients.

Conclusions and Recommendations

The asymptomatic community's acquired UTI is high, with an overall prevalence of 26.6% (92/346). From the isolated pathogens, 21.67% (75/346) were bacterial pathogens, while 4.9% (17/346) were fungal pathogens. Among bacterial pathogens, *E. coli*, the most frequent bacterium, followed by *K. pneumoniae*, *enterococcus species*, and *P. aeruginosa*, accounting for, respectively, the overall prevalence of MDR bacteria was 61(81.3%). About 13 (81.3%) of E. coli, 9(81.8%) of *K. pneumoniae*, 8(80%) of *Enterococcus species*, 7 (77.8%) of *P. aeruginosa*, and *CoNs* 7(87.5%). Most Gram-negative and Gram-positive isolates were resistant to tetracycline, trimethoprim-sulfamethoxazole, penicillin, chloramphenicol, and Nitrofurantoin, respectively. However, uropathogens were susceptible to amikacin, it was more effective against these pathogens. Patients with low CD4 counts were more at risk of developing bacterial urinary tract infections, previous hospitalization and diabetes status, and having a history of catheterizations than their counterparts. Management of UTI among symptomatic HIV patients should be supported by laboratory results of urine culture. Regular monitoring of antimicrobial resistance patterns should be undertaken by healthcare providers. Individuals at risk for bacterial associated UTI should have good adherence to ART and their CD4 levels should be monitored on a regular basis. Health information dissemination should be given about UTI and the habit of drug use for HIV positive patients.

List of abbreviation and acronyms

AIDS; Acquired immune deficiency syndrome, ARTC; Attending antiretroviral therapy clinic, ART; Antiretroviral therapy, ATCC; American Type Culture collection, Ba; Blood agar, CFU; Colony Forming Unit, CLED; Cysteine Lactose Electrolyte Deficient, CONS; Coagulase negative Staphylococci, HAART; Highly active antiretroviral therapy, HIV; Human Immunodeficiency Virus, MACA; MacConkey agar, MDR; Multi-Drug Resistant, MSU; Midstream urine, OIS; Opportunistic infections, UTI; Urinary Tract Infection, VCT Voluntary counseling and Testing.

Data availability statement

Data supporting the conclusions of this article are within the manuscript and is available on reasonable request from the principal investigators due to ethical reasons.

Funding

No external funds were obtained

Competing interests

The authors declare that they have no competing interest.

Authors’ contributions

Conceived and designed the experiments: Mihret Tilahun, Chernet Belayhun, Abdurrahman Seid, Bekele Sharew, Tewodros Dessie, Habtamu Mekonen ; Performed the experiments: Mihret Tilahun, Abdurrahman Seid, Agumas Shibabaw; Analyzed the data: Mihret Tilahun, Abdurrahman Seid, Daniel G/tsadik, Mesfin Fiseha, Melkam Tesfaye, Alemu Gedefie, Melaku Ashagrie Belete; Wrote and edited the manuscript: Mihret Tilahun, Abdurrahman Seid, Bekele Sharew, Alemu Gedefie, Habtu Debash, Agumas Shibabaw, Melaku Ashagrie Belete and Habtamu Mekonnen. All authors read and approved the final manuscript.

Acknowledgement

The authors would like to acknowledge Department of Medical Laboratory Science, College of Medicine and Health Sciences, Wollo University for providing the laboratory set up and facilities to conduct the experiments. Dessie comprehensive specialized hospital and all study participants are gratefully acknowledged for their kind cooperation during data collection.

References

1. Çoban B, Ülkü N, Kaplan H, Topal B, Erdoğan H, Baskın E. Five-year assessment of causative agents and antibiotic resistances in urinary tract infections. Turkish Archives of Pediatrics/Türk Pediatri Arşivi. 2014;49(2):124.
2. Flores-Mireles AL, Walker JN, Caparon M, Hultgren SJ. Urinary tract infections: epidemiology, mechanisms of infection and treatment options. Nature reviews microbiology. 2015;13(5):269-84.
3. Eshetie S, Unakal C, Gelaw A, Ayelign B, Endris M, Moges F. Multidrug resistant and carbapenemase producing Enterobacteriaceae among patients with urinary tract infection at referral Hospital, Northwest Ethiopia. Antimicrobial resistance and infection control. 2015;4(1):1-8.
4. Ahmed SS, Shariq A, Alsalloom AA, Babikir IH, Alhomoud BN. Uropathogens and their antimicrobial resistance patterns: Relationship with urinary tract infections. International Journal of Health Sciences. 2019;13(2):48.
5. Rao P, Chennu M, Vennila T, Kosanam S, Ponsudha P, Suriyakrishnaan K, et al. Assessment of Bacterial Isolates from the Urine Specimens of Urinary Tract Infected Patient. BioMed Research International. 2022;2022.
6. Paschke AA, Zaoutis T, Conway PH, Xie D, Keren R. Previous antimicrobial exposure is associated with drug-resistant urinary tract infections in children. Pediatrics. 2010;125(4):664-72.
7. Burton RJ, Albur M, Eberl M, Cuff SM. Using artificial intelligence to reduce diagnostic workload without compromising detection of urinary tract infections. BMC medical informatics and decision making. 2019;19(1):1-11.
8. Frank-Peterside N, Okerentugba P, Nwodo C, Okonko I. Prevalence of bacterial uropathogens in a cohort of HIV-positive males in Port Harcourt, Nigeria. Cancer Biology. 2013;3(4):12-7.
9. Birhanu MY, Habtegiorgis SD, Gietaneh W, Alemu S, Tsegaye TB, Bekele GM, et al. Magnitude and associated factors of urinary tract infections among adults living with HIV in Ethiopia. Systematic review and meta-analysis. PloS one. 2022;17(4):e0264732.
10. Bitew A, Zena N, Abdeta A. Bacterial and fungal profile, antibiotic susceptibility patterns of bacterial pathogens and associated risk factors of urinary tract infection among symptomatic pediatrics patients attending St. Paul's Hospital Millennium Medical College: a cross-sectional study. Infection and drug resistance. 2022;15:1613.
11. Foxman B. Urinary tract infection syndromes: occurrence, recurrence, bacteriology, risk factors, and disease burden. Infectious Disease Clinics. 2014;28(1):1-13.
12. Islam MA, Islam MR, Khan R, Amin MB, Rahman M, Hossain MI, et al. Prevalence, etiology and antibiotic resistance patterns of community-acquired urinary tract infections in Dhaka, Bangladesh. Plos one. 2022;17(9):e0274423.

13. Haider MZ, Annamaraju P. Bladder Catheterization. StatPearls [Internet]: StatPearls Publishing; 2022.
14. Magiorakos A-P, Srinivasan A, Carey RB, Carmeli Y, Falagas M, Giske C, et al. Multidrug-resistant, extensively drug-resistant and pandrug-resistant bacteria: an international expert proposal for interim standard definitions for acquired resistance. Clinical microbiology and infection. 2012;18(3):268-81.
15. Bigwan E, Wakjissa F. Prevalence of urinary tract infections among HIV patients attending a non-governmental health facility in Jos, Plateau State, Nigeria. International Journal of Biomedical and Advance Research. 2013;4(8):528-33.
16. Murray PR, Rosenthal KS, Pfaller MA. Medical microbiology E-book: Elsevier Health Sciences; 2020.
17. Pezzlo M. Laboratory diagnosis of urinary tract infections: guidelines, challenges, and innovations. Clinical Microbiology Newsletter. 2014;36(12):87-93.
18. Bullock NO, Aslanzadeh J. Biochemical profile-based microbial identification systems. Advanced techniques in diagnostic microbiology: Springer; 2013. p. 87-121.
19. Malik UM, Khan AB, Satti ML. Comparative evaluation of CHROMagar and API 20C AUX in isolation and identification of Candida species. Journal of Islamic International Medical College (JIIMC). 2018;13(2):85-90.
20. Institute CLS. CLSI Performance standard for antimicrobial susceptibility testing, M100. 2021.
21. Behzadi P, Urbán E, Matuz M, Benkő R, Gajdács M. The role of gram-negative bacteria in urinary tract infections: current concepts and therapeutic options. Advances in Microbiology, Infectious Diseases and Public Health. 2020:35-69.
22. Fatima SS, Mussaed EA. Urinary tract infection. Bacterial Identification and Drug Susceptibility Patterns in Pregnant and Non Pregnant UTI Patients: Springer; 2018. p. 1-22.
23. Nigussie D, Amsalu A. Prevalence of uropathogen and their antibiotic resistance pattern among diabetic patients. Turkish Journal of Urology. 2017;43(1):85.
24. Nabaigwa BI, Mwambi B, Okiria J, Oyet C. Common uropathogens among diabetic patients with urinary tract infection at Jinja Regional Referral Hospital, Uganda. African Journal of Laboratory Medicine. 2018;7(1):1-3.
25. Debalke S, Cheneke W, Tassew H, Awol M. Urinary tract infection among antiretroviral therapy users and nonusers in Jimma University Specialized Hospital, Jimma, Ethiopia. International journal of microbiology. 2014;2014.
26. Girma A, Aemiro A. The Bacterial Profile and Antimicrobial Susceptibility Patterns of Urinary Tract Infection Patients at Pawe General Hospital, Northwest Ethiopia. Scientifica. 2022;2022.
27. Tessema NN, Ali MM, Zenebe MH. Bacterial associated urinary tract infection, risk factors, and drug susceptibility profile among adult people living with HIV at Haswassa University Comprehensive Specialized Hospital, Hawassa, Southern Esthiopia. Scientific Reports. 2020;10(1):1-9.

28. Getu Y, Ali I, Lema T, Belay H, Yeshetela B. Bacteriuria and antimicrobial susceptibility pattern among HIV patients attending ALERT Center, Addis Ababa, Ethiopia. Am J Health Res. 2017;5(3):76-82.
29. Fenta G, Legese M, Weldearegay G. Bacteriuria and their antibiotic susceptibility patterns among people living with HIV attending Tikur Anbessa Specialized and Zewditu Memorial Hospital ART Clinics, Addis Ababa, Ethiopia. J Bacteriol Parasitol. 2016;7(05).
30. Marami D, Balakrishnan S, Seyoum B. Prevalence, Antimicrobial Susceptibility Pattern of Bacterial Isolates, and Associated Factors of Urinary Tract Infections among HIV- Positive Patients at Hiwot Fana Specialized University Hospital, Eastern Ethiopia. Canadian Journal of Infectious Diseases and Medical Microbiology. 2019;2019.
31. Okechukwu A, Thairu Y. Bacteria urinary tract infection in HIV-infected children and adolescents in Abuja, Nigeria: a cross-sectional study. African Journal of Clinical and Experimental Microbiology. 2019;20(4):306-14.
32. Iweriebor B, Obi C, Akinyemi O, Ramalivhana N, Hattori T, Okoh A. Uropathogens isolated from HIV-infected patients from Limpopo Province, South Africa. African Journal of Biotechnology. 2012;11(46):10598-604.
33. Xavier TF, Auxilia A, Kannan M. Isolation and characterization of UTI pathogens from HIV positive patients of Karur District, Tamil Nadu, India. Int J Curr Microbiol Appl Sci. 2015;4(1):558-63.
34. Sharma V, Gupta V, Mittal M. Prevalence of uropathogens in diabetic patients and their antimicrobial susceptibility pattern. Natl J lab Med. 2012;1(1):26-8.
35. Odabasi Z, Mert A. Candida urinary tract infections in adults. World journal of urology. 2020;38(11):2699-707.
36. Behzadi P, Behzadi E, Ranjbar R. Urinary tract infections and Candida albicans. Central European journal of urology. 2015;68(1):96-101.
37. Achkar JM, Fries BC. Candida infections of the genitourinary tract. Clinical microbiology reviews. 2010;23(2):253-73.
38. Erdem I, Ali RK, Ardic E, Omar SE, Mutlu R, Topkaya AE. Community-acquired lower urinary tract infections: Etiology, antimicrobial resistance, and treatment results in female patients. Journal of global infectious diseases. 2018;10(3):129.
39. Alemu A, Dagnew M, Alem M, Gizachew M. Uropathogenic bacterial isolates and their antimicrobial susceptibility patterns among HIV/AIDS patients attending Gondar University Specialized Hospital Gondar, Northwest Ethiopia. 2013.
40. Biradar SK, Doddamani P. Prevalence and antibiogram of uropathogens in a tertiary care hospital. World J Pharmaceut Res. 2013;2:1534-43.

41. Ifeanyichukwu I, Emmanuel N, Chika E, Anthonia O, Esther U-I, Ngozi A, et al. Frequency and antibiogram of uropathogens isolated from urine samples of HIV infected patients on antiretroviral therapy. American Journal of BioScience. 2013;1(3):50-3.
42. Ejerssa AW, Gadisa DA, Orjino TA. Prevalence of bacterial uropathogens and their antimicrobial susceptibility patterns among pregnant women in Eastern Ethiopia: hospital-based cross-sectional study. BMC women's health. 2021;21(1):1-15.
43. Fierer J, Looney D, Kok M, Pechère J-C. Nature and pathogenicity of micro-organisms. Infectious diseases. 2010:3.
44. Nwadioha S, Nwokedi E, Ikeh I, Egesie J, Kashibu E. Antibiotic susceptibility pattern of uropathogenic bacterial isolates from AIDS patients in a Nigerian tertiary hospital. J Med Med Sci. 2010;1(11):530-4.
45. Desta MK. College of Health Sciences School of Allied Health Sciences Department of Medical Laboratory Sciences: Addis Ababa University; 2017.
46. Mansouri S, Abbasi S. Prevalence of multiple drug resistant clinical isolates of extended-spectrum beta-lactamase producing Enterobacteriaceae in Southeast Iran. 2010.
47. Leski TA, Taitt CR, Bangura U, Stockelman MG, Ansumana R, Cooper WH, et al. High prevalence of multidrug resistant Enterobacteriaceae isolated from outpatient urine samples but not the hospital environment in Bo, Sierra Leone. BMC infectious diseases. 2016;16(1):1-9.
48. Shatalov A. Prevalence and antibiotic resistance pattern of Escherichia coli and Klebsiella pneumoniae in urine tract infections at the La Paz Medical center, Malabo, Equatorial Guinea. Open Journal of Medical Microbiology. 2015;5(04):177.
49. Adler A, Katz DE, Marchaim D. The continuing plague of extended-spectrum β-lactamase–producing Enterobacteriaceae infections. Infectious Disease Clinics. 2016;30(2):347-75.
50. Kim H-R, Hwang SS, Kim HJ, Lee SM, Yoo C-G, Kim YW, et al. Impact of extensive drug resistance on treatment outcomes in non-HIV-infected patients with multidrug-resistant tuberculosis. Clinical Infectious Diseases. 2007;45(10):1290-5.
51. Kibret M, Abera B. Antimicrobial susceptibility patterns of E. coli from clinical sources in northeast Ethiopia. African health sciences. 2011;11:40-5.
52. Moges F, Mengistu G, Genetu A. Multiple drug resistance in Urinary pathogens at Gondar College of Medical science hospital, Ethiopia. East African medical journal. 2002;79(8):415-20.
53. Yadav KK, Adhikari N, Khadka R, Pant AD, Shah B. Multidrug resistant Enterobacteriaceae and extended spectrum β-lactamase producing Escherichia coli: a cross-sectional study in National Kidney Center, Nepal. Antimicrobial resistance and infection control. 2015;4(1):1-7.
54. Murugesh K, Deepa S, Ravindranath C, Venkatesha D. Multi drug resistant uropathogens in HIV: Are they a threat to community? International Journal of Scientific Study. 2014;2(3):38-42.

55. Biadglegne F, Abera B. Antimicrobial resistance of bacterial isolates from urinary tract infections at Felge Hiwot Referral Hospital, Ethiopia. The Ethiopian Journal of Health Development. 2009;23(3).
56. Teklu DS, Negeri AA, Legese MH, Bedada TL, Woldemariam HK, Tullu KD. Extended-spectrum beta-lactamase production and multi-drug resistance among Enterobacteriaceae isolated in Addis Ababa, Ethiopia. Antimicrobial Resistance & Infection Control. 2019;8(1):1-12.
57. Ibrahim M, Bilal N, Hamid M. Increased multi-drug resistant Escherichia coli from hospitals in Khartoum state, Sudan. African health sciences. 2012;12(3):368-75.

Figure legend

Figure 1. Frequency of uropathogens isolates among Community Acquired UTI HIV-seropositive patients attending at Dessie Referral Hospital, Dessie, Ethiopia From February 1, 2021 to march 30, 2021.

Click here to access/download;Manuscript;Revised manuscript .docx

Uropathogens, multidrug resistance pattern of bacterial and associated factors of community-acquired urinary tract infection among HIV positive patients attending Antiretroviral Therapy Clinics of Northeast Ethiopia.

Mihret Tilahun[1*], Mesfin Fiseha[1], Mihreteab Alebachew[1], Alemu Gedefie[1], Endris Ebrahim[1], Melkam Tesfaye[1], Melaku Ashagrie Belete[1], Abdurahaman Seid [1], Daniel Gebretsadik [1], Ermiyas Alemayehu[1], Wondmagegn Demsiss[1], Bekele Sharew[1], Agumas Shibabaw[1], Habtamu Mekonnen[2] Tewodros Dessie [2]

[1]Department of Medical Laboratory Sciences, College of Medicine and Health Science, Wollo University, Dessie, Ethiopia

[2] Amhara Public Health Institute Dessie branch

Authors' information:

Mihret Tilahun (MSc in Medical Microbiology): tilahunmihret21@gmail.com

Mesfin Fiseha (MSc in hematology and immunohematology): mesfinfiseha40@gmail.com

Alemu Gedefie (MSc in Medical Microbiology): alemugedefie@gmail.com

Mihreteab Alebachew (MSc in medical parasitology and vector control): mihreteab71@gmail.com

Endris Ebrahim (MSc in immunology): endris.index@gmail.com

Melkam Tesfaye (PhD fellow in infectious diseases: Melkam66@gmail.com

Melaku Ashagrie Belete (MSc in Medical Microbiology): melakuashagrie@gmail.com

Abdurahaman Seid (MSc., Asst Prof in Medical Microbiology): gechdk2000@yahoo.com

Daniel Gebretsadik (MSc., Asst Prof in Tropical and infectious diseases): gebretsadikd@gmail.com

Ermiyas Alemayehu ((MSc in clinical chemistry): ermiyas0009@gmail.com

Wondmagegn Demsiss (MSc., Asst Prof in Medical Microbiology): *wonde1980@gmail.com*

Bekele Sharew PhD, Asst Professor in Medical Microbiology): bsharew@gmail.com

Agumas Shibabaw (PhD, Asst Professor in Medical Microbiology): agumas2000@gmail.com

Habtamu Mekonnen (MSc in Medical Microbiology: habtamumeko36@gmial.com

Tewodros Dessie (MSc in Medical Microbiology: tedydess21@gmial.com

Corresponding author

Mihret Tilahun (MSc.)

Department of Medical Laboratory Science

College of Medicine and Health Sciences,
Wollo University, Dessie, Ethiopia
Telephone: +251- 920988307
Fax: +251 333115250
PO.BOX: 1145
Email: tilahunmihret21@gmail.com

Abstract

Background: Urinary tract infections are one of the most common bacterial and fungal infections in humans, occurring both in the community and in health-care settings. It is a significant health issue among HIV-infected patients. Urinary tract infection causes by drug resistance pathogen is now widespread and poses a serious clinical risk. This study investigated bacterial and fungal profile, multidrug resistance profile of bacterial pathogens and associated Factors of Community-acquired urinary tract infection among HIV-positive patients attending Antiretroviral Therapy Clinics in Northeast Ethiopia from February 1, 2021 to March 30, 2021.

Methods: Institutional based cross-sectional study was conducted at Dessie Referral Hospital. Socio-demographical and clinical data were collected using structured questionnaires from community-acquired urinary tract infection suspected HIV-Positive patients. About 10 ml Clean-catch midstream urine was collected and inoculated into Blood agar, MacConkey and Cysteine lactose electrolyte deficient media. Gram stain and biochemical tests were performed to identify isolates and antimicrobial susceptibility pattern was performed on disc diffusion techniques. Data were entered and analyzed using SPSS version-25. Descriptive statistics such as tables and bar graphs were used to summarize the findings. Both binary and multivariable logistic regression analysis was performed and a P value of < 0.05 with an adjusted odds ratio with their 95% confidence interval (CI) was used as statistically significant associations.

Results: Among the total 346 study participants, 92 (26.6%) were culture positive. About 75 (21.67%) were bacterial and 17 (4.9%) were fungal pathogens. From a total of 75 bacteria isolates 51(68%) were Gram-negative bacteria. The most commonly isolated Gram-negative bacteria were *E. coli* 16 (21.33%) followed by *K. pneumoniae* 11(14.67%). From the 17 fungal isolate of fungi 8(47.1%) were represented *by C. tropicalis.* The overall prevalence of Multi-Drug Resistance bacteria was 61(81.3%). About 13 (81.3%) of *E. coli*, 9(81.8%) of *K. pneumoniae*, 8(80%) of *Enterococcus* species, 7 (77.8%) of *P. aeruginosa*, and *CoNs* 7(87.5%) were the most frequently exhibited Multi-Drug Resistance. Amikacin, and gentamicin were effective against Gram-negative uropathogens. participants with age >44year, being female, being daily labor, being farmer, unable to read and write, patients with CD4 count of ≤ 200 cells/mm^3 and CD4 count of 201-350 cells/mm^3, who had chronic diabetics, patients having a

history of hospitalization and who had urgency of urinations were statistically significant association with significant bacteriuria.

Conclusion

There are high uropathogens and multi-drug resistance pathogens in symptomatic patients. The most dominant bacterial isolates were *E. coli* followed by *K. pneumoniae* and *S. aureus*. Amikacin and gentamicin were effective against Gram-negative uropathogens. Management of UTI among symptomatic HIV patients should be supported by laboratory result of urine culture. Good adherence to ART and their CD4 levels should be monitored regularly. Health information dissemination should be given about UTIs and the habit of drug use for HIV-positive patients Regular culture test of antimicrobial susceptibility patterns should be performed.

Keywords: Uropathogens, community-acquired UTI, Multidrug resistance, Ethiopia

Introduction

Urinary tract infection (UTI) is an infection of the urinary tract, including the kidneys, ureters and bladder, urethra, and accessory structures that collect and store and release urine from the body. It occurs when microorganisms, typically bacteria from the digestive tract, cling to the urethral opening and begin to multiply(1-3).

Globally, the burden of UTI is estimated to be 150 million cases every year (4). Based on previous epidemiological studies from Tanzania and elsewhere in sub-Saharan Africa, urinary tract infections were estimated at 35–45% (5). In Ethiopia the pooled prevalence of urinary tract infection was 15.97%(6) and Urinary tract infections in HIV patients in Ethiopia were 12.8% (7).

Urinary tract infections (UTIs) are caused by gram-negative and gram-positive bacteria, as well as certain fungi. Uro-pathogenic *Escherichia coli (E. coli)* is the most common cause of both uncomplicated and complicated UTIs. *Escherichia coli* is the most common agent involved in uncomplicated UTIs, followed by *Klebsiella pneumoniae(K.pneumoniae)*, *Staphylococcus saprophyticus (S.Saprophyticus)*, *Enterococcus faecalis (E. faecalis)*, *group B streptococcus(*GBS*)*, *Proteus mirabilis (P.mirabilis)*, *Pseudomonas aeruginosa* (*P. aeruginosa)*, *Staphylococcus aureus (S.aureus)*, and *Candida* spp (8). Following uro-pathogenic *Escherichia coli* as the most common causative agent for complicated UTIs, the order of prevalence for causative agents is *Enterococcus* spp., *K. pneumoniae*, *Candida* spp., *S. aureus*, *P. mirabilis*, *P. aeruginosa*, and GBS (9).

Candidauria is the presence of *Candida spp.* in the urine, such as *C. albicans*. Candidauria is classified as asymptomatic (in healthy people or patients) or symptomatic. Patients with cystitis, epididymorchitis, prostatis, pyelonephritis, and renal candidiasis experience symptomatic candidiasis (10). On the other hand, a symptomatic Candidauria is usually harmless and is not considered a disease. C. albicans is one of the most common fungi that can cause Candidauria (20% of nosocomial infections) (11).

Globally, particularly in developing countries, the emergence of antibiotic resistance in the treatment of urinary tract infections is a major public health issue (12). Recently, the most common pathogens of UTIs have developed resistance to most antimicrobial agents (13). The three most commonly isolated bacteria *E. coli*, *Klebsiella spp.*, and *Proteus spp.* had high resistance rates to amoxicillin and tetracycline of 80.1%–90.0% and low sensitivity rates of 0–25% to nitrofurantoin, ciprofloxacin, and gentamicin. Many research studies in the world and

Africa, including different parts of Ethiopia, showed that more than 50% of uro-pathogenic bacteria developed multidrug resistance (MDR). About 70.0% of bacteria from India (14), 79.3% from Wolayta Sodo Ethiopia (15), 78.4% from Addis Ababa (16) and 91.7% from Gondar (17) developed MDR.

In Ethiopia, The antimicrobial test analysis showed that 152 (47.85%) of the isolates were resistant to two or more antimicrobials (18). Resistance to these antimicrobial agents in UTI cases will thus have an impact on future treatment and management of the infection with these drugs. Since the majority of treatments are initiated empirically, knowledge of the organisms, their epidemiological characteristics, adequate treatment, control of these conditions necessitate and antibacterial susceptibility is required (19).

Urinary tract infections cause increased mortality, disability, hospitalization, economic loss in the HIV population, renal failure, and hypertension. It is prone to nephritic complications such as urinary tract obstruction, which causes renal colic, pelvic-ureteric junction obstruction, benign prostate hyperplasia, urethral strictures, bladder dysfunction, and urinary stasis (20).

A systematic review indicated that being female, having a history of recurrent UTI, having low socioeconomic status, having diabetes mellitus, genitourinary tract abnormalities, having a low CD4 count, history of catheterization, history of hospitalization and chronic diabetics status were independent factors for the occurrence of urinary tract infection (7, 15). An-other study on Factors associated with urinary tract infections among HIV-1 infected patients also showed that low CD4 count, chronic hepatitis, and being female have been linked to the occurrence of bacterial UTI (21).

Despite numerous studies on bacterial UTI, antimicrobial susceptibility testing, and HIV infection-related factors in developed countries, there is little data on the burden of bacterial UTI and antimicrobial susceptibility patterns among UTI-symptomatic HIV-infected patients in resource-limited settings, including Ethiopia in general, and no study was done in the study area in particular. As a result, the purpose of this study was to determine the bacterial and fungal profiles, multidrug resistance profiles of bacterial pathogens, and associated factors for community-acquired urinary tract infection among HIV-positive patients attending Antiretroviral Therapy Clinics in Northeast Ethiopia.

Methods and materials

Study design, area and period

A health facility-based cross-sectional study was conducted at a comprehensive specialized hospital in Dessie town from February 1, 2021 to March 30, 2021. According to the Dessie town health administrative office, in the town, there are also 16 governmental health institutions (1 comprehensive specialized hospital, 1 general hospital, 8 health centers) and 3 private general hospitals, 6 higher private clinics, and the hospital serves about 12 million people living in the catchment areas. The Dessie comprehensive specialized hospital serves about seven million people living in the catchment areas and has a 600 average daily patient flow also has 800 staff, among whom about 600 are health professionals. It has 600 beds, 8 wards, and different OPDs like adult, pediatric, emergency, and TB & HIV. Also, the hospital provides different services like laboratory, pharmacy, imaging (X-ray, ultra-sound and city scan), and a comprehensive ART service for about 9,500 people.

Population

All HIV-positive individuals living in Dessie town and nearby catchments were source the of populations. All HIV- patients with symptomatic to community acquired UTI who visited the ART clinic of a comprehensive specialized hospital during the study period before 48 hours of admissions were target populations.

Eligibility criteria of study participants

The HIV-positive adults who were ≥18 years old and complaining of UTI symptoms suggestive of UTI including; lower abdominal or flank pain, dysuria and hematuria, urgency, frequency, hesitance, and consented to be part of the study. Patients who were mentally handicapped were not included in the study because they were unable to consent

Variables

Dependent variables included the prevalence of bacterial and fungal community-acquired UTI and the antimicrobial susceptibility pattern of uro-pathogenic bacteria. While age, sex, marital status, educational level, residence, history of catheterization in the past year, Current ART status, symptoms of UTI (dysuria, urgency, frequency, flank pain, and urine color), diabetes, CD4+cell count, cotrimoxazole usage, and history of hospitalization in the past year were independent variables.

Sample size and sampling technique

The required sample size was determined by using single population proportion formula considering the following assumptions: Prevalence = 28.6% (prevalence of culture proven UTI among HIV seropositive and UTI symptomatic patients from previous study conducted in Ethiopia (22) . Sample size,

$$n = \frac{z^2 * P\,(1 - P)}{d^2}$$

Where: n= the required sample size Z = Z score for 95 % confidence interval = 1.96, p = prevalence=28.6% (0.286), d = tolerable error =5 %, 1-p=Q then, 0.714, n = $((1.96)^2$ x 0.286x (1-0.286))/ $(0.05)^2$**=314. With 10% non-response rate 32.** However, during the study period, a total of 346 UTI symptomatic HIV-patients and fulfilling the inclusion criteria were included consecutively.

Operational definitions

UTI: Urinary tract infections are one of the most common infections in humans that can happen anywhere along the urinary tract and it is caused by microorganisms (microscopic organisms that are too small to be seen by the naked eye). There are several different types of microorganisms which include bacteria, protozoa, fungi and algae (23, 24).

Community-acquired urinary tract infection (CA-UTI); Community-acquired UTI is an infection if an individual with UTI is suspected before hospital admission and specimens are collected from the outpatient or within less than 48 hours of hospital admission (25).

History of catheterization: the use of or insertion of a catheter into the bladder or participants who have catheterized before to data collections (26).

History of hospitalization: participants who were taken to a health facility and kept there for 48 hrs for treatment before to data collections in the past six months or more months.

Multidrug resistance: A bacterium that is simultaneously resistant to three or more antimicrobial categories (27).

Data and specimen collection

A pretested structured questionnaire was used to collect data on sociodemographic characteristics and associated factors. The participants' recent CD4+ count was retrieved from their medical records. About 10 mL of voided clean-catch midstream fresh urine was collected from each participant by using a leak-proof and sterile wide-mouthed screw-capped container. These specimens were labeled and stored in a cold box (4°C) (28) and before being transported to the Wollo University College of Medicine and Health Sciences microbiology laboratory for analysis.

Isolation and identification of bacteria

Bacterial isolation and phenotypic characterizations were performed using the recommended culture and biochemical tests (29). A calibrated loop that delivers 0.001 mL of urine was used to inoculate each urine sample on to the Cysteine lactose electrolyte deficient agar (Oxoid Ltd, UK). The plates were incubated aerobically at 37°C. After overnight incubation, Cysteine lactose electrolyte-deficient agar plates with a growth of $\geq 10^4$-10^5CFU/mL (colony-forming units per milliliter) were considered significant. Gram stain was performed and sub cultured on to MacConkey agar (HiMediaTM) and 5% blood agar plates (HiMediaTM). However, bacteria that did not show growth after 24 hrs of incubation were incubated again for the next 24 hrs and discarded as negative and the colony count was not significant (30). From plates with significant bacteria growth, the phenotypic characterization of bacteria was carried out using colony characteristics, gram reactions, and a series of biochemical reactions, including catalase, coagulase, oxidase, urease, indole, citrate utilization, lysine decarboxylase, glucose, lactose fermentation, gas and H2S production, and motility tests (31).

Yeast Identification

Yeasts were identified using routine diagnostic methods such as Gram stain, Germ Tube Test, Carbohydrate Fermentation, and Assimilation Tests, as well as chromogenic medium (CHROMagar Candida Medium (Biomerieux, France) according to the manufacturer's instructions (32).

Antimicrobial susceptibility testing

An antimicrobial susceptibility test was performed using a Kirby–Bauer disk diffusion method based on the Clinical and Laboratory Standards Institute (CLSI) recommendation (33). Three to five pure colonies of a similar appearance to each isolate were emulsified in 4 ml of sterile normal saline. The inoculum suspension was standardized by comparing the turbidity with 0.5 McFarland

standards. A Muller-Hinton agar plate (HiMediaTM) was streaked with the prepared inoculum suspension using a sterile swab, and antibiotic discs were placed on each agar plate after 5 minutes using sterile forceps. The antimicrobials (Oxoid Ltd) that were used for bacterial susceptibility testing were, amoxicillin-clavulanic acid (AMC) 10µg, ampicillin (AMP) 10µg, amikacin (Amk) 30µg, cefotaxime (CTX) 30µg, ceftriaxone (CRO) 30µg, trimethoprim-sulphamethoxazole (SXT) 25µg, ciprofloxacin (CIP) 5µg, gentamycin (Gen) 10 µg, ceftazidime (CAZ) 30µg, nitrofurantoin (F) 300µg, tetracycline (TE) 30µg and penicillin (Pen) 10µg (33). Within 15 minutes after the application of the discs, the plates were incubated at 35°c for 18 hours and the plates were examined for growth. The diameters of zone of inhibition (in mm) were measured by using a graduated caliper in millimeters and results interpreted as sensitive (S), intermediate (I), or resistance (R) (33).

Data quality management

The questionnaire was initially prepared in English, and translated into the local language (Amharic) by language experts back to English by other experts to assure its consistency and accuracy. The questionnaire was pretested on 5% of HIV-positive patients at Dessie health center, Dessie, Ethiopia. The quality of culture media was tested for sterility and performance. The sterility of culture media was checked by incubating overnight at 37 ºC without specimen inoculation. Any physical change like cracks, excess moisture, color, hemolysis, dehydration, and contamination was assessed & the expiration date was also checked. The new batch culture medium and antimicrobial disks were checked for performance and quality using the American Type Culture Collection (ATCC) reference strains such as *E. coli* (ATCC® 25922), *S. aureus* (ATCC® 25923), *Klebsiella pneumonia* (ATCC 700603) and *P. aeruginosa* (ATCC® 27853).

Statistical analysis

The data generated was entered every day into epi-data version 4.6.0.4. The data was then exported and analyzed using the Social Sciences Statistical Package (SPSS) version 25. The frequency and percentage descriptive statistics were calculated and presented using graphs and tables. A bivariable analysis was conducted to classify factors that separately affect the frequency of dependent variables. Variables with a P-value less than or equal to 0.25 in the bivariable analysis were subjected to multi-variable analysis. adjusted odd ratio with a p-value of <0.05 and 95% CI taken as statistically significant. Finally, the results were presented in words, graphs and tables.

Ethical considerations

Ethical clearance and permissions were obtained from the ethical review committee of Wollo University College of Medicine and Health Sciences with protocol number CMHS 365/2021 and a support letter was written to Dessie comprehensive specialized Hospital. Permission was obtained from Dessie comprehensive specialized hospitals. Written assent was also obtained from family and surrogates to obtain information from those who can't give data. Information obtained from the study participants was kept confidential. Positive results were communicated and given to attending physicians for appropriate treatment.

Results

Socio-demographic characteristics

A total of 346 HIV-positive patients were included in this study. The age of the study participants ranged from 21 to 80 years, with a mean (±SD) age of 39.44 (±10.87) years, and 116 (33.5%) of them were in the age range of 35–44 years. About 180 (52.02%) of the respondents were females. Regarding educational status, 115 (33.2%) of the respondents had primary level education. Concerning occupational status, 90 (26.01%) were self-employed. Concerning residences, 215 (62.1%) of them were urban dwellers, as shown in **(Table 1).**

Table 1. Sociodemographic characteristics of UTI symptomatic HIV positive patients attending at Dessie referral hospital, Dessie, Ethiopia.

Variable	Category	Urinary tract infection		Frequency	Percentage
		Positive No (%)	Negative No (%)		
Sex	Male	42(25.3)	124(74.7)	166	(47.98)
	Female	50(27.8)	130(72.2)	180	(52.02)
Residence	Rural	62(28.8)	153(71.2)	215	62.1
	Urban	30(22.9)	101(77.1)	131	37.9
Age (in years)	18–24	10(18.9)	43(81.1)	53	15.3
	25–34	21(24.7)	64(75.3)	85	24.6)
	35–44	30(25.9)	86(74.1)	116	33.5
	>44	31(33.7)	61(66.3)	92	26.6
Educational status	Unable to read and write	42(36.5)	73(63.5)	115	33.2
	Primary	20(23.3)	66(76.7)	86	24.9
	Secondary	22(24.2)	69(75.8)	91	26.3

	College/University	8(14.8)	46(85.2)	54	15.6
Marital status	Married	45(37.2)	76(62.8)	121	35
	Unmarried	18(22.5)	62(77.5)	80	23.1
	Divorced	13(13.7)	82(86.3)	95	27.5
	Widowed	16(32)	34(68)	50	14.5
Occupation	House Wife	15(25.9)	43(74.1)	58	16.8
	Civil Servant	10(22.2)	35(77.8)	45	13
	Self-Employee	9(18)	41(82)	50	14.5
	Driver	10(20.8)	38(79.2)	48	13.9
	Daily Labor	23(25.6)	67(74.4)	90	26.01
	Farmer	25(45.5)	30(55.5)	55	15.9

Clinical characteristics of study participants

The clinical picture of study participants revealed that 150 (43.4%) and 140(40.5%) had a history of previous hospitalization and catheterization, of which 35(30.4%) and 42(30%) were culture positive for community-acquired urinary tract infections, respectively. About 121(35%) of the respondents had diabetes at the time of the survey, with positive growth of 32(25.6%). About 105 (30.3%) HIV patients had 201-350 cells/mm3 CD4 count with positive growth of 35(33.3%). Moreover, 210(60.7%), 200 (57.8%), 165 (47.7%), and 164 (47.4%) participants showed dysuria, frequency of urination, flank pain, and abnormal urine color with positive growth of 58(27.6%), 55(27.5%), 61 (37%), and 64 (39%), respectively (**Table 2)**.

Table 2. Frequency of clinical characteristics of UTI symptomatic HIV positive patients attending Dessie Referral Hospital, Dessie, Ethiopia (February 1, 2021- march 30, 2021).

Variables	Category	Culture Result		Frequency	Percentage
		Positive No (%)	Negative No (%)		
History of hospitalization	Yes	35(30.4)	115(69.6)	150	43.4
	No	57(29.1)	139(70.9)	196	56.6
Use of cotrimoxazole as prophylaxis	Yes	40(26)	114(74)	154	44.5
	No	54(29.1)	138(70.9)	192	55.5
Diabetes	Yes	32(25.6)	89(74.4)	121	35
	No	60(24.6)	184(75.4)	244	65
History of catheterization	Yes	42(30)	120(70)	140	40.5
	No	50(24.3)	136(75.7)	206	59.5
UTI symptoms					
Dysuria	Yes	58(27.6)	152(72.4)	210	60.7
	No	34(25)	102(75)	136	39.3
Frequency of	Yes	55(27.5)	155(72.5)	200	57.8

urination	No	37(25.3)	109(74.7)	146	42.2
Urine color	abnormal	64(39)	100(61)	164	47.4
	Normal	28(15.4)	154(84.6)	182	52.6
Flank pain	Yes	61(37)	104(63)	165	47.7
	No	31(17.1)	157(82.9)	181	52.8
CD4+ count	≤200 cells/mm^3	24(32.9)	49(67.1)	73	21.1
	201-350 cells/mm3	35(33.3)	70(66.7)	105	30.3
	351-500 cells/mm3	20(23.8)	64(762)	84	24.3
	≥500 cells/mm3	13(15.3)	72(84.7)	85	24.6

Prevalence of community acquired UTI and isolated pathogens

The prevalence of community-acquired UTIs Out of three hundred Forty-six (346) urine samples collected, significant bacteriuria and yeast growth were observed in 92 samples, giving an overall prevalence of 26.6% (92/346) of which 21.67% (75/346) were bacteria and 4.9% (17/346) were fungal candida. Among 75 bacterial isolates, the most frequent bacterium was E. *coli* which accounted for 21.33% (16/75) followed by *K. pneumoniae*, *enterococcus species*, and *P. aeruginosa*, accounting for 14.67% (11/75), 13.33% (10/75), and 12% (9/75), respectively. The least number of bacterial isolates were *Enterobacter cloacae*, which accounted for 4 (5.33%). Gram-positive bacteria accounted for only 32% (24/75) of the bacterial isolates, of which 41.67% (10/24) of the isolates were *Enterococcus species*. Of 17 candida isolates, 47.1% (8/17) were represented by *C. tropicalis* (**Fig.1)**

Antimicrobial susceptibility pattern of bacterial Uropathogens

Gram Negative bacteria

Antimicrobial susceptibility patterns of the isolated Gram-negative and Gram-positive uropathogens. About 52.9%, 52.9%, and 58.8% of the total isolated gram-negative uropathogens were susceptible to ceftriaxone, ciprofloxacin, and gentamicin, respectively, whereas 51%, 72.5%, 72.5%, 84.3%, 60.8% and 60.8% of the isolates were resistant to amikacin, ampicillin, amoxicillin-clavulanic acid, trimethoprim-sulphsmethsoxazole, nitrofurantoin, and ceftazidime, respectively. About 62.5% *E. coli* strains of were sensitive to gentamycin. But, 87.5%, 87.5%, 81.3%, 75%, 62.5% and 56 % of bacterial isolates were resistant to tetracycline, Trimethoprim-sulphamethoxazole, amoxicillin-Clavulanic acid, ampicillin, nitrofurantoin, and ceftazidime, respectively. Similarly, 80% of ampicillin, 90.9% of trimethoprim-sulphamethoxazole, 90.9% of tetracycline and 63.6% of nitrofurantoin, and 63.6% ceftazidime, 54.5% was resistance to K. *pneumoniae*. However, (54.5%) of

gentamicin, and 54.5% of both amikacin and ciprofloxacillin, were sensitive against *K. pneumoniae* **(Table 3).**

Table 3. Antimicrobial susceptibility pattern of Gram-negative bacterial isolates among Community acquired UTI HIV-positive patients at Dessie Referral Hospital, Dessie, Ethiopia

Etiologic agents	Pattern	Antimicrobial agents N (%)										
		AMP	AMC	AK	CRO	CIP	GN	CAZ	TE	CTX	SXT	F
E. coli (16)	S	4(25)	3(18.7)	9(56.2	9(56.2	8(50)	10(62.5)	7(43.8)	2(12.5)	8(50)	2(12.5)	6(37.5)
	R	12(75)	13(81.3)	7(43.8)	7(43.8)	8(50)	6(37.5)	9(56.2)	14(87.5)	8(50)	14(87.5)	10(62.5
K. pneumoniae (11)	S	3(27.3)	4(36.4)	5(45.5)	6(54.5)	5(45.5)	6(54.5)	4(36.4)	1(9.1)	6(54.5)	1(9.1)	4(36.4)
	R	8(72.7	7(63.6)	6(54.5)	5(45.5)	6(54.5)	5(45.5)	7(63.6)	10(90.9)	5(45.5)	10(90.9)	7(63.6)
P. mirabilis (5)	S	1(20)	2(40)	3(60)	3(60)	3(60)	1(20)	2(40)	1(20)	3(60)	1(20)	2(40)
	R	4(80)	3(60)	2(40)	2(40)	2(40)	4(80)	3(60)	4(80)	2(40)	4(80)	3(60)
P. aeruginosa (9)	S	3(33.3)	2(22.2)	5(55.6)	5(55.6)	5(55.6)	6(66.7)	4(44.4)	1(11.1)	4(44.4)	2(22.2)	3(33.3)
	R	6(66.7)	7(87.8)	4(44.4)	4(44.4)	4(44.4)	3(33.3)	5(55.6)	8(88.9)	5(55.6)	7(87.8)	6(66.7)
Enterobacter cloacae. (4)	S	1(25)	1(25)	2(50)	2(50)	2(50)	3(75)	1(25)	1(25)	2(50)	1(25)	2(50)
	R	3(75)	3(75)	2(50)	2(50)	2(50)	1(25)	3(75)	3(75)	2(50)	3(75)	2(500
Providencia spp (6)	S	2(33.3)	2(33.3)	3(50)	2(33.3)	4(66.7)	4(66.7)	2(33.3)	1(16.7)	3(50)	1(16.7)	3(50)
	R	4(66.7)	4(66.7)	3(50)	4(66.7)	2(33.3)	2(33.30	4(66.7)	5(83.3)	3(50)	5(83.3)	3(50)
Total (51)	S	14(27.5)	14(27.5)	25(49)	27(52.9)	27(52.9)	30(58.8)	20(39.2)	7(13.7)	26(51)	8(15.7)	20(39.2)
	R	37(72.5)	37(72.5)	26(51)	24(47.1)	24(47.1)	21(41.2)	31(60.8	44(86.3	25(49)	43(84.3)	31(60.80

S: sensitive; R: resistant; AMP: ampicillin; AMC: Amoxicillin-Clavulanic acid; CTX: cefotaxime; CAZ: Ceftazidime; F: Nitrofurantoin; CRO: ceftriaxone; CIP: ciprofloxacin; GN: gentamicin; AK: Amikacin; TE: tetracycline; SXT: Trimethoprim-sulphsmethsoxazole.

Gram Positive bacteria

From the total isolated Gram-positive uropathogens, 66.7% of isolates were susceptible to clindamycin. Whereas, 87.5%, 83.3%, 75%, 58.3%, and 58.3% of Gram-positive isolates were resistant to tetracycline, trimethoprim-sulfamethoxazole, penicillin, chloramphenicol, and nitrofurantoin, respectively. About 70% of clindamycin and 60% of ciprofloxacin were more effective against *Enterococcus species*, respectively. However, *Enterococcus species* showed more resistance to 90% for tetracycline, 80% for trimethoprim-sulphsmethsoxazole and 70% for nitrofurantoin. Likewise, *coagulase-negative staphylococci* (CoNs)

were resistant to most of the antimicrobials 87.5% for tetracycline, 87.5% for trimethoprim-sulphsmethsoxazole, and 75% for penicillin and chloramphenicol each **(Table 4).**

Table 4. Antimicrobial susceptibility pattern of Gram-positive bacterial isolates among community-acquired UTI HIV-positive patients attending at Dessie Referral Hospital, Dessie, Ethiopia

Bacterial isolates	Antimicrobial Agents N (%)										
		E<br>N (%)	P<br>N (%)	C<br>N (%)	CXT<br>N (%)	F	CL<br>N (%)	CIP<br>N (%)	SXT<br>N (%)	FOX<br>N (%)	TE<br>N (%)
S. *aureus* (6)	S	4(66.7)	1(16.7)	3(50)	3(50)	2(33.3)	4(66.7)	4(66.7)	1(16.7)	3(50)	1(16.7)
	R	2(33.3)	5(83.3)	3(50)	3(50)	4(66.7)	2(33.3)	2(33.3)	5(83.3)	3(50)	5(83.3)
CoNS (8)	S	3(37.5)	2(25)	2(25)	3(37.5)	5(62.5)	5(62.5)	2(25)	1(12.5)	NT	1(12.5)
	R	5(62.5)	6(75)	6(75)	5(62.5)	3(37.5)	3(37.5)	6(75)	7(87.5)	NT	7(87.5)
Enterococcus species (10)	S	4(40)	3(30)	5(50)	6(60)	3(30)	7(70)	6(60)	2(20)	4(40)	1(10)
	R	6(60)	7(70)	5(50)	4(40)	7(70)	3(30)	4(40)	8(80)	6(60)	9(90)
Total (24)	S	11(45.83)	6(25)	10(41.7)	12(50)	10(41.7)	16(66.7)	12(50)	4(16.7)	7(43.75)	3(12.5)
	R	13(54.17)	18(75)	14(58.3)	12(50)	14(58.3)	8(33.3)	12(50)	20(83.3)	9(56.25)	21(87.5)

Key: **CL** = Clindamycin, **E** = Erythromycin, **C** = Chloramphenicol, **CIP** = Ciprofloxacin, **TE** = Tetracycline, **SXT** = Trimethoprim-Sulfamethoxazole, **CTX**= cefotaxime, **FOX**=Cefoxitin, **F:** nitrofurantoin**,** **P** =penicillin, **R** = Resistant, **OX**=oxacillin**,** **FOX**=Cefoxitin **S** = Sensitive, **NT** = Not tested

Multi-drug resistance patterns of the bacterial isolates

Overall, 70 (93.3%) bacterial isolates were resistant to at least one antimicrobial agent, and 63 (84%) isolates were resistant to ≥2 antimicrobials. About 11 (14.7%) isolates had resistance to five or more antimicrobials. The overall prevalence of MDR bacteria (a bacterium that is simultaneously resistant to three or more antimicrobial categories) was 61(81.3%). About *13* (81.3%) of *E. coli*, 9(81.8%) of *K. pneumoniae*, *8*(80%) of *Enterococcus species*, *7 (77.8%) of P. aeruginosa*, and *CoNs* 7(87.5%) were the most frequently exhibited MDR **(Table 5).**

Table 5. Multi-drug resistance patterns of uropathogens bacterial isolates among UTI symptomatic HIV-positive patients attending at Dessie Referral Hospital, Dessie, Ethiopia (February 1, 2021- March 30, 2021).

Isolated organisms	Total	MDR to antimicrobials						
		R0 N (%)	R1 N (%)	R2 N (%)	R3 N (%)	R4 N (%)	≥R5 N (%)	MDR N (%)
E. coli	16	1(6.3)	1(6.3)	1(6.3)	4(25)	7(43.7)	2(12.5)	13(81.3)
K. pneumoniae	11	1(9.1)	1(9.1)	-	4(36.4)	2(18.2)	3(27.3)	9(81.8)
S. aureus	6	1(16.7)	-	-	3(50)	2(33.3)	-	5(83.3)
P. mirabilis	5	0	1(20)	-	2(40)	1(20)	1(20)	4(80)
CoNs	8	1(12.5)	-	-	4(50)	2(25)	1(12.5)	7(87.5)
P. aeruginosa	9	0	1(11.1)	1(11.1)	3(33.3)	2(22.2)	2(22.2)	7(77.8)
Enterobacter Cloacae	4	0	1(25)	-	1(25)	2(50)	-	3(75)
Enterococcus species	10	1(10)	1(10)	-	4(40)	3(30)	1(10)	8(80)
Providencia species	6	0	1(16.7)	-	2(33.3)	2(33.3)	1(16.7)	5(83.3)
Total	75	5(6.7)	7(9.3)	2(2.7)	27(36)	23(30.7)	11(14.7)	61(81.3)

R0=No antibiotic resistance category, R1=Resistance to one antibiotic category, R2=Resistance to two antibiotics category, R3=Resistance to three antibiotics category, R4=Resistance to four antibiotics category, R5=Resistance to five antibiotics category

Factors associated with significant bacteriuria

In the current study, bivariate analysis was performed on sociodemographic characteristics such as sex, family size, residence, occupations, immunization status, education status, current marital status, current CD4 count, history of hospitalizations, catheterizations, and status of diabetics; and signs and symptoms of urinary tract infections. Multivariate analysis was done by including variables that had a p-value of ≤0.25 in bivariate analysis. After adjustment for age, sex, occupation, educational status, current CD4+ count (cells/mm3), history of previous hospitalization and diabetes status, history of catheterizations, and urgency of the urinary

tract. Age >44year [AOR=1.56, 95%CI: 1.11-9.35, P=0.001], being female [AOR=3.29, 95%CI: 1.26-13.41, P=0.002], being daily labor [AOR=4.54, 95%CI: 1.41-21.71, P=0.003], being farmer [AOR=6.54, 95%CI: 1.21-23.51, P=0.004], participants unable to read and write [AOR=4.58, 95%CI: 1.21-13.58, P=0.001], HIV patients with CD4 count of ≤ 200 cells/mm^3 (AOR: 7.44; 95%CI: 1.64-27.21, P=0.001) and CD4 count of 201-350 cells/mm^3 (AOR: 4.55; 95%CI: 1.36-18.28, P=0.009), patients who had chronic diabetics (AOR: 4.80; 95%CI: 1.85-16.31, p=0.009), patients who had chronic diabetics (AOR: 4.80; 95%CI: 1.85-16.31, p=0.009), patients having a history of hospitalization (AOR: 6.27; 95%CI: 1.25-15.17, p=0.002) and patients who had the urgency of urinations (AOR: 6.51; 95%CI: 1.75-36.31, p=0.0025) were statistically significant associated risk factors for community acquired urinary tract infections (Table 6).

Table 6. Bivariable and multivariable analysis for variables associated with bacteriuria among UTI symptomatic HIV-positive patients attending at Dessie Referral Hospital, Dessie, Ethiopia (February 1, 2021- march 30, 2021).

Characteristics		Urine Culture Result		COR (95% CI)	p-value	AOR (95% CI)	P-value
		Positive n (%)	Negative n (%)				
Age in years	18–24	10(18.9)	43(81.1)	1	1		
	25–34	21(24.7)	64(75.3)	0.65(0.25-5.295)	0.984		
	35–44	30(25.9)	86(74.1)	0.985(0.154-14.038)	0.838		
	>44	31(33.7)	61(66.3)	1.56 (1.11-19.035)	0.012	1.56(1.11-9.35)	0.001
Sex	Male	42(25.3)	124(74.7)	1	1	1	1
	Female	50(27.8)	130(72.2)	1.79(1.1-3.655)	0.185	3.29(1.26- 13.41)	0.002
Residence	Urban	62(28.8)	153(71.2)	1	1		
	Rural	30(22.9)	101(77.1)	1.4(0.427-3.026)	0.89		
Occupation	House Wife	15(25.9)	43(74.1)	1	1		
	Civil Servant	10(22.2)	35(77.8)	0.95(0.41-5.34)	0.54		
	Self-Employee	9(18)	41(82)	1.25(0.36-13.54)	0.36		
	Driver	10(20.8)	38(79.2)	0.71(0.15-16.12)	0.65		
	Daily Labor	23(25.6)	67(74.4)	3.842(1.37-17.91)	0.17	4.54(1.41-21.71)	0.003
	Farmer	25(45.5)	30(55.5)	4.842(1.17-17.91)	0.18	6.54(1.21-23.51)	0.004
Educational status	Unable to read and write	42(36.5)	73(63.5)	3.85(1.76-12.61)	0.114	4.58(1.21-13.85)	0.001
	Primary	20(23.3)	66(76.7)	0.98 (0.928-12.398)	0.265		
	Secondary	22(24.2)	69(75.8)	1.95(0.43-12.22)	0.655		
	College/University	8(14.8)	46(85.2)	1	1	1	1
Current marital status	Married	45(37.2)	76(62.8)	1.8([0.61-16.86)	0.43		
	Unmarried	18(22.5)	62(77.5)	1.369(0.325-5.766)	0.668		
	Divorced	13(13.7)	82(86.3)	1.05(0.153 -7.270)	0.956		
	Widowed	16(32)	34(68)	1	1		
Current CD4+ count (cells/mm^3)	≤200	24(32.9)	49(67.1)	5.29(1.39-18.51)	**0.018**	7.44(1.64-27.21)	**0.001**
	201-350	35(33.3)	70(66.7)	4.18(1.55-17.10)	**0.024**	4.55(1.36-18.28)	**0.009**

	351-500	20(23.8)	64(762)	0.98(0.57-12.56)	0.705	0.99(0.89-12.24)	0.861
	≥501	13(15.3)	72(84.7)	1	1	1	1
History of hospitalization	Yes	35(30.4)	115(69.6)	1.98(1.78- 13.28)	0.181	6.27(1.25- 15.17)	0.002
	No	57(29.1)	139(70.9)	1	1	1	1
Use of cotrimoxazole as prophylaxis	Yes	40(26)	114(74)	1	1		
	No	54(29.1)	138(70.9)	0.953(0.455-1.995)	0.898		
Chronic diabetes	Yes	32((25.6)	89(74.4)	3.78(1.22-15.42)	0.016	4.80(1.85- 16.31)	0.009
	No	60(24.6)	184(75.4)	1	1	1	1
History of catheterization	Yes	42(30)	120(70)	1.794(1.53-16.03)	0.048	4.65(1.55- 26.31)	0.0012
	No	50(24.3)	136(75.7)	1	1		
Dysuria	Yes	58(27.6)	152(72.4)	0.753(0.368-1.979)	0.711		
	No	34(25.4)	100(74.6)	1	1		
Frequency of urination	Yes	55(27.5)	155(72.5)	0.629(0.395-1.741)	0.621		
	No	37(25.3)	109(74.7)	1	1		
Urgency	Yes	64(39)	100(61)	1.427(1.89-22.55)	0.039	6.51(1.75- 36.31)	0.0025
	No	28(15.4)	154(84.6)	1	1		
Flank pain	Yes	61(37)	104(63)	1.193(0.548-2.599)	0.657		
	No	31(17.1)	157(82.9)	1	1		

Discussion

A urinary tract infection (UTI) is defined as the invasion of the urinary tract by one or more uro-pathogenic bacteria species, which results in significant bacteriuria and the presence of symptoms such as dysuria, pain and burning while urinating, cloudy urine, and urine appears red, bright pink or cola-colored (34). In this study, the prevalence of significant bacteriuria was 26.6% [95% CI: 21.5% -31.2%]. The current study is comparable with a study done in Hawassa (23.1%)(35) and Uganda (22.0%)(36). However, this finding was relatively higher than studies done in Jimma (13.7%) (37), Gojjam Ethiopia (20.6%)(38), Hawassa (4.9%) (39), Addis Ababa, Ethiopia (18.6%) and 11.3% (16, 40) and Harar, Ethiopia (18%) (41). But, it is lower than studies conducted in Nigeria (32.5%) (42), South Africa (48.7%) (43), Nepal (54.76%) and India (100%) and (49.15%) (44, 45). This variation might be due to differences in sample size, the degree of the immune status of the study participants, geographical variation, ART status and socioeconomic conditions.

In the current study, significant fungal growth was observed in 4.9% (17/346). Of the 17 yeast isolates, 47.1% (8/17) were represented by *C. tropicalis*. The study also supported C. albicans, C. glabrata, and C. tropicalis have been reported as the three most common species among young and adult individuals (46). Studies showed that within hospital settings at least 11.3 % of community-acquired UTIs are caused by Candida species and Candidauria (47). In study conducted in Addis Ababa, Candidauria represented 7% UTIs in which *C. albicans* was the leading cause of Candidauria and 81.1% candida species were isolated (22), and study done in two microorganisms were Candida spp. (Candida glabrata and Candida krusei) (48).

Among 75 bacterial isolates, 21.33% (16/75) were *E. coli*, the most frequent bacterium, followed by *K. pneumoniae*, *enterococcus species*, and *P. aeruginosa*, accounting for 14.67% (11/75), 13.33% (10/75), and 12% (9/75), respectively. Similarly, the finding showed that *E. coli* was the predominant in those studies in Bahirdar Ethiopia (38.1%)(41), Gondar, Ethiopia (56.1%)(49) , Jimma, Ethiopia (54.3%) (37), and Tertiary Care Hospital, India (41.7%)(16), Saudi Arabia (50) and Ethiopia (39). However, it was inconsistent with the findings reported in Ebony State, Nigeria (51) and India (44), they found that *S. aureus* (45.3%) and *P. aeruginosa* (41.9%), and in Turkey the most common causative agent was *E. coli* (66.6% of cases) followed by *K. pneumoniae* (16.6%) and others such as *Enterobacter spp*. (7.7%) (48)were the commonest urinary tract pathogens. This discrepancy might be due to differences in the test facility, strain variety, geographical variation, or study group variations. The presence of a unique structure that aids bacteria attachment to uroepithelial cells, allowing for multiplication and tissue invasion, could explain the predominance of *E. coli* (52, 53).

Even though not statistically significant association was observed in this study, we found that the bacteriuria was high among females (27.8%) than males (25.3%). This finding is in agreement with various studies conducted in Ethiopia (40, 41, 49), South Africa (43), and Nigeria (51). This could be explained by physiological and anatomical differences between males and females, such as males' shorter urethra, a decrease in normal vaginal flora, and females have less acidic vaginal surface pH (40).

In the current study, about 52.9%, 52.9%, and 58.8% of the total isolated Gram-negative uropathogens were susceptible to ceftriaxone, ciprofloxacin, and gentamicin, respectively, whereas, 51%, 72.5%, 72.3%, 84.3%, 60.8%, and 60.8% of the isolates were resistant to amikacin, ampicillin, amoxicillin-Clavulanic acid, and trimethoprim-sulphamethoxazole. The

other study on urinary tract bacterial and fungal isolates supported these current findings with Frequency Percentage sulphamethoxazole, nitrofurantoin, and ceftazidime and Most of the urinary bacterial isolates were highly sensitive to ceftazidime (95%) and ciprofloxacin (88% (54).

In this study, 87.5%, 87.5%, 81.3%, 75%, 62.5%, and 56 % of *E. coli* strains were resistant to tetracycline, Trimethoprim-sulphsmethsoxazole, amoxicillin-Clavulanic acid, ampicillin, nitrofurantoin, and ceftazidime, respectively. Similarly, 80% of ampicillin, 90.9% of trimethoprim-sulphsmethsoxazole, 90.9% of tetracycline, 63.6% of nitrofurantoin, and 63.6% of ceftazidime, 54.5% was resistance to *K. pneumoniae*. There were also similar findings from studies conducted in Addis Ababa which indicated *K. pneumoniae* had the highest level of resistance against trimethoprim-sulphamethoxazole (86.4%) cefotaxime (86.4%), cefepime (85.4%), ceftazidime (85.4%), amoxicillin-clavulanic acid (85.4%), gentamicin (70.0%), and ciprofloxacin (50.5% (55), In Iran: trimethoprim-sulphamethoxazole (91.4%), ceftazidime (91.4%), and gentamicin (82.8%) (56) ; in Sierra Leone: ciprofloxacin (73.4%) and gentamicin (60%) (57), in Equatorial Guinea: trimethoprim-sulphamethoxazole (100%), amoxicillin-clavulanic acid (96.6%), gentamicin (86.2%) and ciprofloxacin (87.5% (58). The occurrence of high antibiotic resistance might be due to misuse and overuse of antibiotics, and poor infection control measures (59) This was comparable with the study done in Gondar, Ethiopia (49) and Harar, Ethiopia (41), *P. aeruginosa* was 100% resistant to ampicillin, cefotaxime, and gentamicin and trimethoprim-sulfamethoxazole and *P.mirabilis* 100% resistant to ampicillin. This is in agreement with a study done in Ethiopia (40).

Similarly, about 87.5%, 83.3%, 75%, 58.3%, and 58.3% of Gram-positive isolates were resistant to tetracycline, trimethoprim-sulfamethoxazole, penicillin, chloramphenicol, and nitrofurantoin, respectively. The dominant isolated Gram-positive *Enterococcus species* showed more resistance to 90% of tetracycline, 80% of trimethoprim-sulphamethoxazole and 70% of nitrofurantoin. Likewise, *coagulase-negative staphylococci* were resistant to 87.5% of tetracycline, 87.5% of trimethoprim-sulphamethoxazole, and 75% of penicillin and chloramphenicol, respectively. This is in line with a study done in Harar, Ethiopia, where 57.1% of S. aureus were resistant to gentamicin, tetracycline, and cefoxitin (57.1%) (41). We also found that 85% of *Coagulase-negative staphylococcus spp*. were resistant to Trimethoprim-sulphamethaxazole (85%) and over 60% were resistant to penicillin, gentamicin, and tetracycline.

Multidrug resistance has serious implications for the health outcome of HIV-infected patients (60). It is quite alarming to note that almost 61(81.3%) of currently isolated bacteria were found to be resistant to three or more antimicrobials. This was higher compared to previous findings reported in Harar, Ethiopia (46%) (41), Dessie (74.6%) (61), Gondar (68%) (62) and Nepal (64.04%)(63) and India (28%) (64) . But it was lower than a report from Gondar, Ethiopia (95%) and 87.4%) (12, 49), Addis Ababa (100%) (40), Bahirdar (93.1%) (65), Nepal (96.84%) (63). These differences might be due to the irrational drug utilization habit of the communities or to distribution of those sensitive and resistant strains of bacteria.

There was an intra-species difference in MDR level. The present study showed that the level of MDR in *E. coli* 13 (81.3%), *K. pneumoniae* 9(81.8%), *Enterococcus species* 8(80%), *P. aeruginosa* 7 (77.8%), and CoNs 7(87.5%). This was comparable with a study conducted in Addis Ababa *K. pneumoniae* (83.5%) and *E. coli* (66.2%) (66), in Equatorial Guinea *E. coli* (74.4%) (58); in Sierra Leone *K. pneumoniae* (73.3%) and *E. coli* (61.5%) (57); in Gondar, *K. pneumoniae* was (95.6%) and *E. coli* was (92.9%) (12); in Khartoum-Sudan *E. coli* (92.2%)(67); and in Equatorial Guinea in *K. pneumoniae* (91.7%) (58). The MDR level among E. coli (50.2%) in Dessie is lower than in our study(61). The difference in MDR levels between *K. pneumoniae* and *E. coli* in our study might be most K. pneumoniae were isolated from blood specimens that were collected from inpatients in the hospitals.

In this study, there was no statistically significant association between significant bacteriuria and patients' residence, marital status, dysuria, frequency of urination, flank pain, and use of cotrimoxazole. However, age, gender, occupation, educational status, current CD4+ count (cells/mm3), previous hospitalization and diabetes status, history of catheterizations, and urinary tract urgency had a statistically significant association with significant bacteriuria. Similar findings were reported from Jimma, Addis Ababa, and Harar (37, 40, 41). Furthermore, age groups >44year [AOR=1.56, 95%CI: 1.11-9.35, P=0.001], being female [AOR=3.29, 95%CI: 1.26- 13.41, P=0.002], being daily labor [AOR=4.54, 95%CI: 1.41-21.71, P=0.003], being farmer [AOR=6.54, 95%CI: 1.21-23.51, P=0.004], participants unable to read and write [AOR=4.58, 95%CI: 1.21-13.58, P=0.001], HIV patients with CD4 count of ≤ 200 cells/mm3 (AOR: 7.44; 95%CI: 1.64-27.21, P=0.001) and CD4 count of 201-350 cells/mm3 (AOR: 4.55; 95%CI: 1.36-18.28, P=0.009), patients who had chronic diabetics (AOR: 4.80; 95%CI: 1.85-16.31, p=0.009), patients having a history of hospitalization (AOR: 6.27; 95%CI: 1.25-15.17, p=0.002) and patients

who had the urgency of urination (AOR: 6.51; 95%CI: 1.75-36.31, p=0.0025). Studies conducted in Nepal support our findings with a significant risk factor to community-acquired urinary tract infections in patients with indwelling urinary catheters (38.3%), Diabetes-mellitus (36%), malignancies (5.2%), and urinary incontinence (11.7%). Urinary catheters are a proven source of infection, especially when they are kept for longer periods (47). Similarly, research finding was supported by studies conducted elsewhere [26, 22, 27, and 29]. The explanation for the inverse relationship between UTI and a CD4+ count is unknown; it is probably due to the impaired immunity at a declining CD4+count that makes it easier for bacterial pathogens to adhere to the urinary epithelium.

Limitations of the study

The study was a single hospital-based study and might not represent all HIV-infected patients and did not include a control group due to budget constraints. We did not attempt to identify other causative agents (anaerobic bacteria, and fungus) that would have made a significant contribution to a true prevalence of UTI in HIV-positive patients.

Conclusions and Recommendations

The symptomatic community's acquired UTI is high, with an overall prevalence of 26.6% (92/346). Of the isolated pathogens, 21.67% (75/346) were bacterial pathogens, while 4.9% (17/346) were fungal pathogens. Among bacterial pathogens, *E. coli*, the most frequent bacterium, followed by *K. pneumoniae*, *enterococcus species*, and *P. aeruginosa*, accounting for, respectively, the overall prevalence of MDR bacteria was 61(81.3%). About 13 (81.3%) of E. coli, 9(81.8%) of *K. pneumoniae*, 8(80%) of *Enterococcus species*, 7 (77.8%) of *P. aeruginosa*, and *CoNs* 7(87.5%). Most Gram-negative and Gram-positive isolates were resistant to tetracycline, trimethoprim-sulfamethoxazole, penicillin, chloramphenicol, and Nitrofurantoin, respectively. However, uropathogens were susceptible to amikacin, it was more effective against these pathogens. Patients with low CD4 counts were more at risk of developing bacterial urinary tract infections, previous hospitalization, diabetes status, and having a history of catheterizations than their counterparts. Management of UTI among symptomatic HIV patients should be supported by laboratory results of urine culture. Regular monitoring of antimicrobial resistance patterns should be undertaken by healthcare providers. Individuals at risk for bacterial-associated UTI should have good adherence to ART and their CD4 levels should be monitored on a regularly. Health information dissemination should be given about UTI and the habit of drug use for HIV-positive patients.

List of abbreviation and acronyms

AIDS; Acquired immune deficiency syndrome, ARTC; Attending antiretroviral therapy clinic, ART; Antiretroviral therapy, ATCC; American Type Culture collections, BA; Blood agar, CFU; Colony Forming Unit, CLED; Cysteine Lactose Electrolyte Deficient, CONS; Coagulase-negative Staphylococci, HAART; Highly active antiretroviral therapy, HIV; Human Immunodeficiency Virus, MACA; MacConkey agar, MDR; Multi-Drug Resistant, MSU; Midstream urine, OIS; Opportunistic infections, UTI; Urinary Tract Infection, VCT Voluntary counseling , and Testing.

Data availability statement

Data supporting the conclusions of this article are within the manuscript and ae available on reasonable request from the principal investigators due to ethical reasons.

Funding

No external funds were obtained only internal support was obtained from Wollo University. The funders had no role in study design, data collection and analysis, the decision to publish, or preparation of the manuscript.

Competing interests

The authors declare that they have no competing interests.

Authors' contributions

Conceived and designed the experiments: Mihret Tilahun, Chernet Belayhun, Abdurrahman Seid, Bekele Sharew, Tewodros Dessie, Habtamu Mekonen; Performed the experiments: Mihret Tilahun, Abdurrahman Seid, Agumas Shibabaw; Analyzed the data: Mihret Tilahun, Abdurrahman Seid, Daniel G/tsadik, Mesfin Fiseha, Melkam Tesfaye, Alemu Gedefie, Melaku Ashagrie Belete; Wrote and edited the manuscript: Mihret Tilahun, Abdurrahman Seid, Bekele Sharew, Alemu Gedefie, Habtu Debash, Agumas Shibabaw, Melaku Ashagrie Belete, and Habtamu Mekonnen. All authors read and approved the final manuscript.

Acknowledgment

The authors would like to acknowledge the Department of Medical Laboratory Science, College of Medicine and Health Sciences, Wollo University for providing the laboratory set up and facilities to conduct the experiments. Dessie comprehensive specialized hospital and all study participants are gratefully acknowledged for their kind cooperation during data collection.

References

1. Syed B, Salahuddin N, Ishtiaq H. Etiologies and microbiological profile of complicated urinary tract infections, among patients admitted in a tertiary care hospital. *Infec Dis J Pakistan*. 2017;26:59-64.
2. Kiranmala K, Johnson R, Savio J, Idiculla J. Microbiologic profile and clinical practices in urinary tract infections in a tertiary care center in Southern India. *Journal of Family Medicine and Primary Care*. 2019;8(9):2888.
3. Nerurkar A, Solanky P, Naik SS. Bacterial pathogens in urinary tract infection and antibiotic susceptibility pattern. *Journal of Pharmaceutical and Biomedical Sciences*. 2012;21(21).
4. Ade-Ojo I, Oluyege A, Adegun P, Akintayo A, Aduloju O, Olofinbiyi B. Prevalence and antimicrobial susceptibility of asymptomatic significant bacteriuria among new antenatal enrollees in Southwest Nigeria. *International Research Journal of Microbiology*. 2013;4(8):197-203.
5. Schmider J, Bühler N, Mkwatta H, Lechleiter A, Mlaganile T, Utzinger J, et al. Microbiological characterisation of community-acquired urinary tract infections in Bagamoyo, Tanzania: *A prospective study*. *Tropical Medicine and Infectious Disease*. 2022;7(6):100.
6. Tegegne KD, Wagaw GB, Gebeyehu NA, Yirdaw LT, Shewangashaw NE, Kassaw MW. Prevalence of urinary tract infections and risk factors among diabetic patients in Ethiopia, a systematic review and meta-analysis. *PloS one*. 2023;18(1):e0278028.
7. Birhanu MY, Habtegiorgis SD, Gietaneh W, Alemu S, Tsegaye TB, Bekele GM, et al. Magnitude and associated factors of urinary tract infections among adults living with HIV in Ethiopia. Systematic review and meta-analysis. *Plos one*. 2022;17(4):e0264732.
8. Çoban B, Ülkü N, Kaplan H, Topal B, Erdoğan H, Baskın E. Five-year assessment of causative agents and antibiotic resistances in urinary tract infections. *Turkish Archives of Pediatrics/Türk Pediatri Arşivi*. 2014;49(2):124.
9. Flores-Mireles AL, Walker JN, Caparon M, Hultgren SJ. Urinary tract infections: epidemiology, mechanisms of infection and treatment options. *Nature reviews microbiology*. 2015;13(5):269-84.
10. Odabasi Z, Mert A. Candida urinary tract infections in adults. *World journal of urology*. 2020;38(11):2699-707.

11. Behzadi P, Behzadi E, Ranjbar R. Urinary tract infections and Candida albicans. *Central European journal of urology*. 2015;68(1):96-101.
12. Eshetie S, Unakal C, Gelaw A, Ayelign B, Endris M, Moges F. Multidrug resistant and carbapenemase producing Enterobacteriaceae among patients with urinary tract infection at referral Hospital, Northwest Ethiopia. *Antimicrobial resistance and infection control*. 2015;4(1):1-8.
13. Ahmed SS, Shariq A, Alsalloom AA, Babikir IH, Alhomoud BN. Uropathogens and their antimicrobial resistance patterns: Relationship with urinary tract infections. *International Journal of Health Sciences*. 2019;13(2):48.
14. Prakash D, Saxena RS. Distribution and antimicrobial susceptibility pattern of bacterial pathogens causing urinary tract infection in urban community of meerut city, India. *International scholarly research notices*. 2013;2013.
15. Haile Hantalo A, Haile Taassaw K, Solomon Bisetegen F, Woldeamanuel Mulate Y. Isolation and Antibiotic Susceptibility Pattern of Bacterial Uropathogens and Associated Factors Among Adult People Living with HIV/AIDS Attending the HIV Center at Wolaita Sodo University Teaching Referral Hospital, South Ethiopia. *HIV/AIDS-Research and Palliative Care*. 2020:799-808.
16. Fenta G, Legese M, Weldearegay G. Bacteriuria and their antibiotic susceptibility patterns among people living with HIV attending Tikur Anbessa Specialized and Zewditu Memorial Hospital ART Clinics, Addis Ababa, Ethiopia. *J Bacteriol Parasitol.* 2016;7(05).
17. Yismaw G, Asrat D, Woldeamanuel Y, Unakal CG. Urinary tract infection: bacterial etiologies, drug resistance profile and associated risk factors in diabetic patients attending Gondar University Hospital, Gondar, Ethiopia. *European Journal of Experimental Biology*. 2012;2(4):889-98.
18. Kibret M, Abera B. Prevalence and antibiogram of bacterial isolates from urinary tract infections at Dessie Health Research Laboratory, Ethiopia. *Asian Pacific journal of tropical biomedicine*. 2014;4(2):164-8.
19. Paschke AA, Zaoutis T, Conway PH, Xie D, Keren R. Previous antimicrobial exposure is associated with drug-resistant urinary tract infections in children. *Pediatrics*. 2010;125(4):664-72.
20. Frank-Peterside N, Okerentugba P, Nwodo C, Okonko I. Prevalence of bacterial uropathogens in a cohort of HIV-positive males in Port Harcourt, Nigeria. Cancer Biology. 2013;3(4):12-7.

21. Skrzat-Klapaczyńska A, Matłosz B, Bednarska A, Paciorek M, Firląg-Burkacka E, Horban A, et al. Factors associated with urinary tract infections among HIV-1 infected patients. *PLOS one*. 2018;13(1):e0190564.
22. Bitew A, Zena N, Abdeta A. Bacterial and fungal profile, antibiotic susceptibility patterns of bacterial pathogens and associated risk factors of urinary tract infection among symptomatic pediatrics patients attending St. Paul's Hospital Millennium Medical College: a cross-sectional study. *Infection and drug resistance*. 2022;15:1613.
23. Foxman B. Urinary tract infection syndromes: occurrence, recurrence, bacteriology, risk factors, and disease burden. *Infectious Disease Clinics*. 2014;28(1):1-13.
24. Lee Y-C, Hsiao C-Y, Hung M-C, Hung S-C, Wang H-P, Huang Y-J, et al. Bacteremic urinary tract infection caused by multidrug-resistant Enterobacteriaceae are associated with severe sepsis at admission: implication for empirical therapy. *Medicine*. 2016;95(20).
25. Islam MA, Islam MR, Khan R, Amin MB, Rahman M, Hossain MI, et al. Prevalence, etiology and antibiotic resistance patterns of community-acquired urinary tract infections in Dhaka, Bangladesh. *Plos one*. 2022;17(9):e0274423.
26. Haider MZ, Annamaraju P. Bladder Catheterization. StatPearls [Internet]: StatPearls Publishing; 2022.
27. Magiorakos A-P, Srinivasan A, Carey RB, Carmeli Y, Falagas M, Giske C, et al. Multidrug-resistant, extensively drug-resistant and pandrug-resistant bacteria: an international expert proposal for interim standard definitions for acquired resistance. *Clinical microbiology and infection.* 2012;18(3):268-81.
28. Bigwan E, Wakjissa F. Prevalence of urinary tract infections among HIV patients attending a non-governmental health facility in Jos, Plateau State, Nigeria. *International Journal of Biomedical and Advance Research.* 2013;4(8):528-33.
29. Murray PR, Rosenthal KS, Pfaller MA. Medical microbiology E-book: *Elsevier Health Sciences*; 2020.
30. Pezzlo M. Laboratory diagnosis of urinary tract infections: guidelines, challenges, and innovations. *Clinical Microbiology Newsletter*. 2014;36(12):87-93.
31. Bullock NO, Aslanzadeh J. Biochemical profile-based microbial identification systems. Advanced techniques in diagnostic microbiology: Springer; 2013. p. 87-121.

32. Malik UM, Khan AB, Satti ML. Comparative evaluation of CHROMagar and API 20C AUX in isolation and identification of Candida species. *Journal of Islamic International Medical College* (JIIMC). 2018;13(2):85-90.
33. Institute CLS. CLSI Performance standard for antimicrobial susceptibility testing, M100. 2021.
34. Behzadi P, Urbán E, Matuz M, Benkő R, Gajdács M. The role of gram-negative bacteria in urinary tract infections: current concepts and therapeutic options. *Advances in Microbiology, Infectious Diseases and Public Health.* 2020:35-69.
35. Nigussie D, Amsalu A. Prevalence of uropathogen and their antibiotic resistance pattern among diabetic patients. *Turkish Journal of Urology*. 2017;43(1):85.
36. Nabaigwa BI, Mwambi B, Okiria J, Oyet C. Common uropathogens among diabetic patients with urinary tract infection at Jinja Regional Referral Hospital, Uganda. *African Journal of Laboratory Medicine*. 2018;7(1):1-3.
37. Debalke S, Cheneke W, Tassew H, Awol M. Urinary tract infection among antiretroviral therapy users and nonusers in Jimma University Specialized Hospital, Jimma, Ethiopia. *International journal of microbiology*. 2014;2014.
38. Girma A, Aemiro A. The Bacterial Profile and Antimicrobial Susceptibility Patterns of Urinary Tract Infection Patients at Pawe General Hospital, Northwest Ethiopia. *Scientifica.* 2022;2022.
39. Tessema NN, Ali MM, Zenebe MH. Bacterial associated urinary tract infection, risk factors, and drug susceptibility profile among adult people living with HIV at Haswassa University Comprehensive Specialized Hospital, Hawassa, Southern Esthiopia. *Scientific Reports*. 2020;10(1):1-9.
40. Getu Y, Ali I, Lema T, Belay H, Yeshetela B. Bacteriuria and antimicrobial susceptibility pattern among HIV patients attending ALERT Center, Addis Ababa, Ethiopia. *Am J Health Res*. 2017;5(3):76-82.
41. Marami D, Balakrishnan S, Seyoum B. Prevalence, Antimicrobial Susceptibility Pattern of Bacterial Isolates, and Associated Factors of Urinary Tract Infections among HIV- Positive Patients at Hiwot Fana Specialized University Hospital, Eastern Ethiopia. C*anadian Journal of Infectious Diseases and Medical Microbiology*. 2019;2019.
42. Okechukwu A, Thairu Y. Bacteria urinary tract infection in HIV-infected children and adolescents in Abuja, Nigeria: a cross-sectional study. *African Journal of Clinical and Experimental Microbiology*. 2019;20(4):306-14.

43. Iweriebor B, Obi C, Akinyemi O, Ramalivhana N, Hattori T, Okoh A. Uropathogens isolated from HIV-infected patients from Limpopo Province, South Africa. *African Journal of Biotechnology*. 2012;11(46):10598-604.
44. Xavier TF, Auxilia A, Kannan M. Isolation and characterization of UTI pathogens from HIV positive patients of Karur District, Tamil Nadu, India. *Int J Curr Microbiol Appl Sci.* 2015;4(1):558-63.
45. Sharma V, Gupta V, Mittal M. Prevalence of uropathogens in diabetic patients and their antimicrobial susceptibility pattern. *Natl J lab Med*. 2012;1(1):26-8.
46. Achkar JM, Fries BC. Candida infections of the genitourinary tract. Clinical microbiology reviews. 2010;23(2):253-73.
47. Aadil Rafeeq Rather1* ANKaTM. Profile of Community Acquired Urinary Tract Infections at a Teriary Care Hospital. Volume 2 Issue 11 November 2019. 2019.
48. Erdem I, Ali RK, Ardic E, Omar SE, Mutlu R, Topkaya AE. Community-acquired lower urinary tract infections: Etiology, antimicrobial resistance, and treatment results in female patients. J*ournal of global infectious diseases*. 2018;10(3):129.
49. Alemu A, Dagnew M, Alem M, Gizachew M. Uropathogenic bacterial isolates and their antimicrobial susceptibility patterns among HIV/AIDS patients attending Gondar University Specialized Hospital Gondar, Northwest Ethiopia. 2013.
50. Biradar SK, Doddamani P. Prevalence and antibiogram of uropathogens in a tertiary care hospital. *World J Pharmaceut Res*. 2013;2:1534-43.
51. Ifeanyichukwu I, Emmanuel N, Chika E, Anthonia O, Esther U-I, Ngozi A, et al. Frequency and antibiogram of uropathogens isolated from urine samples of HIV infected patients on antiretroviral therapy. *American Journal of BioScience*. 2013;1(3):50-3.
52. Ejerssa AW, Gadisa DA, Orjino TA. Prevalence of bacterial uropathogens and their antimicrobial susceptibility patterns among pregnant women in Eastern Ethiopia: hospital-based cross-sectional study. *BMC women's health*. 2021;21(1):1-15.
53. Fierer J, Looney D, Kok M, Pechère J-C. Nature and pathogenicity of micro-organisms. Infectious diseases. 2010:3.
54. Nwadioha S, Nwokedi E, Ikeh I, Egesie J, Kashibu E. Antibiotic susceptibility pattern of uropathogenic bacterial isolates from AIDS patients in a Nigerian tertiary hospital. *J Med Med Sci*. 2010;1(11):530-4.

55. Teklu DS, Negeri AA, Legese MH, Bedada TL, Woldemariam HK, Tullu KD. Extended-spectrum beta-lactamase production and multi-drug resistance among Enterobacteriaceae isolated in Addis Ababa, Ethiopia. *Antimicrobial Resistance & Infection Control.* 2019;8:1-12.
56. Mansouri S, Abbasi S. Prevalence of multiple drug resistant clinical isolates of extended-spectrum beta-lactamase producing Enterobacteriaceae in Southeast Iran. 2010.
57. Leski TA, Taitt CR, Bangura U, Stockelman MG, Ansumana R, Cooper WH, et al. High prevalence of multidrug resistant Enterobacteriaceae isolated from outpatient urine samples but not the hospital environment in Bo, Sierra Leone. *BMC infectious diseases.* 2016;16(1):1-9.
58. Shatalov A. Prevalence and antibiotic resistance pattern of Escherichia coli and Klebsiella pneumoniae in urine tract infections at the La Paz Medical center, Malabo, Equatorial Guinea. *Open Journal of Medical Microbiology*. 2015;5(04):177.
59. Adler A, Katz DE, Marchaim D. The continuing plague of extended-spectrum β-lactamase–producing Enterobacteriaceae infections. *Infectious Disease Clinics.* 2016;30(2):347-75.
60. Kim H-R, Hwang SS, Kim HJ, Lee SM, Yoo C-G, Kim YW, et al. Impact of extensive drug resistance on treatment outcomes in non-HIV-infected patients with multidrug-resistant tuberculosis. *Clinical Infectious Diseases.* 2007;45(10):1290-5.
61. Kibret M, Abera B. Antimicrobial susceptibility patterns of E. coli from clinical sources in northeast Ethiopia. *African health sciences*. 2011;11:40-5.
62. Moges F, Mengistu G, Genetu A. Multiple drug resistance in Urinary pathogens at Gondar College of Medical science hospital, Ethiopia. East African medical journal. 2002;79(8):415-20.
63. Yadav KK, Adhikari N, Khadka R, Pant AD, Shah B. Multidrug resistant Enterobacteriaceae and extended spectrum β-lactamase producing Escherichia coli: a cross-sectional study in National Kidney Center, Nepal. *Antimicrobial resistance and infection control*. 2015;4(1):1-7.
64. Murugesh K, Deepa S, Ravindranath C, Venkatesha D. Multi drug resistant uropathogens in HIV: Are they a threat to community? International Journal of Scientific Study. 2014;2(3):38-42.
65. Biadglegne F, Abera B. Antimicrobial resistance of bacterial isolates from urinary tract infections at Felge Hiwot Referral Hospital, Ethiopia. *The Ethiopian Journal of Health Development.* 2009;23(3).
66. Teklu DS, Negeri AA, Legese MH, Bedada TL, Woldemariam HK, Tullu KD. Extended-spectrum beta-lactamase production and multi-drug resistance among Enterobacteriaceae isolated in Addis Ababa, Ethiopia. *Antimicrobial Resistance & Infection Control*. 2019;8(1):1-12.

67. Ibrahim M, Bilal N, Hamid M. Increased multi-drug resistant Escherichia coli from hospitals in Khartoum state, Sudan. *African health sciences.* 2012;12(3):368-75.

Figure 1 legends

Etiology of UTI among HIV patients attending Antiretroviral Therapy Clinics of Northeast Ethiopia

Author’s Response to Editor and Reviewer comments

Manuscript ID number:
PONE-D- 22-31717

Title of paper:

Uropathogens, multidrug resistance pattern of bacteria and associated factors of community-acquired urinary tract infection among HIV positive patients attending antiretroviral therapy clinics of Northeast Ethiopia

General

We appreciate all of the constructive feedback on our manuscript from the reviewers, which has helped us improve the paper's clarity and scientific plausibility. We thank the PLOS ONE scholarly editors for their insightful comments and for allowing us to update the text. All of the concerns raised by the reviewers and editors have been addressed, and the text has been updated as a result. Furthermore, both the updated article and the response letter address all of the editorial revisions that are required. The changes are shown with track changes in the file labelled "Revised Manuscript with Track Modifications." The following is a detailed rebuttal.

Editor Comments:

Date: Jul 28 2023 02:00PM
To: "Alemayehu Tadesse" alextadesse21@gmail.com
From: "PLOS ONE" plosone@plos.org
Subject: PLOS ONE Decision: Revision required [PONE-D-22-31717R1]
Attachment(s): Reviewed manuscript PONE-D-22-3171.docx

Author’s Response

We modify the title Uropathogens, multidrug resistance pattern of bacterial and associated factors of community-acquired urinary tract infection among HIV positive patients attending Antiretroviral Therapy Clinics of Northeast Ethiopia. But there is mix up of the information’s when the editors sent the manuscript to the author the PONE-D-22-3171.docx is another title and this email is Alemayehu Tadesse" alextadesse21@gmail.com is not the authors email the correct author and corresponding author is Mihret Tilahun with email tilahunmihret21@gmail.com . even Alemayehu Tadesse is not coauthor in this paper please see again.

PONE-D-22-31717R1
Uropathogens, multidrug resistance pattern of bacterial and associated factors of community-acquired urinary tract infection among HIV positive patients attending Antiretroviral Therapy Clinics of Northeast Ethiopia
PLOS ONE

Dear Dr. Tadesse,

Thank you for submitting your manuscript to PLOS ONE. After careful consideration, we feel that it has merit but does not fully meet PLOS ONE's publication criteria as it
currently stands. Therefore, we invite you to submit a revised version of the manuscript that addresses the points raised during the review process.

==============================

The manuscript needs language editor.

the laboratory methods must be standard mainly the drug resistance test.

Author response

- Thanks for the critical comments the language parts and the e laboratory methods must be standard was edited.

==============================

Please submit your revised manuscript by Sep 11 2023 11:59PM. If you will need more time than this to complete your revisions, please reply to this message or contact the
journal office at plosone@plos.org. When you're ready to submit your revision, log on to https://www.editorialmanager.com/pone/ and select the 'Submissions Needing
Revision' folder to locate your manuscript file.

Please include the following items when submitting your revised manuscript:

A rebuttal letter that responds to each point raised by the academic editor and reviewer(s). You should upload this letter as a separate file labeled 'Response to Reviewers'.

A marked-up copy of your manuscript that highlights changes made to the original version. You should upload this as a separate file labeled 'Revised Manuscript with Track Changes'.

An unmarked version of your revised paper without tracked changes. You should upload this as a separate file labeled 'Manuscript'.

Author response

- Thanks for the critical comments the A rebuttal letter that responds to each point raised by the academic editor and reviewer(s). You should upload this letter as a separate file labeled 'Response to Reviewers and A marked-up copy of your manuscript that highlights changes made to the original version. You should upload this as a separate file labeled 'Revised Manuscript with Track Changes And unmarked version of your revised paper without tracked changes were uploaded previously and now again we can do it

If you would like to make changes to your financial disclosure, please include your updated statement in your cover letter.

The financial disclosure continues as it is

Guidelines for resubmitting your figure files are available below the reviewer comments at the end of this letter.

If applicable, we recommend that you deposit your laboratory protocols in protocols.io to enhance the reproducibility of your results. Protocols.io assigns your

protocol its own identifier (DOI) so that it can be cited independently in the future. For instructions see: https://journals.plos.org/plosone/s/submission-guidelines#loc-laboratory-protocols.

Additionally, PLOS ONE offers an option for publishing peer-reviewed Lab Protocol articles, which describe protocols hosted on protocols.io. Read more information on sharing protocols at https://plos.org/protocols?utm_medium=editorial-email&utm_source=authorletters&utm_campaign=protocols.

We look forward to receiving your revised manuscript

Author response

Thanks for the laboratory protocols are the same as mentioned in the table format

Comments to authors

. The new title of this manuscript is confusing and still lengthy. Authors should consider this title: Please consider, “Uropathogens: multidrug resistance and risk factors of community-acquired UTI among HIV patients attending antiretroviral therapy clinics of Northeast Ethiopia”.

Author response

Thanks for the the title are modified based on recommendation to **Uropathogens: multidrug resistance and risk factors of community-acquired UTI among HIV patients attending antiretroviral therapy clinics of Northeast Ethiopia**

2. The manuscript still has a significant number of omissions, typographical and grammatical errors. Authors will need to carefully go through the manuscript for thorough corrections or better still, employ the services of a script/copy editor. (eg. Another not “An-other”; caused not “causes”; CT-scan not “city-scan”; use of upper-case within sentences; etc…)

Author response

Thanks for the the title are modified based on recommendation to **made corrections and highlighted**

3. All genus and specie names of microorganisms should be italicized. This remains pesistent in the manuscript (eg. Fungi species like C. albicans, C. glabrata, and C. tropicalis, in several parts of the manuscript and are not italicized).

Author response

Thanks for the the title are modified based on recommendation to **made corrections and highlighted and italized**

4. The authors have not discussed why they could not establish any association between bacteriuria and symptoms of UTI in their stud

Author response

Thanks for the title are modified based on recommendation to **we have discussed on the the total**

Finally, we thank you for your critical review of the paper.

Sincerely,

Mihret Tilahun

Manuscript

Click here to access/download;Manuscript;Round two Revised manuscript clean.docx

Uropathogens: Multidrug resistance and risk factors of community-acquired UTI among HIV patients attending antiretroviral therapy clinics of Northeast Ethiopia

Mihret Tilahun[1*], Mesfin Fiseha[1], Mihreteab Alebachew[1], Alemu Gedefie[1], Endris Ebrahim[1], Melkam Tesfaye[1], Melaku Ashagrie Belete[1], Abdurahaman Seid [1], Daniel Gebretsadik [1], Ermiyas Alemayehu[1], Wondmagegn Demsiss[1], Bekele Sharew[1], Agumas Shibabaw[1], Habtamu Mekonnen[2], Tewodros Dessie [2]

[1]Department of Medical Laboratory Sciences, College of Medicine and Health Science, Wollo University, Dessie, Ethiopia

[2] Amhara Public Health Institute Dessie branch

Authors' information:

Mihret Tilahun (MSc in Medical Microbiology): tilahunmihret21@gmail.com

Mesfin Fiseha (MSc in hematology and immunohematology): mesfinfiseha40@gmail.com

Alemu Gedefie (MSc in Medical Microbiology): alemugedefie@gmail.com

Mihreteab Alebachew (MSc in medical parasitology and vector control): mihreteab71@gmail.com

Endris Ebrahim (MSc in immunology): endris.index@gmail.com

Melkam Tesfaye (Ph.D. fellow in infectious diseases): Melkam66@gmail.com

Melaku Ashagrie Belete (MSc in Medical Microbiology): melakuashagrie@gmail.com

Abdurahaman Seid (MSc., Asst Prof in Medical Microbiology): gechdk2000@yahoo.com

Daniel Gebretsadik (MSc., Asst Prof in Tropical and infectious diseases): gebretsadikd@gmail.com

Ermiyas Alemayehu ((MSc in clinical chemistry): ermiyas0009@gmail.com

Wondmagegn Demsiss (MSc., Asst Prof in Medical Microbiology): *wonde1980@gmail.com*

Bekele Sharew (Ph.D., Asst Professor in Medical Microbiology): bsharew@gmail.com

Agumas Shibabaw (Ph.D., Asst Professor in Medical Microbiology): agumas2000@gmail.com

Habtamu Mekonnen (MSc in Medical Microbiology): habtamumeko36@gmial.com

Tewodros Dessie (MSc in Medical Microbiology): tedydess21@gmial.com

Corresponding author

Mihret Tilahun (MSc.)

Department of Medical Laboratory Science

College of Medicine and Health Sciences,
Wollo University, Dessie, Ethiopia
Telephone: +251- 920988307
Fax: +251 333115250
PO.BOX: 1145
Email: tilahunmihret21@gmail.com

Abstract

Background: Urinary tract infections are the common bacterial and fungal infections in humans, occurring both in the community and in immunocompromised patients in health care settings. UTI has significant health impact on HIV-infected patients. Drug resistance pathogens is now widespread and poses a serious clinical risk and causes urinary tract infection. This study investigated uropathogens, multidrug resistance pattern of bacteria and associated factors of community-acquired urinary tract infection among HIV positive patients attending antiretroviral therapy clinic of Northeast Ethiopia from February 1, 2021, to March 30, 2021.

Methods: Institutional based cross-sectional study was conducted at Dessie comprehensive specialized Hospital. Socio-demographic and clinical data were collected by using structured questionnaire from HIV patients suspected of community-acquired urinary tract infection. About 10 ml of clean-catch midstream urine was collected and inoculated into Blood agar, MacConkey, and Cysteine lactose electrolyte deficient media. Gram stain and biochemical tests were performed to identify isolates and antimicrobial susceptibility pattern was performed on disc diffusion techniques. Data were entered and analyzed using SPSS version-25. Both bivariate and multivariable logistic regression analysis was performed and a P value of < 0.05 with an adjusted odds ratio with their 95% confidence interval (CI) was used as statistically significant associations.

Results: From the total 346 study participants, 92 (26.6%) were culture positive and 75 (81.52%) were bacterial and 17 (18.48%) were fungal pathogens. From a total of 75 bacteria isolates 51(68%) were Gram-negative bacteria and the most commonly isolated bacteria were *E. coli* 16 (21.33%) followed by *K. pneumoniae* 11(14.67%) and *enterococcus species* 10(10.87. Of the 17 fungal isolates of fungi, 8(47.1%) were represented *by C. tropicalis.* The overall prevalence of Multi-Drug Resistance bacteria was 61(81.3%). About 13 (81.3%) of *E. coli*, 9(81.8%) of *K. pneumoniae*, 8(80%) of *Enterococcus* species, 7 (77.8%) of *P. aeruginosa*, and *CoNs* 7(87.5%) were the most frequently exhibited multi-drug Resistance. Amikacin, and gentamicin were effective against Gram-negative uropathogens. Participants with age >44year, being female, being daily labor, being farmer, unable to read and write, patients with CD4 count of ≤ 200 cells/mm^3 and CD4 count of 201-350 cells/mm^3, who had chronic diabetics, patients having a history of

hospitalization and who had urgency of urinations were statistically significant association with significant urinary tract infections.

Conclusion: The burden of community acquired urinary tract infections among HIV patients is alarmingly increased. Therefore, behavior change communications might be considered for promoting the health status of HIV patients. Moreover, CD4 level monitoring and therapeutics selection based on microbiological culture are quite advisable for management of urinary tract infections of HIV patients
Keywords: Bacteriuria, Candidauria, Uropathogens, community-acquired UTI, Multidrug resistance, Ethiopia

Introduction

Urinary tract infection (UTI) is an infection of the urinary tract, including the kidneys, ureters and bladder, urethra, and accessory structures that collect, store and release of urine from the body. Urinary tract infection occurs when microorganisms, typically bacteria from the digestive tract, entering to the urethral opening and begin to multiply (1-3).

Globally, the burden of UTI is estimated to be 150 million cases every year (4). Based on previous epidemiological studies from Tanzania and elsewhere in sub-Saharan Africa, urinary tract infections were estimated from 35–45% cases (5). In Ethiopia systematic review and meta-analysis indicated that, the pooled prevalence of urinary tract infection was 15.97%(6) and urinary tract infections in HIV patients in Ethiopia were 12.8% (7).

Urinary tract infections are caused by bacteria, as well as certain fungi and *Escherichia coli (E. coli)* is the most common cause of both uncomplicated and complicated UTIs, followed by *Klebsiella pneumonia (K.pneumoniae)*, *Staphylococcus saprophyticus (S.saprophyticus)*, *Enterococcus faecalis (E. faecalis)*, *group B streptococcus(GBS)*, *Proteus mirabilis (P.mirabilis)*, *Pseudomonas aeruginosa* (*P. aeruginosa)*, *Staphylococcus aureus (S.aureus)*, and *Candida* spp (8). Evidence showed that *Escherichia coli* is the most common causative agent for complicated UTIs. The order of prevalence for causative agents is *Enterococcus* spp., *K. pneumoniae*, *Candida* spp., *S. aureus*, *P. mirabilis*, *P. aeruginosa*, and GBS (9). Candidauria is the presence of *Candida spp.* in the urine, such as *C. albicans*. Patients with cystitis, epididymorchitis, prostatitis, pyelonephritis, and renal candidiasis experience symptomatic candidiasis (10). C. albicans is one of the most common fungi that can cause Candidauria (20% of nosocomial infections) (11).

In developing countries, the emergence of antibiotic resistance in the treatment of urinary tract infections is a major public health issue (12). Recently, the most common pathogens of UTIs developed resistance to most antimicrobial agents (13). *E. coli*, *Klebsiella species* and *Proteus species* had high resistance rates to amoxicillin and tetracycline of 80.1%–90.0% and low sensitivity rates of 0–25% to nitrofurantoin, ciprofloxacin, and gentamicin. Different evidences across the world and Africa including Ethiopia showed that more than 50% of uropathogenic bacteria were developed multidrug resistance (MDR). About 70.0% of bacteria from India (14), and in Ethiopia 79.3% from Wolayta Sodo Ethiopia (15), 78.4% from Addis Ababa (16) and 91.7% from Gondar (17) developed MDR. The antimicrobial test analysis showed that 152 (47.85%) of the isolates were resistant to two or more antimicrobials (18). Majority of treatments are initiated empirically,

knowledge of the organisms, their epidemiological characteristics, adequate treatment, control of these conditions necessitate and antibacterial susceptibility is required (19).

Urinary tract infections cause increased mortality, disability, hospitalization, economic loss in the HIV population, renal failure and hypertension. It is predisposed to nephritic complications such as urinary tract obstruction, which causes renal colic, pelvic-ureteric junction obstruction, benign prostate hyperplasia, urethral strictures, bladder dysfunction, and urinary stasis (20).

A systematic review indicated that being female, having a history of recurrent UTI, having low socioeconomic status, having diabetes mellitus, genitourinary tract abnormalities, having a low CD4 count, history of catheterization, history of hospitalization and chronic diabetics status were independent factors for the occurrence of urinary tract infection (7, 15). Another study on factors associated with urinary tract infections among HIV infected patients with low CD4 count, chronic hepatitis, and being female (21).

Despite numerous studies on bacterial UTI, antimicrobial susceptibility testing, and HIV infection-related factors in developed countries, there is little data on the burden of bacterial UTI and antimicrobial susceptibility patterns among UTI-symptomatic HIV-infected patients in resource-limited settings, including Ethiopia in general, and no study was done in the study area in particular. As a result, the purpose of this study was to determine the uropathogens, multidrug resistance pattern of bacteria and associated factors of community-acquired urinary tract infection among HIV positive patients attending antiretroviral therapy clinics of Northeast Ethiopia.

Methods and materials

Study design, area and period

A hospital based cross-sectional study was conducted at Dessie Comprehensive Specialized Hospital (DCSH) from February 1, 2021 to March 30, 2021. According to the Dessie town health administrative office, in the town, there are 16 governmental health institutions (1 comprehensive specialized hospital, 1 general hospital, 8 health centers) and 3 private general hospitals, 6 higher private clinics, and the hospital serves about 12 million people living in the catchment areas. The Dessie comprehensive specialized hospital serves about 12 million people living in the catchment areas and has a 600 average daily patient flow also has 800 staff, among whom about 600 are health professionals. It has 600 beds, 8 wards, and different OPDs like adult, pediatric, emergency, and TB & HIV. Also, the hospital provides different services like laboratory, pharmacy, imaging (X-

ray, ultra-sound and city scan), and Dessie comprehensive specialized hospital ART service serves for 9,500 HIV positive.

Population

The source of populations were all HIV-positive individuals attending at DCSH. Whereas, HIV-positive patients who had symptomatic to community acquired UTI and visited the ART clinic of Dessie comprehensive specialized hospital during the study period before 48 hours of admissions were the target populations.

Eligibility criteria of study participants

HIV-positive patients who were ≥18 years old with suggestive symptoms of UTI including; lower abdominal or flank pain, dysuria and hematuria, urgency, frequency urinations were included in the study while patients who were mentally ill and unable to give samples were not included in the study.

Variables

Dependent variable the prevalence of bacterial and fungal community-acquired UTI was the dependent variable. While socio-demographic characteristics like (age, sex, marital status, educational level, residence) and clinical profile like (history of catheterization in the past year, Current ART status, symptoms of UTI (dysuria, urgency, frequency, flank pain, and urine color), diabetes, CD4+cell count, cotrimoxazole usage, and history of hospitalization) in the past year were independent variables.

Sample size and sampling technique

The required sample size was determined by using single population proportion formula by considering the proportion 28.6%(22) from previous Ethiopian study, marginal error of 5%, and 95% confidence interval = 1.96 by using the following formula:

$$n = \frac{(Z_{a/2})^2 * p*(1-p)}{d^2}$$

The calculated sample size was 314. Including 10% non-response rate a total of **346 UTI** symptomatic HIV-patients and fulfilling the inclusion criteria were included consecutively.

Operational definitions

UTI: Urinary tract infections are one of the most common infections in humans that can happen anywhere along the urinary tract and it is caused by microorganisms (microscopic organisms that

are too small to be seen by the naked eye). There are several different types of microorganisms which include bacteria, protozoa, fungi and algae (23, 24).

Community-acquired urinary tract infection (CA-UTI); Community-acquired UTI is an infection if an individual with UTI is suspected before hospital admission and specimens are collected from the outpatient or within less than 48 hours of hospital admission (25).

History of catheterization: the use of or insertion of a catheter into the bladder or participants who have catheterized before to data collections (26).

History of hospitalization: participants who were taken to a health facility and kept there for 48 hrs for treatment before to data collections in the past six months or more months.

Multidrug resistance: A bacterium that is simultaneously resistant to three or more antimicrobial categories (27).

Data and specimen collection

A pre-tested structured questionnaire was used to collect information on socio-demographic data (age, residence, and occupation), and clinical history (existing antibiotic treatment, previous antibiotic therapy, previous history of hospitalization for a longer period). The participants' recent CD4+ count was retrieved from their medical records. About 10 mL of voided clean-catch midstream fresh urine was collected from each study participant by using a leak-proof and sterile wide-mouthed screw-capped container. These specimens were labeled and stored in a cold box (4°C) and transported to the Wollo University College of Medicine and Health Sciences microbiology laboratory for analysis within 1 hour (28).

Isolation and identification of bacteria

Bacterial isolation and phenotypic characterization were performed using the recommended culture and biochemical tests (29). A calibrated loop that delivers 0.001 mL of urine was used to inoculate each urine sample on to the Cysteine lactose electrolyte deficient agar (Oxoid Ltd, UK). The plates were incubated aerobically at 37°C for 24 hours and colony count growth of $\geq 10^4$-10^5CFU/mL (colony-forming units per milliliter) was considered significant. Gram stain was performed from significant growth and sub cultured on to MacConkey agar (HiMediaTM) and 5% blood agar plates (HiMediaTM). However, bacteria that did not show growth after 24 hrs of incubation were further incubated for 24 hours and discarded as negative and the colony count was not significant (30). Colony characteristics, Gram reactions, and a series of biochemical reactions, including catalase, coagulase, oxidase, urease, indole, citrate utilization, lysine decarboxylase, glucose, lactose

fermentation, gas and H2S production, and motility tests were used for the isolations of bacteria (31).

Fungal Identification

Yeasts were identified by using routine diagnostic methods such as Gram stain, germ tube test, carbohydrate fermentation, and assimilation tests, as well as chromogenic medium (CHROMagar Candida Medium (Biomerieux, France) according to the manufacturer's instructions (32).

Antimicrobial susceptibility testing

An antimicrobial susceptibility test was performed by using Kirby–Bauer disk diffusion method based on the clinical laboratory standards Institute (CLSI) recommendation (33). About 3–5 pure colonies of isolated species from nutrient agar were picked and transferred to a tube containing 5 mL of tryptone-soya broth and mixed well to make a homogenous suspension. The suspension was incubated at 37°C until the turbidity of the suspension matched to a 0.5 McFarland standard. Using a sterile swab, the suspension was inoculated over the entire surface of the Mueller Hinton agar plate. The selected antimicrobial disks were put on the inoculated plates and incubated at 37°C for 16–18 hours. Antimicrobial agents were selected based on CLSI recommendations and local (Ethiopian) prescription habits for bacteria. The antimicrobials (Oxoid Ltd) that were used for bacterial susceptibility testing were, amoxicillin-clavulanic acid (AMC) 10µg, ampicillin (AMP) 10µg, amikacin (Amk) 30µg, cefotaxime (CTX) 30µg, ceftriaxone (CRO) 30µg, trimethoprim- sulphamethoxazole (SXT) 25µg, ciprofloxacin (CIP) 5µg, gentamycin (Gen) 10 µg, ceftazidime (CAZ) 30µg, nitrofurantoin (F) 300µg, tetracycline (TE) 30µg and penicillin (Pen) 10µg (33). Within 15 minutes after the application of the discs, the plates were incubated at 35°c for 18 hours. Diameters of zones of inhibitions were measured using digital caliper. The antimicrobial susceptibility test results were interpreted as sensitive, intermediate, or resistant based on the standardized CLSI guidelines and the isolates were considered MDR, resistant at least one antimicrobial in three or more antimicrobial categories (33).

Quality assurance

The questionnaire was pretested on 5% of HIV-positive patients at Dessie health center. The sterility of culture media was checked by incubating 5 % the prepared media for overnight at 37 ºC without specimen inoculation. The collected data was checked for completeness and adequate recording on the worksheet both during and after data collection. Standard Operating Procedures (SOP) were strictly followed for each microbiological procedure. All clinical specimens were collected, transported, and processed correctly. The expiration dates of the media, reagents, and Muller Hilton agar antimicrobial discs were

checked before use. The new batch culture medium and antimicrobial disks were checked for performance and quality using the American Type Culture Collection (ATCC) reference strains such as *E. coli* (ATCC® 25922), *S. aureus* (ATCC® 25923), *Klebsiella pneumonia* (ATCC 700603) and *P. aeruginosa* (ATCC® 27853).

Data management and statistical analysis

The data was entered into epi-data version 4.6.0.4 and exported to Social Sciences Statistical Package (SPSS) version 25 for analysis. Descriptive statistics were computed and presented using graphs and tables. A bivariate analysis was conducted to classify factors that separately affect the frequency of dependent variables. Variables with a P-value less than or equal to 0.25 in the bivariate analysis were subjected to multi-variable analysis. Finally, variables with an adjusted odd ratio and a p-value of <0.05 was considered as statistically significant.

Ethical considerations

Ethical clearance and permissions were obtained from the ethical review committee of Wollo University College of Medicine and Health Sciences with protocol number CMHS 365/2021 and a support letter was written to Dessie comprehensive specialized Hospital. Permission was obtained from Dessie comprehensive specialized hospitals. Written assent was also obtained from family and surrogates to obtain information from those who can't give data. Information obtained from the study participants was kept confidential. Positive results were communicated and given to attending physicians for appropriate treatment.

Results

Socio-demographic characteristics

A total of 346 HIV-positive patients with a response rate of 100% were included in this study. The age of the study participants was ranged from 21 to 80 years with a mean (±SD) age of 39.44 (±10.87) years, and 116 (33.5%) of them were in the age range of 35–44 years. About 180 (52.02%) of the respondents were females. Regarding educational status, 115 (33.2%) of the respondents had a primary-level of education. Moreover, 90 (26.01%) were self-employed and 215 (62.1%) of them were urban dwellers **(Table 1).**

Table 1. Sociodemographic characteristics of UTI symptomatic HIV positive patients attending at Dessie comprehensive specialized hospital, Dessie, Ethiopia.

Variable	Category	Urinary tract infection		Total, No (%)	Percentage
		Positive No (%)	Negative No (%)		
Sex	Male	42(25.3)	124(74.7)	166	47.98
	Female	50(27.8)	130(72.2)	180	52.02
Residence	Rural	62(28.8)	153(71.2)	215	62.1
	Urban	30(22.9)	101(77.1)	131	37.9
Age (in years)	18–24	10(18.9)	43(81.1)	53	15.3
	25–34	21(24.7)	64(75.3)	85	24.6)
	35–44	30(25.9)	86(74.1)	116	33.5
	>44	31(33.7)	61(66.3)	92	26.6
Educational status	Unable to read and write	42(36.5)	73(63.5)	115	33.2
	Primary	20(23.3)	66(76.7)	86	24.9
	Secondary	22(24.2)	69(75.8)	91	26.3
	College/University	8(14.8)	46(85.2)	54	15.6
Marital status	Married	45(37.2)	76(62.8)	121	35
	Unmarried	18(22.5)	62(77.5)	80	23.1
	Divorced	13(13.7)	82(86.3)	95	27.5
	Widowed	16(32)	34(68)	50	14.5
Occupation	House Wife	15(25.9)	43(74.1)	58	16.8
	Civil Servant	10(22.2)	35(77.8)	45	13
	Self-Employee	9(18)	41(82)	50	14.5
	Driver	10(20.8)	38(79.2)	48	13.9
	Daily Labor	23(25.6)	67(74.4)	90	26.01
	Farmer	25(45.5)	30(55.5)	55	15.9

Clinical characteristics of study participants

The clinical picture of study participants revealed that 150 (43.4%) and 140(40.5%) had a history of previous hospitalization and catheterization, of which 35(30.4%) and 42(30%) were culture positive for community-acquired urinary tract infections, respectively. About 121(35%) of the respondents had diabetes during the period of the study, with positive growth of 32(25.6%). About 105 (30.3%) HIV patients had 201-350 cells/mm3 CD4 count with positive growth of 35(33.3%). Moreover, 210(60.7%), 200 (57.8%), 165 (47.7%), and 164 (47.4%) participants showed dysuria, frequency of urination, flank pain, and abnormal urine color with positive growth of 58(27.6%), 55(27.5%), 61 (37%), and 64 (39%), respectively (**Table 2)**.

Table 2. Frequency of clinical characteristics of UTI symptomatic HIV positive patients attending Dessie comprehensive specialized hospital, Dessie, Ethiopia (February 1, 2021-march 30, 2021).

Variables	Category	Culture Result		Frequency	Percentage
		Positive No (%)	Negative No (%)		
History of hospitalization	Yes	35(30.4)	115(69.6)	150	43.4
	No	57(29.1)	139(70.9)	196	56.6
Use of cotrimoxazole as prophylaxis	Yes	40(26)	114(74)	154	44.5
	No	54(29.1)	138(70.9)	192	55.5
Diabetes	Yes	32(25.6)	89(74.4)	121	35
	No	60(24.6)	184(75.4)	244	65
History of catheterization	Yes	42(30)	120(70)	140	40.5
	No	50(24.3)	136(75.7)	206	59.5
UTI symptoms					
Dysuria	Yes	58(27.6)	152(72.4)	210	60.7
	No	34(25)	102(75)	136	39.3
Frequency of urination	Yes	55(27.5)	155(72.5)	200	57.8
	No	37(25.3)	109(74.7)	146	42.2
Urine color	abnormal	64(39)	100(61)	164	47.4
	Normal	28(15.4)	154(84.6)	182	52.6
Flank pain	Yes	61(37)	104(63)	165	47.7
	No	31(17.1)	157(82.9)	181	52.8
CD4+ count	≤200 cells/mm^3	24(32.9)	49(67.1)	73	21.1
	201-350 cells/mm3	35(33.3)	70(66.7)	105	30.3
	351-500 cells/mm3	20(23.8)	64(762)	84	24.3
	≥500 cells/mm3	13(15.3)	72(84.7)	85	24.6

Prevalence of community-acquired UTI and isolated pathogens

The overall prevalence of community-acquired UTIs was 26.6% (92/346) of which 81.52% (75/92) were bacteria and 18.48% (17/346) were fungal candida. Among 75 bacterial isolates, the most frequent bacterium was E. *coli* which accounted for 21.33% (16/75) followed by *K. pneumoniae*, *enterococcus species*, and *P. aeruginosa*, accounting for 14.67% (11/75), 13.33% (10/75), and 12% (9/75), respectively. The least number of bacterial isolates were *Enterobacter cloacae*, which accounted for 5.33% (4 /75). Gram-positive bacteria accounted for only 32% (24/75) of the bacterial isolates, of which 41.67% (10/24) of the isolates were *Enterococcus species*. Of 17 candida isolates, 47.1% (8/17) were represented by *C. tropicalis* (**Fig.1)**

Antimicrobial susceptibility pattern of bacterial Uropathogens

Gram Negative bacteria

From the tested antimicrobial susceptibility patterns, about 52.9%, 52.9%, and 58.8% of the total isolated Gram-negative uropathogens were susceptible to ceftriaxone, ciprofloxacin, and gentamicin, respectively, whereas 51%, 72.5%, 72.5%, 84.3%, 60.8% and 60.8% of the isolates were resistant to amikacin, ampicillin, amoxicillin-clavulanic acid, trimethoprim-sulphsmethsoxazole, nitrofurantoin, and ceftazidime, respectively. About 62.5% of *E. coli* strains were sensitive to gentamycin. But, 87.5%, 87.5%, 81.3%, 75%, 62.5% and 56 % of bacterial isolates *E. coli* were resistant to tetracycline, Trimethoprim-sulphamethoxazole, amoxicillin-Clavulanic acid, ampicillin, nitrofurantoin, and ceftazidime, respectively. Similarly, 80% of ampicillin, 90.9% of trimethoprim-sulphamethoxazole, 90.9% of tetracycline and 63.6% of nitrofurantoin, and 63.6% ceftazidime, 54.5% was resistance to K. *pneumoniae*. However, (54.5%) of gentamicin, and 54.5% of both amikacin and ciprofloxacillin, were sensitive to *K. pneumoniae* **(Table 3).**

Table 3. Antimicrobial susceptibility pattern of Gram-negative bacterial isolates from Community-acquired UTI among HIV-positive patients at Dessie comprehensive specialized hospital, Dessie, Ethiopia

Etiologic agents	Pattern	Antimicrobial agents N (%)										
		AMP	AMC	AK	CRO	CIP	GN	CAZ	TE	CTX	SXT	F
E. coli (16)	S	4(25)	3(18.7)	9(56.2	9(56.2	8(50)	10(62.5)	7(43.8)	2(12.5)	8(50)	2(12.5)	6(37.5)
	R	12(75)	13(81.3)	7(43.8)	7(43.8)	8(50)	6(37.5)	9(56.2)	14(87.5)	8(50)	14(87.5)	10(62.5
K. pneumoniae (11)	S	3(27.3)	4(36.4)	5(45.5)	6(54.5)	5(45.5)	6(54.5)	4(36.4)	1(9.1)	6(54.5)	1(9.1)	4(36.4)
	R	8(72.7	7(63.6)	6(54.5)	5(45.5)	6(54.5)	5(45.5)	7(63.6)	10(90.9)	5(45.5)	10(90.9)	7(63.6)
P. mirabilis (5)	S	1(20)	2(40)	3(60)	3(60)	3(60)	1(20)	2(40)	1(20)	3(60)	1(20)	2(40)
	R	4(80)	3(60)	2(40)	2(40)	2(40)	4(80)	3(60)	4(80)	2(40)	4(80)	3(60)
P. aeruginosa (9)	S	3(33.3)	2(22.2)	5(55.6)	5(55.6)	5(55.6)	6(66.7)	4(44.4)	1(11.1)	4(44.4)	2(22.2)	3(33.3)
	R	6(66.7)	7(87.8)	4(44.4)	4(44.4)	4(44.4)	3(33.3)	5(55.6)	8(88.9)	5(55.6)	7(87.8)	6(66.7)
Enterobacter cloacae. (4)	S	1(25)	1(25)	2(50)	2(50)	2(50)	3(75)	1(25)	1(25)	2(50)	1(25)	2(50)
	R	3(75)	3(75)	2(50)	2(50)	2(50)	1(25)	3(75)	3(75)	2(50)	3(75)	2(500
Providencia spp (6)	S	2(33.3)	2(33.3)	3(50)	2(33.3)	4(66.7)	4(66.7)	2(33.3)	1(16.7)	3(50)	1(16.7)	3(50)
	R	4(66.7)	4(66.7)	3(50)	4(66.7)	2(33.3)	2(33.30	4(66.7)	5(83.3)	3(50)	5(83.3)	3(50)
Total (51)	S	14(27.5)	14(27.5)	25(49)	27(52.9)	27(52.9)	30(58.8)	20(39. 2)	7(13.7)	26(51)	8(15.7)	20(39.2)
	R	37(72.5)	37(72.5)	26(51)	24(47.1)	24(47.1)	21(41.2)	31(60. 8	44(86.3	25(49)	43(84.3)	31(60.8 0

S: sensitive; R: resistant; AMP: ampicillin; AMC: Amoxicillin-Clavulanic acid; CTX: cefotaxime; CAZ: Ceftazidime; F: Nitrofurantoin; CRO: ceftriaxone; CIP: ciprofloxacin; GN: gentamicin; AK: Amikacin; TE: tetracycline; SXT: Trimethoprim-sulphamethoxazole.

Gram Positive bacteria

From the total isolated Gram-positive uropathogens, 66.7% of isolates were susceptible to clindamycin. Whereas, 87.5%, 83.3%, 75%, 58.3%, and 58.3% of Gram-positive isolates were resistant to tetracycline, trimethoprim-sulfamethoxazole, penicillin, chloramphenicol, and nitrofurantoin, respectively. About *Enterococcus species* was sensitive for 70% and 60% of clindamycin ciprofloxacin, respectively. However, *Enterococcus species* showed more resistance to 90% for tetracycline, 80% for trimethoprim-sulphsmethsoxazole, and 70% for nitrofurantoin. Likewise, *coagulase-negative staphylococci* (CoNs) were resistant to most of the antimicrobials 87.5% for tetracycline, 87.5% for trimethoprim-sulphamethoxazole, and 75% for penicillin and chloramphenicol each **(Table 4).**

Table 4. Antimicrobial susceptibility pattern of Gram-positive bacterial isolates from community-acquired UTI among HIV-positive patients attending Dessie comprehensive specialized hospital, Dessie, Ethiopia

Bacterial isolates	Antimicrobial Agents N (%)										
		E N (%)	P N (%)	C N (%)	CTX N (%)	F	CL N (%)	CIP N (%)	SXT N (%)	FOX N (%)	TE N (%)
S. *aureus* (6)	S	4(66.7)	1(16.7)	3(50)	3(50)	2(33.3**)**	4(66.7)	4(66.7)	1(16.7)	3(50)	1(16.7)
	R	2(33.3)	5(83.3)	3(50)	3(50)	4(66.7)	2(33.3)	2(33.3)	5(83.3)	3(50)	5(83.3)
CoNS (8)	S	3(37.5)	2(25)	2(25)	3(37.5)	5(62.5)	5(62.5)	2(25)	1(12.5)	NT	1(12.5)
	R	5(62.5)	6(75)	6(75)	5(62.5)	3(37.5)	3(37.5)	6(75)	7(87.5)	NT	7(87.5)
Enterococcus species (10)	S	4(40)	3(30)	5(50)	6(60)	3(30)	7(70)	6(60)	2(20)	4(40)	1(10)
	R	6(60)	7(70)	5(50)	4(40)	7(70)	3(30)	4(40)	8(80)	6(60)	9(90)
Total (24)	S	11(45.83)	6(25)	10(41.7)	12(50)	10(41.7)	16(66.7)	12(50)	4(16.7)	7(43.75)	3(12.5)
	R	13(54.17)	18(75)	14(58.3)	12(50)	14(58.3)	8(33.3)	12(50)	20(83.3)	9(56.25)	21(87.5)

Key: **CL** = Clindamycin, **E** = Erythromycin, **C** = Chloramphenicol, **CIP** = Ciprofloxacin, **TE** = Tetracycline, **SXT** = Trimethoprim-Sulfamethoxazole, **CTX**= cefotaxime, **FOX=**Cefoxitin, **F:** nitrofurantoin**,** **P** =penicillin, **R =** Resistant, **FOX=**Cefoxitin **S =** Sensitive, **NT =** Not tested

Multi-drug resistance patterns of the bacterial isolates

Overall, 70 (93.3%) bacterial isolates were resistant to at least one antimicrobial agent, and 63 (84%) isolates were resistant to ≥2 antimicrobials. About 11 (14.7%) isolates had resistance to five or more antimicrobials. The overall prevalence of MDR bacteria (a bacterium that is simultaneously resistant to three or more antimicrobial categories) was 81.3%(61/75). About *13* (81.3%) of *E. coli*, 9(81.8%) of *K. pneumoniae, 8*(80%) of *Enterococcus species, 7 (77.8%) of P. aeruginosa*, and *CoNs* 7(87.5%) were the most frequently exhibited MDR **(Table 5).**

Table 5. Multi-drug resistance patterns of uropathogenic bacterial isolates among UTI symptomatic HIV-positive patients attending Dessie comprehensive specialized hospital, Dessie, Ethiopia (February 1, 2021- March 30, 2021).

Isolated organisms	Total	MDR to antimicrobials						
		R0 N (%)	R1 N (%)	R2 N (%)	R3 N (%)	R4 N (%)	≥R5 N (%)	MDR N (%)
E. coli	16	1(6.3)	1(6.3)	1(6.3)	4(25)	7(43.7)	2(12.5)	13(81.3)
K. pneumoniae	11	1(9.1)	1(9.1)	-	4(36.4)	2(18.2)	3(27.3)	9(81.8)
S. aureus	6	1(16.7)	-	-	3(50)	2(33.3)	-	5(83.3)
P. mirabilis	5	0	1(20)	-	2(40)	1(20)	1(20)	4(80)
CoNs	8	1(12.5)	-	-	4(50)	2(25)	1(12.5)	7(87.5)
P. aeruginosa	9	0	1(11.1)	1(11.1)	3(33.3)	2(22.2)	2(22.2)	7(77.8)
Enterobacter Cloacae	4	0	1(25)	-	1(25)	2(50)	-	3(75)
Enterococcus species	10	1(10)	1(10)	-	4(40)	3(30)	1(10)	8(80)
Providencia species	6	0	1(16.7)	-	2(33.3)	2(33.3)	1(16.7)	5(83.3)
Total	75	5(6.7)	7(9.3)	2(2.7)	27(36)	23(30.7)	11(14.7)	61(81.3)

R0=No antibiotic resistance category, R1=Resistance to one antibiotic category, R2=Resistance to two antibiotics category, R3=Resistance to three antibiotics category, R4=Resistance to four antibiotics category, R5=Resistance to five antibiotics category

Factors associated with CA-UTI

In the current study, bivariate analysis was performed on sociodemographic characteristics such as sex, family size, residence, occupations, immunization status, educational status, current marital status, current CD4 count, history of hospitalizations, catheterizations, and status of diabetics; and signs and symptoms of urinary tract infections. Multivariate analysis was done by including variables that had a p-value of ≤0.25 in bivariate analysis. After adjustment for age, sex, occupation, educational status, current CD4+ count (cells/mm3), history of previous hospitalization and diabetes status, history of catheterizations, and urgency of the urinary tract. Age >44year [AOR=1.56,

95%CI: 1.11-9.35, P=0.001], being female [AOR=3.29, 95%CI: 1.26-13.41, P=0.002], being daily labor [AOR=4.54, 95%CI: 1.41-21.71, P=0.003], being farmer [AOR=6.54, 95%CI: 1.21-23.51, P=0.004], participants unable to read and write [AOR=4.58, 95%CI: 1.21-13.58, P=0.001], HIV patients with CD4 count of ≤ 200 cells/mm^3 (AOR: 7.44; 95%CI: 1.64-27.21, P=0.001) and CD4 count of 201-350 cells/mm^3 (AOR: 4.55; 95%CI: 1.36-18.28, P=0.009), patients who had chronic diabetics (AOR: 4.80; 95%CI: 1.85-16.31, p=0.009), patients who had chronic diabetics (AOR: 4.80; 95%CI: 1.85-16.31, p=0.009), patients having a history of hospitalization (AOR: 6.27; 95%CI: 1.25-15.17, p=0.002) and patients who had the urgency of urinations (AOR: 6.51; 95%CI: 1.75-36.31, p=0.0025) were statistically significant associated risk factors for community acquired urinary tract infections (Table 6).

Table 6. Bivariate and multivariable analysis for variables associated with CA-UTI among HIV patients attending Dessie comprehensive specialized hospital, Dessie, Ethiopia (February 1, 2021- march 30, 2021).

Characteristics		Urine Culture Result		COR (95% CI)	p-value	AOR (95% CI)	P-value
		Positive n (%)	Negative n (%)				
Age in years	18–24	10(18.9)	43(81.1)	1	1		
	25–34	21(24.7)	64(75.3)	0.65(0.25-5.295)	0.984		
	35–44	30(25.9)	86(74.1)	0.985(0.154-14.038)	0.838		
	>44	31(33.7)	61(66.3)	1.56 (1.11-19.035)	0.012	1.56(1.11-9.35)	0.001
Sex	Male	42(25.3)	124(74.7)	1	1	1	1
	Female	50(27.8)	130(72.2)	1.79(1.1-3.655)	0.185	3.29(1.26- 13.41)	0.002
Residence	Urban	62(28.8)	153(71.2)	1	1		
	Rural	30(22.9)	101(77.1)	1.4(0.427-3.026)	0.89		
Occupation	House Wife	15(25.9)	43(74.1)	1	1		
	Civil Servant	10(22.2)	35(77.8)	0.95(0.41-5.34)	0.54		
	Self-Employee	9(18)	41(82)	1.25(0.36-13.54)	0.36		
	Driver	10(20.8)	38(79.2)	0.71(0.15-16.12)	0.65		
	Daily Labor	23(25.6)	67(74.4)	3.842(1.37-17.91)	0.17	4.54(1.41-21.71)	0.003
	Farmer	25(45.5)	30(55.5)	4.842(1.17-17.91)	0.18	6.54(1.21-23.51)	0.004
Educational status	Unable to read and write	42(36.5)	73(63.5)	3.85(1.76-12.61)	0.114	4.58(1.21-13.85)	0.001
	Primary	20(23.3)	66(76.7)	0.98 (0.928-12.398)	0.265		
	Secondary	22(24.2)	69(75.8)	1.95(0.43-12.22)	0.655		
	College/University	8(14.8)	46(85.2)	1	1	1	1
Current marital status	Married	45(37.2)	76(62.8)	1.8([0.61-16.86)	0.43		
	Unmarried	18(22.5)	62(77.5)	1.369(0.325-5.766)	0.668		
	Divorced	13(13.7)	82(86.3)	1.05(0.153 -7.270)	0.956		
	Widowed	16(32)	34(68)	1	1		
	≤200	24(32.9)	49(67.1)	5.29(1.39-18.51)	**0.018**	7.44(1.64-27.21)	**0.001**

Current CD4+ count (cells/mm^3)	201-350	35(33.3)	70(66.7)	4.18(1.55-17.10)	**0.024**	4.55(1.36-18.28)	**0.009**
	351-500	20(23.8)	64(762)	0.98(0.57-12.56)	0.705	0.99(0.89-12.24)	0.861
	≥501	13(15.3)	72(84.7)	1	1	1	1
History of hospitalization	Yes	35(30.4)	115(69.6)	1.98(1.78- 13.28)	0.181	6.27(1.25- 15.17)	0.002
	No	57(29.1)	139(70.9)	1	1	1	1
Use of cotrimoxazole as prophylaxis	Yes	40(26)	114(74)	1	1		
	No	54(29.1)	138(70.9)	0.953(0.455-1.995)	0.898		
Chronic diabetes	Yes	32((25.6)	89(74.4)	3.78(1.22-15.42)	0.016	4.80(1.85- 16.31)	0.009
	No	60(24.6)	184(75.4)	1	1	1	1
History of catheterization	Yes	42(30)	120(70)	1.794(1.53-16.03)	0.048	4.65(1.55- 26.31)	0.0012
	No	50(24.3)	136(75.7)	1	1		
Dysuria	Yes	58(27.6)	152(72.4)	0.753(0.368-1.979)	0.711		
	No	34(25.4)	100(74.6)	1	1		
Frequency of urination	Yes	55(27.5)	155(72.5)	0.629(0.395-1.741)	0.621		
	No	37(25.3)	109(74.7)	1	1		
Urgency	Yes	64(39)	100(61)	1.427(1.89-22.55)	0.039	6.51(1.75- 36.31)	0.0025
	No	28(15.4)	154(84.6)	1	1		
Flank pain	Yes	61(37)	104(63)	1.193(0.548-2.599)	0.657		
	No	31(17.1)	157(82.9)	1	1		

Discussion

A urinary tract infection (UTI) is defined as the invasion of the urinary tract by one or more uropathogenic bacteria species, which results in significant bacteriuria and the presence of symptoms such as dysuria, pain and burning during urinating, cloudy urine, and urine appears red, bright pink (34). In this study, the prevalence of urinary tract infection was 26.6% [95% CI: 21.5% -31.2%]. The current study is in line with study done in Hawassa (23.1%)(35) and Uganda (22.0%)(36). However, this finding was relatively higher than studies done in Jimma (13.7%) (37), Gojjam Ethiopia (20.6%)(38), Hawassa (4.9%) (39), Addis Ababa, Ethiopia (18.6%) and 11.3% (16, 40) and Harar, Ethiopia (18%) (41). But, it is lower than studies conducted in Nigeria (32.5%) (42), South Africa (48.7%) (43), Nepal (54.76%) and India (100%), and (49.15%) (44, 45). This variation might be due to differences in sample size, the degree of the immune status of the study participants, geographical variation, ART status, and socioeconomic conditions.

In the current study, urinary tract infection with fungal was 4.9% (17/346). Of the 17 fungal isolates, 47.1% (8/17) were represented by *C. tropicalis*. The study reported on *C. albicans*, *C. glabrata*, and *C. tropicalis* supported the current study with the report of fungal species (46). Studies showed that within hospital settings at least 11.3 % of community-acquired UTIs are caused by Candida species and Candidauria (47). In a study conducted in Addis Ababa, Candidauria represented 7% UTIs in which *C. albicans* was the leading cause of Candidauria and 81.1% candida species were isolated (22), and a study was done in two microorganisms Candida spp. *(Candida glabrata* and *Candida krusei*) (48).

Among 75 bacterial isolates, 21.33% (16/75) were *E. coli*, the most frequent bacterium, followed by *K. pneumoniae*, *enterococcus species*, and *P. aeruginosa*, accounting for 14.67% (11/75), 13.33% (10/75), and 12% (9/75), respectively. Similarly, *E. coli* was predominant in studies in different parts of Ethiopia and across different countries like, Bahirdar (38.1%)(41), Gondar, (56.1%)(49), and Jimma, (54.3%) (37) from Ethiopia, tertiary care hospital, India (41.7%)(16), Saudi Arabia (50) and Ethiopia (39). However, it was inconsistent with the findings reported in Ebony State, Nigeria (51) and India (44), they reported that *S. aureus* (45.3%) and *P. aeruginosa* (41.9%) and in Turkey the most common causative agent was *E. coli* (66.6%) followed by *K. pneumoniae* (16.6%) and others such as *Enterobacter spp*. (7.7%) (48) were the commonest urinary tract pathogens. This discrepancy might be due to differences in the test facility, strain variety, geographical variation and study group variations. The presence of unique structure that aids bacteria attachment to uroepithelial cells, allowing for multiplication and tissue invasion, could explain the predominance of *E. coli* (52, 53).

Even though no statistically significant association was observed in this study, we found that the bacteriuria was high among females (27.8%) than males (25.3%). This finding is in agreement with various studies conducted in Ethiopia (40, 41, 49), South Africa (43), and Nigeria (51). This could be explained by physiological and anatomical differences between males and females, such as males' shorter urethra, a decrease in normal vaginal flora, and females' less acidic vaginal surface pH (40).

In the current study, about 52.9%, 52.9%, and 58.8% of the total isolated Gram-negative uropathogens were susceptible to ceftriaxone, ciprofloxacin, and gentamicin, respectively, whereas, 51%, 72.5%, 72.3%, 84.3%, 60.8%, and 60.8% of the isolates were resistant to amikacin, ampicillin, amoxicillin-Clavulanic acid, and trimethoprim-sulphamethoxazole. The other study on urinary tract

bacterial and fungal isolates supported these current findings with frequency percentage sulphamethoxazole, nitrofurantoin, and ceftazidime, and most of the urinary bacterial isolates were highly sensitive to ceftazidime (95%), and ciprofloxacin (88% (54).

In this study, 87.5%, 87.5%, 81.3%, 75%, 62.5%, and 56 % of *E. coli* strains were resistant to tetracycline, trimethoprim-sulphamethoxazole, amoxicillin-Clavulanic acid, ampicillin, nitrofurantoin, and ceftazidime, respectively. Similarly, 80% of ampicillin, 90.9% of trimethoprim-sulphamethoxazole, 90.9% of tetracycline, 63.6% of nitrofurantoin, and 63.6% of ceftazidime, 54.5% was resistance to *K. pneumoniae*. There were also similar findings from studies conducted in Addis Ababa which indicated *K. pneumoniae* had the highest level of resistance against trimethoprim-sulphamethoxazole (86.4%) cefotaxime (86.4%), cefepime (85.4%), ceftazidime (85.4%), amoxicillin-clavulanic acid (85.4%), gentamicin (70.0%), and ciprofloxacin (50.5% (55), In Iran: trimethoprim-sulphamethoxazole (91.4%), ceftazidime (91.4%), and gentamicin (82.8%) (56); in Sierra Leone: ciprofloxacin (73.4%) and gentamicin (60%) (57), in Equatorial Guinea: trimethoprim-sulphamethoxazole (100%), amoxicillin-clavulanic acid (96.6%), gentamicin (86.2%) and ciprofloxacin (87.5% (58). The occurrence of high antibiotic resistance might be due to misuse and overuse of antibiotics, and poor infection control measures (59) This was comparable with the study done in Gondar, Ethiopia (49) and Harar, Ethiopia (41), *P. aeruginosa* was 100% resistant to ampicillin, cefotaxime, and gentamicin and trimethoprim-sulfamethoxazole and *P.mirabilis* 100% resistant to ampicillin. This is in agreement with a study done in Ethiopia (40).

Similarly, about 87.5%, 83.3%, 75%, 58.3%, and 58.3% of Gram-positive isolates were resistant to tetracycline, trimethoprim-sulfamethoxazole, penicillin, chloramphenicol, and nitrofurantoin, respectively. The dominant isolated Gram-positive *Enterococcus species* showed more resistance to 90% of tetracycline, 80% of trimethoprim-sulphamethoxazole, and 70% of nitrofurantoin. Likewise, *coagulase-negative staphylococci* were resistant to 87.5% of tetracycline and trimethoprim-sulphamethoxazole, and 75% of penicillin and chloramphenicol, respectively. This is in line with a study done in Harar, Ethiopia, where 57.1% of *S. aureus* were resistant to gentamicin, tetracycline, and cefoxitin (57.1%) (41). We also found that 85% of *Coagulase-negative staphylococcus spp*. were resistant to trimethoprim-sulphamethoxazole (85%) and over 60% were resistant to penicillin, gentamicin, and tetracycline.

Multidrug resistance has serious implications for the health outcome of HIV-infected patients (60). It is quite alarming to note that almost 61(81.3%) of currently isolated bacteria were found to be resistant to three or more antimicrobials. This was higher compared to previous findings reported in Harar, Ethiopia (46%) (41), Dessie (74.6%) (61), Gondar (68%) (62), and Nepal (64.04%)(63) and India (28%) (64). But it was lower than a report from Gondar, Ethiopia (95%) and 87.4%) (12, 49), Addis Ababa (100%) (40), Bahirdar (93.1%) (65), Nepal (96.84%) (63). These differences might be due to the irrational drug utilization habit of the communities or to the distribution of those sensitive and resistant strains of bacteria.

There was an intra-species difference in MDR level. The present study showed that the level of MDR in *E. coli* 13 (81.3%), *K. pneumoniae* 9(81.8%), *Enterococcus species* 8(80%), *P. aeruginosa* 7 (77.8%), and CoNs 7(87.5%). This was comparable with a study conducted in Addis Ababa *K. pneumoniae* (83.5%) and *E. coli* (66.2%) (66), in Equatorial Guinea *E. coli* (74.4%) (58); in Sierra Leone *K. pneumoniae* (73.3%) and *E. coli* (61.5%) (57); in Gondar, *K. pneumoniae* was (95.6%) and *E. coli* was (92.9%) (12); in Khartoum-Sudan *E. coli* (92.2%)(67); and in Equatorial Guinea in *K. pneumoniae* (91.7%) (58). The MDR level among E. coli (50.2%) in Dessie is lower than in our study(61). The difference in MDR levels between *K. pneumoniae* and *E. coli* in our study might be most K. pneumoniae were isolated from blood specimens that were collected from inpatients in the hospitals.

In this study, there was no statistically significant association between significant bacteriuria and patients residence, marital status, dysuria, frequency of urination, flank pain, and use of co-trimoxazole. However, age, gender, occupation, educational status, current CD4+ count (cells/mm3), previous hospitalization and diabetes status, history of catheterizations, and urinary tract urgency had a statistically significant association with significant bacteriuria and Candidauria. Similar findings were reported from Jimma, Addis Ababa, and Harar (37, 40, 41). Furthermore, age groups >44year [AOR=1.56, 95%CI: 1.11-9.35, P=0.001], being female [AOR=3.29, 95%CI: 1.26-13.41, P=0.002], being daily labor [AOR=4.54, 95%CI: 1.41-21.71, P=0.003], being farmer [AOR=6.54, 95%CI: 1.21-23.51, P=0.004], participants unable to read and write [AOR=4.58, 95%CI: 1.21-13.58, P=0.001], HIV patients with CD4 count of ≤ 200 cells/mm3 (AOR: 7.44; 95%CI: 1.64-27.21, P=0.001) and CD4 count of 201-350 cells/mm3 (AOR: 4.55; 95%CI: 1.36-18.28, P=0.009), patients who had chronic diabetics (AOR: 4.80; 95%CI: 1.85-16.31, p=0.009), patients having a history of hospitalization (AOR: 6.27; 95%CI: 1.25-15.17, p=0.002) and patients

who had the urgency of urination (AOR: 6.51; 95%CI: 1.75-36.31, p=0.0025). Studies conducted in Nepal support our findings with a significant risk factor for community-acquired urinary tract infections in patients with indwelling urinary catheters (38.3%), Diabetes-mellitus (36%), malignancies (5.2%), and urinary incontinence (11.7%). Urinary catheters are a proven source of infection, especially when they are kept for longer periods (47). Similarly, research finding was supported by studies conducted elsewhere [26, 22, 27, and 29]. The explanation for the inverse relationship between UTI and a CD4+ count is unknown; it is probably due to the impaired immunity at a declining CD4+ count that makes it easier for bacterial pathogens to adhere to the urinary epithelium.

Limitations of the study

The study was a single hospital-based study and might not represent all HIV-infected patients and did not include a control group due to budget constraints. We did not attempt to identify other causative agents (anaerobic bacteria, and fungus) that would have made a significant contribution to a true prevalence of UTI in HIV-positive patients.

Conclusions and Recommendations

The symptomatic community's acquired UTI is high, with an overall prevalence of 26.6% (92/346). Of the isolated pathogens, 81.52% (75/92) were bacterial pathogens, while 18.48% (17/92) were fungal pathogens. Among bacterial pathogens, *E. coli*, the most frequent bacterium, followed by *K. pneumoniae*, *enterococcus species*, and *P. aeruginosa*, accounting for, respectively. The overall prevalence of MDR bacteria was 61(81.3%). About 13 (81.3%) of *E. coli*, 9(81.8%) of *K. pneumoniae*, 8(80%) of *Enterococcus species*, 7 (77.8%) of *P. aeruginosa*, and *CoNs* 7(87.5%). Most Gram-negative and Gram-positive isolates were resistant to tetracycline, trimethoprim-sulfamethoxazole, penicillin, chloramphenicol, and nitrofurantoin, respectively. However, uropathogenic were susceptible to amikacin, it was more effective against these pathogens. Patients who had low CD4 counts, previous hospitalization, diabetes status, and having history of catheterizations than their counterparts were more at risk of developing bacterial urinary tract infections. Management of UTI among symptomatic HIV patients should be supported by laboratory results of urine culture. Regular monitoring of antimicrobial resistance patterns should be undertaken by healthcare providers. Individuals at risk for bacterial-associated UTI should have good adherence to ART and their CD4 levels should be monitored regularly. Health information dissemination should be given about UTIs and the habit of drug use for HIV-positive patients.

List of abbreviations and acronyms

AIDS; Acquired immune deficiency syndrome, ARTC; Attending antiretroviral therapy clinic, ART; Antiretroviral therapy, ATCC; American Type Culture collections, BA; Blood agar, CFU; Colony Forming Unit, CLED; Cysteine Lactose Electrolyte Deficient, CONS; Coagulase-negative Staphylococci, HAART; Highly active antiretroviral therapy, HIV; Human Immunodeficiency Virus, MACA; MacConkey agar, MDR; Multi-Drug Resistant, MSU; Midstream urine, OIS; Opportunistic infections, UTI; Urinary Tract Infection, VCT Voluntary counseling, and Testing.

Data availability statement

Data supporting the conclusions of this article are within the manuscript and are available on reasonable request from the principal investigators due to ethical reasons.

Funding

The authors were no obtained specific fundings for this work. only internal material support was obtained from Wollo University. The funders had no role in study design, data collection, and analysis, the decision to publish, or the preparation of the manuscript.

Competing interests

The authors declare that they have no competing interests.

Authors' contributions

Conceived and designed the experiments: Mihret Tilahun, Abdurrahman Seid, Bekele Sharew, Tewodros Dessie, Habtamu Mekonen; Performed the experiments: Mihret Tilahun, Abdurrahman Seid, Agumas Shibabaw; Analyzed the data: Mihret Tilahun, Abdurrahman Seid, Daniel G/tsadik, Mesfin Fiseha, Melkam Tesfaye, Alemu Gedefie, Melaku Ashagrie Belete; Wrote and edited the manuscript: Mihret Tilahun, Abdurrahman Seid, Bekele Sharew, Alemu Gedefie, Habtu Debash, Agumas Shibabaw, Melaku Ashagrie Belete, and Habtamu Mekonnen. All authors read and approved the final manuscript.

Acknowledgment

The authors would like to acknowledge the Department of Medical Laboratory Science, College of Medicine and Health Sciences, Wollo University for providing the laboratory setup and facilities to conduct the experiments. Dessie comprehensive specialized hospital and all study participants are gratefully acknowledged for their kind cooperation during data collection.

References

1. Syed B, Salahuddin N, Ishtiaq H. Etiologies and microbiological profile of complicated urinary tract infections, among patients admitted in a tertiary care hospital. *Infec Dis J Pakistan*. 2017;26:59-64.
2. Kiranmala K, Johnson R, Savio J, Idiculla J. Microbiologic profile and clinical practices in urinary tract infections in a tertiary care center in Southern India. *Journal of Family Medicine and Primary Care*. 2019;8(9):2888.
3. Nerurkar A, Solanky P, Naik SS. Bacterial pathogens in urinary tract infection and antibiotic susceptibility pattern. *Journal of Pharmaceutical and Biomedical Sciences*. 2012;21(21).
4. Ade-Ojo I, Oluyege A, Adegun P, Akintayo A, Aduloju O, Olofinbiyi B. Prevalence and antimicrobial susceptibility of asymptomatic significant bacteriuria among new antenatal enrollees in Southwest Nigeria. *International Research Journal of Microbiology*. 2013;4(8):197-203.
5. Schmider J, Bühler N, Mkwatta H, Lechleiter A, Mlaganile T, Utzinger J, et al. Microbiological characterization of community-acquired urinary tract infections in Bagamoyo, Tanzania: *A prospective study*. *Tropical Medicine and Infectious Disease*. 2022;7(6):100.
6. Tegegne KD, Wagaw GB, Gebeyehu NA, Yirdaw LT, Shewangashaw NE, Kassaw MW. Prevalence of urinary tract infections and risk factors among diabetic patients in Ethiopia, a systematic review and meta-analysis. *PloS one*. 2023;18(1):e0278028.
7. Birhanu MY, Habtegiorgis SD, Gietaneh W, Alemu S, Tsegaye TB, Bekele GM, et al. The magnitude and associated factors of urinary tract infections among adults living with HIV in Ethiopia. Systematic review and meta-analysis. *Plos one*. 2022;17(4):e0264732.
8. Çoban B, Ülkü N, Kaplan H, Topal B, Erdoğan H, Baskın E. Five-year assessment of causative agents and antibiotic resistances in urinary tract infections. *Turkish Archives of Pediatrics/Türk Pediatri Arşivi*. 2014;49(2):124.
9. Flores-Mireles AL, Walker JN, Caparon M, Hultgren SJ. Urinary tract infections: epidemiology, mechanisms of infection and treatment options. *Nature reviews microbiology*. 2015;13(5):269-84.
10. Odabasi Z, Mert A. Candida urinary tract infections in adults. *World journal of urology*. 2020;38(11):2699-707.
11. Behzadi P, Behzadi E, Ranjbar R. Urinary tract infections and Candida albicans. *Central European journal of urology*. 2015;68(1):96-101.
12. Eshetie S, Unakal C, Gelaw A, Ayelign B, Endris M, Moges F. Multidrug-resistant and carbapenemase-producing Enterobacteriaceae among patients with urinary tract infection at

Referral Hospital, Northwest Ethiopia. *Antimicrobial resistance and infection control*. 2015;4(1):1-8.

13. Ahmed SS, Shariq A, Alsalloom AA, Babikir IH, Alhomoud BN. Uropathogens and their antimicrobial resistance patterns: Relationship with urinary tract infections. *International Journal of Health Sciences*. 2019;13(2):48.
14. Prakash D, Saxena RS. Distribution and antimicrobial susceptibility pattern of bacterial pathogens causing urinary tract infection in the urban community of Meerut city, India. *International scholarly research notices*. 2013;2013.
15. Haile Hantalo A, Haile Taassaw K, Solomon Bisetegen F, Woldeamanuel Mulate Y. Isolation and Antibiotic Susceptibility Pattern of Bacterial Uropathogens and Associated Factors Among Adult People Living with HIV/AIDS Attending the HIV Center at Wolaita Sodo University Teaching Referral Hospital, South Ethiopia. *HIV/AIDS-Research and Palliative Care*. 2020:799-808.
16. Fenta G, Legese M, Weldearegay G. Bacteriuria and their antibiotic susceptibility patterns among people living with HIV attending Tikur Anbessa Specialized and Zewditu Memorial Hospital ART Clinics, Addis Ababa, Ethiopia. *J Bacteriol Parasitol.* 2016;7(05).
17. Yismaw G, Asrat D, Woldeamanuel Y, Unakal CG. Urinary tract infection: bacterial etiologies, drug resistance profile, and associated risk factors in diabetic patients attending Gondar University Hospital, Gondar, Ethiopia. *European Journal of Experimental Biology*. 2012;2(4):889-98.
18. Kibret M, Abera B. Prevalence and antibiogram of bacterial isolates from urinary tract infections at Dessie Health Research Laboratory, Ethiopia. *Asian Pacific journal of tropical biomedicine*. 2014;4(2):164-8.
19. Paschke AA, Zaoutis T, Conway PH, Xie D, Keren R. Previous antimicrobial exposure is associated with drug-resistant urinary tract infections in children. *Pediatrics*. 2010;125(4):664-72.
20. Frank-Peterside N, Okerentugba P, Nwodo C, Okonko I. Prevalence of bacterial uropathogens in a cohort of HIV-positive males in Port Harcourt, Nigeria. Cancer Biology. 2013;3(4):12-7.
21. Skrzat-Klapaczyńska A, Matłosz B, Bednarska A, Paciorek M, Firląg-Burkacka E, Horban A, et al. Factors associated with urinary tract infections among HIV-1 infected patients. *PLOS one*. 2018;13(1):e0190564.
22. Bitew A, Zena N, Abdeta A. Bacterial and fungal profile, antibiotic susceptibility patterns of bacterial pathogens and associated risk factors of urinary tract infection among symptomatic

pediatrics patients attending St. Paul's Hospital Millennium Medical College: a cross-sectional study. *Infection and drug resistance*. 2022;15:1613.
23. Foxman B. Urinary tract infection syndromes: occurrence, recurrence, bacteriology, risk factors, and disease burden. *Infectious Disease Clinics*. 2014;28(1):1-13.
24. Lee Y-C, Hsiao C-Y, Hung M-C, Hung S-C, Wang H-P, Huang Y-J, et al. Bacteremic urinary tract infection caused by multidrug-resistant Enterobacteriaceae are associated with severe sepsis at admission: implication for empirical therapy. *Medicine*. 2016;95(20).
25. Islam MA, Islam MR, Khan R, Amin MB, Rahman M, Hossain MI, et al. Prevalence, etiology and antibiotic resistance patterns of community-acquired urinary tract infections in Dhaka, Bangladesh. *Plos one*. 2022;17(9):e0274423.
26. Haider MZ, Annamaraju P. Bladder Catheterization. StatPearls [Internet]: StatPearls Publishing; 2022.
27. Magiorakos A-P, Srinivasan A, Carey RB, Carmeli Y, Falagas M, Giske C, et al. Multidrug-resistant, extensively drug-resistant and pan drug-resistant bacteria: an international expert proposal for interim standard definitions for acquired resistance. *Clinical microbiology and infection.* 2012;18(3):268-81.
28. Bigwan E, Wakjissa F. Prevalence of urinary tract infections among HIV patients attending a non-governmental health facility in Jos, Plateau State, Nigeria. *International Journal of Biomedical and Advance Research.* 2013;4(8):528-33.
29. Murray PR, Rosenthal KS, Pfaller MA. Medical microbiology E-book: *Elsevier Health Sciences*; 2020.
30. Pezzlo M. Laboratory diagnosis of urinary tract infections: guidelines, challenges, and innovations. *Clinical Microbiology Newsletter*. 2014;36(12):87-93.
31. Bullock NO, Aslanzadeh J. Biochemical profile-based microbial identification systems. Advanced techniques in diagnostic microbiology: Springer; 2013. p. 87-121.
32. Malik UM, Khan AB, Satti ML. Comparative evaluation of CHROMagar and API 20C AUX in isolation and identification of Candida species. *Journal of Islamic International Medical College* (JIIMC). 2018;13(2):85-90.
33. Institute CLS. CLSI Performance standard for antimicrobial susceptibility testing, M100. 2021.

34. Behzadi P, Urbán E, Matuz M, Benkő R, Gajdács M. The role of gram-negative bacteria in urinary tract infections: current concepts and therapeutic options. *Advances in Microbiology, Infectious Diseases, and Public Health.* 2020:35-69.
35. Nigussie D, Amsalu A. Prevalence of uropathogenic and their antibiotic resistance pattern among diabetic patients. *Turkish Journal of Urology*. 2017;43(1):85.
36. Nabaigwa BI, Mwambi B, Okiria J, Oyet C. Common uropathogens among diabetic patients with urinary tract infection at Jinja Regional Referral Hospital, Uganda. *African Journal of Laboratory Medicine*. 2018;7(1):1-3.
37. Debalke S, Cheneke W, Tassew H, Awol M. Urinary tract infection among antiretroviral therapy users and nonusers in Jimma University Specialized Hospital, Jimma, Ethiopia. *International journal of microbiology*. 2014;2014.
38. Girma A, Aemiro A. The Bacterial Profile and Antimicrobial Susceptibility Patterns of Urinary Tract Infection Patients at Pawe General Hospital, Northwest Ethiopia. *Scientifica.* 2022;2022.
39. Tessema NN, Ali MM, Zenebe MH. Bacterial associated urinary tract infection, risk factors, and drug susceptibility profile among adult people living with HIV at Haswassa University Comprehensive Specialized Hospital, Hawassa, Southern Ethiopia. *Scientific Reports*. 2020;10(1):1-9.
40. Getu Y, Ali I, Lema T, Belay H, Yeshetela B. Bacteriuria and antimicrobial susceptibility pattern among HIV patients attending ALERT Center, Addis Ababa, Ethiopia. *Am J Health Res*. 2017;5(3):76-82.
41. Marami D, Balakrishnan S, Seyoum B. Prevalence, Antimicrobial Susceptibility Pattern of Bacterial Isolates, and Associated Factors of Urinary Tract Infections among HIV- Positive Patients at Hiwot Fana Specialized University Hospital, Eastern Ethiopia. C*anadian Journal of Infectious Diseases and Medical Microbiology*. 2019;2019.
42. Okechukwu A, Thairu Y. Bacteria urinary tract infection in HIV-infected children and adolescents in Abuja, Nigeria: a cross-sectional study. *African Journal of Clinical and Experimental Microbiology*. 2019;20(4):306-14.
43. Iweriebor B, Obi C, Akinyemi O, Ramalivhana N, Hattori T, Okoh A. uropathogens isolated from HIV-infected patients from Limpopo Province, South Africa. *African Journal of Biotechnology*. 2012;11(46):10598-604.

44. Xavier TF, Auxilia A, Kannan M. Isolation and characterization of UTI pathogens from HIV positive patients of Karur District, Tamil Nadu, India. *Int J Curr Microbiol Appl Sci.* 2015;4(1):558-63.
45. Sharma V, Gupta V, Mittal M. Prevalence of uropathogens in diabetic patients and their antimicrobial susceptibility pattern. *Natl J lab Med*. 2012;1(1):26-8.
46. Achkar JM, Fries BC. Candida infections of the genitourinary tract. Clinical microbiology reviews. 2010;23(2):253-73.
47. Aadil Rafeeq Rather1* ANKaTM. Profile of Community Acquired Urinary Tract Infections at a Teriary Care Hospital. Volume 2 Issue 11 November 2019. 2019.
48. Erdem I, Ali RK, Ardic E, Omar SE, Mutlu R, Topkaya AE. Community-acquired lower urinary tract infections: Etiology, antimicrobial resistance, and treatment results in female patients. J*ournal of global infectious diseases*. 2018;10(3):129.
49. Alemu A, Dagnew M, Alem M, Gizachew M. Uropathogenic bacterial isolates and their antimicrobial susceptibility patterns among HIV/AIDS patients attending Gondar University Specialized Hospital Gondar, Northwest Ethiopia. 2013.
50. Biradar SK, Doddamani P. Prevalence and antibiogram of uropathogens in a tertiary care hospital. *World J Pharmaceut Res*. 2013;2:1534-43.
51. Ifeanyichukwu I, Emmanuel N, Chika E, Anthonia O, Esther U-I, Ngozi A, et al. Frequency and antibiogram of uropathogens isolated from urine samples of HIV-infected patients on antiretroviral therapy. *American Journal of BioScience*. 2013;1(3):50-3.
52. Ejerssa AW, Gadisa DA, Orjino TA. Prevalence of bacterial uropathogens and their antimicrobial susceptibility patterns among pregnant women in Eastern Ethiopia: hospital-based cross-sectional study. *BMC women's health*. 2021;21(1):1-15.
53. Fierer J, Looney D, Kok M, Pechère J-C. Nature and pathogenicity of micro-organisms. Infectious diseases. 2010:3.
54. Nwadioha S, Nwokedi E, Ikeh I, Egesie J, Kashibu E. Antibiotic susceptibility pattern of uropathogenic bacterial isolates from AIDS patients in a Nigerian tertiary hospital. *J Med Med Sci*. 2010;1(11):530-4.
55. Teklu DS, Negeri AA, Legese MH, Bedada TL, Woldemariam HK, Tullu KD. Extended-spectrum beta-lactamase production and multi-drug resistance among Enterobacteriaceae isolated in Addis Ababa, Ethiopia. *Antimicrobial Resistance & Infection Control.* 2019;8:1-12.

56. Mansouri S, Abbasi S. Prevalence of multiple drug resistant clinical isolates of extended-spectrum beta-lactamase-producing Enterobacteriaceae in Southeast Iran. 2010.
57. Leski TA, Taitt CR, Bangura U, Stockelman MG, Ansumana R, Cooper WH, et al. High prevalence of multidrug-resistant Enterobacteriaceae isolated from outpatient urine samples but not the hospital environment in Bo, Sierra Leone. *BMC infectious diseases.* 2016;16(1):1-9.
58. Shatalov A. Prevalence and antibiotic resistance pattern of Escherichia coli and Klebsiella pneumoniae in urine tract infections at the La Paz Medical Center, Malabo, Equatorial Guinea. *Open Journal of Medical Microbiology*. 2015;5(04):177.
59. Adler A, Katz DE, Marchaim D. The continuing plague of extended-spectrum β-lactamase–producing Enterobacteriaceae infections. *Infectious Disease Clinics.* 2016;30(2):347-75.
60. Kim H-R, Hwang SS, Kim HJ, Lee SM, Yoo C-G, Kim YW, et al. Impact of extensive drug resistance on treatment outcomes in non-HIV-infected patients with multidrug-resistant tuberculosis. *Clinical Infectious Diseases.* 2007;45(10):1290-5.
61. Kibret M, Abera B. Antimicrobial susceptibility patterns of E. coli from clinical sources in northeast Ethiopia. *African health sciences*. 2011;11:40-5.
62. Moges F, Mengistu G, Genetu A. Multiple drug resistance in Urinary pathogens at Gondar College of Medical science hospital, Ethiopia. East African medical journal. 2002;79(8):415-20.
63. Yadav KK, Adhikari N, Khadka R, Pant AD, Shah B. Multidrug-resistant Enterobacteriaceae and extended-spectrum β-lactamase producing Escherichia coli: a cross-sectional study in National Kidney Center, Nepal. *Antimicrobial resistance and infection control*. 2015;4(1):1-7.
64. Murugesh K, Deepa S, Ravindranath C, Venkatesha D. Multi-drug resistant uropathogens in HIV: Are they a threat to the community? International Journal of Scientific Study. 2014;2(3):38-42.
65. Biadglegne F, Abera B. Antimicrobial resistance of bacterial isolates from urinary tract infections at Felge Hiwot Referral Hospital, Ethiopia. *The Ethiopian Journal of Health Development.* 2009;23(3).
66. Teklu DS, Negeri AA, Legese MH, Bedada TL, Woldemariam HK, Tullu KD. Extended-spectrum beta-lactamase production and multi-drug resistance among Enterobacteriaceae isolated in Addis Ababa, Ethiopia. *Antimicrobial Resistance & Infection Control*. 2019;8(1):1-12.
67. Ibrahim M, Bilal N, Hamid M. Increased multi-drug resistant Escherichia coli from hospitals in Khartoum state, Sudan. *African health sciences.* 2012;12(3):368-75.

Figure 1 legends

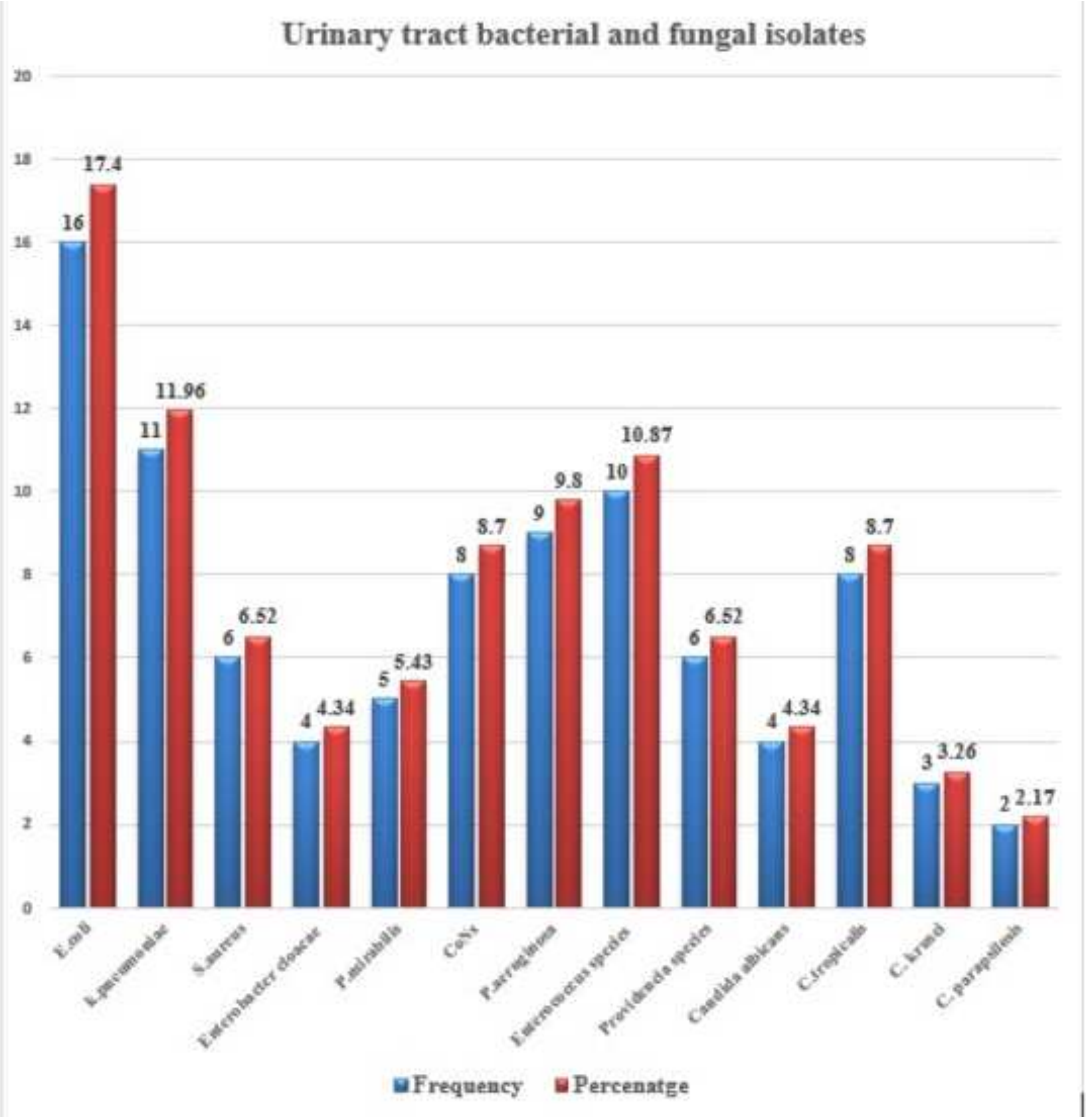
Urinary tract bacterial and fungal isolates
20
18
16
14
12
10
8
6
4
2
0
16
17.4
11
11.96
6
6.52
4
4.34
5
5.43
8
8.7
9
9.8
10
10.87
6
6.52
4
4.34
8
8.7
3
3.26
2
2.17
E.coli
K.pneumoniae
S.aureus
Enterobacter cloacae
P.mirabilis
CoNs
P.aeruginosa
Enterococcus species
Providencia species
Candida albicans
C.tropicalis
C. parapsilosis
Frequency
Percenatge

Click here to access/download
Supporting Information
Ethical clearance.pdf

Uropathogens: multidrug resistance and risk factors of community-acquired UTI among HIV patients attending antiretroviral therapy clinics of Northeast Ethiopia

Mihret Tilahun[1*], Mesfin Fiseha[1], Mihreteab Alebachew[1], Alemu Gedefie[1], Endris Ebrahim[1], Melkam Tesfaye[1], Melaku Ashagrie Belete[1], Abdurahaman Seid [1], Daniel Gebretsadik [1], Ermiyas Alemayehu[1], Wondmagegn Demsiss[1], Bekele Sharew[1], Agumas Shibabaw[1], Habtamu Mekonnen[2], Tewodros Dessie [2]

[1]Department of Medical Laboratory Sciences, College of Medicine and Health Science, Wollo University, Dessie, Ethiopia

[2] Amhara Public Health Institute Dessie branch

Authors' information:

Mihret Tilahun (MSc in Medical Microbiology): tilahunmihret21@gmail.com

Mesfin Fiseha (MSc in hematology and immunohematology): mesfinfiseha40@gmail.com

Alemu Gedefie (MSc in Medical Microbiology): alemugedefie@gmail.com

Mihreteab Alebachew (MSc in medical parasitology and vector control): mihreteab71@gmail.com

Endris Ebrahim (MSc in immunology): endris.index@gmail.com

Melkam Tesfaye (Ph.D. fellow in infectious diseases): Melkam66@gmail.com

Melaku Ashagrie Belete (MSc in Medical Microbiology): melakuashagrie@gmail.com

Abdurahaman Seid (MSc., Asst Prof in Medical Microbiology): gechdk2000@yahoo.com

Daniel Gebretsadik (MSc., Asst Prof in Tropical and infectious diseases): gebretsadikd@gmail.com

Ermiyas Alemayehu ((MSc in clinical chemistry): ermiyas0009@gmail.com

Wondmagegn Demsiss (MSc., Asst Prof in Medical Microbiology): *wonde1980@gmail.com*

Bekele Sharew (Ph.D., Asst Professor in Medical Microbiology): bsharew@gmail.com

Agumas Shibabaw (Ph.D., Asst Professor in Medical Microbiology): agumas2000@gmail.com

Habtamu Mekonnen (MSc in Medical Microbiology): habtamumeko36@gmial.com

Tewodros Dessie (MSc in Medical Microbiology): tedydess21@gmial.com

Corresponding author

Mihret Tilahun (MSc.)

Department of Medical Laboratory Science

College of Medicine and Health Sciences,
Wollo University, Dessie, Ethiopia
Telephone: +251- 920988307
Fax: +251 333115250
PO.BOX: 1145
Email: tilahunmihret21@gmail.com

Abstract

Background: Urinary tract infections are the common bacterial and fungal infections in humans, occurring both in the community and in immunocompromised patients in health care settings. UTI has significant health impact on HIV-infected patients. Drug resistance pathogens is now widespread and poses a serious clinical risk and causes urinary tract infection. This study investigated uropathogens, multidrug resistance pattern of bacteria and associated factors of community-acquired urinary tract infection among HIV positive patients attending antiretroviral therapy clinic of Northeast Ethiopia from February 1, 2021, to March 30, 2021.

Methods: Institutional based cross-sectional study was conducted at Dessie comprehensive specialized Hospital. Socio-demographic and clinical data were collected by using structured questionnaire from HIV patients suspected of community-acquired urinary tract infection. About 10 ml of clean-catch midstream urine was collected and inoculated into Blood agar, MacConkey, and Cysteine lactose electrolyte deficient media. Gram stain and biochemical tests were performed to identify isolates and antimicrobial susceptibility pattern was performed on disc diffusion techniques. Data were entered and analyzed using SPSS version-25. Both bivariate and multivariable logistic regression analysis was performed and a P value of < 0.05 with an adjusted odds ratio with their 95% confidence interval (CI) was used as statistically significant associations.

Results: From the total 346 study participants, 92 (26.6%) were culture positive and 75 (81.52%) were bacterial and 17 (18.48%) were fungal pathogens. From a total of 75 bacteria isolates 51(68%) were Gram-negative bacteria and the most commonly isolated bacteria were *E. coli* 16 (21.33%) followed by *K. pneumoniae* 11(14.67%) and *enterococcus species* 10(10.87). Of the 17 fungal isolates of fungi, 8(47.1%) were represented *by C. tropicalis*. The overall prevalence of Multi-Drug Resistance bacteria was 61(81.3%). About 13 (81.3%) of *E. coli*, 9(81.8%) of *K. pneumoniae*, 8(80%) of *Enterococcus* species, 7 (77.8%) of *P. aeruginosa*, and *CoNs* 7(87.5%) were the most frequently exhibited multi-drug Resistance. Amikacin, and gentamicin were effective against Gram-negative uropathogens. Participants with age >44year, being female, being daily labor, being farmer, unable to read and write, patients with CD4 count of ≤ 200 cells/mm^3 and CD4 count of 201-350 cells/mm^3, who had chronic diabetics, patients having a history of

hospitalization and who had urgency of urinations were statistically significant association with significant urinary tract infections.

Conclusion: The burden of community acquired urinary tract infections among HIV patients is alarmingly increased. Therefore, behavior change communications might be considered for promoting the health status of HIV patients. Moreover, CD4 level monitoring and therapeutics selection based on microbiological culture are quite advisable for management of urinary tract infections of HIV patients

Keywords: Bacteriuria, Candidauria, Uropathogens, community-acquired UTI, Multidrug resistance, Ethiopia

Introduction

Urinary tract infection (UTI) is an infection of the urinary tract, including the kidneys, ureters and bladder, urethra, and accessory structures that collect, store and release of urine from the body. Urinary tract infection occurs when microorganisms, typically bacteria from the digestive tract, entering to the urethral opening and begin to multiply (1-3).

Globally, the burden of UTI is estimated to be 150 million cases every year (4). Based on previous epidemiological studies from Tanzania and elsewhere in sub-Saharan Africa, urinary tract infections were estimated from 35–45% cases (5). In Ethiopia systematic review and meta-analysis indicated that, the pooled prevalence of urinary tract infection was 15.97%(6) and urinary tract infections in HIV patients in Ethiopia were 12.8% (7).

Urinary tract infections are caused by bacteria, as well as certain fungi and *Escherichia coli (E. coli)* is the most common cause of both uncomplicated and complicated UTIs, followed by *Klebsiella pneumonia (K.pneumoniae)*, *Staphylococcus saprophyticus (S.saprophyticus)*, *Enterococcus faecalis (E. faecalis)*, *group B streptococcus(GBS)*, *Proteus mirabilis (P.mirabilis)*, *Pseudomonas aeruginosa* (*P. aeruginosa)*, *Staphylococcus aureus (S.aureus)*, and *Candida* spp (8). Evidence showed that *Escherichia coli* is the most common causative agent for complicated UTIs. The order of prevalence for causative agents is *Enterococcus* spp., *K. pneumoniae*, *Candida* spp., *S. aureus*, *P. mirabilis*, *P. aeruginosa*, and GBS (9). Candidauria is the presence of *Candida spp.* in the urine, such as *C. albicans*. Patients with cystitis, epididymorchitis, prostatitis, pyelonephritis, and renal candidiasis experience symptomatic candidiasis (10). C. albicans is one of the most common fungi that can cause Candidauria (20% of nosocomial infections) (11).

In developing countries, the emergence of antibiotic resistance in the treatment of urinary tract infections is a major public health issue (12). Recently, the most common pathogens of UTIs developed resistance to most antimicrobial agents (13). *E. coli*, *Klebsiella species* and *Proteus species* had high resistance rates to amoxicillin and tetracycline of 80.1%–90.0% and low sensitivity rates of 0–25% to nitrofurantoin, ciprofloxacin, and gentamicin. Different evidences across the world and Africa including Ethiopia showed that more than 50% of uropathogenic bacteria were developed multidrug resistance (MDR). About 70.0% of bacteria from India (14), and in Ethiopia 79.3% from Wolayta Sodo Ethiopia (15), 78.4% from Addis Ababa (16) and 91.7% from Gondar (17) developed MDR. The antimicrobial test analysis showed that 152 (47.85%) of the isolates were resistant to two or more antimicrobials (18). Majority of treatments are initiated empirically,

knowledge of the organisms, their epidemiological characteristics, adequate treatment, control of these conditions necessitate and antibacterial susceptibility is required (19).

Urinary tract infections cause increased mortality, disability, hospitalization, economic loss in the HIV population, renal failure and hypertension. It is predisposed to nephritic complications such as urinary tract obstruction, which causes renal colic, pelvic-ureteric junction obstruction, benign prostate hyperplasia, urethral strictures, bladder dysfunction, and urinary stasis (20).

A systematic review indicated that being female, having a history of recurrent UTI, having low socioeconomic status, having diabetes mellitus, genitourinary tract abnormalities, having a low CD4 count, history of catheterization, history of hospitalization and chronic diabetics status were independent factors for the occurrence of urinary tract infection (7, 15). Another study on factors associated with urinary tract infections among HIV infected patients with low CD4 count, chronic hepatitis, and being female (21).

Despite numerous studies on bacterial UTI, antimicrobial susceptibility testing, and HIV infection-related factors in developed countries, there is little data on the burden of bacterial UTI and antimicrobial susceptibility patterns among UTI-symptomatic HIV-infected patients in resource-limited settings, including Ethiopia in general, and no study was done in the study area in particular. As a result, the purpose of this study was to determine the uropathogens, multidrug resistance pattern of bacteria and associated factors of community-acquired urinary tract infection among HIV positive patients attending antiretroviral therapy clinics of Northeast Ethiopia.

Methods and materials

Study design, area and period

A hospital based cross-sectional study was conducted at Dessie Comprehensive Specialized Hospital (DCSH) from February 1, 2021 to March 30, 2021. According to the Dessie town health administrative office, in the town, there are 16 governmental health institutions (1 comprehensive specialized hospital, 1 general hospital, 8 health centers) and 3 private general hospitals, 6 higher private clinics, and the hospital serves about 12 million people living in the catchment areas. The Dessie comprehensive specialized hospital serves about 12 million people living in the catchment areas and has a 600 average daily patient flow also has 800 staff, among whom about 600 are health professionals. It has 600 beds, 8 wards, and different OPDs like adult, pediatric, emergency, and TB & HIV. Also, the hospital provides different services like laboratory, pharmacy, imaging (X-

ray, ultra-sound and city scan), and Dessie comprehensive specialized hospital ART service serves for 9,500 HIV positive.

Population

The source of populations were all HIV-positive individuals attending at DCSH. Whereas, HIV-positive patients who had symptomatic to community acquired UTI and visited the ART clinic of Dessie comprehensive specialized hospital during the study period before 48 hours of admissions were the target populations.

Eligibility criteria of study participants

HIV-positive patients who were ≥18 years old with suggestive symptoms of UTI including; lower abdominal or flank pain, dysuria and hematuria, urgency, frequency urinations were included in the study while patients who were mentally ill and unable to give samples were not included in the study.

Variables

Dependent variable the prevalence of bacterial and fungal community-acquired UTI was the dependent variable. While socio-demographic characteristics like (age, sex, marital status, educational level, residence) and clinical profile like (history of catheterization in the past year, Current ART status, symptoms of UTI (dysuria, urgency, frequency, flank pain, and urine color), diabetes, CD4+cell count, cotrimoxazole usage, and history of hospitalization) in the past year were independent variables.

Sample size and sampling technique

The required sample size was determined by using single population proportion formula by considering the proportion 28.6%(22) from previous Ethiopian study, marginal error of 5%, and 95% confidence interval = 1.96 by using the following formula:

$$n = \frac{(Z_{a/2})^2 * p*(1-p)}{d^2}$$

The calculated sample size was 314. Including 10% non-response rate a total of **346 UTI** symptomatic HIV-patients and fulfilling the inclusion criteria were included consecutively.

Operational definitions

UTI: Urinary tract infections are one of the most common infections in humans that can happen anywhere along the urinary tract and it is caused by microorganisms (microscopic organisms that

are too small to be seen by the naked eye). There are several different types of microorganisms which include bacteria, protozoa, fungi and algae (23, 24).

Community-acquired urinary tract infection (CA-UTI); Community-acquired UTI is an infection if an individual with UTI is suspected before hospital admission and specimens are collected from the outpatient or within less than 48 hours of hospital admission (25).

History of catheterization: the use of or insertion of a catheter into the bladder or participants who have catheterized before to data collections (26).

History of hospitalization: participants who were taken to a health facility and kept there for 48 hrs for treatment before to data collections in the past six months or more months.

Multidrug resistance: A bacterium that is simultaneously resistant to three or more antimicrobial categories (27).

Data and specimen collection

A pre-tested structured questionnaire was used to collect information on socio-demographic data (age, residence, and occupation), and clinical history (existing antibiotic treatment, previous antibiotic therapy, previous history of hospitalization for a longer period). The participants' recent CD4+ count was retrieved from their medical records. About 10 mL of voided clean-catch midstream fresh urine was collected from each study participant by using a leak-proof and sterile wide-mouthed screw-capped container. These specimens were labeled and stored in a cold box (4°C) and transported to the Wollo University College of Medicine and Health Sciences microbiology laboratory for analysis within 1 hour (28).

Isolation and identification of bacteria

Bacterial isolation and phenotypic characterization were performed using the recommended culture and biochemical tests (29). A calibrated loop that delivers 0.001 mL of urine was used to inoculate each urine sample on to the Cysteine lactose electrolyte deficient agar (Oxoid Ltd, UK). The plates were incubated aerobically at 37°C for 24 hours and colony count growth of $\geq 10^4$-10^5CFU/mL (colony-forming units per milliliter) was considered significant. Gram stain was performed from significant growth and sub cultured on to MacConkey agar (HiMediaTM) and 5% blood agar plates (HiMediaTM). However, bacteria that did not show growth after 24 hrs of incubation were further incubated for 24 hours and discarded as negative and the colony count was not significant (30). Colony characteristics, Gram reactions, and a series of biochemical reactions, including catalase, coagulase, oxidase, urease, indole, citrate utilization, lysine decarboxylase, glucose, lactose

fermentation, gas and H2S production, and motility tests were used for the isolations of bacteria (31).

Fungal Identification

Yeasts were identified by using routine diagnostic methods such as Gram stain, germ tube test, carbohydrate fermentation, and assimilation tests, as well as chromogenic medium (CHROMagar Candida Medium (Biomerieux, France) according to the manufacturer's instructions (32).

Antimicrobial susceptibility testing

An antimicrobial susceptibility test was performed by using Kirby–Bauer disk diffusion method based on the clinical laboratory standards Institute (CLSI) recommendation (33). About 3–5 pure colonies of isolated species from nutrient agar were picked and transferred to a tube containing 5 mL of tryptone-soya broth and mixed well to make a homogenous suspension. The suspension was incubated at 37°C until the turbidity of the suspension matched to a 0.5 McFarland standard. Using a sterile swab, the suspension was inoculated over the entire surface of the Mueller Hinton agar plate. The selected antimicrobial disks were put on the inoculated plates and incubated at 37°C for 16–18 hours. Antimicrobial agents were selected based on CLSI recommendations and local (Ethiopian) prescription habits for bacteria. The antimicrobials (Oxoid Ltd) that were used for bacterial susceptibility testing were, amoxicillin-clavulanic acid (AMC) 10µg, ampicillin (AMP) 10µg, amikacin (Amk) 30µg, cefotaxime (CTX) 30µg, ceftriaxone (CRO) 30µg, trimethoprim- sulphamethoxazole (SXT) 25µg, ciprofloxacin (CIP) 5µg, gentamycin (Gen) 10 µg, ceftazidime (CAZ) 30µg, nitrofurantoin (F) 300µg, tetracycline (TE) 30µg and penicillin (Pen) 10µg (33). Within 15 minutes after the application of the discs, the plates were incubated at 35°c for 18 hours. Diameters of zones of inhibitions were measured using digital caliper. The antimicrobial susceptibility test results were interpreted as sensitive, intermediate, or resistant based on the standardized CLSI guidelines and the isolates were considered MDR, resistant at least one antimicrobial in three or more antimicrobial categories (33).

Quality assurance

The questionnaire was pretested on 5% of HIV-positive patients at Dessie health center. The sterility of culture media was checked by incubating 5 % the prepared media for overnight at 37 ºC without specimen inoculation. The collected data was checked for completeness and adequate recording on the worksheet both during and after data collection. Standard Operating Procedures (SOP) were strictly followed for each microbiological procedure. All clinical specimens were collected, transported, and processed correctly. The expiration dates of the media, reagents, and Muller Hilton agar antimicrobial discs were

checked before use. The new batch culture medium and antimicrobial disks were checked for performance and quality using the American Type Culture Collection (ATCC) reference strains such as *E. coli* (ATCC® 25922), *S. aureus* (ATCC® 25923), *Klebsiella pneumonia* (ATCC 700603) and *P. aeruginosa* (ATCC® 27853).

Data management and statistical analysis

The data was entered into epi-data version 4.6.0.4 and exported to Social Sciences Statistical Package (SPSS) version 25 for analysis. Descriptive statistics were computed and presented using graphs and tables. A bivariate analysis was conducted to classify factors that separately affect the frequency of dependent variables. Variables with a P-value less than or equal to 0.25 in the bivariate analysis were subjected to multi-variable analysis. Finally, variables with an adjusted odd ratio and a p-value of <0.05 was considered as statistically significant.

Ethical considerations

Ethical clearance and permissions were obtained from the ethical review committee of Wollo University College of Medicine and Health Sciences with protocol number CMHS 365/2021 and a support letter was written to Dessie comprehensive specialized Hospital. Permission was obtained from Dessie comprehensive specialized hospitals. Written assent was also obtained from family and surrogates to obtain information from those who can't give data. Information obtained from the study participants was kept confidential. Positive results were communicated and given to attending physicians for appropriate treatment.

Results

Socio-demographic characteristics

A total of 346 HIV-positive patients with a response rate of 100% were included in this study. The age of the study participants was ranged from 21 to 80 years with a mean (±SD) age of 39.44 (±10.87) years, and 116 (33.5%) of them were in the age range of 35–44 years. About 180 (52.02%) of the respondents were females. Regarding educational status, 115 (33.2%) of the respondents had a primary-level of education. Moreover, 90 (26.01%) were self-employed and 215 (62.1%) of them were urban dwellers **(Table 1).**

Table 1. Sociodemographic characteristics of UTI symptomatic HIV positive patients attending at Dessie comprehensive specialized hospital, Dessie, Ethiopia.

Variable	Category	Urinary tract infection		Total, No (%)	Percentage
		Positive No (%)	Negative No (%)		
Sex	Male	42(25.3)	124(74.7)	166	47.98
	Female	50(27.8)	130(72.2)	180	52.02
Residence	Rural	62(28.8)	153(71.2)	215	62.1
	Urban	30(22.9)	101(77.1)	131	37.9
Age (in years)	18–24	10(18.9)	43(81.1)	53	15.3
	25–34	21(24.7)	64(75.3)	85	24.6)
	35–44	30(25.9)	86(74.1)	116	33.5
	>44	31(33.7)	61(66.3)	92	26.6
Educational status	Unable to read and write	42(36.5)	73(63.5)	115	33.2
	Primary	20(23.3)	66(76.7)	86	24.9
	Secondary	22(24.2)	69(75.8)	91	26.3
	College/University	8(14.8)	46(85.2)	54	15.6
Marital status	Married	45(37.2)	76(62.8)	121	35
	Unmarried	18(22.5)	62(77.5)	80	23.1
	Divorced	13(13.7)	82(86.3)	95	27.5
	Widowed	16(32)	34(68)	50	14.5
Occupation	House Wife	15(25.9)	43(74.1)	58	16.8
	Civil Servant	10(22.2)	35(77.8)	45	13
	Self-Employee	9(18)	41(82)	50	14.5
	Driver	10(20.8)	38(79.2)	48	13.9
	Daily Labor	23(25.6)	67(74.4)	90	26.01
	Farmer	25(45.5)	30(55.5)	55	15.9

Clinical characteristics of study participants

The clinical picture of study participants revealed that 150 (43.4%) and 140(40.5%) had a history of previous hospitalization and catheterization, of which 35(30.4%) and 42(30%) were culture positive for community-acquired urinary tract infections, respectively. About 121(35%) of the respondents had diabetes during the period of the study, with positive growth of 32(25.6%). About 105 (30.3%) HIV patients had 201-350 cells/mm3 CD4 count with positive growth of 35(33.3%). Moreover, 210(60.7%), 200 (57.8%), 165 (47.7%), and 164 (47.4%) participants showed dysuria, frequency of urination, flank pain, and abnormal urine color with positive growth of 58(27.6%), 55(27.5%), 61 (37%), and 64 (39%), respectively (**Table 2)**.

Table 2. Frequency of clinical characteristics of UTI symptomatic HIV positive patients attending Dessie comprehensive specialized hospital, Dessie, Ethiopia (February 1, 2021-march 30, 2021).

Variables	Category	Culture Result		Frequency	Percentage
		Positive No (%)	Negative No (%)		
History of hospitalization	Yes	35(30.4)	115(69.6)	150	43.4
	No	57(29.1)	139(70.9)	196	56.6
Use of cotrimoxazole as prophylaxis	Yes	40(26)	114(74)	154	44.5
	No	54(29.1)	138(70.9)	192	55.5
Diabetes	Yes	32(25.6)	89(74.4)	121	35
	No	60(24.6)	184(75.4)	244	65
History of catheterization	Yes	42(30)	120(70)	140	40.5
	No	50(24.3)	136(75.7)	206	59.5
UTI symptoms					
Dysuria	Yes	58(27.6)	152(72.4)	210	60.7
	No	34(25)	102(75)	136	39.3
Frequency of urination	Yes	55(27.5)	155(72.5)	200	57.8
	No	37(25.3)	109(74.7)	146	42.2
Urine color	abnormal	64(39)	100(61)	164	47.4
	Normal	28(15.4)	154(84.6)	182	52.6
Flank pain	Yes	61(37)	104(63)	165	47.7
	No	31(17.1)	157(82.9)	181	52.8
CD4+ count	≤200 cells/mm^3	24(32.9)	49(67.1)	73	21.1
	201-350 cells/mm3	35(33.3)	70(66.7)	105	30.3
	351-500 cells/mm3	20(23.8)	64(762)	84	24.3
	≥500 cells/mm3	13(15.3)	72(84.7)	85	24.6

Prevalence of community-acquired UTI and isolated pathogens

The overall prevalence of community-acquired UTIs was 26.6% (92/346) of which 81.52% (75/92) were bacteria and 18.48% (17/346) were fungal candida. Among 75 bacterial isolates, the most frequent bacterium was E. *coli* which accounted for 21.33% (16/75) followed by *K. pneumoniae*, *enterococcus species*, and *P. aeruginosa*, accounting for 14.67% (11/75), 13.33% (10/75), and 12% (9/75), respectively. The least number of bacterial isolates were *Enterobacter cloacae*, which accounted for 5.33% (4 /75). Gram-positive bacteria accounted for only 32% (24/75) of the bacterial isolates, of which 41.67% (10/24) of the isolates were *Enterococcus species*. Of 17 candida isolates, 47.1% (8/17) were represented by *C. tropicalis* (**Fig.1)**

Antimicrobial susceptibility pattern of bacterial Uropathogens

Gram Negative bacteria

From the tested antimicrobial susceptibility patterns, about 52.9%, 52.9%, and 58.8% of the total isolated Gram-negative uropathogens were susceptible to ceftriaxone, ciprofloxacin, and gentamicin, respectively, whereas 51%, 72.5%, 72.5%, 84.3%, 60.8% and 60.8% of the isolates were resistant to amikacin, ampicillin, amoxicillin-clavulanic acid, trimethoprim-sulphsmethsoxazole, nitrofurantoin, and ceftazidime, respectively. About 62.5% of *E. coli* strains were sensitive to gentamycin. But, 87.5%, 87.5%, 81.3%, 75%, 62.5% and 56 % of bacterial isolates *E. coli* were resistant to tetracycline, Trimethoprim-sulphamethoxazole, amoxicillin-Clavulanic acid, ampicillin, nitrofurantoin, and ceftazidime, respectively. Similarly, 80% of ampicillin, 90.9% of trimethoprim-sulphamethoxazole, 90.9% of tetracycline and 63.6% of nitrofurantoin, and 63.6% ceftazidime, 54.5% was resistance to K. *pneumoniae*. However, (54.5%) of gentamicin, and 54.5% of both amikacin and ciprofloxacillin, were sensitive to *K. pneumoniae* **(Table 3).**

Table 3. Antimicrobial susceptibility pattern of Gram-negative bacterial isolates from Community-acquired UTI among HIV-positive patients at Dessie comprehensive specialized hospital, Dessie, Ethiopia

Etiologic agents	Pattern	Antimicrobial agents N (%)										
		AMP	AMC	AK	CRO	CIP	GN	CAZ	TE	CTX	SXT	F
E. coli (16)	S	4(25)	3(18.7)	9(56.2	9(56.2	8(50)	10(62.5)	7(43.8)	2(12.5)	8(50)	2(12.5)	6(37.5)
	R	12(75)	13(81.3)	7(43.8)	7(43.8)	8(50)	6(37.5)	9(56.2)	14(87.5)	8(50)	14(87.5)	10(62.5
K. pneumoniae (11)	S	3(27.3)	4(36.4)	5(45.5)	6(54.5)	5(45.5)	6(54.5)	4(36.4)	1(9.1)	6(54.5)	1(9.1)	4(36.4)
	R	8(72.7	7(63.6)	6(54.5)	5(45.5)	6(54.5)	5(45.5)	7(63.6)	10(90.9)	5(45.5)	10(90.9)	7(63.6)
P. mirabilis (5)	S	1(20)	2(40)	3(60)	3(60)	3(60)	1(20)	2(40)	1(20)	3(60)	1(20)	2(40)
	R	4(80)	3(60)	2(40)	2(40)	2(40)	4(80)	3(60)	4(80)	2(40)	4(80)	3(60)
P. aeruginosa (9)	S	3(33.3)	2(22.2)	5(55.6)	5(55.6)	5(55.6)	6(66.7)	4(44.4)	1(11.1)	4(44.4)	2(22.2)	3(33.3)
	R	6(66.7)	7(87.8)	4(44.4)	4(44.4)	4(44.4)	3(33.3)	5(55.6)	8(88.9)	5(55.6)	7(87.8)	6(66.7)
Enterobacter cloacae. (4)	S	1(25)	1(25)	2(50)	2(50)	2(50)	3(75)	1(25)	1(25)	2(50)	1(25)	2(50)
	R	3(75)	3(75)	2(50)	2(50)	2(50)	1(25)	3(75)	3(75)	2(50)	3(75)	2(500
Providencia spp (6)	S	2(33.3)	2(33.3)	3(50)	2(33.3)	4(66.7)	4(66.7)	2(33.3)	1(16.7)	3(50)	1(16.7)	3(50)
	R	4(66.7)	4(66.7)	3(50)	4(66.7)	2(33.3)	2(33.30	4(66.7)	5(83.3)	3(50)	5(83.3)	3(50)
Total (51)	S	14(27.5)	14(27.5)	25(49)	27(52.9)	27(52.9)	30(58.8)	20(39.2)	7(13.7)	26(51)	8(15.7)	20(39.2)
	R	37(72.5)	37(72.5)	26(51)	24(47.1)	24(47.1)	21(41.2)	31(60.8	44(86.3	25(49)	43(84.3)	31(60.80

S: sensitive; R: resistant; AMP: ampicillin; AMC: Amoxicillin-Clavulanic acid; CTX: cefotaxime; CAZ: Ceftazidime; F: Nitrofurantoin; CRO: ceftriaxone; CIP: ciprofloxacin; GN: gentamicin; AK: Amikacin; TE: tetracycline; SXT: Trimethoprim-sulphamethoxazole.

Gram Positive bacteria

From the total isolated Gram-positive uropathogens, 66.7% of isolates were susceptible to clindamycin. Whereas, 87.5%, 83.3%, 75%, 58.3%, and 58.3% of Gram-positive isolates were resistant to tetracycline, trimethoprim-sulfamethoxazole, penicillin, chloramphenicol, and nitrofurantoin, respectively. About *Enterococcus species* was sensitive for 70% and 60% of clindamycin ciprofloxacin, respectively. However, *Enterococcus species* showed more resistance to 90% for tetracycline, 80% for trimethoprim-sulphsmethsoxazole, and 70% for nitrofurantoin. Likewise, *coagulase-negative staphylococci* (CoNs) were resistant to most of the antimicrobials 87.5% for tetracycline, 87.5% for trimethoprim-sulphamethoxazole, and 75% for penicillin and chloramphenicol each **(Table 4).**

Table 4. Antimicrobial susceptibility pattern of Gram-positive bacterial isolates from community-acquired UTI among HIV-positive patients attending Dessie comprehensive specialized hospital, Dessie, Ethiopia

Bacterial isolates		Antimicrobial Agents N (%)									
		E N (%)	P N (%)	C N (%)	CTX N (%)	F	CL N (%)	CIP N (%)	SXT N (%)	FOX N (%)	TE N (%)
S. *aureus* (6)	S	4(66.7)	1(16.7)	3(50)	3(50)	2(33.3**)**	4(66.7)	4(66.7)	1(16.7)	3(50)	1(16.7)
	R	2(33.3)	5(83.3)	3(50)	3(50)	4(66.7)	2(33.3)	2(33.3)	5(83.3)	3(50)	5(83.3)
CoNS (8)	S	3(37.5)	2(25)	2(25)	3(37.5)	5(62.5)	5(62.5)	2(25)	1(12.5)	NT	1(12.5)
	R	5(62.5)	6(75)	6(75)	5(62.5)	3(37.5)	3(37.5)	6(75)	7(87.5)	NT	7(87.5)
Enterococcus species (10)	S	4(40)	3(30)	5(50)	6(60)	3(30)	7(70)	6(60)	2(20)	4(40)	1(10)
	R	6(60)	7(70)	5(50)	4(40)	7(70)	3(30)	4(40)	8(80)	6(60)	9(90)
Total (24)	S	11(45.83)	6(25)	10(41.7)	12(50)	10(41.7)	16(66.7)	12(50)	4(16.7)	7(43.75)	3(12.5)
	R	13(54.17)	18(75)	14(58.3)	12(50)	14(58.3)	8(33.3)	12(50)	20(83.3)	9(56.25)	21(87.5)

Key: CL = Clindamycin, **E** = Erythromycin, **C** = Chloramphenicol, **CIP** = Ciprofloxacin, **TE** = Tetracycline, **SXT** = Trimethoprim-Sulfamethoxazole, **CTX**= cefotaxime, **FOX**=Cefoxitin, **F:** nitrofurantoin**, P** =penicillin, **R** = Resistant, **FOX**=Cefoxitin **S** = Sensitive, **NT** = Not tested

Multi-drug resistance patterns of the bacterial isolates

Overall, 70 (93.3%) bacterial isolates were resistant to at least one antimicrobial agent, and 63 (84%) isolates were resistant to ≥2 antimicrobials. About 11 (14.7%) isolates had resistance to five or more antimicrobials. The overall prevalence of MDR bacteria (a bacterium that is simultaneously resistant to three or more antimicrobial categories) was 81.3%(61/75). About *13* (81.3%) of *E. coli*, 9(81.8%) of *K. pneumoniae, 8*(80%) of *Enterococcus species, 7 (77.8%) of P. aeruginosa*, and *CoNs* 7(87.5%) were the most frequently exhibited MDR **(Table 5).**

Table 5. Multi-drug resistance patterns of uropathogenic bacterial isolates among UTI symptomatic HIV-positive patients attending Dessie comprehensive specialized hospital, Dessie, Ethiopia (February 1, 2021- March 30, 2021).

Isolated organisms	Total	MDR to antimicrobials						
		R0 N (%)	R1 N (%)	R2 N (%)	R3 N (%)	R4 N (%)	≥R5 N (%)	MDR N (%)
E. coli	16	1(6.3)	1(6.3)	1(6.3)	4(25)	7(43.7)	2(12.5)	13(81.3)
K. pneumoniae	11	1(9.1)	1(9.1)	-	4(36.4)	2(18.2)	3(27.3)	9(81.8)
S. aureus	6	1(16.7)	-	-	3(50)	2(33.3)	-	5(83.3)
P. mirabilis	5	0	1(20)	-	2(40)	1(20)	1(20)	4(80)
CoNs	8	1(12.5)	-	-	4(50)	2(25)	1(12.5)	7(87.5)
P. aeruginosa	9	0	1(11.1)	1(11.1)	3(33.3)	2(22.2)	2(22.2)	7(77.8)
Enterobacter Cloacae	4	0	1(25)	-	1(25)	2(50)	-	3(75)
Enterococcus species	10	1(10)	1(10)	-	4(40)	3(30)	1(10)	8(80)
Providencia species	6	0	1(16.7)	-	2(33.3)	2(33.3)	1(16.7)	5(83.3)
Total	75	5(6.7)	7(9.3)	2(2.7)	27(36)	23(30.7)	11(14.7)	61(81.3)

R0=No antibiotic resistance category, R1=Resistance to one antibiotic category, R2=Resistance to two antibiotics category, R3=Resistance to three antibiotics category, R4=Resistance to four antibiotics category, R5=Resistance to five antibiotics category

Factors associated with CA-UTI

In the current study, bivariate analysis was performed on sociodemographic characteristics such as sex, family size, residence, occupations, immunization status, educational status, current marital status, current CD4 count, history of hospitalizations, catheterizations, and status of diabetics; and signs and symptoms of urinary tract infections. Multivariate analysis was done by including variables that had a p-value of ≤0.25 in bivariate analysis. After adjustment for age, sex, occupation, educational status, current CD4+ count (cells/mm3), history of previous hospitalization and diabetes status, history of catheterizations, and urgency of the urinary tract. Age >44year [AOR=1.56,

95%CI: 1.11-9.35, P=0.001], being female [AOR=3.29, 95%CI: 1.26-13.41, P=0.002], being daily labor [AOR=4.54, 95%CI: 1.41-21.71, P=0.003], being farmer [AOR=6.54, 95%CI: 1.21-23.51, P=0.004], participants unable to read and write [AOR=4.58, 95%CI: 1.21-13.58, P=0.001], HIV patients with CD4 count of ≤ 200 cells/mm^3 (AOR: 7.44; 95%CI: 1.64-27.21, P=0.001) and CD4 count of 201-350 cells/mm^3 (AOR: 4.55; 95%CI: 1.36-18.28, P=0.009), patients who had chronic diabetics (AOR: 4.80; 95%CI: 1.85-16.31, p=0.009), patients who had chronic diabetics (AOR: 4.80; 95%CI: 1.85-16.31, p=0.009), patients having a history of hospitalization (AOR: 6.27; 95%CI: 1.25-15.17, p=0.002) and patients who had the urgency of urinations (AOR: 6.51; 95%CI: 1.75-36.31, p=0.0025) were statistically significant associated risk factors for community acquired urinary tract infections (Table 6).

Table 6. Bivariate and multivariable analysis for variables associated with CA-UTI among HIV patients attending Dessie comprehensive specialized hospital, Dessie, Ethiopia (February 1, 2021- march 30, 2021).

Characteristics		Urine Culture Result		COR (95% CI)	p-value	AOR (95% CI)	P-value
		Positive n (%)	Negative n (%)				
Age in years	18–24	10(18.9)	43(81.1)	1	1		
	25–34	21(24.7)	64(75.3)	0.65(0.25-5.295)	0.984		
	35–44	30(25.9)	86(74.1)	0.985(0.154-14.038)	0.838		
	>44	31(33.7)	61(66.3)	1.56 (1.11-19.035)	0.012	1.56(1.11-9.35)	0.001
Sex	Male	42(25.3)	124(74.7)	1	1	1	1
	Female	50(27.8)	130(72.2)	1.79(1.1-3.655)	0.185	3.29(1.26- 13.41)	0.002
Residence	Urban	62(28.8)	153(71.2)	1	1		
	Rural	30(22.9)	101(77.1)	1.4(0.427-3.026)	0.89		
Occupation	House Wife	15(25.9)	43(74.1)	1	1		
	Civil Servant	10(22.2)	35(77.8)	0.95(0.41-5.34)	0.54		
	Self-Employee	9(18)	41(82)	1.25(0.36-13.54)	0.36		
	Driver	10(20.8)	38(79.2)	0.71(0.15-16.12)	0.65		
	Daily Labor	23(25.6)	67(74.4)	3.842(1.37-17.91)	0.17	4.54(1.41-21.71)	0.003
	Farmer	25(45.5)	30(55.5)	4.842(1.17-17.91)	0.18	6.54(1.21-23.51)	0.004
Educational status	Unable to read and write	42(36.5)	73(63.5)	3.85(1.76-12.61)	0.114	4.58(1.21-13.85)	0.001
	Primary	20(23.3)	66(76.7)	0.98 (0.928-12.398)	0.265		
	Secondary	22(24.2)	69(75.8)	1.95(0.43-12.22)	0.655		
	College/University	8(14.8)	46(85.2)	1	1	1	1
Current marital status	Married	45(37.2)	76(62.8)	1.8([0.61-16.86)	0.43		
	Unmarried	18(22.5)	62(77.5)	1.369(0.325-5.766)	0.668		
	Divorced	13(13.7)	82(86.3)	1.05(0.153 -7.270)	0.956		
	Widowed	16(32)	34(68)	1	1		
	≤200	24(32.9)	49(67.1)	5.29(1.39-18.51)	**0.018**	7.44(1.64-27.21)	**0.001**

Current CD4+ count (cells/mm^3)	201-350	35(33.3)	70(66.7)	4.18(1.55-17.10)	**0.024**	4.55(1.36-18.28)	**0.009**
	351-500	20(23.8)	64(762)	0.98(0.57-12.56)	0.705	0.99(0.89-12.24)	0.861
	≥501	13(15.3)	72(84.7)	1	1	1	1
History of hospitalization	Yes	35(30.4)	115(69.6)	1.98(1.78- 13.28)	0.181	6.27(1.25- 15.17)	0.002
	No	57(29.1)	139(70.9)	1	1	1	1
Use of cotrimoxazole as prophylaxis	Yes	40(26)	114(74)	1	1		
	No	54(29.1)	138(70.9)	0.953(0.455-1.995)	0.898		
Chronic diabetes	Yes	32((25.6)	89(74.4)	3.78(1.22-15.42)	0.016	4.80(1.85- 16.31)	0.009
	No	60(24.6)	184(75.4)	1	1	1	1
History of catheterization	Yes	42(30)	120(70)	1.794(1.53-16.03)	0.048	4.65(1.55- 26.31)	0.0012
	No	50(24.3)	136(75.7)	1	1		
Dysuria	Yes	58(27.6)	152(72.4)	0.753(0.368-1.979)	0.711		
	No	34(25.4)	100(74.6)	1	1		
Frequency of urination	Yes	55(27.5)	155(72.5)	0.629(0.395-1.741)	0.621		
	No	37(25.3)	109(74.7)	1	1		
Urgency	Yes	64(39)	100(61)	1.427(1.89-22.55)	0.039	6.51(1.75- 36.31)	0.0025
	No	28(15.4)	154(84.6)	1	1		
Flank pain	Yes	61(37)	104(63)	1.193(0.548-2.599)	0.657		
	No	31(17.1)	157(82.9)	1	1		

Discussion

A urinary tract infection (UTI) is defined as the invasion of the urinary tract by one or more uropathogenic bacteria species, which results in significant bacteriuria and the presence of symptoms such as dysuria, pain and burning during urinating, cloudy urine, and urine appears red, bright pink (34). In this study, the prevalence of urinary tract infection was 26.6% [95% CI: 21.5% -31.2%]. The current study is in line with study done in Hawassa (23.1%)(35) and Uganda (22.0%)(36). However, this finding was relatively higher than studies done in Jimma (13.7%) (37), Gojjam Ethiopia (20.6%)(38), Hawassa (4.9%) (39), Addis Ababa, Ethiopia (18.6%) and 11.3% (16, 40) and Harar, Ethiopia (18%) (41). But, it is lower than studies conducted in Nigeria (32.5%) (42), South Africa (48.7%) (43), Nepal (54.76%) and India (100%), and (49.15%) (44, 45). This variation might be due to differences in sample size, the degree of the immune status of the study participants, geographical variation, ART status, and socioeconomic conditions.

In the current study, urinary tract infection with fungal was 4.9% (17/346). Of the 17 fungal isolates, 47.1% (8/17) were represented by *C. tropicalis*. The study reported on *C. albicans*, *C. glabrata*, and *C. tropicalis* supported the current study with the report of fungal species (46). Studies showed that within hospital settings at least 11.3 % of community-acquired UTIs are caused by Candida species and Candidauria (47). In a study conducted in Addis Ababa, Candidauria represented 7% UTIs in which *C. albicans* was the leading cause of Candidauria and 81.1% candida species were isolated (22), and a study was done in two microorganisms Candida spp. *(Candida glabrata* and *Candida krusei*) (48).

Among 75 bacterial isolates, 21.33% (16/75) were *E. coli*, the most frequent bacterium, followed by *K. pneumoniae*, *enterococcus species*, and *P. aeruginosa*, accounting for 14.67% (11/75), 13.33% (10/75), and 12% (9/75), respectively. Similarly, *E. coli* was predominant in studies in different parts of Ethiopia and across different countries like, Bahirdar (38.1%)(41), Gondar, (56.1%)(49), and Jimma, (54.3%) (37) from Ethiopia, tertiary care hospital, India (41.7%)(16), Saudi Arabia (50) and Ethiopia (39). However, it was inconsistent with the findings reported in Ebony State, Nigeria (51) and India (44), they reported that *S. aureus* (45.3%) and *P. aeruginosa* (41.9%) and in Turkey the most common causative agent was *E. coli* (66.6%) followed by *K. pneumoniae* (16.6%) and others such as *Enterobacter spp*. (7.7%) (48) were the commonest urinary tract pathogens. This discrepancy might be due to differences in the test facility, strain variety, geographical variation and study group variations. The presence of unique structure that aids bacteria attachment to uroepithelial cells, allowing for multiplication and tissue invasion, could explain the predominance of *E. coli* (52, 53).

Even though no statistically significant association was observed in this study, we found that the bacteriuria was high among females (27.8%) than males (25.3%). This finding is in agreement with various studies conducted in Ethiopia (40, 41, 49), South Africa (43), and Nigeria (51). This could be explained by physiological and anatomical differences between males and females, such as males' shorter urethra, a decrease in normal vaginal flora, and females' less acidic vaginal surface pH (40).

In the current study, about 52.9%, 52.9%, and 58.8% of the total isolated Gram-negative uropathogens were susceptible to ceftriaxone, ciprofloxacin, and gentamicin, respectively, whereas, 51%, 72.5%, 72.3%, 84.3%, 60.8%, and 60.8% of the isolates were resistant to amikacin, ampicillin, amoxicillin-Clavulanic acid, and trimethoprim-sulphamethoxazole. The other study on urinary tract

bacterial and fungal isolates supported these current findings with frequency percentage sulphamethoxazole, nitrofurantoin, and ceftazidime, and most of the urinary bacterial isolates were highly sensitive to ceftazidime (95%), and ciprofloxacin (88% (54).

In this study, 87.5%, 87.5%, 81.3%, 75%, 62.5%, and 56 % of *E. coli* strains were resistant to tetracycline, trimethoprim-sulphamethoxazole, amoxicillin-Clavulanic acid, ampicillin, nitrofurantoin, and ceftazidime, respectively. Similarly, 80% of ampicillin, 90.9% of trimethoprim-sulphamethoxazole, 90.9% of tetracycline, 63.6% of nitrofurantoin, and 63.6% of ceftazidime, 54.5% was resistance to *K. pneumoniae*. There were also similar findings from studies conducted in Addis Ababa which indicated *K. pneumoniae* had the highest level of resistance against trimethoprim-sulphamethoxazole (86.4%) cefotaxime (86.4%), cefepime (85.4%), ceftazidime (85.4%), amoxicillin-clavulanic acid (85.4%), gentamicin (70.0%), and ciprofloxacin (50.5% (55), In Iran: trimethoprim-sulphamethoxazole (91.4%), ceftazidime (91.4%), and gentamicin (82.8%) (56); in Sierra Leone: ciprofloxacin (73.4%) and gentamicin (60%) (57), in Equatorial Guinea: trimethoprim-sulphamethoxazole (100%), amoxicillin-clavulanic acid (96.6%), gentamicin (86.2%) and ciprofloxacin (87.5% (58). The occurrence of high antibiotic resistance might be due to misuse and overuse of antibiotics, and poor infection control measures (59) This was comparable with the study done in Gondar, Ethiopia (49) and Harar, Ethiopia (41), *P. aeruginosa* was 100% resistant to ampicillin, cefotaxime, and gentamicin and trimethoprim-sulfamethoxazole and *P.mirabilis* 100% resistant to ampicillin. This is in agreement with a study done in Ethiopia (40).

Similarly, about 87.5%, 83.3%, 75%, 58.3%, and 58.3% of Gram-positive isolates were resistant to tetracycline, trimethoprim-sulfamethoxazole, penicillin, chloramphenicol, and nitrofurantoin, respectively. The dominant isolated Gram-positive *Enterococcus species* showed more resistance to 90% of tetracycline, 80% of trimethoprim-sulphamethoxazole, and 70% of nitrofurantoin. Likewise, *coagulase-negative staphylococci* were resistant to 87.5% of tetracycline and trimethoprim-sulphamethoxazole, and 75% of penicillin and chloramphenicol, respectively. This is in line with a study done in Harar, Ethiopia, where 57.1% of *S. aureus* were resistant to gentamicin, tetracycline, and cefoxitin (57.1%) (41). We also found that 85% of *Coagulase-negative staphylococcus spp*. were resistant to trimethoprim-sulphamethoxazole (85%) and over 60% were resistant to penicillin, gentamicin, and tetracycline.

Multidrug resistance has serious implications for the health outcome of HIV-infected patients (60). It is quite alarming to note that almost 61(81.3%) of currently isolated bacteria were found to be resistant to three or more antimicrobials. This was higher compared to previous findings reported in Harar, Ethiopia (46%) (41), Dessie (74.6%) (61), Gondar (68%) (62), and Nepal (64.04%)(63) and India (28%) (64). But it was lower than a report from Gondar, Ethiopia (95%) and 87.4%) (12, 49), Addis Ababa (100%) (40), Bahirdar (93.1%) (65), Nepal (96.84%) (63). These differences might be due to the irrational drug utilization habit of the communities or to the distribution of those sensitive and resistant strains of bacteria.

There was an intra-species difference in MDR level. The present study showed that the level of MDR in *E. coli* 13 (81.3%), *K. pneumoniae* 9(81.8%), *Enterococcus species* 8(80%), *P. aeruginosa* 7 (77.8%), and CoNs 7(87.5%). This was comparable with a study conducted in Addis Ababa *K. pneumoniae* (83.5%) and *E. coli* (66.2%) (66), in Equatorial Guinea *E. coli* (74.4%) (58); in Sierra Leone *K. pneumoniae* (73.3%) and *E. coli* (61.5%) (57); in Gondar, *K. pneumoniae* was (95.6%) and *E. coli* was (92.9%) (12); in Khartoum-Sudan *E. coli* (92.2%)(67); and in Equatorial Guinea in *K. pneumoniae* (91.7%) (58). The MDR level among E. coli (50.2%) in Dessie is lower than in our study(61). The difference in MDR levels between *K. pneumoniae* and *E. coli* in our study might be most K. pneumoniae were isolated from blood specimens that were collected from inpatients in the hospitals.

In this study, there was no statistically significant association between significant bacteriuria and patients residence, marital status, dysuria, frequency of urination, flank pain, and use of co-trimoxazole. However, age, gender, occupation, educational status, current CD4+ count (cells/mm3), previous hospitalization and diabetes status, history of catheterizations, and urinary tract urgency had a statistically significant association with significant bacteriuria and Candidauria. Similar findings were reported from Jimma, Addis Ababa, and Harar (37, 40, 41). Furthermore, age groups >44year [AOR=1.56, 95%CI: 1.11-9.35, P=0.001], being female [AOR=3.29, 95%CI: 1.26-13.41, P=0.002], being daily labor [AOR=4.54, 95%CI: 1.41-21.71, P=0.003], being farmer [AOR=6.54, 95%CI: 1.21-23.51, P=0.004], participants unable to read and write [AOR=4.58, 95%CI: 1.21-13.58, P=0.001], HIV patients with CD4 count of ≤ 200 cells/mm3 (AOR: 7.44; 95%CI: 1.64-27.21, P=0.001) and CD4 count of 201-350 cells/mm3 (AOR: 4.55; 95%CI: 1.36-18.28, P=0.009), patients who had chronic diabetics (AOR: 4.80; 95%CI: 1.85-16.31, p=0.009), patients having a history of hospitalization (AOR: 6.27; 95%CI: 1.25-15.17, p=0.002) and patients

who had the urgency of urination (AOR: 6.51; 95%CI: 1.75-36.31, p=0.0025). Studies conducted in Nepal support our findings with a significant risk factor for community-acquired urinary tract infections in patients with indwelling urinary catheters (38.3%), Diabetes-mellitus (36%), malignancies (5.2%), and urinary incontinence (11.7%). Urinary catheters are a proven source of infection, especially when they are kept for longer periods (47). Similarly, research finding was supported by studies conducted elsewhere [26, 22, 27, and 29]. The explanation for the inverse relationship between UTI and a CD4+ count is unknown; it is probably due to the impaired immunity at a declining CD4+ count that makes it easier for bacterial pathogens to adhere to the urinary epithelium.

Limitations of the study

The study was a single hospital-based study and might not represent all HIV-infected patients and did not include a control group due to budget constraints. We did not attempt to identify other causative agents (anaerobic bacteria, and fungus) that would have made a significant contribution to a true prevalence of UTI in HIV-positive patients.

Conclusions and Recommendations

The symptomatic community's acquired UTI is high, with an overall prevalence of 26.6% (92/346). Of the isolated pathogens, 81.52% (75/92) were bacterial pathogens, while 18.48% (17/92) were fungal pathogens. Among bacterial pathogens, *E. coli*, the most frequent bacterium, followed by *K. pneumoniae*, *enterococcus species*, and *P. aeruginosa*, accounting for, respectively. The overall prevalence of MDR bacteria was 61(81.3%). About 13 (81.3%) of *E. coli*, 9(81.8%) of *K. pneumoniae*, 8(80%) of *Enterococcus species*, 7 (77.8%) of *P. aeruginosa*, and *CoNs* 7(87.5%). Most Gram-negative and Gram-positive isolates were resistant to tetracycline, trimethoprim-sulfamethoxazole, penicillin, chloramphenicol, and nitrofurantoin, respectively. However, uropathogenic were susceptible to amikacin, it was more effective against these pathogens. Patients who had low CD4 counts, previous hospitalization, diabetes status, and having history of catheterizations than their counterparts were more at risk of developing bacterial urinary tract infections. Management of UTI among symptomatic HIV patients should be supported by laboratory results of urine culture. Regular monitoring of antimicrobial resistance patterns should be undertaken by healthcare providers. Individuals at risk for bacterial-associated UTI should have good adherence to ART and their CD4 levels should be monitored regularly. Health information dissemination should be given about UTIs and the habit of drug use for HIV-positive patients.

List of abbreviations and acronyms

AIDS; Acquired immune deficiency syndrome, ARTC; Attending antiretroviral therapy clinic, ART; Antiretroviral therapy, ATCC; American Type Culture collections, BA; Blood agar, CFU; Colony Forming Unit, CLED; Cysteine Lactose Electrolyte Deficient, CONS; Coagulase-negative Staphylococci, HAART; Highly active antiretroviral therapy, HIV; Human Immunodeficiency Virus, MACA; MacConkey agar, MDR; Multi-Drug Resistant, MSU; Midstream urine, OIS; Opportunistic infections, UTI; Urinary Tract Infection, VCT Voluntary counseling, and Testing.

Data availability statement

Data supporting the conclusions of this article are within the manuscript and are available on reasonable request from the principal investigators due to ethical reasons.

Funding

The authors were no obtained specific fundings for this work. only internal material support was obtained from Wollo University. The funders had no role in study design, data collection, and analysis, the decision to publish, or the preparation of the manuscript.

Competing interests

The authors declare that they have no competing interests.

Authors' contributions

Conceived and designed the experiments: Mihret Tilahun, Abdurrahman Seid, Bekele Sharew, Tewodros Dessie, Habtamu Mekonen; Performed the experiments: Mihret Tilahun, Abdurrahman Seid, Agumas Shibabaw; Analyzed the data: Mihret Tilahun, Abdurrahman Seid, Daniel G/tsadik, Mesfin Fiseha, Melkam Tesfaye, Alemu Gedefie, Melaku Ashagrie Belete; Wrote and edited the manuscript: Mihret Tilahun, Abdurrahman Seid, Bekele Sharew, Alemu Gedefie, Habtu Debash, Agumas Shibabaw, Melaku Ashagrie Belete, and Habtamu Mekonnen. All authors read and approved the final manuscript.

Acknowledgment

The authors would like to acknowledge the Department of Medical Laboratory Science, College of Medicine and Health Sciences, Wollo University for providing the laboratory setup and facilities to conduct the experiments. Dessie comprehensive specialized hospital and all study participants are gratefully acknowledged for their kind cooperation during data collection.

References

1. Syed B, Salahuddin N, Ishtiaq H. Etiologies and microbiological profile of complicated urinary tract infections, among patients admitted in a tertiary care hospital. *Infec Dis J Pakistan*. 2017;26:59-64.
2. Kiranmala K, Johnson R, Savio J, Idiculla J. Microbiologic profile and clinical practices in urinary tract infections in a tertiary care center in Southern India. *Journal of Family Medicine and Primary Care*. 2019;8(9):2888.
3. Nerurkar A, Solanky P, Naik SS. Bacterial pathogens in urinary tract infection and antibiotic susceptibility pattern. *Journal of Pharmaceutical and Biomedical Sciences*. 2012;21(21).
4. Ade-Ojo I, Oluyege A, Adegun P, Akintayo A, Aduloju O, Olofinbiyi B. Prevalence and antimicrobial susceptibility of asymptomatic significant bacteriuria among new antenatal enrollees in Southwest Nigeria. *International Research Journal of Microbiology*. 2013;4(8):197-203.
5. Schmider J, Bühler N, Mkwatta H, Lechleiter A, Mlaganile T, Utzinger J, et al. Microbiological characterization of community-acquired urinary tract infections in Bagamoyo, Tanzania: *A prospective study*. *Tropical Medicine and Infectious Disease*. 2022;7(6):100.
6. Tegegne KD, Wagaw GB, Gebeyehu NA, Yirdaw LT, Shewangashaw NE, Kassaw MW. Prevalence of urinary tract infections and risk factors among diabetic patients in Ethiopia, a systematic review and meta-analysis. *PloS one*. 2023;18(1):e0278028.
7. Birhanu MY, Habtegiorgis SD, Gietaneh W, Alemu S, Tsegaye TB, Bekele GM, et al. The magnitude and associated factors of urinary tract infections among adults living with HIV in Ethiopia. Systematic review and meta-analysis. *Plos one*. 2022;17(4):e0264732.
8. Çoban B, Ülkü N, Kaplan H, Topal B, Erdoğan H, Baskın E. Five-year assessment of causative agents and antibiotic resistances in urinary tract infections. *Turkish Archives of Pediatrics/Türk Pediatri Arşivi*. 2014;49(2):124.
9. Flores-Mireles AL, Walker JN, Caparon M, Hultgren SJ. Urinary tract infections: epidemiology, mechanisms of infection and treatment options. *Nature reviews microbiology*. 2015;13(5):269-84.
10. Odabasi Z, Mert A. Candida urinary tract infections in adults. *World journal of urology*. 2020;38(11):2699-707.
11. Behzadi P, Behzadi E, Ranjbar R. Urinary tract infections and Candida albicans. *Central European journal of urology*. 2015;68(1):96-101.
12. Eshetie S, Unakal C, Gelaw A, Ayelign B, Endris M, Moges F. Multidrug-resistant and carbapenemase-producing Enterobacteriaceae among patients with urinary tract infection at

Referral Hospital, Northwest Ethiopia. *Antimicrobial resistance and infection control*. 2015;4(1):1-8.

13. Ahmed SS, Shariq A, Alsalloom AA, Babikir IH, Alhomoud BN. Uropathogens and their antimicrobial resistance patterns: Relationship with urinary tract infections. *International Journal of Health Sciences*. 2019;13(2):48.
14. Prakash D, Saxena RS. Distribution and antimicrobial susceptibility pattern of bacterial pathogens causing urinary tract infection in the urban community of Meerut city, India. *International scholarly research notices*. 2013;2013.
15. Haile Hantalo A, Haile Taassaw K, Solomon Bisetegen F, Woldeamanuel Mulate Y. Isolation and Antibiotic Susceptibility Pattern of Bacterial Uropathogens and Associated Factors Among Adult People Living with HIV/AIDS Attending the HIV Center at Wolaita Sodo University Teaching Referral Hospital, South Ethiopia. *HIV/AIDS-Research and Palliative Care*. 2020:799-808.
16. Fenta G, Legese M, Weldearegay G. Bacteriuria and their antibiotic susceptibility patterns among people living with HIV attending Tikur Anbessa Specialized and Zewditu Memorial Hospital ART Clinics, Addis Ababa, Ethiopia. *J Bacteriol Parasitol.* 2016;7(05).
17. Yismaw G, Asrat D, Woldeamanuel Y, Unakal CG. Urinary tract infection: bacterial etiologies, drug resistance profile, and associated risk factors in diabetic patients attending Gondar University Hospital, Gondar, Ethiopia. *European Journal of Experimental Biology*. 2012;2(4):889-98.
18. Kibret M, Abera B. Prevalence and antibiogram of bacterial isolates from urinary tract infections at Dessie Health Research Laboratory, Ethiopia. *Asian Pacific journal of tropical biomedicine*. 2014;4(2):164-8.
19. Paschke AA, Zaoutis T, Conway PH, Xie D, Keren R. Previous antimicrobial exposure is associated with drug-resistant urinary tract infections in children. *Pediatrics*. 2010;125(4):664-72.
20. Frank-Peterside N, Okerentugba P, Nwodo C, Okonko I. Prevalence of bacterial uropathogens in a cohort of HIV-positive males in Port Harcourt, Nigeria. Cancer Biology. 2013;3(4):12-7.
21. Skrzat-Klapaczyńska A, Matłosz B, Bednarska A, Paciorek M, Firląg-Burkacka E, Horban A, et al. Factors associated with urinary tract infections among HIV-1 infected patients. *PLOS one*. 2018;13(1):e0190564.
22. Bitew A, Zena N, Abdeta A. Bacterial and fungal profile, antibiotic susceptibility patterns of bacterial pathogens and associated risk factors of urinary tract infection among symptomatic

pediatrics patients attending St. Paul's Hospital Millennium Medical College: a cross-sectional study. *Infection and drug resistance*. 2022;15:1613.

23. Foxman B. Urinary tract infection syndromes: occurrence, recurrence, bacteriology, risk factors, and disease burden. *Infectious Disease Clinics*. 2014;28(1):1-13.
24. Lee Y-C, Hsiao C-Y, Hung M-C, Hung S-C, Wang H-P, Huang Y-J, et al. Bacteremic urinary tract infection caused by multidrug-resistant Enterobacteriaceae are associated with severe sepsis at admission: implication for empirical therapy. *Medicine*. 2016;95(20).
25. Islam MA, Islam MR, Khan R, Amin MB, Rahman M, Hossain MI, et al. Prevalence, etiology and antibiotic resistance patterns of community-acquired urinary tract infections in Dhaka, Bangladesh. *Plos one*. 2022;17(9):e0274423.
26. Haider MZ, Annamaraju P. Bladder Catheterization. StatPearls [Internet]: StatPearls Publishing; 2022.
27. Magiorakos A-P, Srinivasan A, Carey RB, Carmeli Y, Falagas M, Giske C, et al. Multidrug-resistant, extensively drug-resistant and pan drug-resistant bacteria: an international expert proposal for interim standard definitions for acquired resistance. *Clinical microbiology and infection.* 2012;18(3):268-81.
28. Bigwan E, Wakjissa F. Prevalence of urinary tract infections among HIV patients attending a non-governmental health facility in Jos, Plateau State, Nigeria. *International Journal of Biomedical and Advance Research.* 2013;4(8):528-33.
29. Murray PR, Rosenthal KS, Pfaller MA. Medical microbiology E-book: *Elsevier Health Sciences*; 2020.
30. Pezzlo M. Laboratory diagnosis of urinary tract infections: guidelines, challenges, and innovations. *Clinical Microbiology Newsletter*. 2014;36(12):87-93.
31. Bullock NO, Aslanzadeh J. Biochemical profile-based microbial identification systems. Advanced techniques in diagnostic microbiology: Springer; 2013. p. 87-121.
32. Malik UM, Khan AB, Satti ML. Comparative evaluation of CHROMagar and API 20C AUX in isolation and identification of Candida species. *Journal of Islamic International Medical College* (JIIMC). 2018;13(2):85-90.
33. Institute CLS. CLSI Performance standard for antimicrobial susceptibility testing, M100. 2021.

34. Behzadi P, Urbán E, Matuz M, Benkő R, Gajdács M. The role of gram-negative bacteria in urinary tract infections: current concepts and therapeutic options. *Advances in Microbiology, Infectious Diseases, and Public Health.* 2020:35-69.
35. Nigussie D, Amsalu A. Prevalence of uropathogenic and their antibiotic resistance pattern among diabetic patients. *Turkish Journal of Urology*. 2017;43(1):85.
36. Nabaigwa BI, Mwambi B, Okiria J, Oyet C. Common uropathogens among diabetic patients with urinary tract infection at Jinja Regional Referral Hospital, Uganda. *African Journal of Laboratory Medicine*. 2018;7(1):1-3.
37. Debalke S, Cheneke W, Tassew H, Awol M. Urinary tract infection among antiretroviral therapy users and nonusers in Jimma University Specialized Hospital, Jimma, Ethiopia. *International journal of microbiology*. 2014;2014.
38. Girma A, Aemiro A. The Bacterial Profile and Antimicrobial Susceptibility Patterns of Urinary Tract Infection Patients at Pawe General Hospital, Northwest Ethiopia. *Scientifica.* 2022;2022.
39. Tessema NN, Ali MM, Zenebe MH. Bacterial associated urinary tract infection, risk factors, and drug susceptibility profile among adult people living with HIV at Haswassa University Comprehensive Specialized Hospital, Hawassa, Southern Ethiopia. *Scientific Reports*. 2020;10(1):1-9.
40. Getu Y, Ali I, Lema T, Belay H, Yeshetela B. Bacteriuria and antimicrobial susceptibility pattern among HIV patients attending ALERT Center, Addis Ababa, Ethiopia. *Am J Health Res*. 2017;5(3):76-82.
41. Marami D, Balakrishnan S, Seyoum B. Prevalence, Antimicrobial Susceptibility Pattern of Bacterial Isolates, and Associated Factors of Urinary Tract Infections among HIV- Positive Patients at Hiwot Fana Specialized University Hospital, Eastern Ethiopia. C*anadian Journal of Infectious Diseases and Medical Microbiology*. 2019;2019.
42. Okechukwu A, Thairu Y. Bacteria urinary tract infection in HIV-infected children and adolescents in Abuja, Nigeria: a cross-sectional study. *African Journal of Clinical and Experimental Microbiology*. 2019;20(4):306-14.
43. Iweriebor B, Obi C, Akinyemi O, Ramalivhana N, Hattori T, Okoh A. uropathogens isolated from HIV-infected patients from Limpopo Province, South Africa. *African Journal of Biotechnology*. 2012;11(46):10598-604.

44. Xavier TF, Auxilia A, Kannan M. Isolation and characterization of UTI pathogens from HIV positive patients of Karur District, Tamil Nadu, India. *Int J Curr Microbiol Appl Sci.* 2015;4(1):558-63.
45. Sharma V, Gupta V, Mittal M. Prevalence of uropathogens in diabetic patients and their antimicrobial susceptibility pattern. *Natl J lab Med*. 2012;1(1):26-8.
46. Achkar JM, Fries BC. Candida infections of the genitourinary tract. Clinical microbiology reviews. 2010;23(2):253-73.
47. Aadil Rafeeq Rather1* ANKaTM. Profile of Community Acquired Urinary Tract Infections at a Teriary Care Hospital. Volume 2 Issue 11 November 2019. 2019.
48. Erdem I, Ali RK, Ardic E, Omar SE, Mutlu R, Topkaya AE. Community-acquired lower urinary tract infections: Etiology, antimicrobial resistance, and treatment results in female patients. J*ournal of global infectious diseases*. 2018;10(3):129.
49. Alemu A, Dagnew M, Alem M, Gizachew M. Uropathogenic bacterial isolates and their antimicrobial susceptibility patterns among HIV/AIDS patients attending Gondar University Specialized Hospital Gondar, Northwest Ethiopia. 2013.
50. Biradar SK, Doddamani P. Prevalence and antibiogram of uropathogens in a tertiary care hospital. *World J Pharmaceut Res*. 2013;2:1534-43.
51. Ifeanyichukwu I, Emmanuel N, Chika E, Anthonia O, Esther U-I, Ngozi A, et al. Frequency and antibiogram of uropathogens isolated from urine samples of HIV-infected patients on antiretroviral therapy. *American Journal of BioScience*. 2013;1(3):50-3.
52. Ejerssa AW, Gadisa DA, Orjino TA. Prevalence of bacterial uropathogens and their antimicrobial susceptibility patterns among pregnant women in Eastern Ethiopia: hospital-based cross-sectional study. *BMC women's health*. 2021;21(1):1-15.
53. Fierer J, Looney D, Kok M, Pechère J-C. Nature and pathogenicity of micro-organisms. Infectious diseases. 2010:3.
54. Nwadioha S, Nwokedi E, Ikeh I, Egesie J, Kashibu E. Antibiotic susceptibility pattern of uropathogenic bacterial isolates from AIDS patients in a Nigerian tertiary hospital. *J Med Med Sci*. 2010;1(11):530-4.
55. Teklu DS, Negeri AA, Legese MH, Bedada TL, Woldemariam HK, Tullu KD. Extended-spectrum beta-lactamase production and multi-drug resistance among Enterobacteriaceae isolated in Addis Ababa, Ethiopia. *Antimicrobial Resistance & Infection Control.* 2019;8:1-12.

56. Mansouri S, Abbasi S. Prevalence of multiple drug resistant clinical isolates of extended-spectrum beta-lactamase-producing Enterobacteriaceae in Southeast Iran. 2010.
57. Leski TA, Taitt CR, Bangura U, Stockelman MG, Ansumana R, Cooper WH, et al. High prevalence of multidrug-resistant Enterobacteriaceae isolated from outpatient urine samples but not the hospital environment in Bo, Sierra Leone. *BMC infectious diseases.* 2016;16(1):1-9.
58. Shatalov A. Prevalence and antibiotic resistance pattern of Escherichia coli and Klebsiella pneumoniae in urine tract infections at the La Paz Medical Center, Malabo, Equatorial Guinea. *Open Journal of Medical Microbiology*. 2015;5(04):177.
59. Adler A, Katz DE, Marchaim D. The continuing plague of extended-spectrum β-lactamase–producing Enterobacteriaceae infections. *Infectious Disease Clinics.* 2016;30(2):347-75.
60. Kim H-R, Hwang SS, Kim HJ, Lee SM, Yoo C-G, Kim YW, et al. Impact of extensive drug resistance on treatment outcomes in non-HIV-infected patients with multidrug-resistant tuberculosis. *Clinical Infectious Diseases.* 2007;45(10):1290-5.
61. Kibret M, Abera B. Antimicrobial susceptibility patterns of E. coli from clinical sources in northeast Ethiopia. *African health sciences*. 2011;11:40-5.
62. Moges F, Mengistu G, Genetu A. Multiple drug resistance in Urinary pathogens at Gondar College of Medical science hospital, Ethiopia. East African medical journal. 2002;79(8):415-20.
63. Yadav KK, Adhikari N, Khadka R, Pant AD, Shah B. Multidrug-resistant Enterobacteriaceae and extended-spectrum β-lactamase producing Escherichia coli: a cross-sectional study in National Kidney Center, Nepal. *Antimicrobial resistance and infection control*. 2015;4(1):1-7.
64. Murugesh K, Deepa S, Ravindranath C, Venkatesha D. Multi-drug resistant uropathogens in HIV: Are they a threat to the community? International Journal of Scientific Study. 2014;2(3):38-42.
65. Biadglegne F, Abera B. Antimicrobial resistance of bacterial isolates from urinary tract infections at Felge Hiwot Referral Hospital, Ethiopia. *The Ethiopian Journal of Health Development.* 2009;23(3).
66. Teklu DS, Negeri AA, Legese MH, Bedada TL, Woldemariam HK, Tullu KD. Extended-spectrum beta-lactamase production and multi-drug resistance among Enterobacteriaceae isolated in Addis Ababa, Ethiopia. *Antimicrobial Resistance & Infection Control*. 2019;8(1):1-12.
67. Ibrahim M, Bilal N, Hamid M. Increased multi-drug resistant Escherichia coli from hospitals in Khartoum state, Sudan. *African health sciences.* 2012;12(3):368-75.

Figure 1 legends

Etiology of UTI among HIV patients attending Antiretroviral Therapy Clinics of Northeast Ethiopia

Author's Response to Editor and Reviewer comments

Manuscript ID number:
PONE-D- 22-31717

Title of paper:

Bacterial and fungal profile, multidrug resistance profile of bacterial pathogens and associated risk factors of community-acquired urinary tract infection among HIV positive patients attending Antiretroviral Therapy Clinics of Northeast Ethiopia

General

We appreciate all of the constructive feedback on our manuscript from the reviewers, which has helped us improve the paper's clarity and scientific plausibility. We thank the PLOS ONE scholarly editors for their insightful comments and for allowing us to update the text. All of the concerns raised by the reviewers and editors have been addressed, and the text has been updated as a result. Furthermore, both the updated article and the response letter address all of the editorial revisions that are required. The changes are shown with track changes in the file labelled "Revised Manuscript with Track Modifications." The following is a detailed rebuttal.

Editor Comments:

1. Please ensure that your manuscript meets PLOS ONE's style requirements, including those for file naming. The PLOS ONE style templates can be found at https://journals.plos.org/plosone/s/file?id=wjVg/PLOSOne_formatting_sample_main_body.pdf and https://journals.plos.org/plosone/s/file?id=ba62/PLOSOne_formatting_sample_title_authors_affiliations.pdf

Author's Response:

- **Thanks for the substantial editorial comment. The revised manuscript is now updated, highlighted and meets PLOS ONE's style requirements, including those for file naming.**

2. We noticed you have some minor occurrence of overlapping text with the following previous publication(s), which needs to be addressed: - http://etd.aau.edu.et/bitstream/handle/123456789/6042/Yemisrach%20Getu.pdf? In your revision ensure you cite all your sources (including your own works), and quote or rephrase any duplicated text outside the methods section. Further consideration is dependent on these concerns being addressed

Author's Response:

- **Thank you for the interesting comment. Minor over lapping's are corrected**

3. We suggest you thoroughly copyedit your manuscript for language usage, spelling, and grammar. If you do not know anyone who can help you do this, you may wish to consider employing a professional scientific editing service.

” ***Author's Response:***

- **Thank you and corrections are made as per the comment; all the authors are senior expert and published many papers and one of the authors Melaku Ashagire Belete is the editors of plose one. So no need of language editors.**

4. Thank you for stating the following financial disclosure: “All the authors are under developing countries and no extrena funding was obtained” At this time, please address the following querie.

Author's Response:

- **Thank you for the interesting comment. financial disclosure corrected as No external funds were obtained only internal support was obtained from Wollo university. The funders had no role in study design, data collection and analysis, decision to publish, or preparation of the manuscript**

Reviewer #1 comments

Reviewer #1: Check the sticky note on your PDF file. Try to address all the comments. I have tried to your entire manuscript it lacks basic thing. if modified and edited with expert in the field it match improved. But I saw some errors that makes me to suspicious on your data. Rewrite the introduction part and discussion part seriously.

Author's Response:

- **Thanks, and we have amended all comments as per request and highlighted and some questions and comments are here below**
  The date was collected from patients before 48 hours. The data written 18 hours is clerical error

1. Write Bacterial and fungal profile as uropathogens, multidrug resistance profile rephrase of bacterial pathogens and associated risk factors as associated factorstof community-acquired urinary tract infection among HIV positive patients attending Antiretroviral Therapy
   Clinics of Northeast Ethiopia

   Author's Response

   We modify the title Uropathogens, multidrug resistance pattern of bacterial and associated factors of community-acquired urinary tract infection among HIV positive patients attending Antiretroviral Therapy Clinics of Northeast Ethiopia.

2. Source of standardized questionaries was where? And why not performed Recent CD4 count?

   Author's Response:

   - Thanks for the vivid comment. The source of questionaries or we were developed the questionaries by modifications from WHO Suspected Urinary Tract Infection patient questionnaire and from other published articles
   - CD4+ cell counts are usually measured when you are diagnosed with HIV (at baseline), every 3 to 6 months during first 2 years or until your CD4 count increases above 300 cells/mm3. So it is better to take from their medical records those who have less than six months

3. Can you justify do you collect mid steam urine or catheterized patients? What about significance .

Author's Response:

- **Thank you for the substantial comment. We have collected mid-stream urine and symptomatic patients so the interpretations are Symptomatic ambulatory patient; voided or clean-catch specimen: report if growth is ≥ 10,000 CFU/ml with 1-2 species of potential pathogen.**

4. **On the result section all comments are corrected per comments and sugestions but the same isolates are 74 not 75 ?**

Author's Response:

- **Thanks for the valuable Comment and you can calculated and subtract again 92-17 =75**

5. **Result and discussion**: how do you identify C.tropicalis from C.albican

Author's Response:

- **Thank you for the valuable comment. Gram stain, Germ Tube Test, Carbohydrate Fermentation, and Assimilation Tests, as well as chromogenic medium (CHROMagar Candida Medium (Biomerieux, France) according to the manufacturer's instructions .**
- **Perry and Miller reported that Candida albicans produces an enzyme b -N-acetyl- galactosaminidase and according to Rousselle et al incorporation of chromogenic or fluorogenic hexosaminidase substrates into the growth medium helps in identification of C. albicans isolates directly on primary isolation. HiCrome Candida Differential Agar is a selective and differential medium, which facilitates rapid isolation of yeasts from mixed cultures and allows differentiation of Candida species namely C. albicans, C. krusei, C. tropicalis and C. glabrata on the basis of coloration and colony morphology. On this medium results are obtained within 48 hours and it is useful for the rapid and presumptive identification of common yeasts in Mycology and Clinical Microbiology Laboratory. Peptone special and yeast extract provides nitrogenous, carbonaceous compounds and other essential growth nutrients. Phosphate buffers the medium well. Chloramphenicol suppresses the accompanying bacterial flora. C. albicans appear as light green colored smooth colonies, C. tropicalis appear as blue to metallic blue colored raised colonies. C. glabrata colonies appear as cream to white smooth colonies, while C. krusei appear as purple fuzzy colonies.**

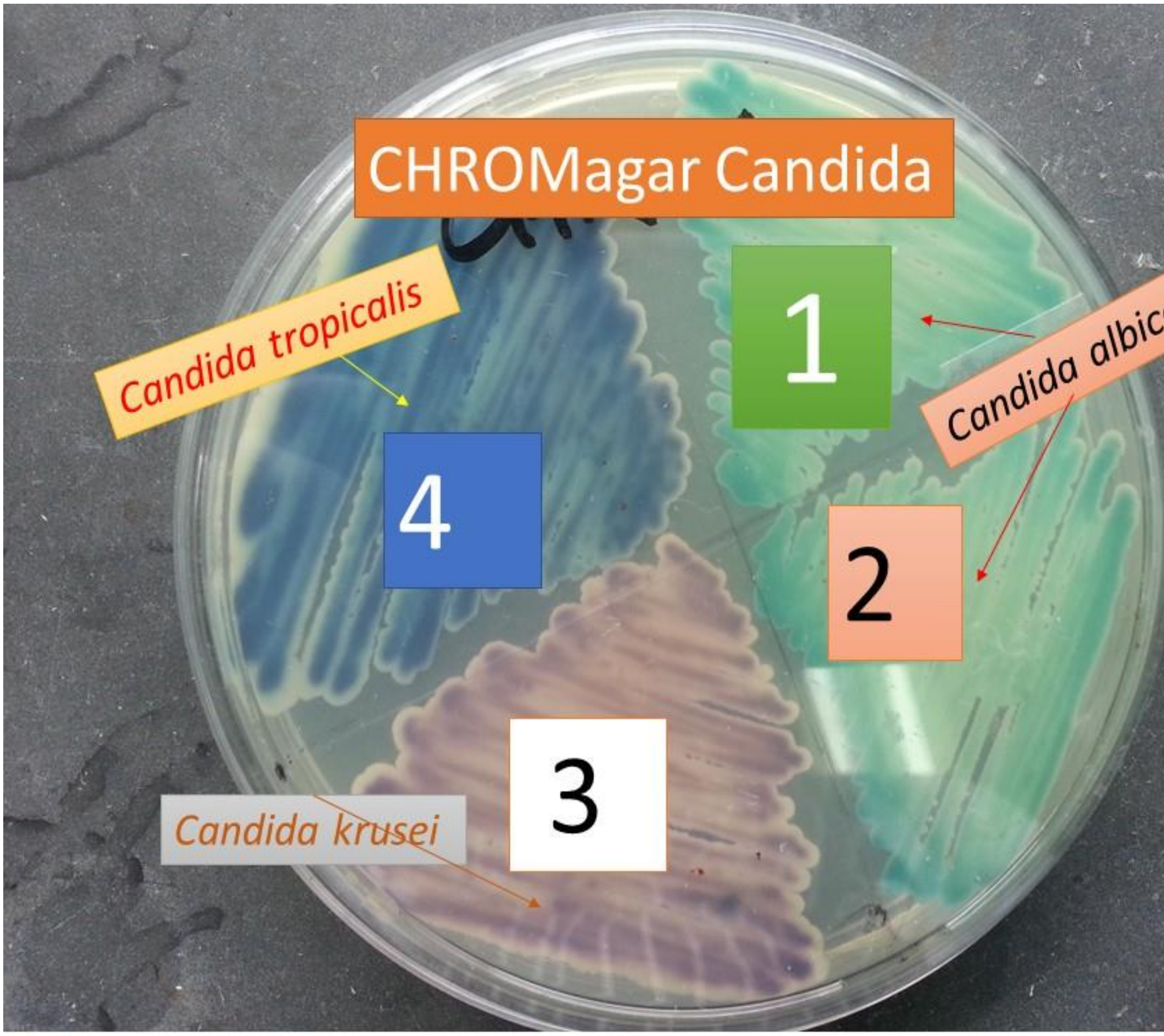

6. Rewrite MDR based on guideline of for table construct from journal for submisions.

Author's Response:

- **Thanks, and we have amended the conclusion part as we write on the A bacterium that is simultaneously resistant for three or more antimicrobial categories. I understand your questions but to write each bacteria it is very length.**

7. **Using references 23 From Hawassa are not ART**
   Author's Response:
   We use Hawassa non-HIV patients as a reference because both HIV and Diabetics are chronic diseases and they lead patients immunocompromised

Reviewer #1: Check the sticky note on your PDF file. Try to address all the comments. I have tried to your entire manuscript it lacks basic thing. if modified and edited with expert in the field it match improved. But I saw some errors that makes me to suspicious on your data. Rewrite the introduction part and discussion part seriously.

Author's Response:

- **Thanks, and we have amended all comments as per request and highlighted**

Reviewer #2 comments

1. The title of this manuscript is quite lengthy. Authors should consider shortening the title to a concise one. Please consider, "Multidrug resistance and associated risk factors of bacterial- and fungal UTI among HIV patients attending Antiretroviral Therapy Clinics of Northeast Ethiopia".

Author's Response:

- **Comments well taken and the manuscript corrected accordingly, thanks.**

2. The manuscript has a significant number of omissions, typographical and grammatical errors, and incoherent sentences. Authors will need to carefully go through the manuscript for corrections or employ the services of a script/copy editor.

Author's Response:

Comment addressed and highlighted, thanks

3. All genus and specie names of microorganisms should be italicized. This is not consistent in the manuscript.

Author's Response:

- **Comment addressed and amended, microorganisms should be italicized and highlighted .**

4. All abbreviations should be written in full in the places where they are first used. This is inconsistent in the manuscript.

Author's Response:

- **Thanks; we really appreciate the comment which helped us to see the gap. Hence, we revised the sentence , included it under and highlighted.**

5. In the introduction, the authors highlight that 'no related study was done in the study area in particular' and hence the need for this study. It would be appropriate to make reference to findings of similar studies done in other sites of similar settings in;
A.Ethiopia,
B. Resource-limited areas of the East African Subregion.

Author's Response:

- **Thanks for critical comments we have to search as we can and we siad that there is limited research in the study area means around dessie comprehensive specialized catchment area.**

6. Additionally, the authors should enumerate the specific objectives of their study in the Introduction

Author's Response:

Comment addressed and amended, thanks. The main objective covers the specific objectives. this study was to determine the bacterial and fungal profiles, multidrug resistance profiles of bacterial pathogens, and associated risk factors for community-acquired urinary tract infection among HIV positive patients

attending Antiretroviral Therapy Clinics in Northeast Ethiopia. If we write every spesfic objective it idea redundancy.

7. **The literature review provided for MDR in the Introduction is scanty.**

<u>Author's Response:</u>

- **Thanks for interesting Comments and we are addressed like Many research studies in the world and Africa, including different parts of Ethiopia, showed that more than 50% of uro-pathogenic bacteria developed multidrug resistance (MDR). About 74% of uro-pathogenic bacteria (6) 70.0% of bacteria from India (7), 79.3% of uro-pathogenic bacterial isolates from Asia Wolayta Sodo Ethiopia (8), in Addis Ababa, 78.4% of Ethiopians (9) and in Gondar, Ethiopia, 91.7% (10) developed MDR thanks**

8. The Introduction should include at least, a sentence or a paragraph on each of the following;

a. Definition, Symptoms and Types of UTI
b. Epidemiology of UTI in Ethiopia, East Africa, and Sub-saharan Africa
c. Epidemiology of HIV in Sub-saharan Africa and Ethiopia
d. Prevalence and Risk of UTI among HIV patients
e. Factors associated with urinary tract infections among HIV Patients (please see Skrzat-Klapaczyńska A, Matłosz B, Bednarska A, et al. Factors associated with urinary tract infections among HIV-1 infected patients. PLoS One. 2018;13(1):e0190564. Published 2018 Jan 11. doi:10.1371/journal.pone.0190564)

<u>Author's Response</u>:

- Thanks for motivating Comments and we are adding the definitions of UTI as follows Urinary tract infection (UTI) is an infection of urinary tract, including the kidneys, ureters and bladder, urethra, and accessory structures that collect and store urine and release it from the body. It occurs when microorganisms, typically bacteria from the digestive tract, cling to the urethral opening and begin to multiply. Globally, the burden of UTI is estimated to be 150 million cases every year (3). Based on previous epidemiological studies from Tanzania and elsewhere in sub-Saharan Africa, the urinary tract infections was estimated at 35–45% (4). In Ethiopia the pooled prevalence of urinary tract infection was 15.97%(5). Urinary tract infections in HIV patients in Ethiopia was 12.8%
- And we have added the recommended reference An other study on Factors associated with urinary tract infections among HIV-1 infected patients also showed that low CD4 count, chronic hepatitis, being female have been linked to occurrence of bacterial UTI (22)

9. Please provide a reference to this statement in the Discussion (pg 15, paragraph 1) ; Multiple studies showed that within hospital settings at least 10–15% of hospital-acquired UTIs are caused by Candida species and Candidauria is especially common in intensive care units

<u>Author's Response</u>:

- Thanks for motivating Comments you can observe reference number 47 ,22 and 48

10. The section in the Study site description can be shortened, Titles of Tables can be shortened

Author's Response:

all The titles are modified to Uropathogens, multidrug resistance pattern of bacterial and associated factors of community-acquired urinary tract infection among HIV positive patients attending Antiretroviral Therapy Clinics of Northeast Ethiopia and Section of study are shortened based on the comments

the table name and figure legends are corrected based on recommendations.

Finally, we thank you for your critical review of the paper.

Sincerely,

Mihret Tilahun
