# Supplementary material for: Uro-pathogens: Multidrug resistance and associated factors of community-acquired UTI among HIV patients attending antiretroviral therapy in Dessie Comprehensive Specialized Hospital, Northeast Ethiopia
Source: PLoS One. 2024 May 31;19(5):e0296480. doi: 10.1371/journal.pone.0296480 (PMC11142584; doi:10.1371/journal.pone.0296480)
Supplement: Attachment — Submitted filename: Response to Reviewers.docx [file pone.0296480.s002.docx]

**Author’s Response to Editor and Reviewer comments**

**Manuscript ID number:**
**PONE-D- 22-31717**

**Title of paper:**

Bacterial and fungal profile, multidrug resistance profile of bacterial pathogens and associated risk factors of community-acquired urinary tract infection among HIV positive patients attending Antiretroviral Therapy Clinics of Northeast Ethiopia

**General**

We appreciate all of the constructive feedback on our manuscript from the reviewers, which has helped us improve the paper's clarity and scientific plausibility. We thank the PLOS ONE scholarly editors for their insightful comments and for allowing us to update the text. All of the concerns raised by the reviewers and editors have been addressed, and the text has been updated as a result. Furthermore, both the updated article and the response letter address all of the editorial revisions that are required. The changes are shown with track changes in the file labelled "Revised Manuscript with Track Modifications." The following is a detailed rebuttal.

**Editor Comments:**

1. Please ensure that your manuscript meets PLOS ONE's style requirements, including those for file naming. The PLOS ONE style templates can be found at https://journals.plos.org/plosone/s/file?id=wjVg/PLOSOne_formatting_sample_main_body.pdf and <https://journals.plos.org/plosone/s/file?id=ba62/PLOSOne_formatting_sample_title_authors_affiliations.pdf>

***Author’s Response:***

- **Thanks for the substantial editorial comment. The revised manuscript is now updated, highlighted and meets PLOS ONE's style requirements, including those for file naming.**

2. We noticed you have some minor occurrence of overlapping text with the following previous publication(s), which needs to be addressed: - <http://etd.aau.edu.et/bitstream/handle/123456789/6042/Yemisrach%20Getu.pdf>? In your revision ensure you cite all your sources (including your own works), and quote or rephrase any duplicated text outside the methods section. Further consideration is dependent on these concerns being addressed
   ***Author’s Response:***

- **Thank you for the interesting comment. Minor over lapping’s are corrected**

3. We suggest you thoroughly copyedit your manuscript for language usage, spelling, and grammar. If you do not know anyone who can help you do this, you may wish to consider employing a professional scientific editing service.

” ***Author’s Response:***

- **Thank you and corrections are made as per the comment; all the authors are senior expert and published many papers and one of the authors Melaku Ashagire Belete is the editors of plose one. So no need of language editors.**

4. Thank you for stating the following financial disclosure: “All the authors are under developing countries and no extrena funding was obtained” At this time, please address the following querie.

***Author’s Response****:*

- **Thank you for the interesting comment.** **financial disclosure corrected as No external funds were obtained only internal support was obtained from Wollo university. The funders had no role in study design, data collection and analysis, decision to publish, or preparation of the manuscript**

**Reviewer #1 comments**

Reviewer #1: Check the sticky note on your PDF file. Try to address all the comments. I have tried to your entire manuscript it lacks basic thing. if modified and edited with expert in the field it match improved. But I saw some errors that makes me to suspicious on your data. Rewrite the introduction part and discussion part seriously.

***Author’s Response:***

- **Thanks, and we have amended all comments as per request and highlighted and some questions and comments are here below**

**The date was collected from patients before 48 hours. The data written 18 hours is clerical error**

1. Write Bacterial and fungal profile as uropathogens, multidrug resistance profile rephrase of bacterial pathogens and associated risk factors as associated factorstof community-acquired urinary tract infection among HIV positive patients attending Antiretroviral Therapy

Clinics of Northeast Ethiopia

***Author’s Response***

**We modify the title Uropathogens, multidrug resistance pattern of bacterial and associated factors of community-acquired urinary tract infection among HIV positive patients attending Antiretroviral Therapy Clinics of Northeast Ethiopia.**

2. Source of standardized questionaries was where? And why not performed Recent CD4 count?

***Author’s Response:***

- Thanks for the vivid comment. The source of questionaries or we were developed the questionaries by modifications from WHO Suspected Urinary Tract Infection patient questionnaire and from other published articles
- CD4+ cell counts are usually measured when you are diagnosed with HIV (at baseline), every 3 to 6 months during first 2 years or until your CD4 count increases above 300 cells/mm3. So it is better to take from their medical records those who have less than six months

3. Can you justify do you collect mid steam urine or catheterized patients? What about significance .

***Author’s Response:***

- **Thank you for the substantial comment. We have** **collected mid-stream urine and symptomatic patients so the interpretations are** **Symptomatic ambulatory patient; voided or clean-catch specimen: report if growth is ≥ 10,000 CFU/ml with 1-2 species of potential pathogen.**

4. **On the result section all comments are corrected per comments and sugestions but the same isolates are 74 not 75 ?**

***Author’s Response:***

- **Thanks for the valuable Comment and you can calculated and subtract again 92-17 =75**

5. **Result and discussion**: how do you identify C.tropicalis from C.albican

***Author’s Response:***

- **Thank you for the valuable comment. Gram stain, Germ Tube Test, Carbohydrate Fermentation, and Assimilation Tests, as well as chromogenic medium (CHROMagar Candida Medium (Biomerieux, France) according to the manufacturer's instructions .**
- **Perry and Miller reported that Candida albicans produces an enzyme b -N-acetyl- galactosaminidase and according to Rousselle et al incorporation of chromogenic or fluorogenic hexosaminidase substrates into the growth medium helps in identification of C. albicans isolates directly on primary isolation. HiCrome Candida Differential Agar is a selective and differential medium, which facilitates rapid isolation of yeasts from mixed cultures and allows differentiation of Candida species namely C. albicans, C. krusei, C. tropicalis and C. glabrata on the basis of coloration and colony morphology. On this medium results are obtained within 48 hours and it is useful for the rapid and presumptive identification of common yeasts in Mycology and Clinical Microbiology Laboratory. Peptone special and yeast extract provides nitrogenous, carbonaceous compounds and other essential growth nutrients. Phosphate buffers the medium well. Chloramphenicol suppresses the accompanying bacterial flora. C. albicans appear as light green colored smooth colonies, C. tropicalis appear as blue to metallic blue colored raised colonies. C. glabrata colonies appear as cream to white smooth colonies, while C. krusei appear as purple fuzzy colonies.**
-

6. Rewrite MDR based on guideline of for table construct from journal for submisions.

***Author’s Response:***

- **Thanks, and we have amended the conclusion part as we write on the A bacterium that is simultaneously resistant for three or more antimicrobial categories. I understand your questions but to write each bacteria it is very length.**

7. **Using references 23 From Hawassa are not ART**

**Author’s Response:**

**We use Hawassa non-HIV patients as a reference because both HIV and Diabetics are chronic diseases and they lead patients immunocompromised**

Reviewer #1: Check the sticky note on your PDF file. Try to address all the comments. I have tried to your entire manuscript it lacks basic thing. if modified and edited with expert in the field it match improved. But I saw some errors that makes me to suspicious on your data. Rewrite the introduction part and discussion part seriously.

***Author’s Response:***

- **Thanks, and we have amended all comments as per request and highlighted**

**Reviewer #2 comments**

1. The title of this manuscript is quite lengthy. Authors should consider shortening the title to a concise one. Please consider, “Multidrug resistance and associated risk factors of bacterial- and fungal UTI among HIV patients attending Antiretroviral Therapy Clinics of Northeast Ethiopia”.

***Author’s Response:***

- **Comments well taken and the manuscript corrected accordingly, thanks.**

2. The manuscript has a significant number of omissions, typographical and grammatical errors, and incoherent sentences. Authors will need to carefully go through the manuscript for corrections or employ the services of a script/copy editor.

**Author’s Response:**

Comment addressed and highlighted, thanks

3. All genus and specie names of microorganisms should be italicized. This is not consistent in the manuscript.

***Author’s Response:***

- **Comment addressed and amended, microorganisms should be italicized and highlighted .**

4. All abbreviations should be written in full in the places where they are first used. This is inconsistent in the manuscript.

***Author’s Response:***

- **Thanks; we really appreciate the comment which helped us to see the gap. Hence, we revised the sentence , included it under and highlighted.**

5. In the introduction, the authors highlight that ‘no related study was done in the study area in particular’ and hence the need for this study. It would be appropriate to make reference to findings of similar studies done in other sites of similar settings in;
   A.Ethiopia,
   B. Resource-limited areas of the East African Subregion.

***Author’s Response:***

- **Thanks for critical comments we have to search as we can and we siad that there is limited research in the study area means around dessie comprehensive specialized catchment area.**

6. **Additionally, the authors should enumerate the specific objectives of their study in the Introduction**

***Author’s Response:***

**Comment addressed and amended, thanks. The main objective covers the specific objectives. this study was to determine the bacterial and fungal profiles, multidrug resistance profiles of bacterial pathogens, and associated risk factors for community-acquired urinary tract infection among HIV positive patients attending Antiretroviral Therapy Clinics in Northeast Ethiopia. If we write every spesfic objective it idea redundancy.**

7. **The literature review provided for MDR in the Introduction is scanty.**

**Author’s Response:**

- **Thanks for interesting Comments and we are addressed like Many research studies in the world and Africa, including different parts of Ethiopia, showed that more than 50% of uro-pathogenic bacteria developed multidrug resistance (MDR). About 74% of uro-pathogenic bacteria (6) 70.0% of bacteria from India (7), 79.3% of uro-pathogenic bacterial isolates from Asia Wolayta Sodo Ethiopia (8), in Addis Ababa, 78.4% of Ethiopians (9) and in Gondar, Ethiopia, 91.7% (10) developed MDR thanks**

8. The Introduction should include at least, a sentence or a paragraph on each of the following;
   a. Definition, Symptoms and Types of UTI
   b. Epidemiology of UTI in Ethiopia, East Africa, and Sub-saharan Africa
   c. Epidemiology of HIV in Sub-saharan Africa and Ethiopia
   d. Prevalence and Risk of UTI among HIV patients
   e. Factors associated with urinary tract infections among HIV Patients (please see Skrzat-Klapaczyńska A, Matłosz B, Bednarska A, et al. Factors associated with urinary tract infections among HIV-1 infected patients. PLoS One. 2018;13(1):e0190564. Published 2018 Jan 11. doi:10.1371/journal.pone.0190564)

**Author’s Response:**

- Thanks for motivating Comments and we are adding the definitions of UTI as follows Urinary tract infection (UTI) is an infection of urinary tract, including the kidneys, ureters and bladder, urethra, and accessory structures that collect and store urine and release it from the body. It occurs when microorganisms, typically bacteria from the digestive tract, cling to the urethral opening and begin to multiply. Globally, the burden of UTI is estimated to be 150 million cases every year (3). Based on previous epidemiological studies from Tanzania and elsewhere in sub-Saharan Africa, the urinary tract infections was estimated at 35–45% (4). In Ethiopia the pooled prevalence of urinary tract infection was 15.97%(5). Urinary tract infections in HIV patients in Ethiopia was 12.8%
- And we have added the recommended reference An other study on Factors associated with urinary tract infections among HIV-1 infected patients also showed that low CD4 count, chronic hepatitis, being female have been linked to occurrence of bacterial UTI (22)

9. Please provide a reference to this statement in the Discussion (pg 15, paragraph 1) ; Multiple studies showed that within hospital settings at least 10–15% of hospital-acquired UTIs are caused by Candida species and Candidauria is especially common in intensive care units
   **Author’s Response:**

- Thanks for motivating Comments you can observe reference number 47 ,22 and 48

10. The section in the Study site description can be shortened, Titles of Tables can be shortened

**Author’s Response:**

all The titles are modified to Uropathogens, multidrug resistance pattern of bacterial and associated factors of community-acquired urinary tract infection among HIV positive patients attending Antiretroviral Therapy Clinics of Northeast Ethiopia and Section of study are shortened based on the comments

the table name and figure legends are corrected based on recommendations.

**Finally, we thank you for your critical review of the paper.**

Sincerely,

Mihret Tilahun
