# Supplementary material for: Uro-pathogens: Multidrug resistance and associated factors of community-acquired UTI among HIV patients attending antiretroviral therapy in Dessie Comprehensive Specialized Hospital, Northeast Ethiopia
Source: PLoS One. 2024 May 31;19(5):e0296480. doi: 10.1371/journal.pone.0296480 (PMC11142584; doi:10.1371/journal.pone.0296480)
Supplement: Attachment — Submitted filename: Review report.docx [file pone.0296480.s005.docx]

**Review report**

Tilahun M et al., assessed the uropathies and associated factors in HIV patients under therapy. Such kinds of studies would be helpful to manage immunosuppression associated coinfections causing high morbidity in these populations. Though, the article needs to be revised in many section which I detailed as follows.

**Title**

I advise the title not to have the word “risk factors” instead better to use associated factors. And it is better to say “Uropathogens: multidrug resistance and risk factors of community-acquired

UTI among HIV patients attending antiretroviral therapy in Dessie Comprehensive specialized Hospital, Northeast Ethiopia”. You conducted the study in just a single site and thus the title should be specific.

**Abstract**

Acronyms should be written in full format in first appearance like UTI. Antiretroviral clinics “of/in” in Dessie comprehensive specialized hospital, northeast Ethiopia….

The abstract lacks statement about fungi. How fungi were recovered. Don’t neglect fungi like the healthcare settings in the country.

From the total of…. Why only MDR? Why not drug resistance? 81.3% of bacteria were MDR? What a shocking result. Are you serious?

Is CD4 count is still there to classify immunosuppression? Urgency of urination can’t be associated factor. It comes due to infection.

**Introduction**

We can’t say no study anytime. Better to say…not enough study, no published data, scarcity of published documents etc…, still the introduction ignores fungi.

**Methods**

The last sentence of study design section is incomplete.

What about antimicrobially treated patients? Did you include them?

Please note that UTI symptoms you mentioned can’t be associated factors. Rather they are symptoms of UTI.

The reference study you used for sample size calculation is not relevant to your study. Their population is paediatric UTI patients and unknown of their HIV status? How you reconcile this?

Where you did the pre-test and in how many peoples?

Do you have PC-II certified facility for fungal culture, especially in case where sporulation would happen?

**Results**

Avoid response rate etc. because you used non random sampling. When writing result if you start with the word…about… then a percentage should be followed. Otherwise better to write full name of numbers. For example, instead of saying about 105 (while it is exactly 105), saying…one hundred and five or about 30% (105/346)….sounds good.

Another important issue is that the prevalence of UTI and bacteria/fungi shouldn’t be similar. Because there may be nonbacterial/non fungal cause of UTI including anaerobic bacteria. If the number of people with UTI and number of bacteria/fungi is similar, 1) there is a single bacteria/fungi isolated from each patient which is unlikely 2) all UTI are caused by bacteria and fungi (again unlikely). Thus, don’t say the prevalence of UTI. Rather, among 346 HIV patients, 92 were clinically confirmed to have UTI and of these bacteria were isolated from 105 clinically confirmed UTI patients.

The AST result should only talk about MDR as your objective is MDR. Why was methicillin resistance not assessed for CONS?

Avoid repeated data….for example…peoples with chronic diabetes.. is repeated.

Symptoms of UTI should be removed as factors throughout the document.

**Discussion**

Trim the discussion to be specific to your objectives. No need to compare to much data. These days, discussions are recommended to be specific and focused.

**Conclusion**

Remove summary of results. Figures are not good to mention in conclusion. Just decide the conclusion based on the results. Like…based on the results, bacteria/fungal UTI was significantly high/low (as compared to national or local data). Bacteria especially …..were MDR… and thus empirical therapy should be discouraged,,, etc… factors like….significantly increased bacterial/fungal UTI. Therefore public health measures…..recommended.

Overall, the paper has been substantially revised from the first revision. Yet, it needs language editions and grammatical corrections. Besides, moderate revisions are recommended before publication.
