# Supplementary material for: Uro-pathogens: Multidrug resistance and associated factors of community-acquired UTI among HIV patients attending antiretroviral therapy in Dessie Comprehensive Specialized Hospital, Northeast Ethiopia
Source: PLoS One. 2024 May 31;19(5):e0296480. doi: 10.1371/journal.pone.0296480 (PMC11142584; doi:10.1371/journal.pone.0296480)
Supplement: Attachment — Submitted filename: Response letter .docx [file pone.0296480.s007.docx]

**Author’s Response to Editor and Reviewer comments**

**Manuscript ID number:**
**PONE-D- 22-31717**

**Title of paper:**

**Uro-pathogens: Multidrug resistance and associated factors of community-acquired UTI among HIV patients attending antiretroviral therapy in Dessie Comprehensive Specialized Hospital, Northeast Ethiopia**
 **General**

We appreciate all of the constructive feedback on our manuscript from the reviewers, which has helped us improve the paper's clarity and scientific plausibility. We thank the PLOS ONE scholarly editors for their insightful comments and for allowing us to update the text. All of the concerns raised by the reviewers and editors have been addressed, and the text has been updated as a result. Furthermore, both the updated article and the response letter address all of the editorial revisions that are required. The changes are shown with track changes in the file labelled "Revised Manuscript with Track Modifications." The following is a detailed rebuttalTilahun M et al., assessed the uropathies and associated factors in HIV patients under therapy. Such kinds of studies would be helpful to manage immunosuppression associated coinfections causing high morbidity in these populations. Though, the article needs to be revised in many sections which I detailed as follows.

**Title**

I advise the title not to have the word “risk factors” instead better to use associated factors. And it is better to say “Uropathogens: multidrug resistance and risk factors of community-acquired

UTI among HIV patients attending antiretroviral therapy in Dessie Comprehensive specialized Hospital, Northeast Ethiopia”. You conducted the study in just a single site and thus the title should be specific.

***Author’s Response***

***We modify the title to Uro-pathogens: Multidrug resistance and associated factors of community-acquired UTI among HIV patients attending antiretroviral therapy in Dessie Comprehensive Specialized Hospital, Northeast Ethiopia***

**Abstract**

Acronyms should be written in full format in first appearance like UTI. Antiretroviral clinics “of/in” in Dessie comprehensive specialized hospital, northeast Ethiopia….

The abstract lacks statement about fungi. How fungi were recovered. Don’t neglect fungi like the healthcare settings in the country.

From the total of…. Why only MDR? Why not drug resistance? 81.3% of bacteria were MDR? What a shocking result. Are you serious?

Is CD4 count is still there to classify immunosuppression? Urgency of urination can’t be associated factor. It comes due to infection.

**Author response**

- Thanks for the critical comments the abstract is corrected based on recommendations Urinary tract infections are common bacterial and fungal infections in humans, occurring both in the community and in immunocompromised patients in healthcare settings. Urinary tract infections have a significant health impact on HIV-infected patients. Nowadays, drug-resistant pathogens are widespread poses a serious clinical risk, and causes urinary tract infection. The common agents of bacteria and fungi that cause urinary tract infection are *Escherichia coli* followed by *Klebsiella pneumonia*, *Staphylococcus saprophyticus, Enterococcus faecalis*, *group B streptococcus*, *Proteus mirabilis*, *Pseudomonas aeruginosa*, *Staphylococcus aureus* and *Candida. albicans*. This study aimed to investigate uro-pathogen, multidrug resistance pattern of bacteria, and associated factors of community-acquired urinary tract infection among HIV-positive patients attending antiretroviral therapy in Dessie comprehensive specialized hospital, Northeast Ethiopia from February 1, 2021, to March 30, 2021

**Introduction**

We can’t say no study anytime. Better to say…not enough study, no published data, scarcity of published documents etc…, still the introduction ignores fungi.

- **Author response**

Thanks for the critical comments we have added about fungus and you can see it. Candidauria is the presence of Candida spp. in the urine, such as C. albicans. Patients with cystitis, epididymorchitis, prostatitis, pyelonephritis, and renal candidiasis experience symptomatic candidiasis (10). C. albicans is one of the most common fungi that can cause Candidauria (20% of nosocomial infections

**Methods**

The last sentence of study design section is incomplete.

What about antimicrobials treated patients? Did you include them?

Please note that UTI symptoms you mentioned can’t be associated factors. Rather they are symptoms of UTI.

The reference study you used for sample size calculation is not relevant to your study. Their population is paediatric UTI patients and unknown of their HIV status? How you reconcile this?

Where you did the pre-test and in how many peoples?

Do you have PC-II certified facility for fungal culture, especially in case where sporulation would happen?

**Author response**

The methology sections were corrected based on the recommendations of the reviewer and the PC-II certified facility for fungal culture, especially in case where sporulation would happen was certified by Ethiopian public health institute and there is BSC class cabinet in our lab and we performed the laboratory procedure with in this cabinet.

**Results**

Avoid response rate etc. because you used non random sampling. When writing result if you start with the word…about… then a percentage should be followed. Otherwise, better to write full name of numbers. For example, instead of saying about 105 (while it is exactly 105), saying…one hundred and five or about 30% (105/346) …. sounds good.

Another important issue is that the prevalence of UTI and bacteria/fungi shouldn’t be similar. Because there may be nonbacterial/non fungal cause of UTI including anaerobic bacteria. If the number of people with UTI and number of bacteria/fungi is similar, 1) there is a single bacteria/fungus isolated from each patient which is unlikely 2) all UTI are caused by bacteria and fungi (again unlikely). Thus, don’t say the prevalence of UTI. Rather, among 346 HIV patients, 92 were clinically confirmed to have UTI and of these bacteria were isolated from 105 clinically confirmed UTI patients.

The AST result should only talk about MDR as your objective is MDR. Why was methicillin resistance not assessed for CONS?

Avoid repeated data…. for example…peoples with chronic diabetes. is repeated.

Symptoms of UTI should be removed as factors throughout the document.

**Author response**

We correct the result based on recommendations In this study, a total of 346 HIV-positive patients were included. The age of the study participants ranged from 21 to 80 years with a mean (±SD) age of 39.44 (±10.87) years, and 116 (33.5%) of them were in the age range of 35–44 years. About 52.02% (180/346) of the respondents were females. Regarding educational status, 33.2% (115/346) of the respondents had a primary level of education. Moreover, 26.01% (90/346) were self-employed and 62.1% (215/346) of them were urban dwellers

Among 346 HIV patients, 92 (26.6%) were clinically confirmed to have UTI and of these bacteria were isolated from 75 clinically confirmed UTI patients and 17 were fungal. Among 75 bacterial isolates, the most frequent bacterium was E. *coli* which accounted for 21.33% (16/75) followed by *K. pneumoniae*, *enterococcus species*, and *P. aeruginosa*, accounting for 14.67% (11/75), 13.33% (10/75), and 12% (9/75), respectively. The least number of bacterial isolates were *Enterobacter cloacae*, which accounted for 5.33% (4 /75). Gram-positive bacteria accounted for only 32% (24/75) of the bacterial isolates, of which 41.67% (10/24) of the isolates were *Enterococcus species*. Of 17 candida isolates, 47.1% (8/17) were represented by *C. tropicalis*

**Discussion**

Trim the discussion to be specific to your objectives. No need to compare to much data. These days, discussions are recommended to be specific and focused.

**Author response**

We correct the disunions based on recommendations In the current study, urinary tract infection with fungal was 4.9% (17/346). Of the 17 fungal isolates, 47.1% (8/17) were represented by C. tropicalis. The study reported on C. albicans, C. glabrata, and C. tropicalis supported the current study with the report of fungal species (46). Studies showed that within hospital settings at least 11.3 % of community-acquired UTIs are caused by candida species (47). In a study conducted in Addis Ababa, Candidauria represented 7% of UTIs in which C. albicans was the leading cause and 81.1% of candida species were isolated (22), and a study was done in two microorganisms Candida spp. (Candida glabrata and Candida krusei).

**Conclusion**

Remove summary of results. Figures are not good to mention in conclusion. Just decide the conclusion based on the results. Like…based on the results, bacteria/fungal UTI was significantly high/low (as compared to national or local data). Bacteria especially …. were MDR… and thus empirical therapy should be discouraged, etc… factors like…. significantly increased bacterial/fungal UTI. Therefore, public health measures…. recommended.

Overall, the paper has been substantially revised from the first revision. Yet, it needs language editions and grammatical corrections. Besides, moderate revisions are recommended before publication.

**Author response**

We correct the conclusions based on recommendations The symptomatic community-acquired UTI caused by bacterial pathogens and fungi is high, as compared to different national and local data. Of the isolated pathogens, bacteria were high as compared to fungal pathogens. Among bacterial pathogens, E. coli is the most frequent bacterium, followed by K. pneumoniae, enterococcus species, and P. aeruginosa, accounting for, respectively. Of the isolated bacteria, 61(81.3%) were resistant to three and above classes of antibiotics (drug classes), and E. coli, K. pneumoniae, Enterococcus species, P. aeruginosa, and CoNs showed high levels of drug resistance. Most Gram-negative and Gram-positive isolates were resistant to tetracycline, trimethoprim-sulfamethoxazole, penicillin, chloramphenicol, and nitrofurantoin, respectively.
